# Generative Deep Learning-Based Efficient Design of Organic Molecules with Tailored Properties

**DOI:** 10.1021/acscentsci.4c00656

**Published:** 2024-08-30

**Authors:** Minhi Han, Joonyoung F. Joung, Minseok Jeong, Dong Hoon Choi, Sungnam Park

**Affiliations:** Department of Chemistry and Research Institute for Natural Science, Korea University, Seoul 02841, Korea

## Abstract

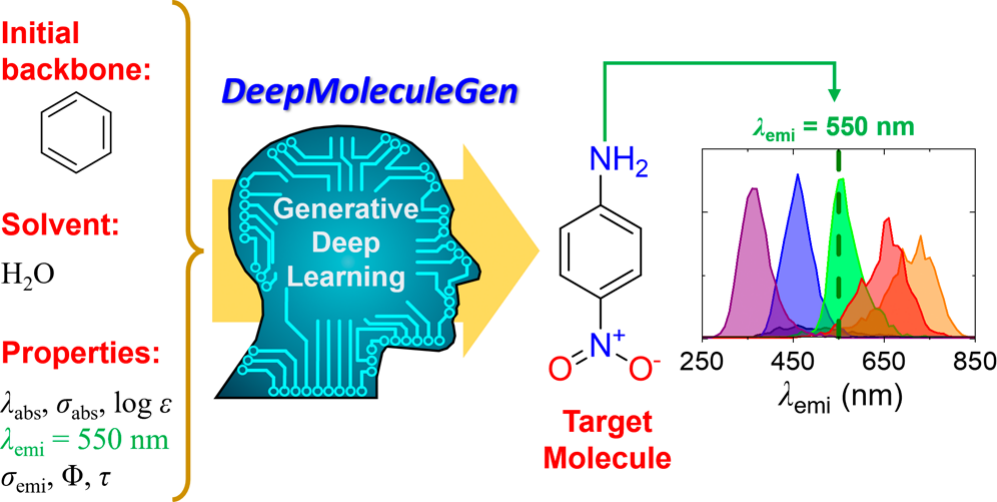

Innovative approaches to design molecules with tailored properties are required in various research areas. Deep learning methods can accelerate the discovery of new materials by leveraging molecular structure–property relationships. In this study, we successfully developed a generative deep learning (Gen-DL) model that was trained on a large experimental database (DB_exp_) including 71,424 molecule/solvent pairs and was able to design molecules with target properties in various solvents. The Gen-DL model can generate molecules with specified optical properties, such as electronic absorption/emission peak position and bandwidth, extinction coefficient, photoluminescence (PL) quantum yield, and PL lifetime. The Gen-DL model was shown to leverage the essential design principles of conjugation effects, Stokes shifts, and solvent effects when it generated molecules with target optical properties. Additionally, the Gen-DL model was demonstrated to generate practically useful molecules developed for real-world applications. Accordingly, the Gen-DL model can be a promising tool for the discovery and design of novel molecules with tailored properties in various research areas, such as organic photovoltaics (OPVs), organic light-emitting diodes (OLEDs), organic photodiodes (OPDs), bioimaging dyes, and so on.

## Abstract 2

Generative deep learning model (*DeepMoleculeGen*) was developed to generate optimal molecules in a given solvent, given the target properties, initial backbone, and solvent.

## I Introduction

The development of new molecules with tailored properties in chemistry and materials science has relied largely on expert knowledge and trial-and-error methods.^1,2^ However, this approach is limited in developing molecules with tailored properties due to the complex nature of molecules and the vast chemical space.^3^ Consequently, deep learning (DL) methods have emerged as a promising tool to effectively explore chemical space and develop the molecules with desired properties in an efficient and target-oriented manner.^4−9^ One approach to finding molecules with desired properties is to virtually generate a large number of molecules using various scaffolds, then use predictive DL models to predict their properties and select the optimal molecule based on the predicted properties.^3,4^ However, this approach still relies on human expertise in scaffold selection and may not fully exploit the chemical space to develop molecules with optimal properties.

In contrast, generative DL models can be a crucial tool to overcome the limitations of human expertise and to design molecules with target properties based on the underlying molecular structure–property relationship.^10−14^ Various generative DL models have been developed based on variational autoencoders (VAEs),^15−17^ generative adversarial networks (GANs),^18,19^ and recurrent neural networks (RNNs).^20−24^ Unlike predictive DL models, generative DL models allow designing new molecules without relying on prior knowledge, greatly expanding the scope of potential materials and open up new avenues of exploration in various research areas.^25−27^ In addition, generative DL models have proven to be successful in designing new molecules with certain optimal properties such as drug-likeness, solubility, and synthetic accessibility.^5,28,29^ Generative DL models have been extensively developed to design molecules with certain properties that are mostly related to drug discovery, such as molecular weight, solubility, and quantitative estimate of drug-likeness (QED). There have been needs for developing generative DL models that can be applicable to design fluorophores and chromophores used in organic light-emitting diodes (OLEDs), organic photovoltaics (OPVs), organic photodiodes (OPDs), and bioimaging. However, these generative DL models are challenging to develop due to the limited accessibility of experimental databases that can be used to train generative DL models, both in terms of the number of molecular structures and the diversity of molecular structures in the experimental databases.

In this study, we developed a generative DL (Gen-DL) model based on a large experimental database of optical properties of organic molecules. The experimental database contains a variety of organic molecules (with absorption and emission spectra ranging from UV to near-IR) and their optical properties in solutions: first absorption peak position (λ_abs_) and bandwidth (σ_abs_), extinction coefficient (*ε*), emission peak position (λ_emi_) and bandwidth (σ_emi_), photoluminescence quantum yield (PLQY, Φ), and photoluminescence lifetime (τ). Accordingly, the Gen-DL model can efficiently generate optimal organic molecules in a given solvent, given the target optical properties and solvent as input. In addition to the target optical properties, the solvent is given as an additional input because the optical properties of molecules are substantially influenced by the surrounding solvent molecules. The Gen-DL model was shown to generate molecules with target optical properties by leveraging the essential design principles such as conjugation effects, Stokes-shifts, and solvent effects. In addition, we demonstrated that the Gen-DL model indeed generated practically useful molecules that were found to be used as fluorophores for OLEDs,^30^ near-IR imaging dyes,^31^ fluorescent dyes,^32^ and photovoltaic materials.^33^

## II Results and Discussion

### II-A Experimental Database for DL Models

The electronic absorption properties (first absorption peak position, λ_abs_; absorption bandwidth, σ_abs_; extinction coefficient, ε) and emission properties (emission peak position, λ_emi_; emission bandwidth, σ_emi_; PLQY, Φ; PL lifetime, τ) can be readily characterized from experimental data (UV–visible absorption and PL spectra, and time-resolved fluorescence signal). We constructed an experimental database (DB_exp_) by collecting the aforementioned seven optical properties of organic molecules from research articles, comprising 71,424 molecule/solvent pairs, expanded from our previous study.^7,8^ To train and validate both Pred-DL and Gen-DL models, three different data sets (DB_Pred-DL_, DB_Gen-DL_, and DB_Test_) were used as shown in Figure S1. The experimental database was split into a training data set (DB_Pred-DL_) and a test data set (DB_Test_) in a 9:1 ratio based on molecular structures. DB_Pred-DL_ consisted of 62,629 molecule/solvent pairs with 23,997 unique organic molecules and was used to retrain the Pred-DL model.^7^ DB_Gen-DL_ included only molecules in solutions and host films by excluding the molecules in solid states and gas phases from DB_Pred-DL_. Additionally, the missing data points in DB_Gen-DL_ were filled using the values predicted by the Pred-DL model. Accordingly, DB_Gen-DL_ consisted of 56,579 molecule/solvent pairs containing 22,819 unique organic molecules in various solvents. DB_Test_ was used as the test data set for the Gen-DL model.

### II-B Generative DL Model

In this study, we successfully developed a Gen-DL model to generate organic molecules with target optical properties in a given solvent (See Figure S5 and S6 for more detailed information). The Gen-DL model takes the target optical properties (λ_abs_, σ_abs_, log ε, λ_emi_, σ_emi_, Φ, τ) and the solvent as input, and then generates molecules step by step by stochastically selecting the actions (i.e., addition, connection, and termination), finally producing the molecular structure with the target optical properties. The three actions are (i) addition of an atom, represented by a vector (with atomic number, formal charge, the number of explicit hydrogen atoms), using a proper bond (single bond, double bond, triple bond, and aromatic bond), (ii) connection of two atoms to make a bond between them, and (iii) termination of the generation process.

**Figure 1:**
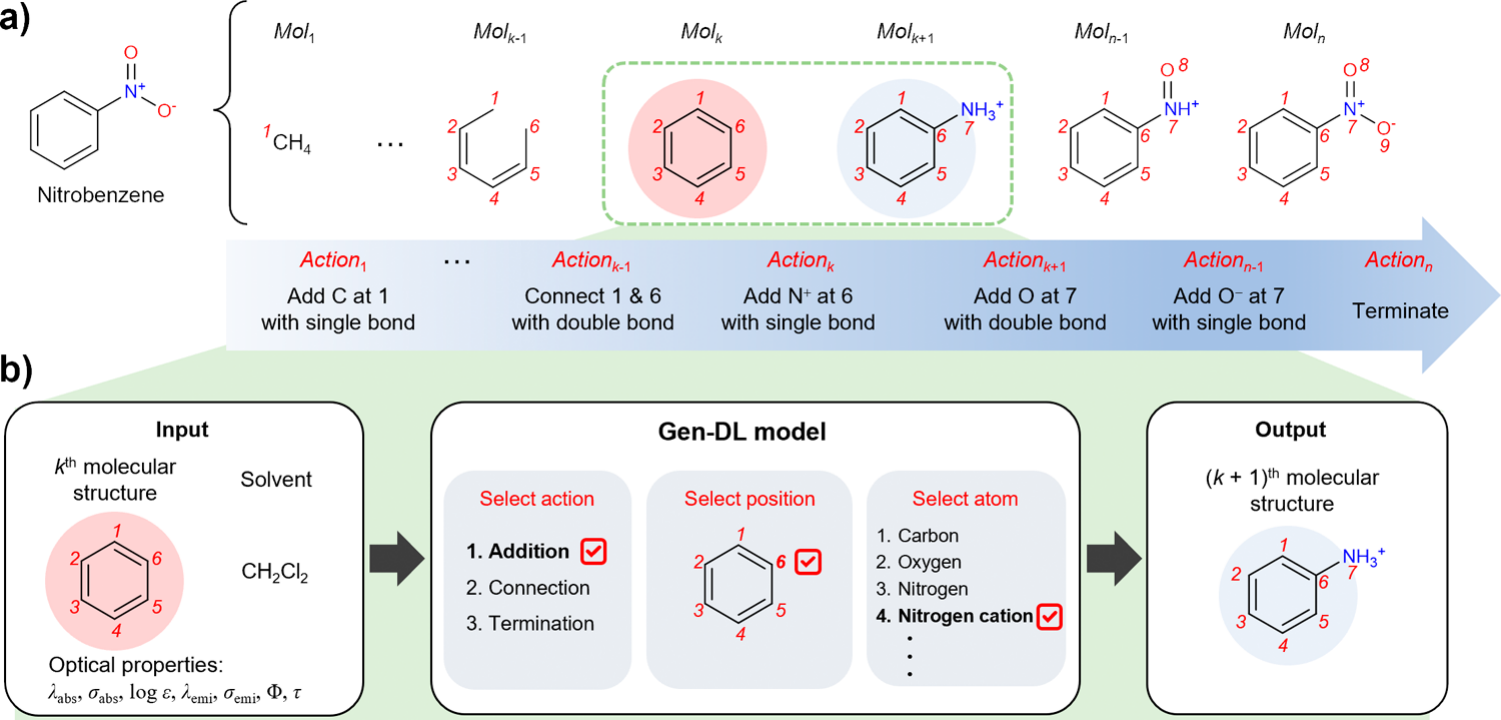
(a) Molecular generation sequence of an input molecule (nitrobenzene) is generated by the stochastic depth-first search algorithm. (b) Schematic illustration of training process of the Gen-DL model. The *k*^th^ molecular structure, solvent (CH_2_Cl_2_), and seven optical properties are used as inputs. The Gen-DL model learns how to generate the (*k* + 1)^th^ molecular structure from the *k*^th^ molecular structure in the sequence by calculating the probability of the next action (the addition of nitrogen cation at the 6 position).

To train the Gen-DL model, first the molecular generation sequence of the input molecules in the DB_Gen-DL_ are generated from scratch by a stochastic depth-first search algorithm.^34^ In the molecular generation process, hydrogen atoms are implicitly included in each molecule. Second, the molecular generation process for every step in the sequence is trained by calculating the probabilities of the next possible actions (addition, connection, and termination). As an example, Figure 1 shows how to train the Gen-DL model using nitrobenzene as an input molecule. The stochastic depth-first search algorithm generates the molecular generation sequence of nitrobenzene from scratch, showing how nitrobenzene can be generated step by step (Figure 1a). Figure 1b shows that the Gen-DL model learns how to generate the (*k* + 1)^th^ molecular structure from the *k*^th^ molecular structure in the sequence by calculating the probability of the next action (the addition of nitrogen cation at the 6 position) under the given condition of solvent (CH_2_Cl_2_) and optical properties. Therefore, during the training process, the Gen-DL model learns how to generate appropriate molecular structures with target properties in a given solvent (See the Supporting Information (SI) for details).

**Figure 2:**
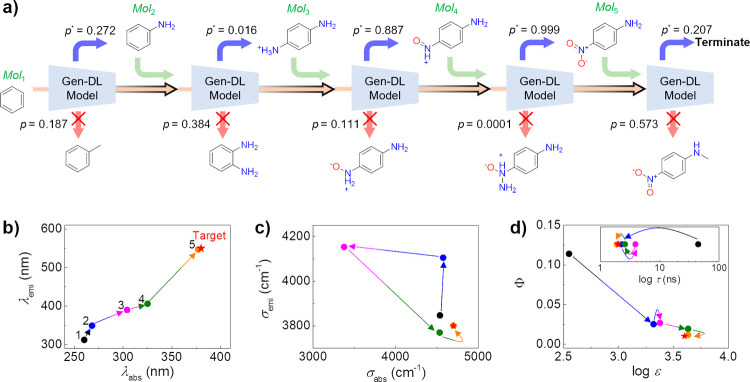
(a) Molecular generation process by the Gen-DL model, given three inputs: initial scaffold (benzene), solvent (H_2_O), and target properties (λ_abs_ = 380 nm, σ_abs_ = 4700 cm^–1^, log ε = 3.6, λ_emi_ = 550 nm, σ_emi_ = 3800 cm^–1^, Φ = 0.01, and τ = 2.0 ns). Gen-DL model generates molecular structures step by step, from the initial scaffold (benzene) to the final molecular structure (*p*-nitroaniline), by calculating the probability of the next possible actions. The probabilities for the selected and nonselected next actions are p* and *p*, respectively. (b, c, d), Changes in the optical properties of the molecular structures generated by the Gen-DL model during the molecular generation process (indicated by the arrows). The target properties are marked with red stars.

After the Gen-DL model is trained, it can generate molecular structures that meet the target optical properties in a given solvent, starting from scratch or from an initially given scaffold. The initial scaffold can be any atoms or any initial molecular backbone structures. During the molecular generation process, the Gen-DL model generates molecular structures step by step by calculating the probabilities of the next possible actions (addition, connection, and termination), finally producing the molecular structure with the target optical properties (Figure 1). Since the Gen-DL model stochastically selects the next possible action, it can select less likely actions, ensuring the diversity of molecular structures generated by the Gen-DL model. Therefore, each time molecular generation is performed, different molecular structures with the same target optical properties can be generated. Figure 2 shows the molecular generation process performed by the Gen-DL model and the changes in the optical properties of the molecular structures generated by the Gen-DL model during the molecular generation process, starting from the initial scaffold (benzene) to the final molecule (*p*-nitroaniline).

### II-C Efficient Generation of Molecules with Tailored Properties by the Gen-DL Model

The performance of our Gen-DL model was examined by generating the molecules with different sets of seven optical properties in different solvents. Note that some of the seven optical properties of molecules were found to be dependent in some sense, as shown in Figure S3. For example, λ_abs_ correlates well with λ_emi_ in that λ_abs_ increases with λ_emi_, but λ_abs_ is usually smaller than λ_emi_ for the given molecules due to the Stokes shift. In addition, σ_abs_ also correlates with σ_emi_. The PLQY (Φ) does not seem to be correlated with any other optical properties. Based on the correlation between optical properties, a target set of seven optical properties was selected for generating new molecules. As shown in Table S2, we examined 21 sets of seven optical properties in five different solvents: toluene, water, acetonitrile (ACN), tetrahydrofuran (THF), and dichloromethane (DCM), ultimately generating 105 sets of molecules with seven optical properties.

It should be noted that the optical properties of the molecules generated by the Gen-DL model were evaluated by our Pred-DL model. As described elsewhere in detail,^8^ we developed a Pred-DL model termed Deep Learning Optical Spectroscopy (DLOS) to predict seven optical properties of organic molecules in solutions, solid states, and gas phases. Since we first reported our initial Pred-DL model, the experimental database has significantly expanded to include a greater diversity of molecular structures and the Pred-DL model has been retrained, thereby improving the Pred-DL model in terms of accuracy and generalization. The performance of the current Pred-DL model is summarized in Figure S2 and Table S1.

**Figure 3:**
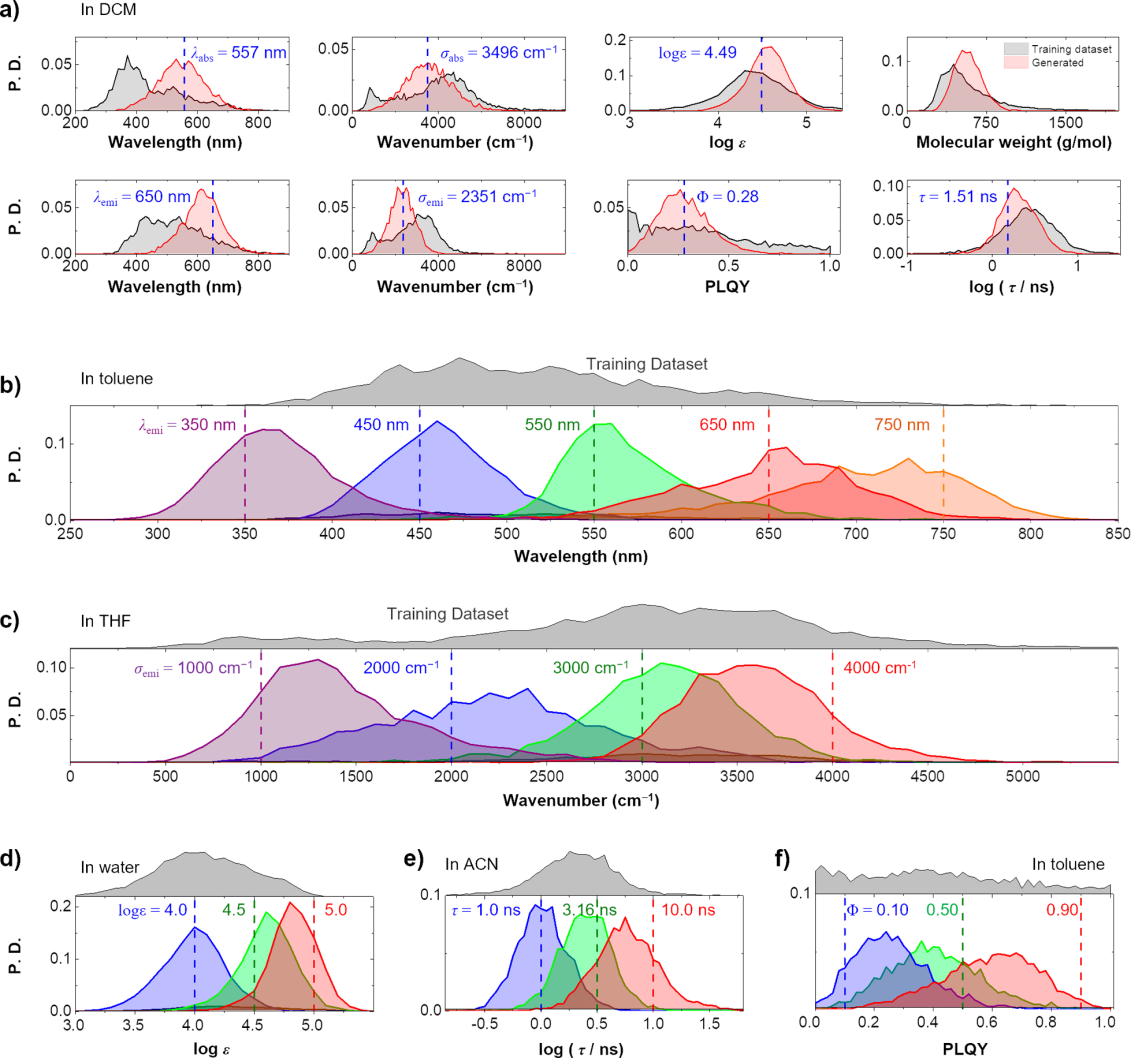
Property-oriented molecular generation by the Gen-DL model. (a) Distribution of the optical properties of generated molecules for given target optical properties (λ_abs_ = 557 nm, σ_abs_ = 3496 cm^–1^, log ε = 4.49, λ_emi_ = 650 nm, σ_emi_ = 2351 cm^–1^, Φ = 0.28, and τ = 1.51 ns) in DCM. The target properties are indicated by dashed vertical lines. The distributions of the optical properties of molecules in DB_Gen-DL_ (DCM) are shown in gray. The distributions of the optical properties of the generated molecules (in DCM) are shown in red. The distributions of the optical properties (b) λ_emi_, (c) σ_emi_, (d) log ε, (e) τ, and (f) Φ of generated molecules. The target optical properties are indicated by dashed vertical lines. The distribution of the optical properties of molecules in DB_Gen-DL_ (DCM) is shown in gray. See Figure S9–S113 for the distributions of the optical properties of generated molecules for different target sets of seven optical properties.

For a given target set of seven optical properties in a solvent, 10,000 molecules were generated by the Gen-DL model. The optical properties of the molecules were estimated by the Pred-DL model (see the additional SI files). Note that the generated molecules whose estimated optical properties were within the error range of the Pred-DL model were considered to be the ones that satisfied the target optical properties in a given solvent (these molecules were designated as molecules with target optical properties, *M*_TOP_). The Gen-DL model was found to exhibit excellent performance in generating *M*_TOP_ in a given solvent (%*M*_TOP_ = ∼ 4.4%), as shown in Figure S9–S113. In addition, our Gen-DL model was found to generate molecules with 88.9% validity, 47.1% uniqueness, and 44.4% novelty (see the SI for details). Figure 3a shows the distributions of seven optical properties of the molecules generated by the Gen-DL model in dichloromethane (DCM). Compared with the distributions of the optical properties of all molecules in DB_Gen-DL_, the seven optical properties of the generated molecules show narrow distributions close to the target optical properties.

#### II-C-1 Absorption and Emission Peak Positions

The absorption and emission peak positions of organic molecules are responsible for the daylight color and the emission color, respectively. Figure 3b shows the remarkable sensitivity of our Gen-DL model in generating molecules with target λ_emi_ value. As the target λ_emi_ value changes from ultraviolet (350 nm) to near-infrared (750 nm), the distribution of λ_emi_ for the generated molecules changes accordingly. Interestingly, when the target λ_emi_ value changes from UV to near-infrared (NIR), the Gen-DL model was found use the molecular backbone structures associated with carbazole (λ_emi_ = ∼350 nm), coumarin (λ_emi_ = ∼450 nm), BODIPY (λ_emi_ = ∼550 nm), squaraine (λ_emi_ = ∼650 nm), and aza-BODIPY (λ_emi_ = ∼750 nm). These molecular backbone structures have been effectively used to develop fluorophores having a given range of λ_emi_. Therefore, the Gen-DL model is shown to find the structure–property relationship (SPR) between the molecular structure and λ_emi_ in DB_Gen-DL_, and using the SPR, the Gen-DL model can effectively generate molecules with target λ_emi_ values.

#### II-C-2 Absorption and Emission Bandwidths

Absorption and emission bandwidths are crucial to the design of chromophores and fluorophores in various research areas such as OLEDs, organic photovoltaics (OPVs), organic photodiodes (OPDs), and bioimaging. Narrow emission bandwidths are desired for fluorophores used in OLEDs, OPDs, and bioimaging, whereas wide absorption bandwidths are preferred for light-harvesting dyes in OPVs.^35−40^ Although some design principles have been used empirically,^41^ controlling the bandwidth of organic molecules remains extremely challenging. As shown in Figure 3c, the Gen-DL model can generate molecules with tuned emission bandwidths, allowing a wide range of bandwidths from narrow (1000 cm^–1^) to broad (4000 cm^–1^) to be readily achieved. In fact, the bandwidth regions of approximately 1,000 and 4,000 cm^–1^ with relatively few data points posed additional challenges for training the Gen-DL model. However, the Gen-DL model was found to generalize the design principles to be able to generate molecules in these bandwidth regions.

#### II-C-3 PLQY and PL Lifetime

PLQY (Φ) and PL lifetime (τ) are molecular properties that are significantly influenced by complicated intra- and intermolecular interactions in solutions and host matrices, and thus they are challenging to predict theoretically in practice. Accordingly, a reliable estimation of the PLQY and PL lifetime of organic molecules is crucial for practical application in OLEDs, OPVs, and OPDs. Our Pred-DL model has proven to be a reliable tool for predicting the PLQY and PL lifetime of organic molecules, and it surpassed the limitations of conventional theoretical calculations.^8,9^ Additionally, as shown in Figure 3e and 3f, the Gen-DL model exhibits the ability to generate molecules with specified Φ and τ values to some extent. Note that the PLQY is measured relatively imprecisely by currently available experimental methods when compared with UV–visible absorption and emission spectra, and the PLQY database is relatively noisy.^42,43^ Therefore, the SPR between molecular structure and PLQY appears to be learned relatively inaccurately by the Gen-DL model.

### II-D Solvent Effects and Structure–Property Relationships Embedded in the Gen-DL Model

#### II-D-1 Solvent-Dependent Molecular Generation

**Figure 4:**
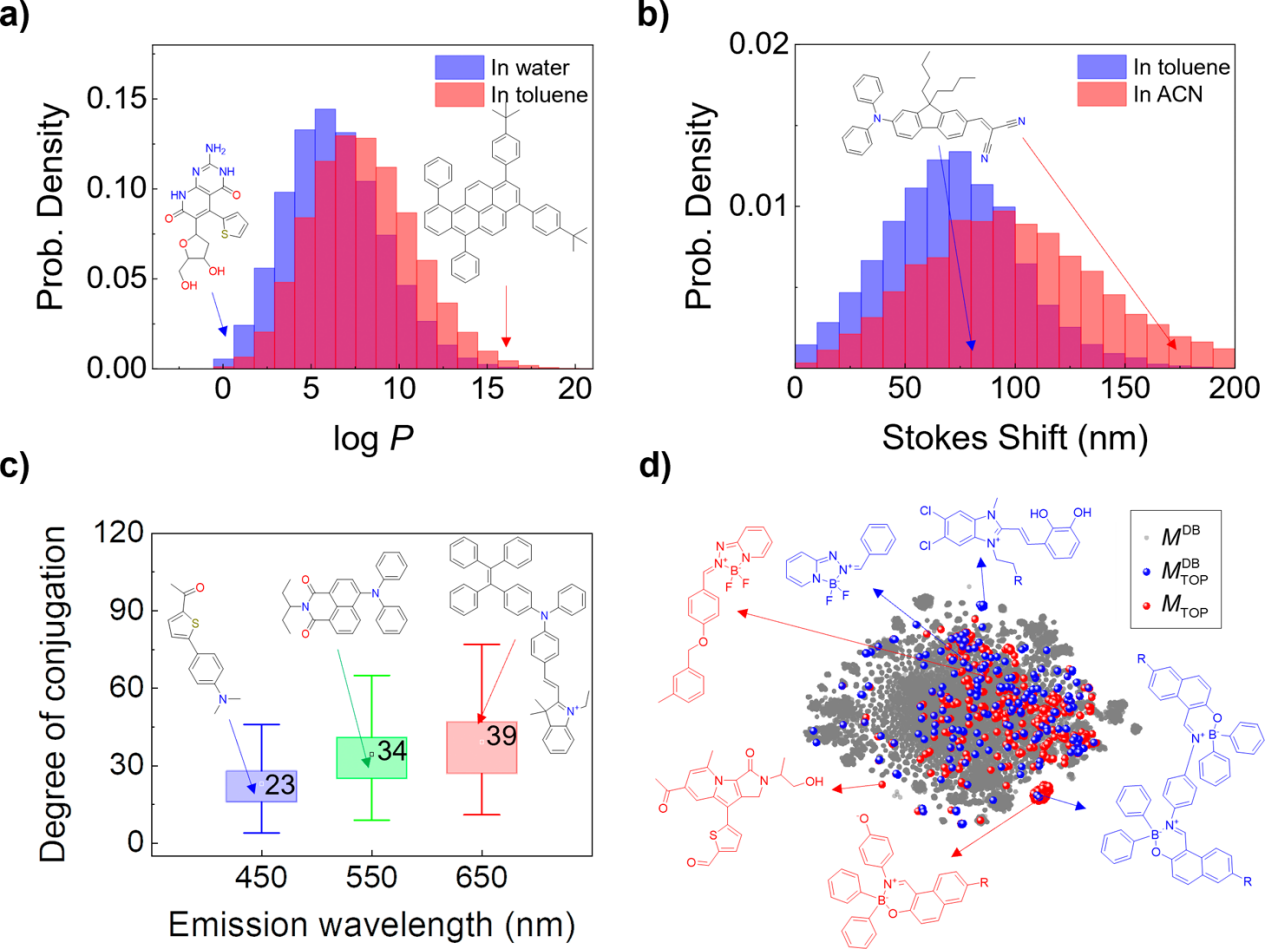
(a) Distribution of log *P* values of generated molecules in toluene and in water. (b) Distribution of the Stokes shift values of the generated molecules in toluene and in ACN. Inset: the molecule with an intramolecular charge transfer (ICT) upon electronic excitation, showing a Stokes shift of 80 nm in toluene and 187 nm in ACN. (c) Degree of conjugation of the generated molecules having different target λ_emi_ values. (d) *t*-distributed stochastic neighbor embedding (*t*-SNE) plot of molecules. **Gray**: Molecules in DB_Gen-DL_. **Blue**: Molecules with target optical properties (λ_abs_ = 452 nm, σ_abs_ = 3942 cm^–1^, log ε = 4.38, λ_emi_ = 550 nm, σ_emi_ = 2820 cm^–1^, Φ = 0.34, τ = 2.17 ns) in DB_Gen-DL_. **Red**: Generated molecules with the target properties.

The optical properties of molecules are significantly influenced by the surrounding solvent molecules in solutions. Our Gen-DL model is built to reflect solvent effects when generating molecules with target optical properties in a specified solvent. To examine how solvent effects are reflected by the Gen-DL model, we used the partition coefficients (*P*) of molecules generated in different solvents. The partition coefficient (*P*) is defined as the ratio of molecular concentrations in a mixture of water and *n*-octanol, and the log *P* value is used as a measure of lipophilicity (or hydrophobicity). In this study, the log *P* values of molecules were estimated using the method proposed by Crippen et al.,^44^ which was implemented in RDKit.^45^ A larger log *P* value indicates that the molecule is more hydrophobic. By properly reflecting solvent effects, our Gen-DL model is expected to generate more hydrophilic molecules in water than in organic solvents. In Figure 4a, the distribution of the log *P* values of the molecules generated in water shows a tendency toward smaller log *P* values compared with the molecules generated in toluene. This indicates that the Gen-DL model reflects solvent effects when generating molecules in different solvents.

The solvent effect on the properties of the generated molecules was further investigated by analysis of the Stokes shift, which is quantified by the difference between the λ_abs_ and λ_emi_ values. A large Stokes shift results from a large change in the charge distribution between the electronic ground and excited states, which often involves significant intramolecular charge transfer (ICT) in the molecule upon electronic excitation. Consequently, the molecules with the ICT upon electronic excitation exhibit a larger Stokes shift in highly polar solvents than in nonpolar solvents. Figure 4b shows the distributions of the Stokes shifts (λ_emi_ minus λ_abs_) for the same molecules in toluene (ε = 2.38, weakly polar) and acetonitrile (ACN, ε = 37.5, highly polar). The molecules generated in ACN exhibit relatively larger Stokes shifts than those in toluene, indicating that the solvent effects are appropriately reflected by the Gen-DL model.

#### II-D-2 Degree of Conjugation of Organic Molecules

One of the key structural features associated with λ_abs_ and λ_emi_ is the conjugation length, which refers to the lengths of the connected *sp* and *sp*^2^ hybridized atoms in a molecule. To quantify the conjugation length in a given molecule, we used the degree of conjugation (DOC), which is defined in this study as the number of bonds that connect the farthest atoms in the conjugated backbone of a molecule. To calculate the DOC of molecules, we developed a Python function using the RDKit library,^45^ which provides a powerful toolkit for cheminformatics (see the SI for details). To investigate the effect of the DOC on λ_emi_, we analyzed the DOC for ∼10,000 molecules generated at different λ_emi_ values in toluene. Figure 4c illustrates the trend in the DOC observed for the molecules at three different λ_emi_ values. As the λ_emi_ value increases, the DOC of the generated molecules tends to increase in toluene. Even though λ_emi_ is influenced by the solvent effect, it is important for the molecule to possess sufficient DOC to achieve target λ_emi_ values. This finding once again highlights the ability of our Gen-DL model to understand and leverage crucial design principles.

#### II-D-3 Exploring Structural Diversity

To investigate the structural diversity of molecules generated by the Gen-DL model, we performed *t*-SNE analysis using the Morgan fingerprints of the molecules, as illustrated in Figure 4d. The *t*-SNE analysis using the Morgan fingerprint provides a two-dimensional visualization of the structural diversity. In the *t*-SNE plot, structurally similar molecules are positioned closely, while structurally distinct molecules are placed farther apart. Figure 4d shows the *t*-SNE analysis of all molecules in DB_Gen-DL_ (in gray, *M*^DB^), the molecules with target optical properties in DB_Gen-DL_ (in blue, *M*_TOP_^DB^), and the generated molecules (in red, *M*_TOP_) with target optical properties (λ_abs_ = 452 nm, σ_abs_ = 3942 cm^–1^, log ε = 4.38, λ_emi_ = 550 nm, σ_emi_ = 2820 cm^–1^, Φ = 0.34, τ = 2.17 ns). Blue dots (*M*_TOP_^DB^) scattered over the distribution of gray dots (*M*^DB^) indicates that the molecules with the same target optical properties in DB_Gen-DL_ have diverse molecular structures. Interestingly, some of the red dots (*M*_TOP_) are located close to the blue dots (*M*_TOP_^DB^), showing that the Gen-DL model generates the molecules having molecular structures similar to those in DB_Gen-DL_. More interestingly, the red dots are found quite away from the blue dots in the *t*-SNE plot. This indicates that the Gen-DL model can generate molecules having different backbone structures with the same target optical properties. The *t*-SNE analysis shows that our Gen-DL model can generate molecules that are not only structurally similar to molecules in DB_Gen-DL_, but also structurally different from those in DB_Gen-DL_. This demonstrates that our Gen-DL model has great potential to generate molecule with new backbone structures that are not included in the training data set (DB_Gen-DL_).

### II-E Validating Practical Applications for Designing Novel and Useful Molecules

One of the ultimate goals of chemistry and materials science is to develop new molecules with desired properties for specific purposes in many research areas. Our Gen-DL model provides an efficient way to achieve this goal by generating molecules that satisfy specific properties in a given solvent. Rafael and co-workers previously demonstrated molecular discovery through the virtual screening and synthesis of new molecular structures.^46^ However, examining the performance of our Gen-DL model via direct synthesis of generated molecules is time-consuming. Therefore, in this study, we used a test data set (DB_Test_), which is mutually exclusive with the training data set (DB_Gen-DL_), to determine whether the molecules generated by Gen-DL model exhibit the target optical properties. In other words, the molecules generated by Gen-DL model were searched in DB_Test_ and the optical properties of the molecules found in DB_Test_ were compared with the target optical properties. This approach allowed us to examine the performance of the Gen-DL model without the need to synthesize molecules directly and measure their optical properties. This validation step can support the efficiency and applicability of our Gen-DL model in guiding the discovery of newly designed molecules.

**Figure 5:**
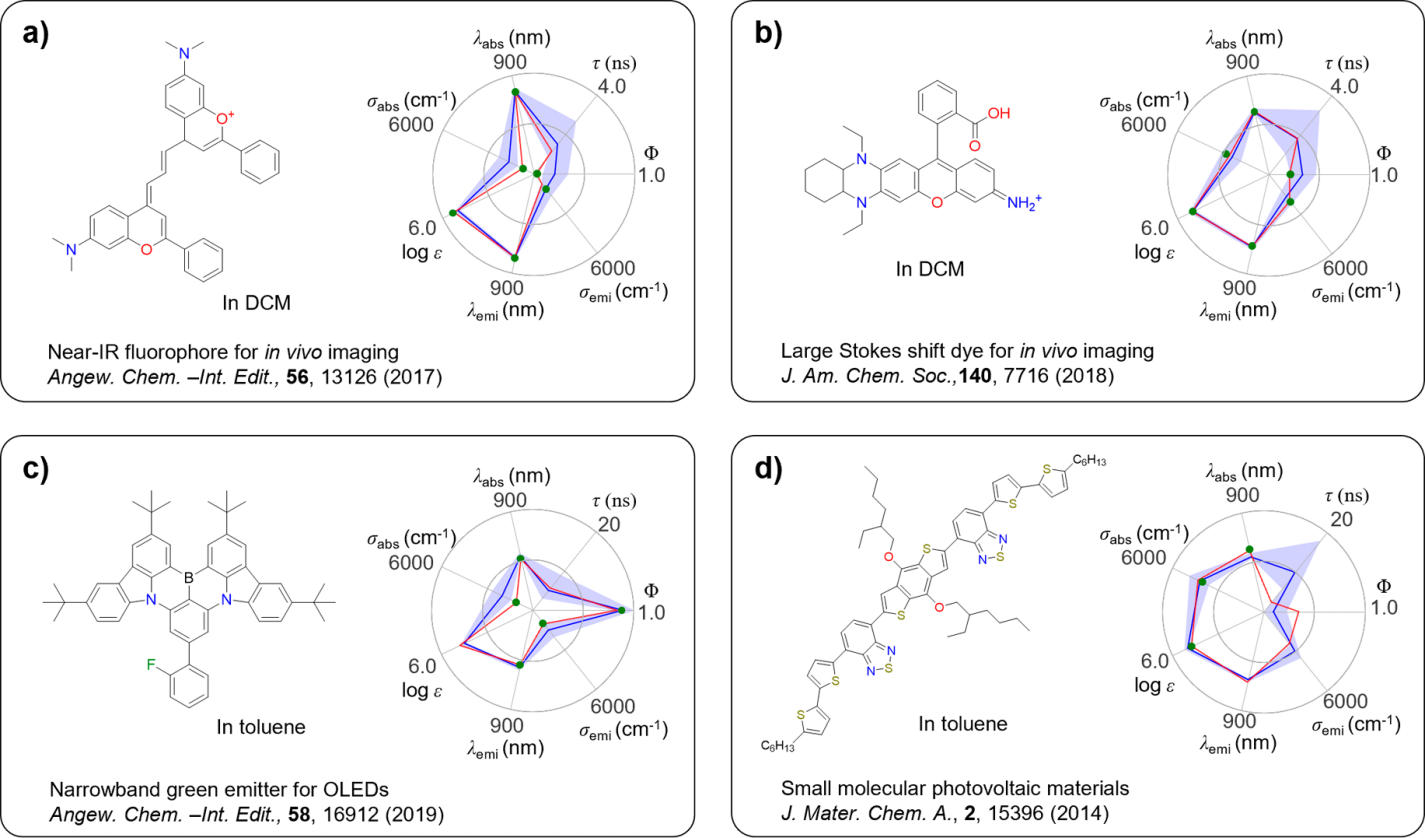
Examples of molecules generated by the Gen-DL model and their practical use. Experimental values (green dots), predicted values (red line), and target values (blue line) are compared in the radar plot. The range of optical properties considered to meet the target optical properties is indicated in shaded blue based on the RMSE of the Pred-DL model.

To directly examine the performance of our Gen-DL model, the molecules generated by the Gen-DL model were identified in DB_Test_ and their experimental properties were compared with the target properties, as shown in Figure 5.

Figure 5 shows several intriguing molecules that were generated by our Gen-DL model and were also found in DB_Test_. As a first example, given suitable target properties (e.g., λ_emi_ = 760 nm and log ε = 5), NIR fluorophores can be generated by the Gen-DL model. In fact, as shown in Figure 5a, the Gen-DL model generated a NIR imaging dye (λ_emi_ = 766 nm) with a large extinction coefficient (log ε = 5.34) that was developed by Sletten and co-workers for applications in high-resolution fluorescence microscopy.^32^ As a second example, imaging dyes with large Stokes shifts and large extinction coefficients are useful for *in vivo* imaging. For this purpose, the target properties (e.g., λ_abs_ = 570 nm, λ_emi_ = 660 nm, Stokes shift = 90 nm, log ε = 5) can be given. Our Gen-DL model was found to generate a fluorophore in Figure 5b which was originally developed by Ren and co-workers for fluorescence microscopy and *in vivo* imaging.^31^ Ren and co-workers showed that the fluorophore in Figure 5b exhibited deep tissue penetration, small autofluorescence interference, and large absorption due to the optimal optical properties (λ_abs_ = 571 nm and λ_emi_ = 651 nm) for biological tissues, the large Stokes shift (80 nm), and the large extinction coefficient (log ε = 5). As a third example, narrowband emitters in OLEDs can be generated by the Gen-DL model with the target properties (e.g., λ_emi_ = 520 nm, σ_emi_ = 1500 cm^–1^, Φ = 0.9). The emitter in Figure 5c, generated by our Gen-DL model, was originally developed by Zhang and co-workers.^30^ They showed that the emitter exhibited green emission (λ_emi_ = 500 nm) with a narrow bandwidth (σ_emi_ = 25 at 500 nm) and a high PLQY (Φ = 0.887). As the last example, organic photovoltaic materials require broad absorption spectra with a significantly large extinction coefficients to achieve high power conversion efficiency. To reflect these characteristics, the target properties (e.g., λ_abs_ = 500 nm, σ_abs_ = 4200 cm^–1^, and log ε = 5) were used as input for the Gen-DL model. As a result, the Gen-DL model generated a molecule in Figure 5d, originally developed by Liang and co-workers for small molecular photovoltaic applications.^33^ They demonstrated that this molecule has a wide absorption spectrum in the visible range (λ_abs_ = 569 nm and σ_abs_ = 4031 cm^–1^ = ∼120 nm at 570 nm), along with a high extinction coefficient (log ε = 4.74). In short, our Gen-DL model is shown to have great potential in generating novel and practically useful molecules that are not included in the training data set (DB_Gen-DL_).

## III Concluding Remarks

In this study, we successfully developed a generative DL model that was trained on a large experimental database (DB_exp_) including 71,424 molecule/solvent pairs and was able to design molecules with target optical properties in various solvents. Our Gen-DL model can generate molecules with specific optical properties, such as specific absorption and emission peak positions, bandwidths, extinction coefficients, PL lifetimes, and PLQY. Notably, the Gen-DL model was found to generate target molecules even in the ranges of the optical properties having insufficient training data. Furthermore, our Gen-DL model effectively reflected the solvent effects in generating molecules with target optical properties, which was verified by investigating log *P* values and Stokes shifts of generated molecules in different solvents. Additionally, using the DOC descriptor, we have demonstrated that our Gen-DL model understands and exploits the essential design principles of conjugation effects on absorption and emission wavelengths. The *t*-SNE analysis showed that the Gen-DL model can generate molecules that are not only structurally similar to molecules in DB_Gen-DL_, but also structurally different from those in DB_Gen-DL_. Lastly, we demonstrated the performance of our Gen-DL model by identifying the molecules generated by the Gen-DL model in the test data set (DB_Test_) and comparing their experimental properties with the target properties, which confirms that our Gen-DL model has great potential to generate new and practically useful molecules that are not included in the training data set (DB_Gen-DL_). Our Gen-DL model is a promising tool for discovering and designing novel molecules that have tailored properties, and it may pave the way for more efficient development of target materials in various research areas, such as OLEDs, OPVs, OPDs, bioimaging fluorophores, and so on. Our Gen-DL model is currently available as a web-based application (http://deep4chem.korea.ac.kr/DeepMoleculeGen).

When it is combined with the previously reported Pred-DL model (DLOS),^7^ the Gen-DL model can be effectively utilized to develop optimal molecules with specified properties in many research areas. The Pred-DL model can be used for efficient virtual screening of large numbers of predesigned molecules to select molecules with desired properties, whereas the Gen-DL model can directly generate molecules having target properties. More importantly, the Gen-DL model may offer the potential to discover new molecular backbones that have not been designed so far. Both Pred-DL and Gen-DL models can be used to build a library of molecules with target properties by screening predesigned molecules based on molecular property predictions and generating molecules with target properties. Synthesizable molecules can be selected from the library and synthesized directly. Finally, after confirming their properties by experiments, they can be used for practical applications in many research areas.

## Data Availability

Our Gen-DL model is publicly available as a web-based application (http://deep4chem.korea.ac.kr/DeepMoleculeGen). The codes and data for implementing our Gen-DL model are available at https://github.com/spark8ku/DeepMoleculeGen. The experimental database can be obtained from https://www.nature.com/articles/s41597-020-00634-8 or the corresponding author upon request for academic purposes only.

## Supporting Information Available

Materials and methods, Figures S1–S8, and Tables S1 and S2 (PDF)
Figures S9–S113, distributions of seven optical properties of generated molecules of every target property (ZIP)
Transparent Peer Review report available (PDF)
