# Supplementary material for: Generative Deep Learning-Based Efficient Design of Organic Molecules with Tailored Properties
Source: ACS Cent Sci. 2024 Aug 30;11(2):219–27. doi: 10.1021/acscentsci.4c00656 (PMC11869130; doi:10.1021/acscentsci.4c00656)
Supplement: Supplementary file 1 — oc4c00656_si_001.pdf [file oc4c00656_si_001.pdf]

Supporting Information

Generative deep learning-based efficient design of organic molecules with tailored properties

Minhi Han,† Joonyoung F. Joung,†‡ Minseok Jeong, Dong Hoon Choi, and Sungnam Park*

Department of Chemistry and Research Institute for Natural Science, Korea University, Seoul, 02841, Korea

*E-mail: spark8@korea.ac.kr (S.P.)

†These authors contributed equally to this work.

‡ Present address: Department of Chemical Engineering, Massachusetts Institute of Technology, Cambridge, Massachusetts 02139, United States

Contents

1. Figure S1. Datasets of the optical properties of organic molecules. ………………………………… S3

2. Figure S2. Results of training the Pred-DL model. ……………………………………………….. S4

3. Table S1. Performance of the Pred-DL model. ………..……………………………………………... S5

4. Figure S3. Correlation maps of seven optical properties in $DB_{Gen\text{-}DL}$. ……..…………………………… S6

5. Table S2. Target combinations of seven optical properties in different solvents. ……..………………..... S7

6. Molecular representation ……………………………………………………………………… S8

7. Figure S4. Schematic illustration of the adjacency and feature matrices for phenol. ………………….. S9

8. Gen-DL model ……………………………………………………………….……………….... S10

9. Figure S5. Schematic illustration of the Gen-DL model. …………………………………………… S11

10. Figure S6. Architecture of the Gen-DL model. …………..………………..…………………… S12

11. Actions for generating subsequent molecules in the Gen-DL model……………………………..… S13

12. Probability matrix of the next possible actions in the Gen-DL model …..…………….………….. S14

13. Figure S7. Illustrations of the probability of the next possible actions. …..……………………….. S15

14. Loss function of the Gen-DL model ..……………..……………………………………………… S16

15. Metrics for the performance of the Gen-DL model ……………………………………………… S17

16. Figure S8. Plots of average conditional generative scores ………………………………………… S18

17. Descriptor for the degree of conjugation of molecules……..…………..………….……………… S20

18. Algorithm S1. Calculation of the degree of conjugation of molecules ………………….………… S20

..

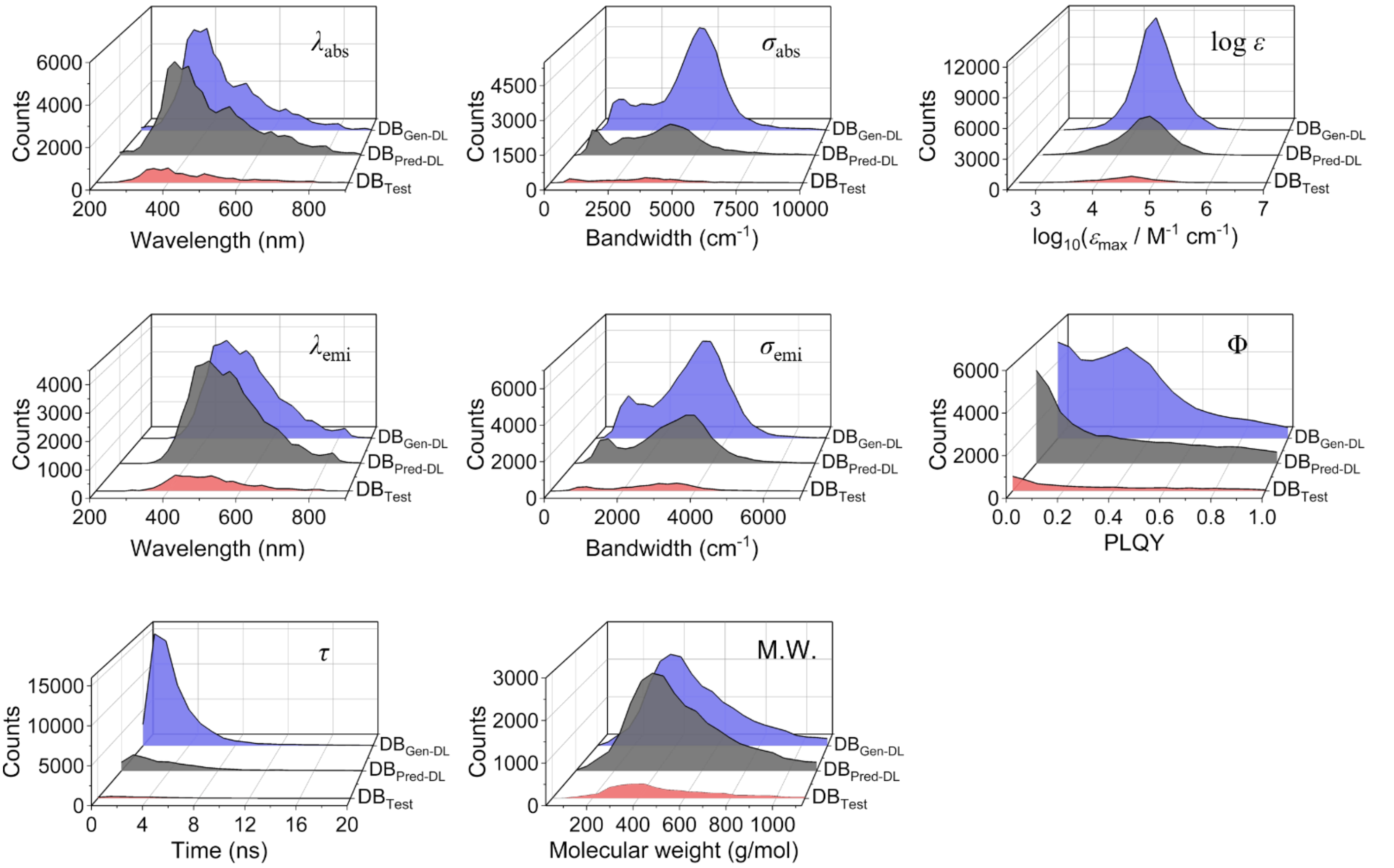

Figure S1. Datasets of the optical properties of organic molecules. Histograms of first absorption peak position (λ_{abs}), bandwidth (σ_{abs}) in the full width at half maximum (FWHM), extinction coefficient in logarithm (log ε), emission peak position (λ_{emi}), bandwidth (σ_{emi}) in the FWHM, PL quantum yield (PLQY, Φ), PL lifetime (τ), and molecular weights (M.W.). **Black**: Training dataset for Pred-DL model ($DB_{Pred\text{-}DL}$). **Blue**: Training dataset for Gen-DL model ($DB_{Gen\text{-}DL}$). **Red**: Test dataset for Gen-DL model (DB_{Test}).

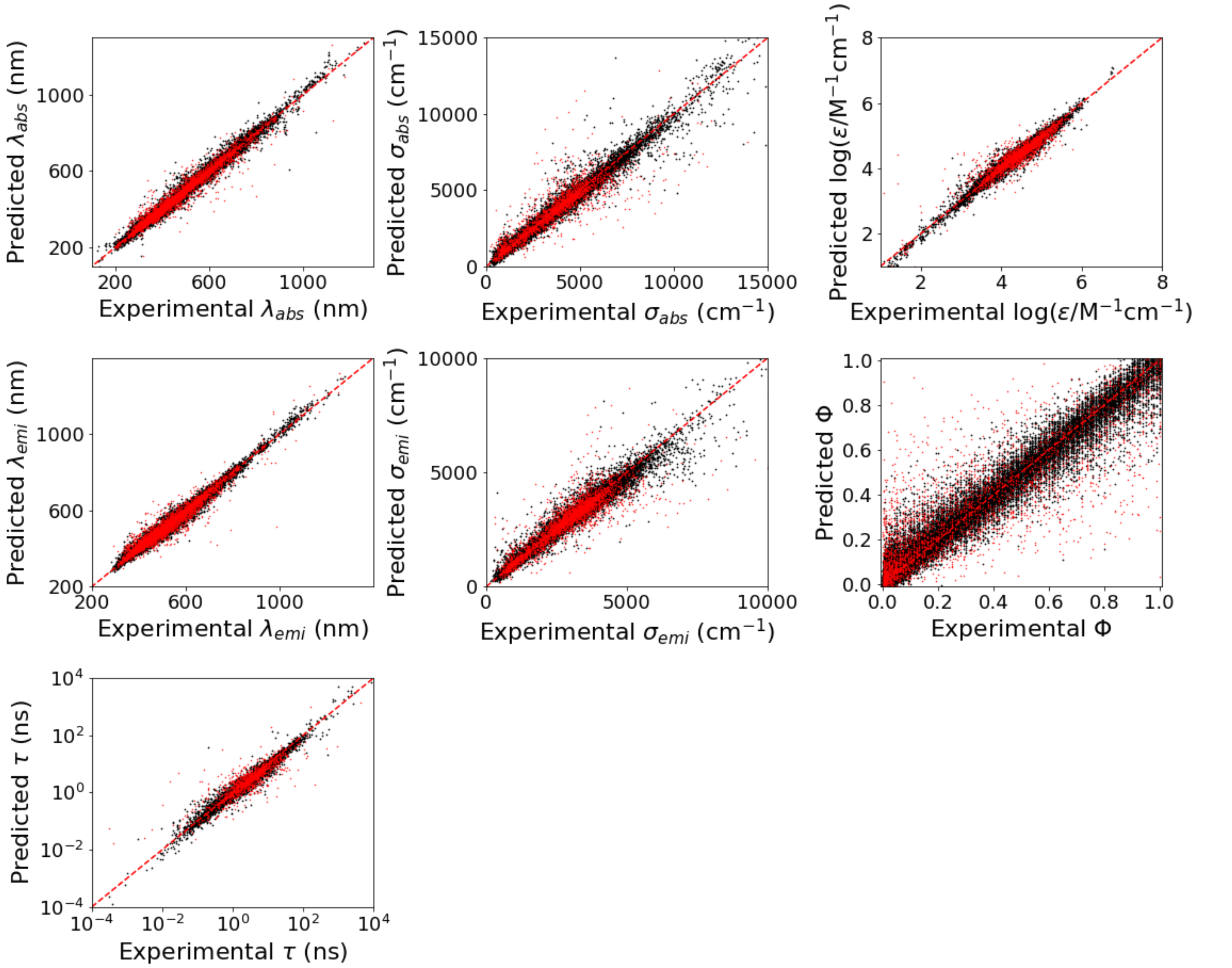

Figure S2. Results of training the Pred-DL model for the first absorption peak position (λ_{abs}), and absorption bandwidth in the FWHM (σ_{abs}), extinction coefficient (ε), emission peak position (λ_{emi}), emission bandwidth in the FWHM (σ_{emi}), PL quantum yield (PLQY, Φ), and PL lifetime (τ). Training and test datasets are in black and red dots, respectively.

Table S1. Performance of the Pred-DL model

	Training dataset		Test dataset	
Property	R^2	RMSE	R^2	RMSE
λ_{abs}	0.990	11.9 nm	0.922	26.6 nm
σ_{abs}	0.968	287.4 cm^{-1}	0.879	630 cm^{-1}
log ε	0.975	0.080	0.872	0.164
λ_{emi}	0.986	11.8 nm	0.946	18.3 nm
σ_{emi}	0.958	220.5 cm^{-1}	0.856	495 cm^{-1}
Φ	0.960	0.052	0.846	0.127
log τ	0.963	0.099	0.876	0.246

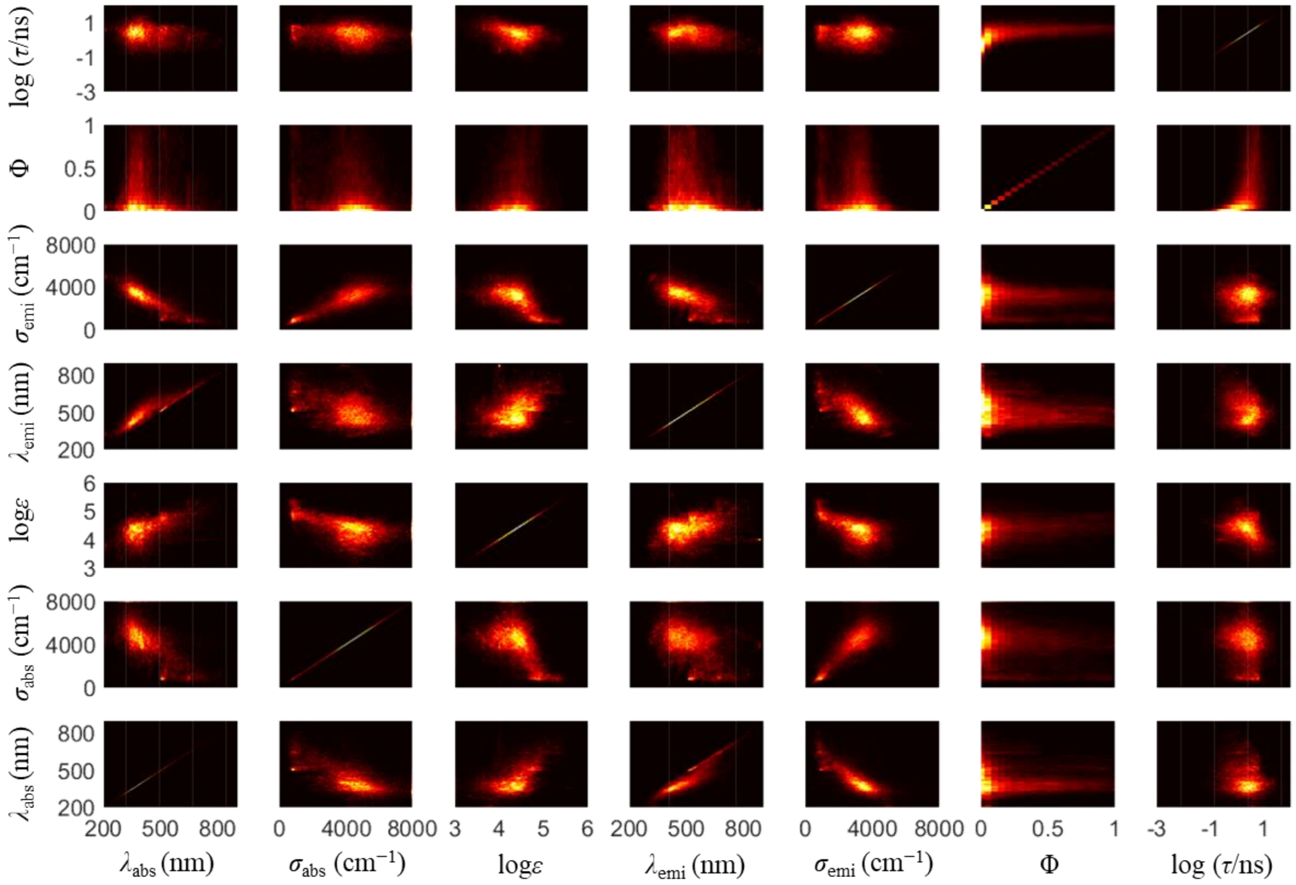

Figure S3. Correlation maps of seven optical properties in $DB_{Gen\text{-}DL}$.

Table S2. Target combinations of seven optical properties in different solvents.

	λ_{abs} (nm)	σ_{abs} (cm^{-1})	log ε	λ_{emi} (nm)	σ_{emi} (cm^{-1})	Φ	τ (ns)
λ_{abs}	350	4819	4.26	452	3576	0.34	2.26
	450	4217	4.34	540	2932	0.33	2.06
	550	2962	4.57	602	2081	0.35	1.90
	650	2155	4.80	685	1450	0.28	1.78
	750	1644	5.00	759	1138	0.21	1.50
σ_{abs}	612	1000	4.91	623	1100	0.36	2.17
	567	2000	4.71	597	1644	0.33	2.25
	491	3000	4.47	549	2414	0.34	2.21
	425	4000	4.29	514	3088	0.34	2.17
log ε	396	4662	4.00	494	3509	0.30	2.31
	436	4153	4.50	520	2897	0.34	1.93
	567	2405	5.00	609	1678	0.34	1.82
λ_{emi}	321	4781	4.19	350	3629	0.32	2.30
	368	4586	4.28	450	3448	0.37	2.28
	452	3942	4.38	550	2820	0.34	2.17
	552	3369	4.54	650	2248	0.27	1.66
	660	2508	4.83	750	1556	0.20	1.52
σ_{emi}	609	1697	4.84	631	1000	0.34	2.13
	509	3303	4.55	572	2000	0.33	2.12
	415	4444	4.31	508	3000	0.35	2.20
	374	4968	4.20	479	4000	0.30	1.85

Molecular representation

In the Gen-DL model, both molecule and solvent (or host) are represented as a graph structure with nodes and edges using adjacency and feature matrices. These matrices serve as inputs to a graph convolutional network (GCN). The adjacency matrix of a molecule has a size of $6 \times N \times N$ where N is the number of heavy atoms. Each element is one-hot encoded to indicate bond information (single, double, triple, and aromatic) as well as the second and third neighboring atoms. Additionally, the feature matrix of a molecule has a size of $N \times 3$, with elements representing the atom, formal charge, and the number of hydrogens bonded to the atom (Figure S4).

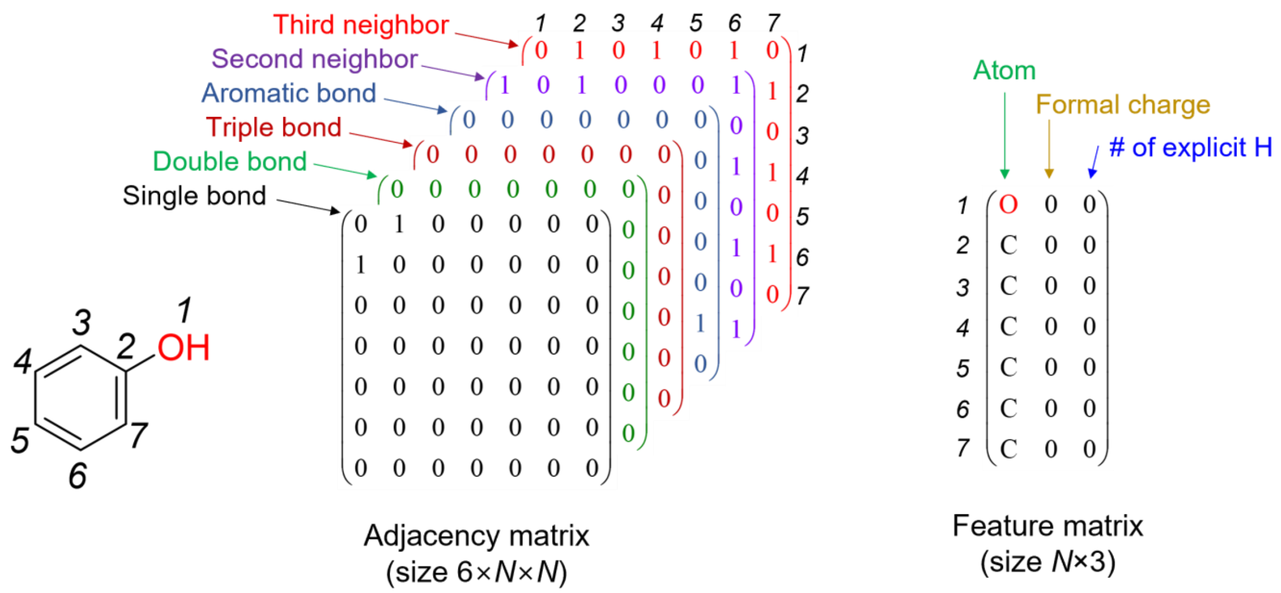

Figure S4. Schematic illustration of the adjacency and feature matrices for phenol.

Gen-DL model

As shown in Figure S5, the molecular generation sequences of the input molecule are stochastically generated from scratch by a stochastic depth-first search (SDFS) algorithm. In the molecular generation process, hydrogen atoms are implicitly included in each molecule. And the molecules in the sequence pass through the graph convolutional network (GCN) layers. The solvent also passes through the GCN layers and the multilayer perceptron (MLP). Then, the solvent vector and the vector of seven optical properties are broadcast and added to the GCN matrix of molecules. During this step, the solvent effect on the optical properties is embedded in the GCN matrix of molecules. Next, all the GCN matrices of molecules are concatenated and pass through the MLP. The chemical space matrix is then generated, followed by the molecular generation layer (MGL). In the MGL, the Gen-DL model learns all the possible molecular generation processes in the sequences by calculating the probabilities of (i) addition of an atom, represented by a vector (with atomic number, formal charge, the number of explicit hydrogen atoms), using a proper bond (single bond, double bond, triple bond, and aromatic bond), (ii) connecting two atoms to make a bond between them, and (iii) terminating the generation process. During the training process, the Gen-DL model learns how to generate the molecules with the target optical properties in a given solvent. The detailed architecture of the Gen-DL model is illustrated in Figure S6. The total number of parameters is 7,991,165. The inputs of the Gen-DL model are the adjacent and feature matrices of molecule and solvent and the target properties. Those inputs are passing through the deep neural network (DNN). The Gen-DL model predicts the probability matrices of the next possible actions to match the action vectors generated by the SDFS algorithm. The loss function is given by the cross entropy. We used batch size = 20, learning rate = 10^{-4}, decay rate = 0.015 for every 5 epochs, negative slope for the leaky ReLU = 0.1, L^2 regularization = 0.0005, the number of recurrent layers in the GRU = 3.

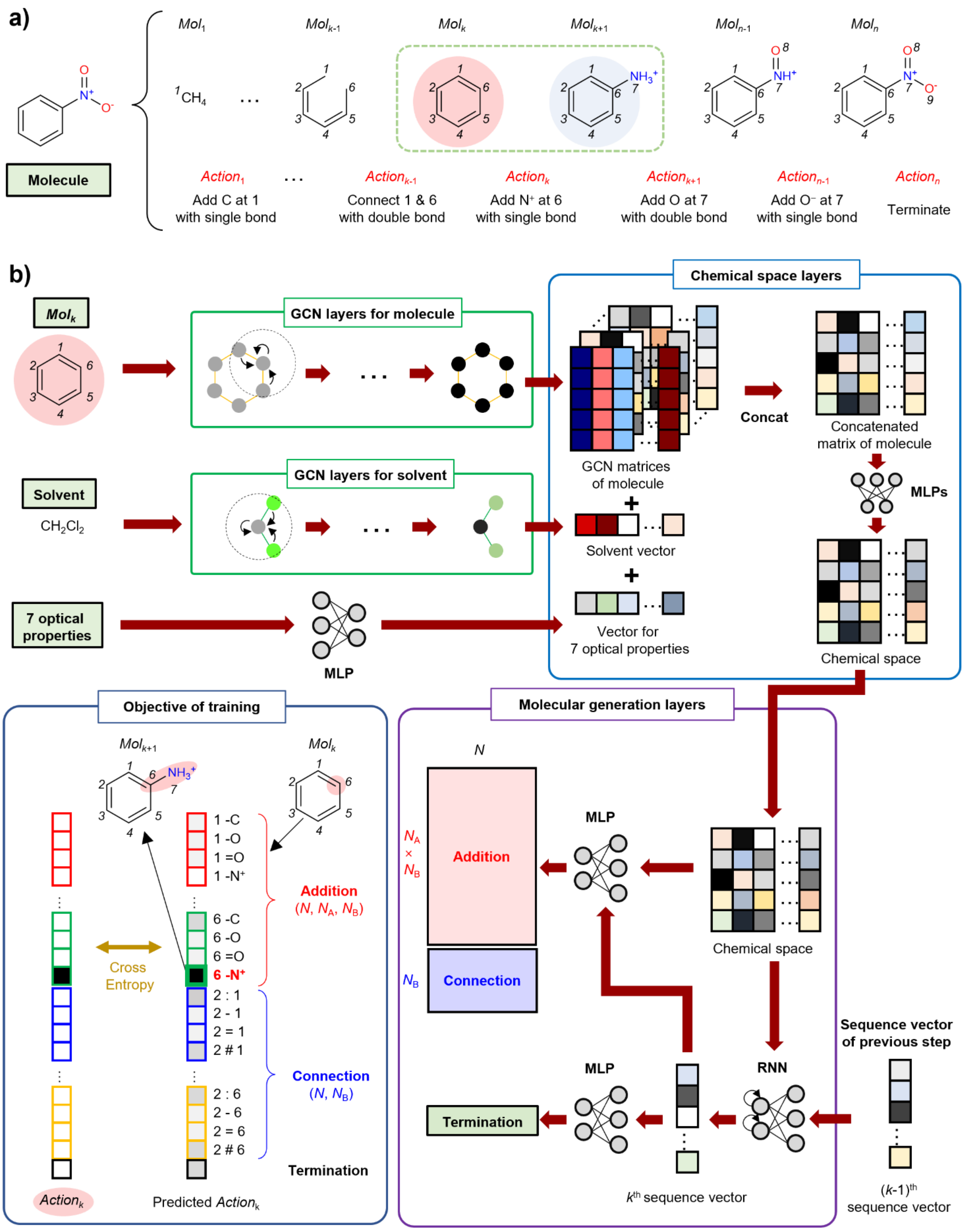

Figure S5. Training process of Gen-DL model. **(a)** Molecular generation sequence of an input molecule is generated by the SDFS algorithm. **(b)** Schematic of the Gen-DL model. The kth molecular structure (Mol_k), solvent (CH_2Cl_2), and seven optical properties are used as inputs. The Gen-DL model learns how to generate the $(k+1)^{th}$ molecular structure from the kth molecular structure in the sequence by calculating the probability of the next action (the addition of nitrogen cation at the 6 position). N: the number of atoms in the molecule; N_A: the number of atom types; N_B: the number of bond types.

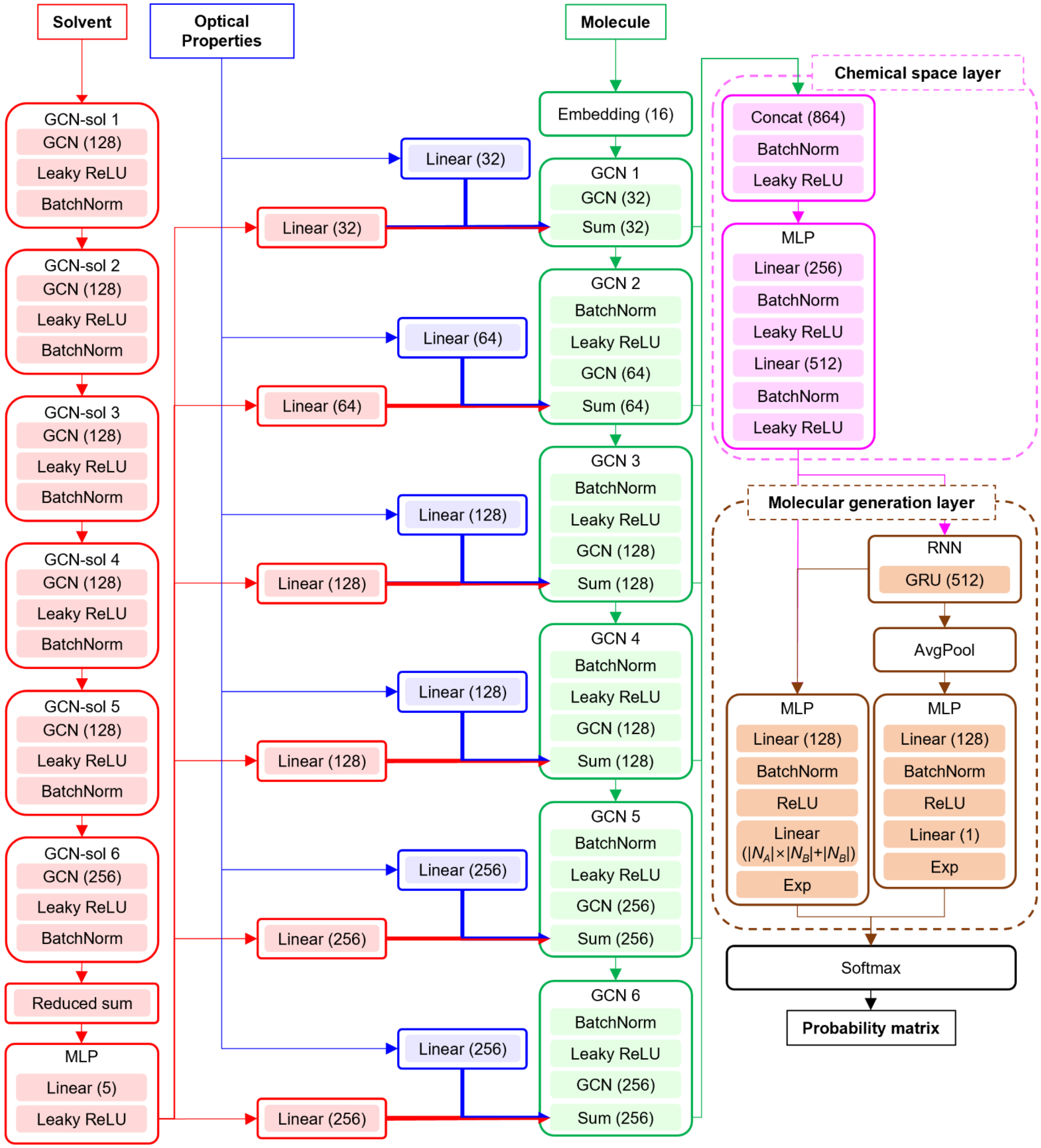

Figure S6. Architecture of the Gen-DL model. GCN, graph convolutional network; MLP, Multilayer perceptron; Sum, element-wise summation; Concat, concatenation; RNN, recurrent neural network; GRU, gated recurrent unit; AvgPool, average pooling.

Actions for generating subsequent molecules in the Gen-DL model

The molecular generation sequences of an input molecule are generated from scratch (a carbon atom) by the stochastic depth first search (SDFS) algorithm, each molecular generation sequence is composed of kth molecular structure and k^{th} action (Figure S5a). We define the kth action that determines whether to add an atom with a proper bond (single bond, double bond, triple and so on), connect two atoms to make a bond between them, or terminate the generation process. The kth action is represented by a vector and consists of the action types (addition, connection, and termination), atom type, bond type, position for adding the atom, and position for connecting the atoms. At the kth generation step (k^{th} molecular structure → $(k+1)^{th}$ molecular structure), (1) If an atom is added to the kth molecular structure to generate the $(k+1)^{th}$ molecular structure, the kth action is "addition" (which is specified by the atom type, the position for the addition, and the bond type). (2) If two atoms of the kth molecular structure are connected to generate the $(k+1)^{th}$ molecular structure, the kth action is "connection" (which is specified by the bond type and the two positions of the connecting atoms). (3) If the kth molecular structure is the terminal molecule in the molecular generation sequence, the kth action is termination. In the molecular generation sequences generated by the SDFS algorithm, the kth molecular structure is used as input to the Gen-DL model, the kth action is compared with the output of the Gen-DL model and used to train the Gen-DL model.

Probability matrix of the next possible actions in the Gen-DL model

As shown in Figure S7, the Gen-DL model calculates the probability tensors of the next possible actions (i.e., addition, connection, and termination) represented as $\hat{p}_{\text{addition}} \in \mathbb{R}^{N \times N_{\text{A}} \times N_{\text{B}}}$, $\hat{p}_{\text{connection}} \in \mathbb{R}^{N \times N_{\text{B}}}$, $\hat{p}_{\text{termination}} \in \mathbb{R}$, respectively. Here, N represents the number of the heavy atoms excluding hydrogen, N_{A} represents the number of the atom types, and N_{B} represents the number of the bond types. There are two ways for selecting the action from the probability tensors. On one hand, the action is selected by the largest element in the probability tensors. For example, (1) if the largest tensor element is selected from $\hat{p}_{\text{addition}}$, its index ($N_j$, $N_{\text{A}j}$, $N_{\text{B}j}$) determines the atomic position (N_j) to add an atom ($N_{\text{A}j}$) with a bond ($N_{\text{B}j}$). (2) If the largest tensor element is selected from $\hat{p}_{\text{connection}}$, its index ($N_j$, $N_{\text{B}j}$) determines the atomic position (N_j) for making a bond ($N_{\text{B}j}$). In fact, two indices of atoms are needed for the connection. However, based on the SDFS algorithm, it is determined which atom can be connected with the atom (N_j) to generate the subsequent molecular structure. (3) If the largest tensor element is selected from $\hat{p}_{\text{termination}}$, the molecular generation process is terminated, and the Gen-DL model outputs the terminal molecule with target properties. If the Gen-DL model selects the largest element of the probability tensor, it will not be able to generate structurally diverse molecules. On the other hand, if the elements of the probability tensor are selected by their own probabilities to select the next actions, the Gen-DL model can generate structurally diverse molecules.

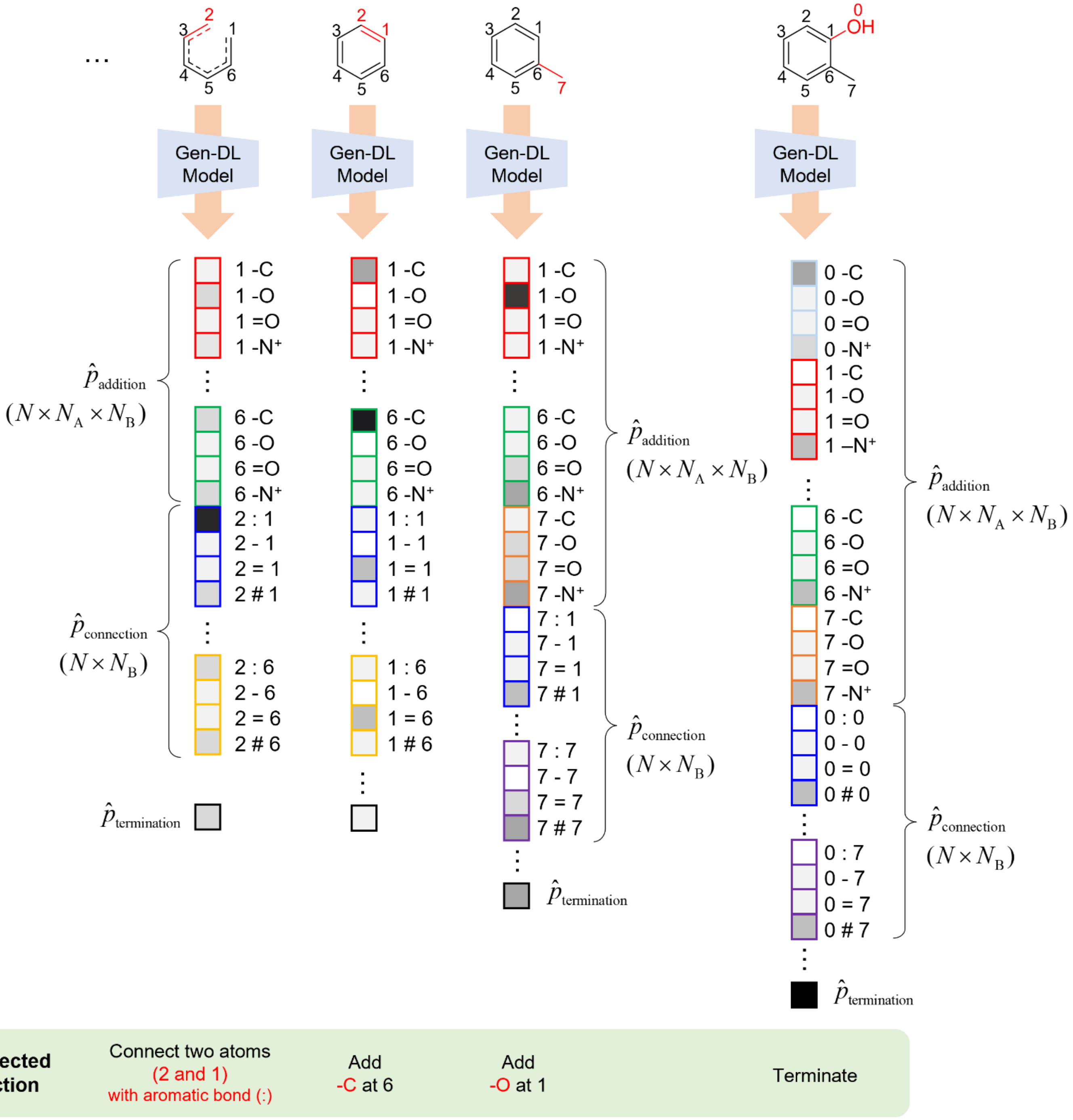

Figure S7. Probability tensors and selection of the next actions. At each step, the Gen-DL model predicts the probabilities of all possible next actions, and then the next action (black filled square) is probabilistically selected to generate the subsequent molecular structure. At the first step, the Gen-DL model selects (2:1) as the next action, which means that two atoms (1 & 2) are connected with an aromatic bond (:). At the second step, the Gen-DL model selects (6-C) as the next action, which means that a carbon atom with single bond (-C) is added to position 6. At the third step, (1-O) is selected so that an oxygen atom with single bond (-O) is added to position 1. At the last step, termination is selected and then the molecular generation process ends.

Loss function of the Gen-DL model

The Gen-DL model was trained with an experimental database, which includes molecular structures and their optical properties in various solvents. In the Gen-DL model, the probability of generating a molecule with target optical properties in a given solvent is expressed by

$$p\left(\text{Molecule} \mid \text{Solvent}, \mathbf{C}\right) = \prod_{k}^{N} p_k\left(Mol_k \mid Mol_{k-1}, \ldots, Mol_0, \text{Solvent}, \mathbf{C}\right)$$
$$\mathbf{C} = (\lambda_{\text{abs}}, \sigma_{\text{abs}}, \log\varepsilon, \lambda_{\text{emi}}, \sigma_{\text{emi}}, \Phi, \tau) \quad (1)$$

where p_i represents the probability of generating the kth molecular structure, conditioned on the $(k-1)^{\text{th}}$ molecular structure, solvent, and optical properties, and N is the number of the molecular generation steps for a given molecule. Here, $Mol_0, \ldots, Mol_N$ represent the molecular structures in the molecular generation sequence (Figure S5a), with Mol_0 being the initial atom (or backbone) and Mol_N being the final molecule. To simplify the generation process, the Gen-DL model uses a recurrent neural network (RNN) to generate molecules sequentially. The RNN maintains a hidden state, which can be thought of as a memory that captures the relevant approximation from the previous steps. The hidden state of the RNN at step k, denoted as h_k^{RNN}, is expressed by

$$h_k^{\text{RNN}} = \text{RNN}_\theta\left(h_{k-1}^{\text{RNN}}, Mol_{k-1}, \text{Solvent}, \mathbf{C}\right) \quad (2)$$

where RNN_θ is the RNN function with parameters θ, h_{k-1}^{RNN} is the hidden state from the previous step, Mol_{k-1} is the molecular structure from the previous step. By using the RNN function, the probability $p_k\left(Mol_k \mid Mol_{k-1}, \ldots, Mol_0, \text{Solvent}, \text{C}\right)$ can be approximated as

$$p_k\left(Mol_k \mid Mol_{k-1}, \ldots, Mol_0, \text{Solvent}, \text{C}\right) \cong p_k\left(Mol_k \mid Mol_{k-1}, h_k^{\text{RNN}}, \text{Solvent}, \text{C}\right) \quad (3)$$

This approximation allows the model to generate molecules without explicitly considering all possible ways of constructing the molecular structure step by step because the hidden state h_k^{RNN} captures the relevant information from the previous steps. To train the Gen-DL model, we define a loss function that quantifies the

difference between the model's predictions and the ground truth. The loss function is based on the negative log-likelihood of the generated molecular structures,

$$L = -\frac{1}{N}\sum_{k=1}^{N}\log\left[p_k(Mol_k \mid Mol_{k-1}, h_k^{\mathrm{RNN}}, \mathrm{Solvent}, \mathrm{C})\right] \tag{4}$$

Metrics for the performance of the Gen-DL model

The performance of the Gen-DL model was examined with metrics such as the validity (%M_{Val}), uniqueness (%M_{Uni}), and novelty (%M_{New}) which are defined by

$$\%M_{\mathrm{Val}} = \frac{\#\text{ of } M_{\mathrm{Val}}}{\#\text{ of } M_{\mathrm{Gen}}} \times 100\% \tag{5}$$

$$\%M_{\mathrm{Uni}} = \frac{\#\text{ of } M_{\mathrm{Uni}}}{\#\text{ of } M_{\mathrm{Gen}}} \times 100\% \tag{6}$$

$$\%M_{\mathrm{New}} = \frac{\#\text{ of } M_{\mathrm{New}}}{\#\text{ of } M_{\mathrm{Gen}}} \times 100\% \tag{7}$$

where M_{Val} represents structurally valid molecules, confirmed by the function implemented in the RDKit (Chem.MolFromSmiles). Next, M_{Uni} represents structurally unique molecules. Finally, M_{New} represents the molecules that are not present in the training dataset ($\mathrm{DB}_{\mathrm{Gen\text{-}DL}}$). The Pred-DL model was used to predict the optical properties of all generated molecules. If the optical properties of the generated molecule are close to the target properties within the root mean square error (RMSE) of the Pred-DL model (λ_{abs} ± 31.6 nm, λ_{emi} ± 26.7 nm, σ_{abs} ± 927 cm^{-1}, σ_{emi} ± 530 cm^{-1}, log ε ± 0.217, Φ ± 0.159, and log(τ/ns) ± 0.277 in Table S1), the optical properties are considered to satisfy the target properties. M_{TOP} is defined as the molecule whose seven optical properties simultaneously satisfy target optical properties in a given solvent.

Note that the performance of the Gen-DL model was found to depend on the epoch of training as shown Figure S8. As the epoch is increased, $\%M_{\mathrm{Val}}$ is gradually increased but $\%M_{\mathrm{New}}$ and $\%M_{\mathrm{Uni}}$ are decreased. To obtain reasonable performance in terms of the above four metrics, we used the Gen-DL model trained for 200 epochs in this study. After training for 200 epochs, the Gen-DL model was found to generate molecules

with $\%M_{\mathrm{Val}}$ = ~88.9 %, $\%M_{\mathrm{Uni}}$ = ~47.1 %, $\%M_{\mathrm{New}}$ = ~44.4 %, and $\%M_{\mathrm{TOP}}$ = ~4.4 %. After training for 680 epochs, $\%M_{\mathrm{Uni}}$ and $\%M_{\mathrm{New}}$ are still over 40 % and appears to be saturated.

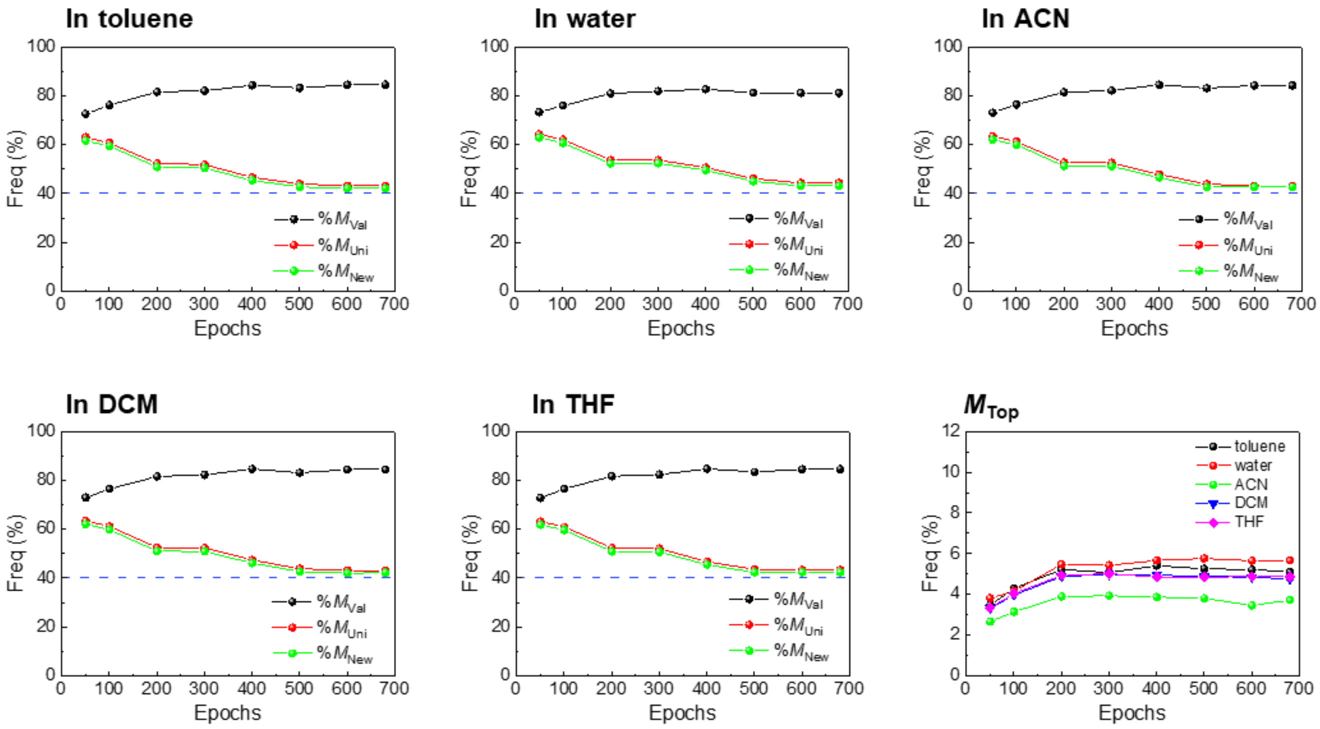

Figure S8. Performance metrics of the Gen-DL model depending on the epoch. 40% is indicated by blue dashed lines. After training for 200 epochs, M_{TOP} appears to be saturated.

Additionally, the structural diversity, D_{Gen}, of M_{TOP} is calculated by

$$D_{\mathrm{Gen}} = \frac{1}{(\#\text{ of } M_{\mathrm{TOP}})^2} \sum_{x \in M_{\mathrm{TOP}}} \sum_{y \in M_{\mathrm{TOP}}} \{1 - T_{\mathrm{S}}(x, y)\} \tag{8}$$

where T_{S} is the Tanimotto similarity. To examine the structural diversity of the generated molecules compared to those from $\mathrm{DB}_{\text{Gen-DL}}$, we used D_{DB}, defined as

$$D_{\mathrm{DB}} = \frac{1}{(\#\text{ of } M_{\mathrm{TOP}})(\#\text{ of } M_{\mathrm{TOP}}^{\mathrm{DB}})} \sum_{x \in M_{\mathrm{TOP}}} \sum_{y \in M_{\mathrm{TOP}}^{\mathrm{DB}}} \{1 - T_{\mathrm{S}}(x, y)\} \tag{9}$$

where $M_{\mathrm{TOP}}^{\mathrm{DB}}$ is the molecules satisfying seven target optical properties in $\mathrm{DB}_{\text{Gen-DL}}$. The closer D_{Gen} and D_{DB} to 1, the more diverse molecules were generated by the Gen-DL model. Finally, to evaluate the efficiency of

generated molecules satisfying target optical properties, we used the enrichment over random (*EOR*), defined by

$$EOR = \frac{\left(\#\ \text{of}\ M_{\text{TOP}}\right)/\left(\#\ \text{of}\ M_{\text{Uni}}\right)}{\left(\#\ \text{of}\ M_{\text{TOP}}^{\text{DB}}\right)/\left(\#\ \text{of}\ M^{\text{DB}}\right)} \quad (10)$$

Additional metrics (D_{Gen}, D_{DB}, and *EOR*) for the performance of the Gen-DL model are also shown in Figure S9~S113.

Descriptor for the degree of conjugation of molecules

In this study, the degree of conjugation (DOC) is defined as the number of bonds between the farthest atoms in the conjugated system with alternating *sp* and sp^2 hybridized atoms. To calculate the DOC of molecules, we developed an algorithm (See Algorithm S1).

Algorithm S1. Calculation of the degree of conjugation of molecules (pseudo code)

```
FUNCTION calculate_degree_of_conjugation(molecule):
    distance_matrix ← CALCULATE_DISTANCE_MATRIX(molecule)
    degree_of_conjugation ← EMPTY_LIST

    FUNCTION recursive_traverse(atom, visited):
        MARK_ATOM_AS_VISITED(atom, visited)
        FOR EACH neighbor OF atom:
            IF (neighbor IS sp OR sp2 HYBRIDIZED) AND (neighbor IS NOT IN VISITED):
                RECURSIVE_TRAVERSE(neighbor, visited)

    FOR EACH atom IN molecule:
        visited ← EMPTY SET
        RECURSIVE_TRAVERSE(atom, visited)
        IF visited IS NOT EMPTY:
            max_distance ← MAX(distance_matrix[visited, visited])
            APPEND max_distance TO degree_of_conjugation

    RETURN MAX(degree_of_conjugation, DEFAULT=0)
```
