# Supplementary figures and images for: Generative Deep Learning-Based Efficient Design of Organic Molecules with Tailored Properties (part 2 of 2)
Source: ACS Cent Sci. 2024 Aug 30;11(2):219–27. doi: 10.1021/acscentsci.4c00656 (PMC11869130; doi:10.1021/acscentsci.4c00656)

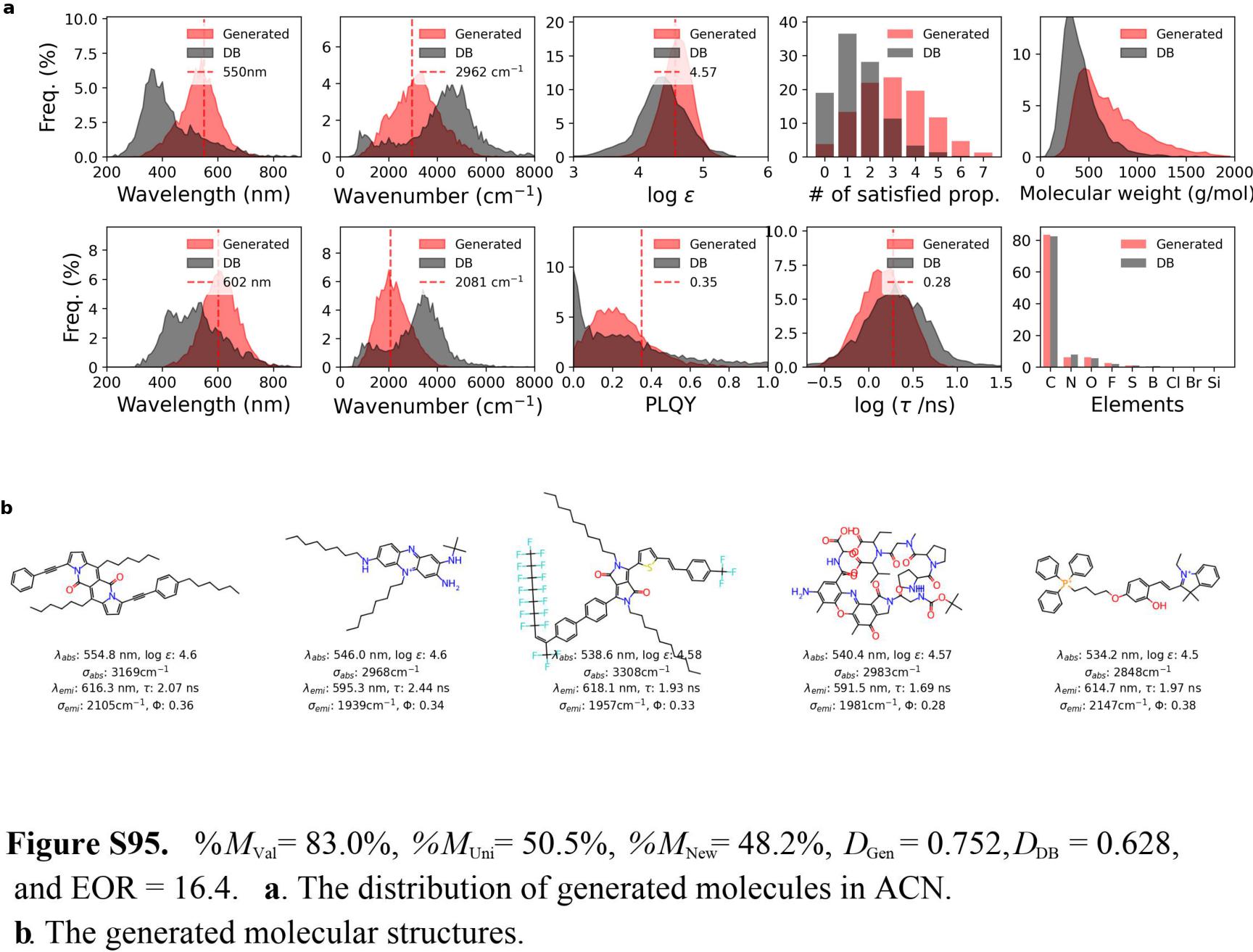

Supplement: Supplementary file 2 — oc4c00656_si_002.zip [file oc4c00656_si_002.zip › FigureS95.jpg]

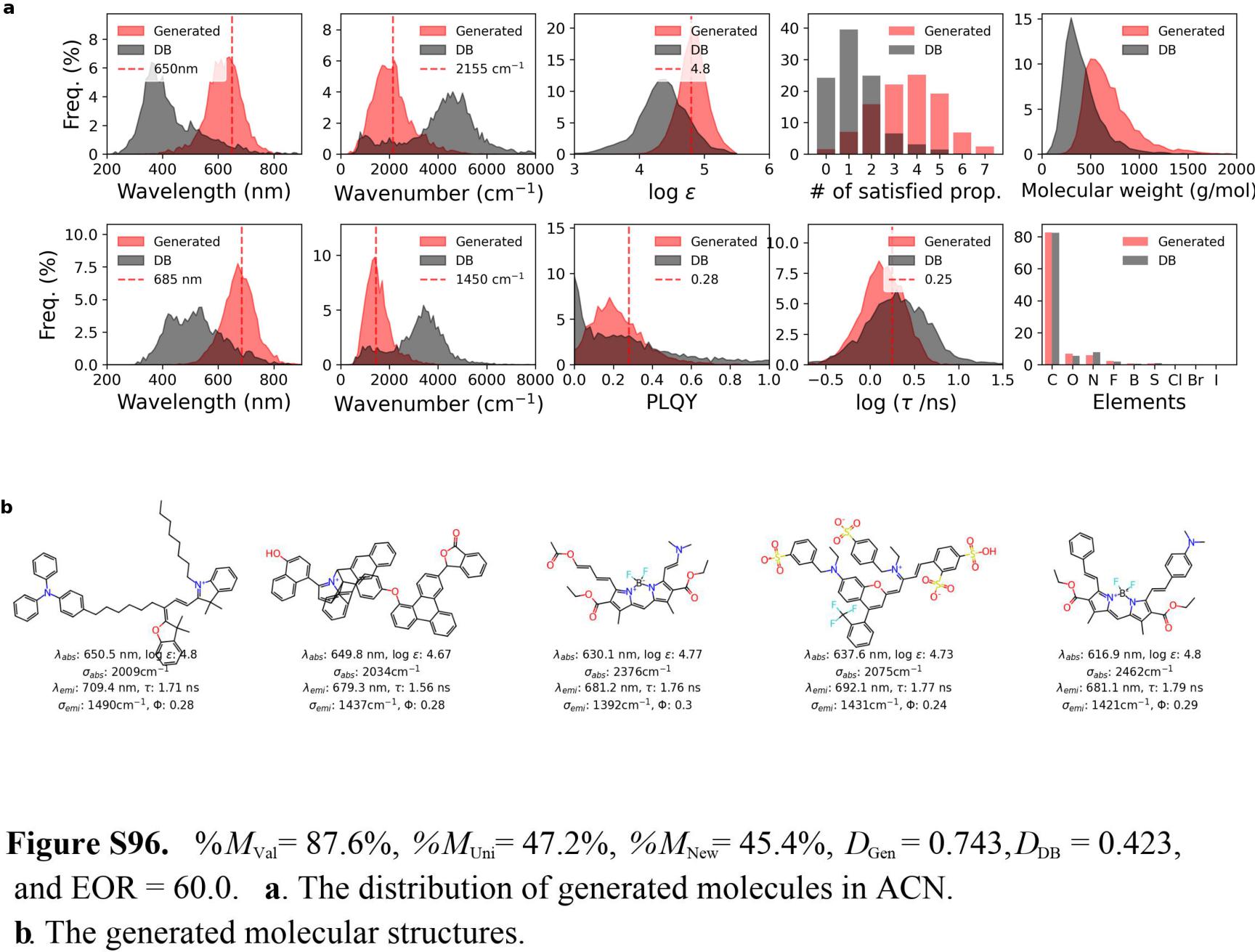

Supplement: Supplementary file 2 — oc4c00656_si_002.zip [file oc4c00656_si_002.zip › FigureS96.jpg]

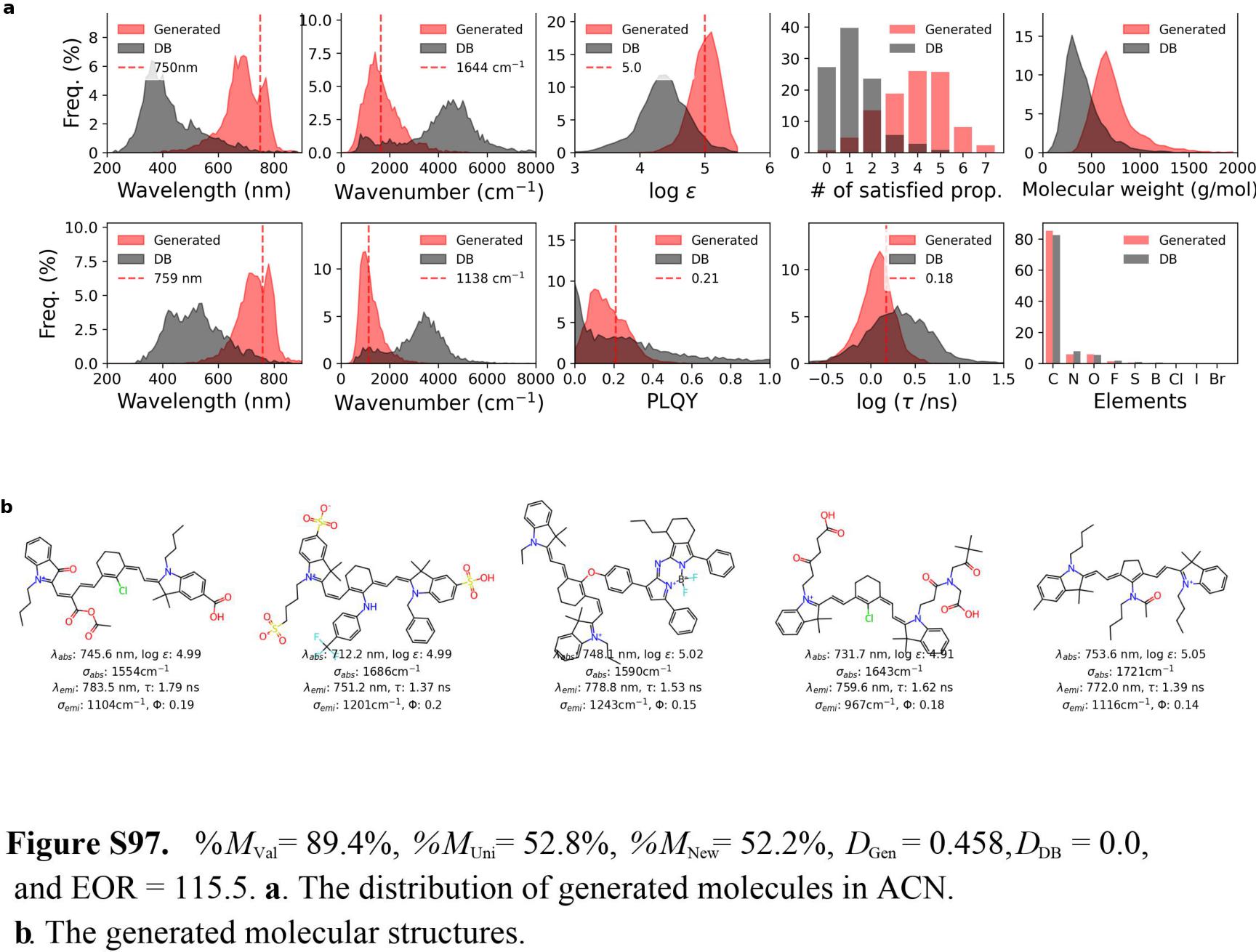

Supplement: Supplementary file 2 — oc4c00656_si_002.zip [file oc4c00656_si_002.zip › FigureS97.jpg]

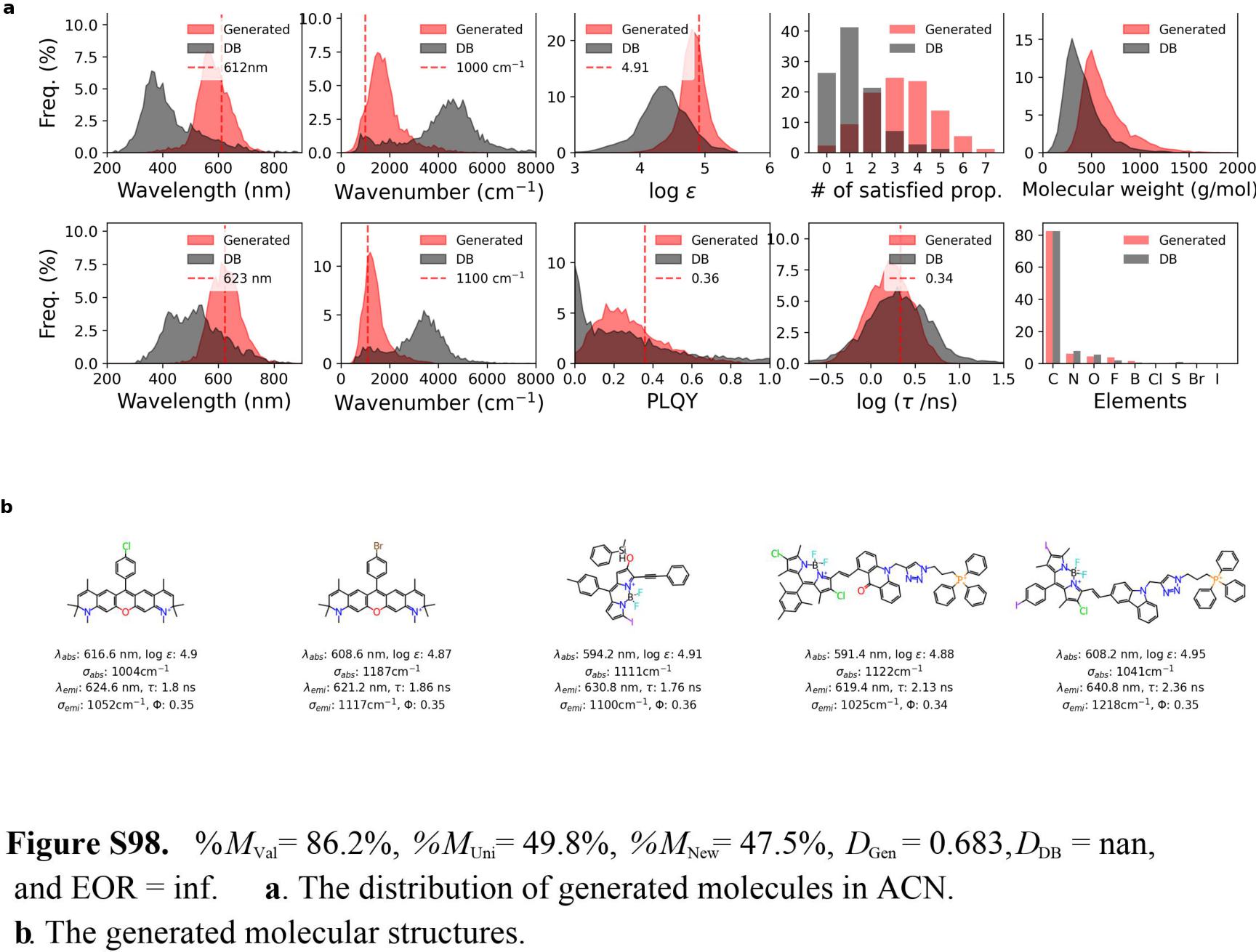

Supplement: Supplementary file 2 — oc4c00656_si_002.zip [file oc4c00656_si_002.zip › FigureS98.jpg]

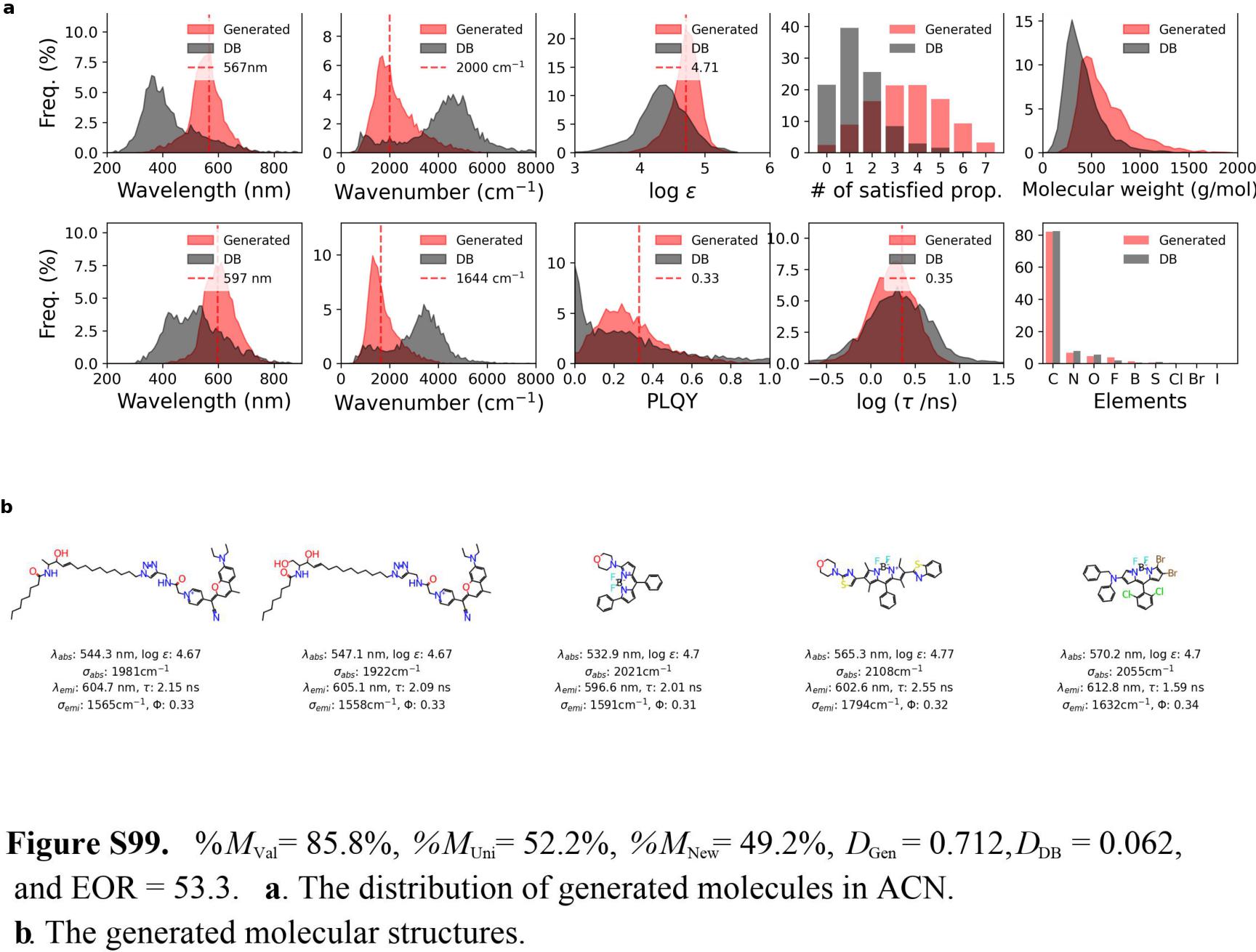

Supplement: Supplementary file 2 — oc4c00656_si_002.zip [file oc4c00656_si_002.zip › FigureS99.jpg]
