# Supplementary figures and images for: Generative Deep Learning-Based Efficient Design of Organic Molecules with Tailored Properties (part 1 of 2)
Source: ACS Cent Sci. 2024 Aug 30;11(2):219–27. doi: 10.1021/acscentsci.4c00656 (PMC11869130; doi:10.1021/acscentsci.4c00656)

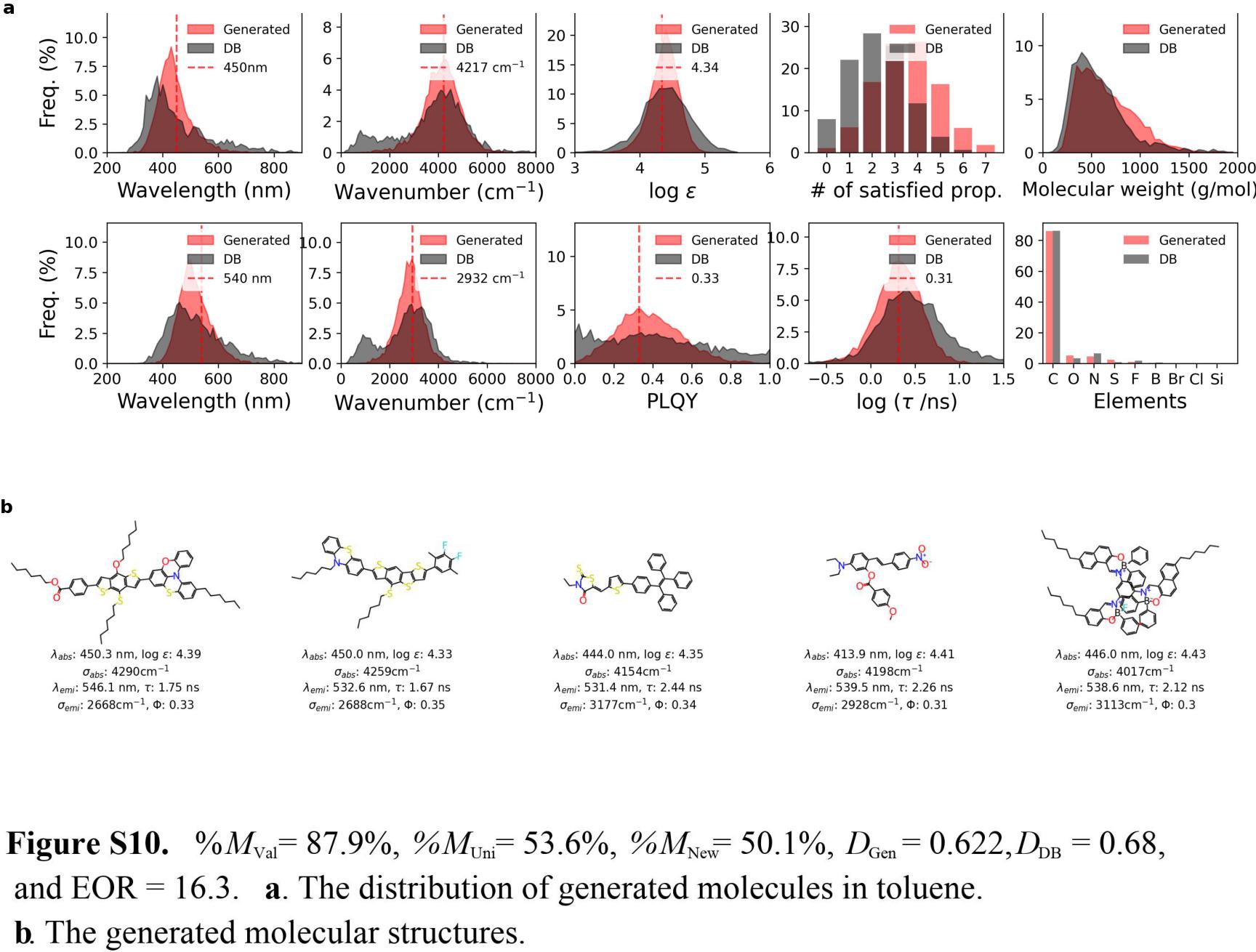

Supplement: Supplementary file 2 — oc4c00656_si_002.zip [file oc4c00656_si_002.zip › FigureS10.jpg]

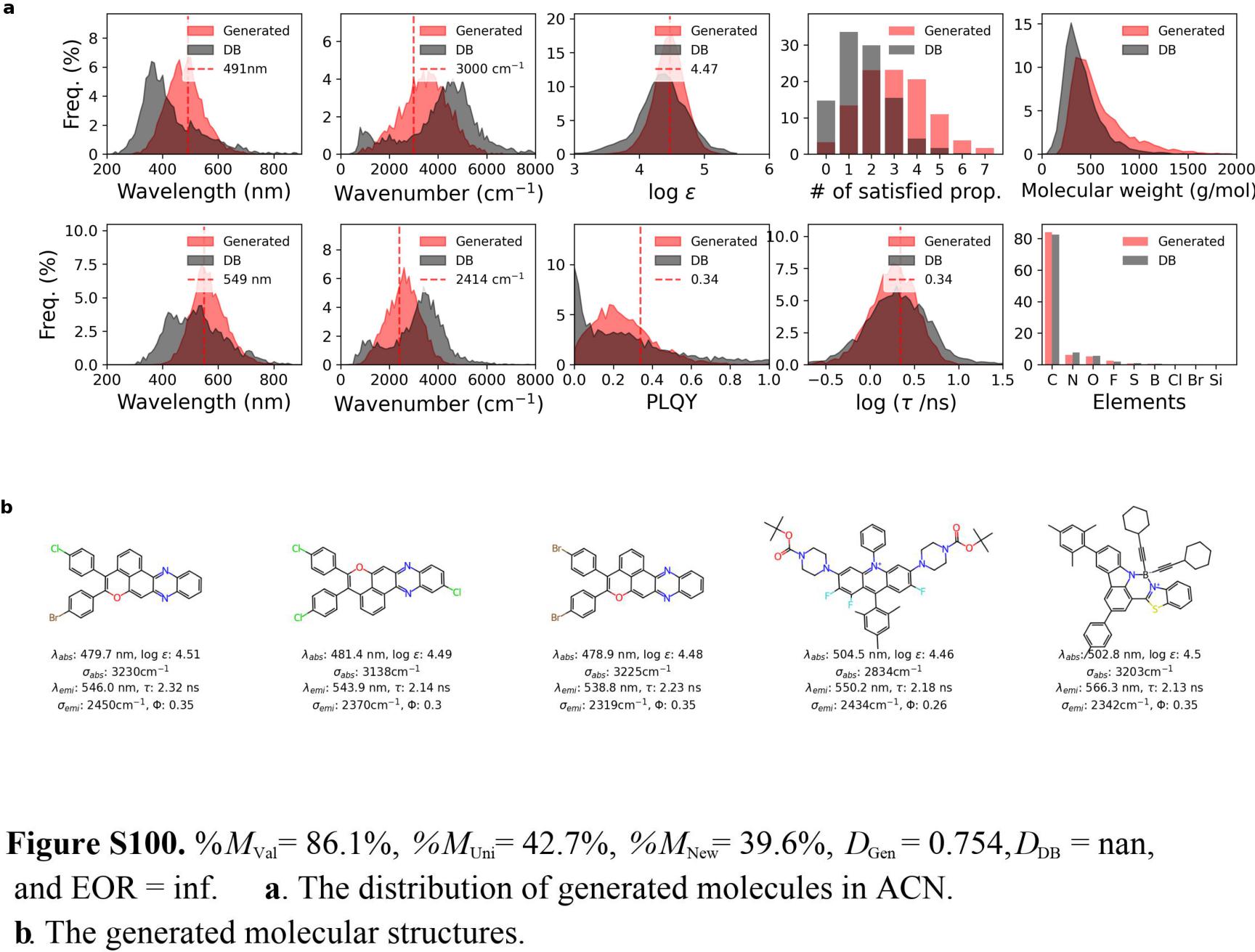

Supplement: Supplementary file 2 — oc4c00656_si_002.zip [file oc4c00656_si_002.zip › FigureS100.jpg]

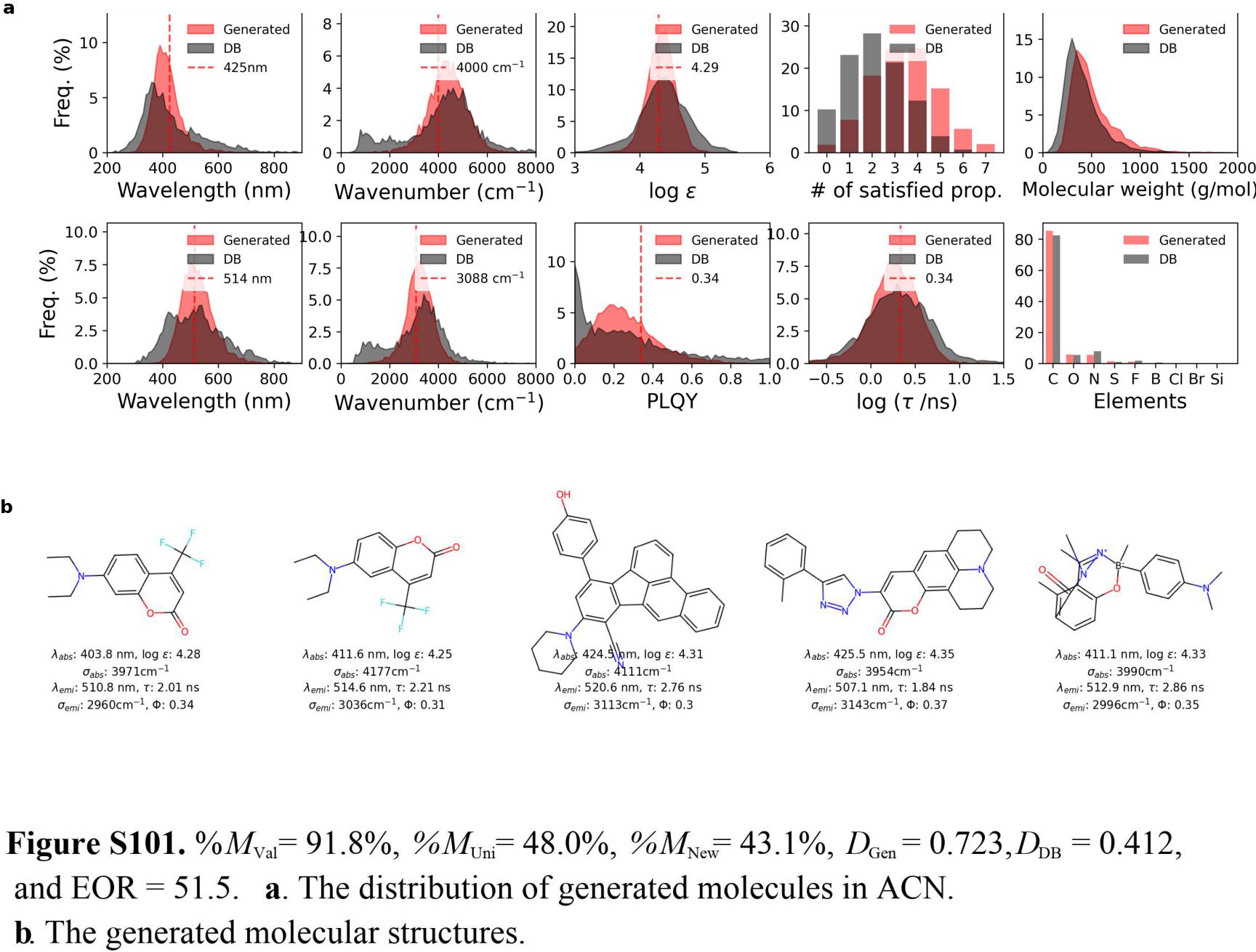

Supplement: Supplementary file 2 — oc4c00656_si_002.zip [file oc4c00656_si_002.zip › FigureS101.jpg]

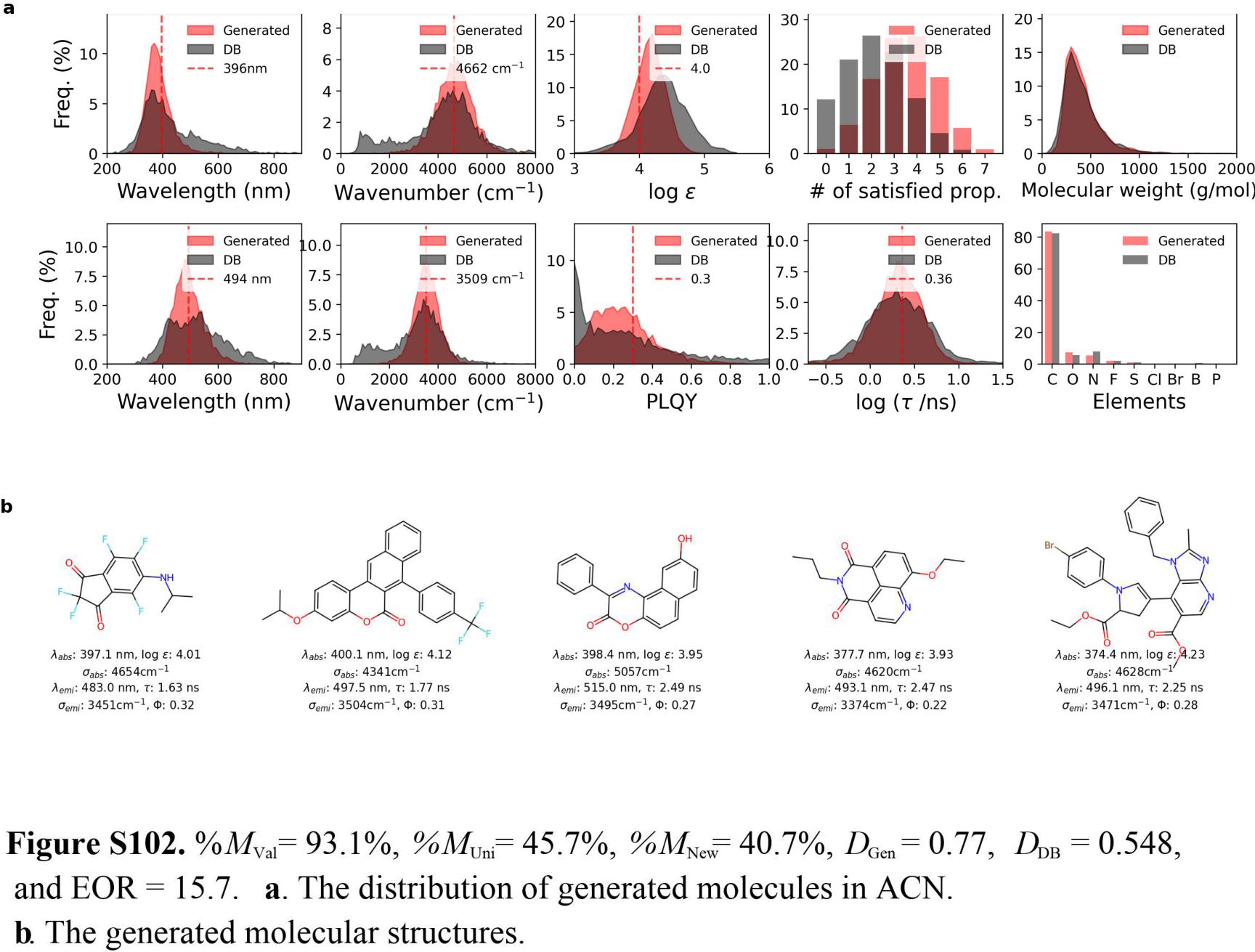

Supplement: Supplementary file 2 — oc4c00656_si_002.zip [file oc4c00656_si_002.zip › FigureS102.jpg]

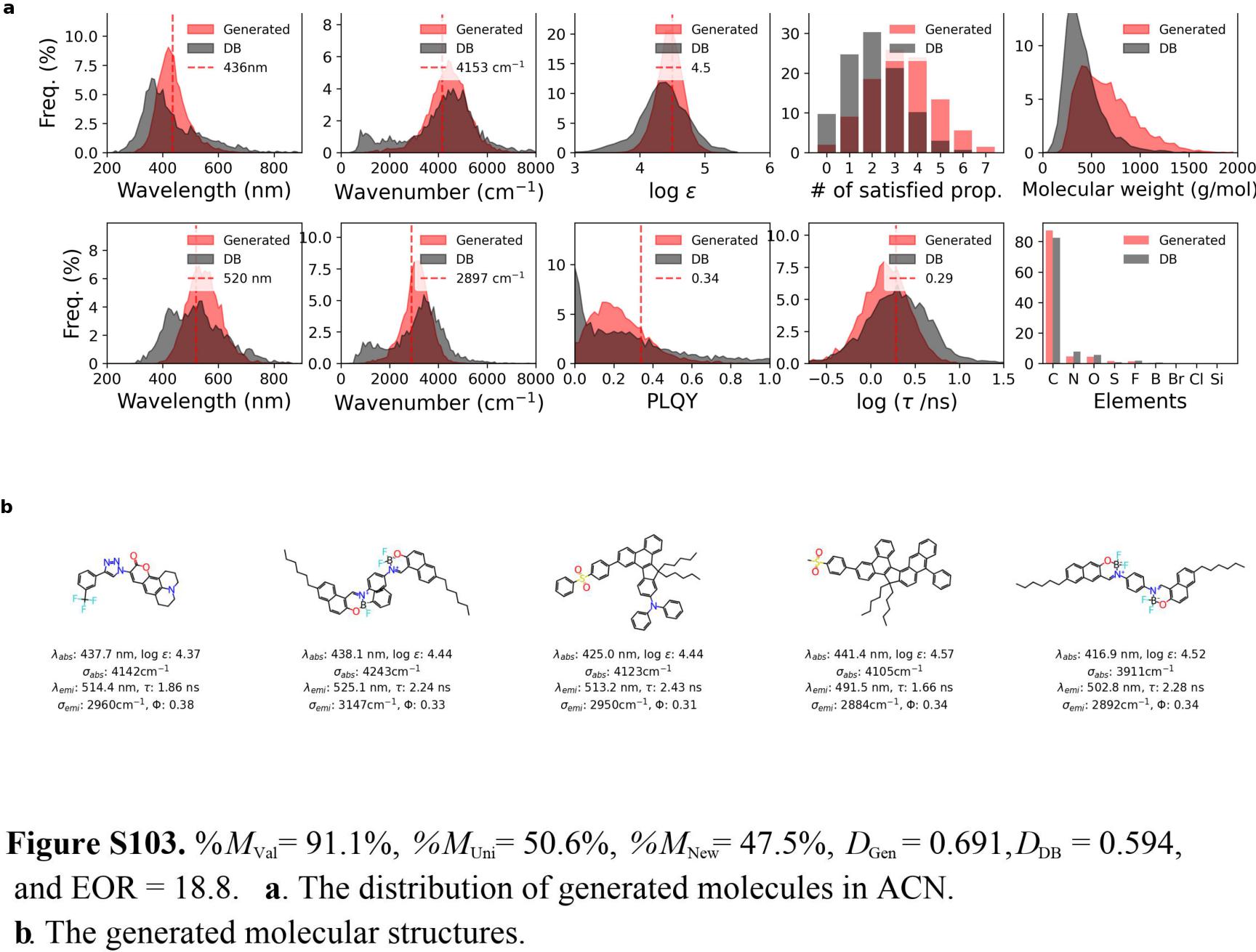

Supplement: Supplementary file 2 — oc4c00656_si_002.zip [file oc4c00656_si_002.zip › FigureS103.jpg]

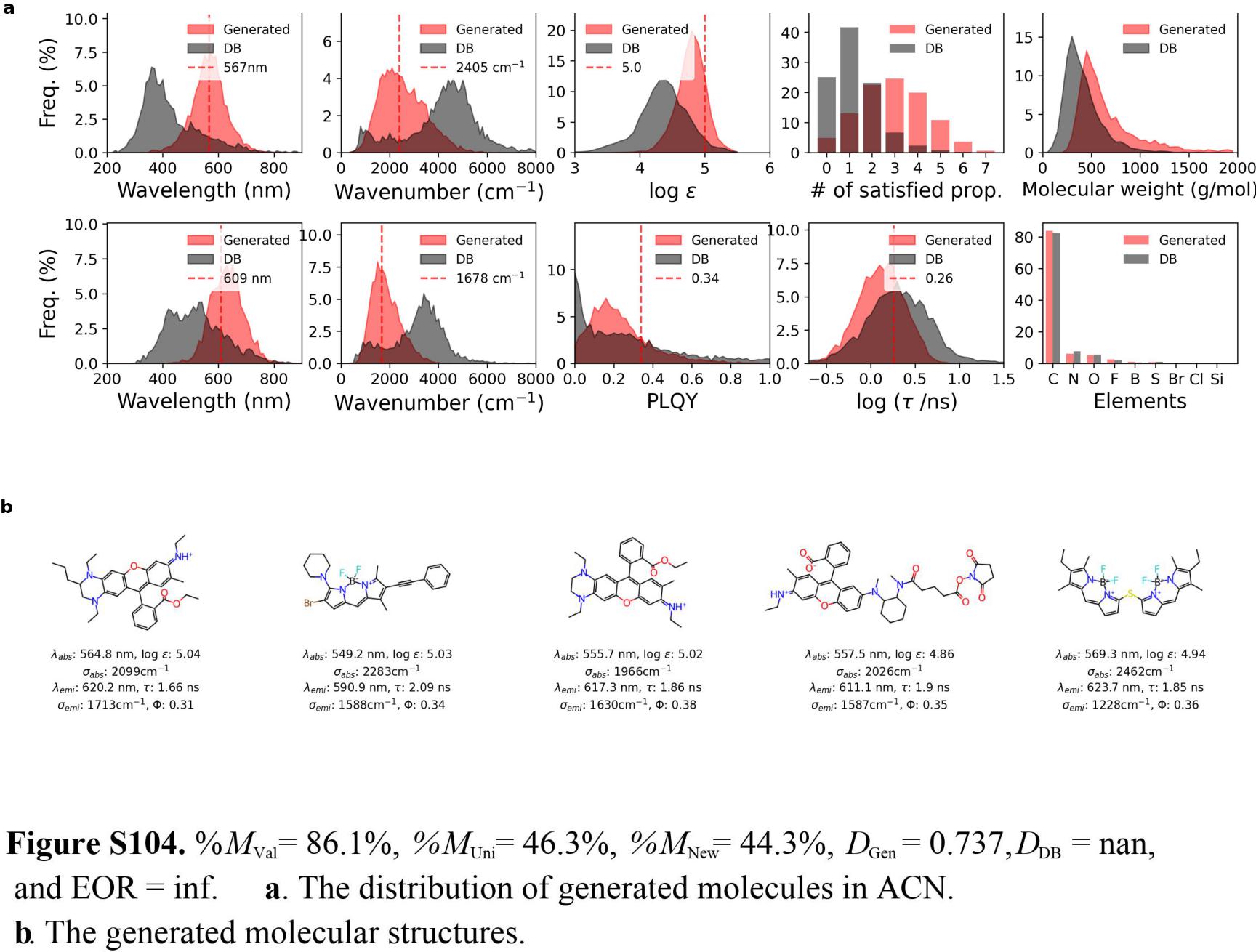

Supplement: Supplementary file 2 — oc4c00656_si_002.zip [file oc4c00656_si_002.zip › FigureS104.jpg]

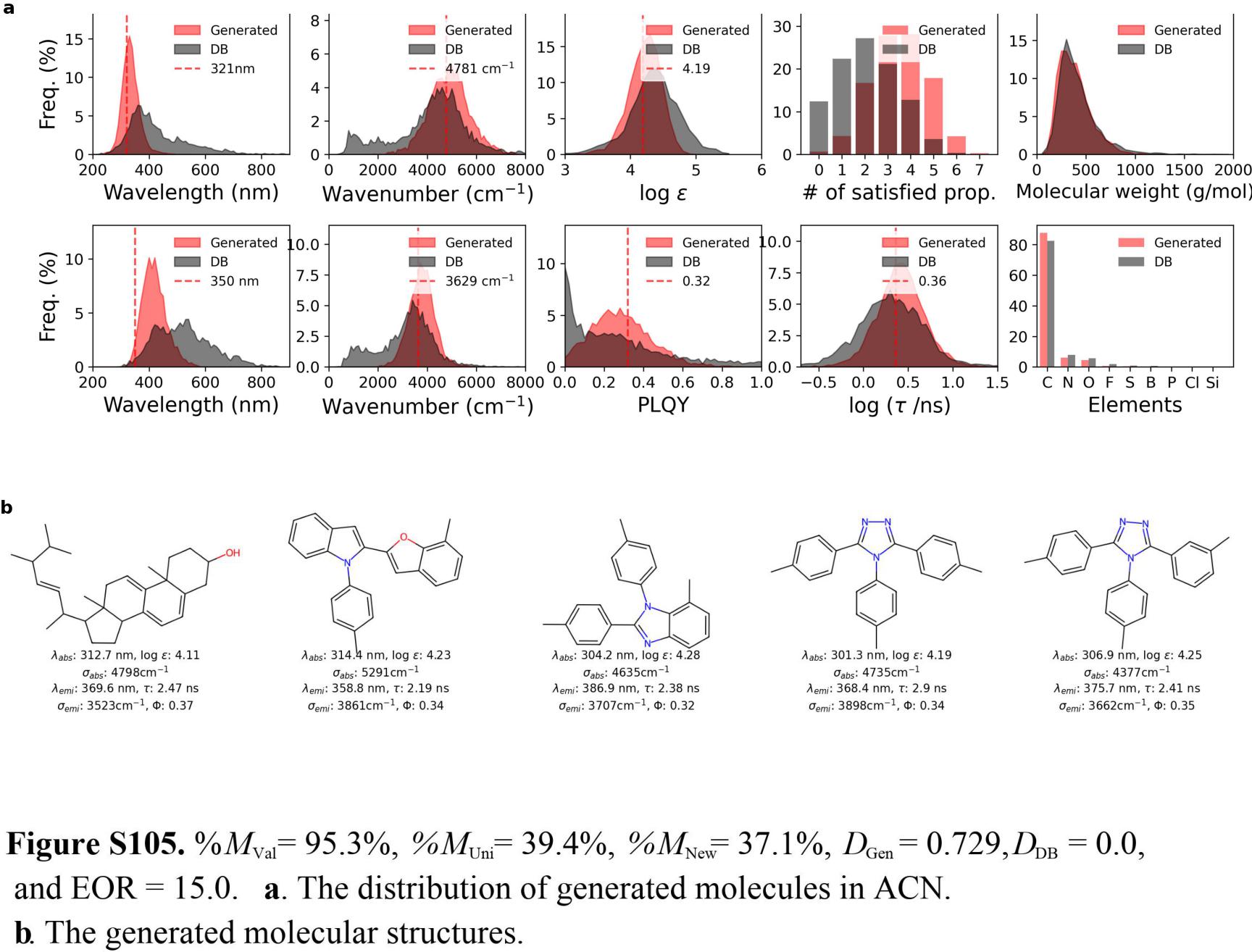

Supplement: Supplementary file 2 — oc4c00656_si_002.zip [file oc4c00656_si_002.zip › FigureS105.jpg]

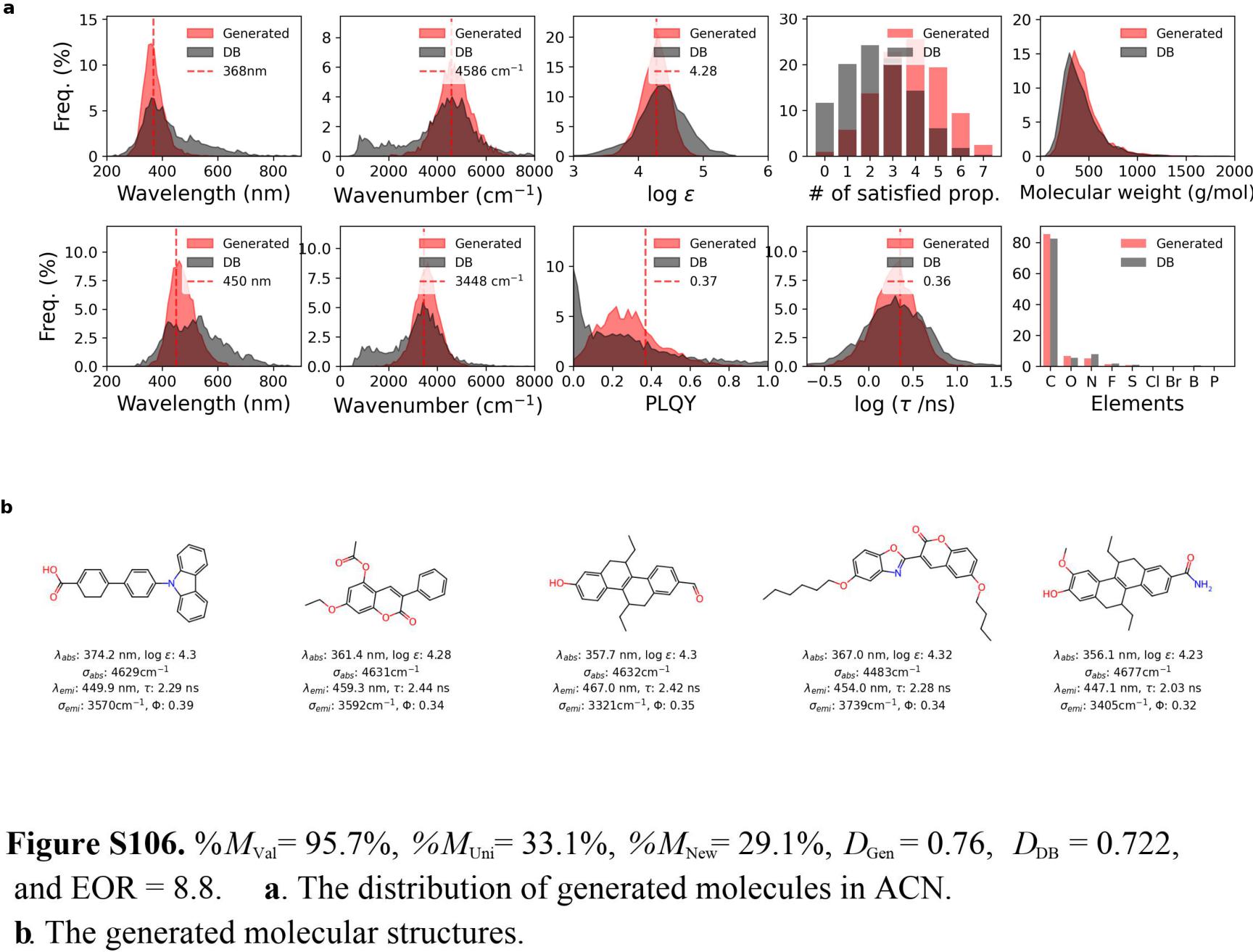

Supplement: Supplementary file 2 — oc4c00656_si_002.zip [file oc4c00656_si_002.zip › FigureS106.jpg]

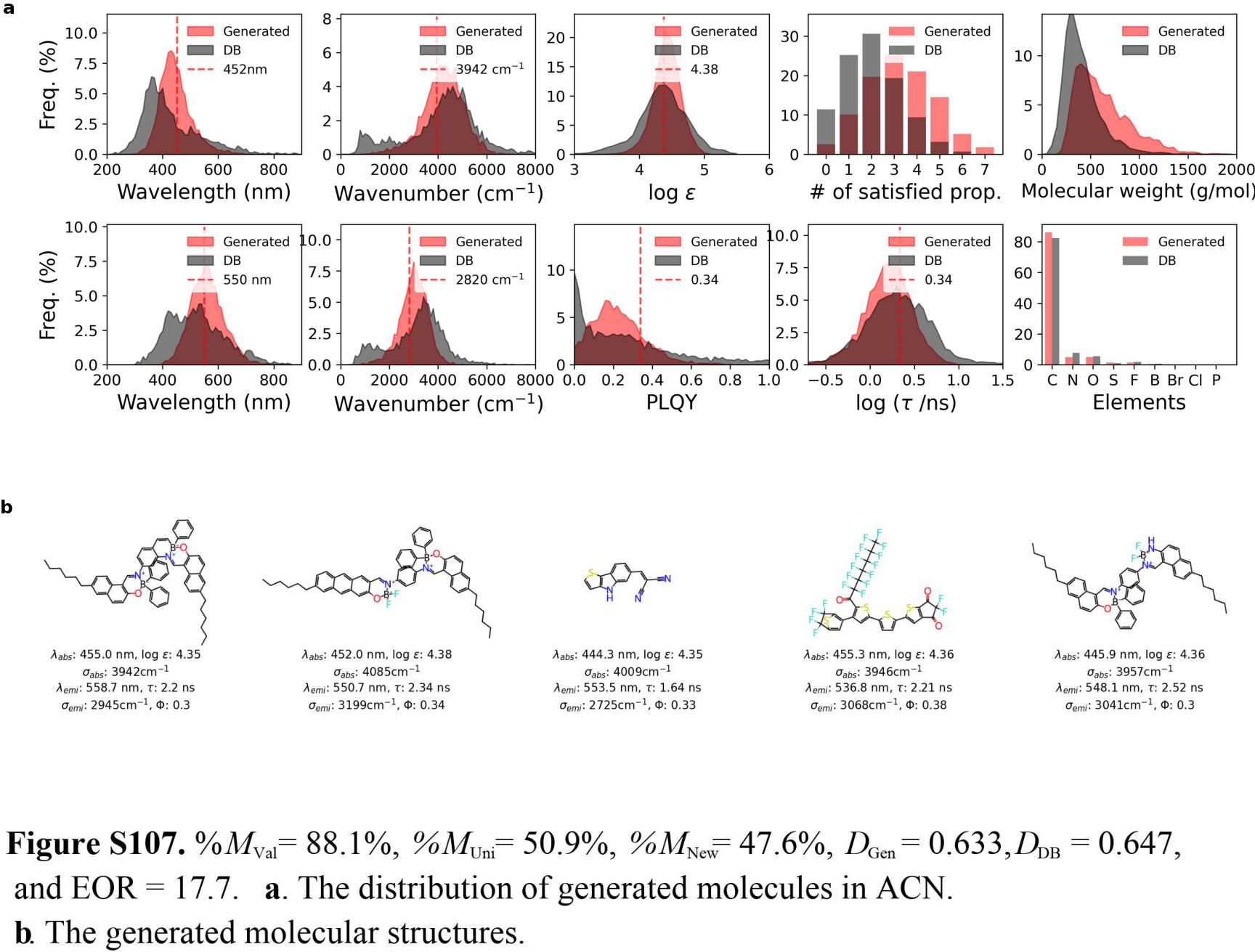

Supplement: Supplementary file 2 — oc4c00656_si_002.zip [file oc4c00656_si_002.zip › FigureS107.jpg]

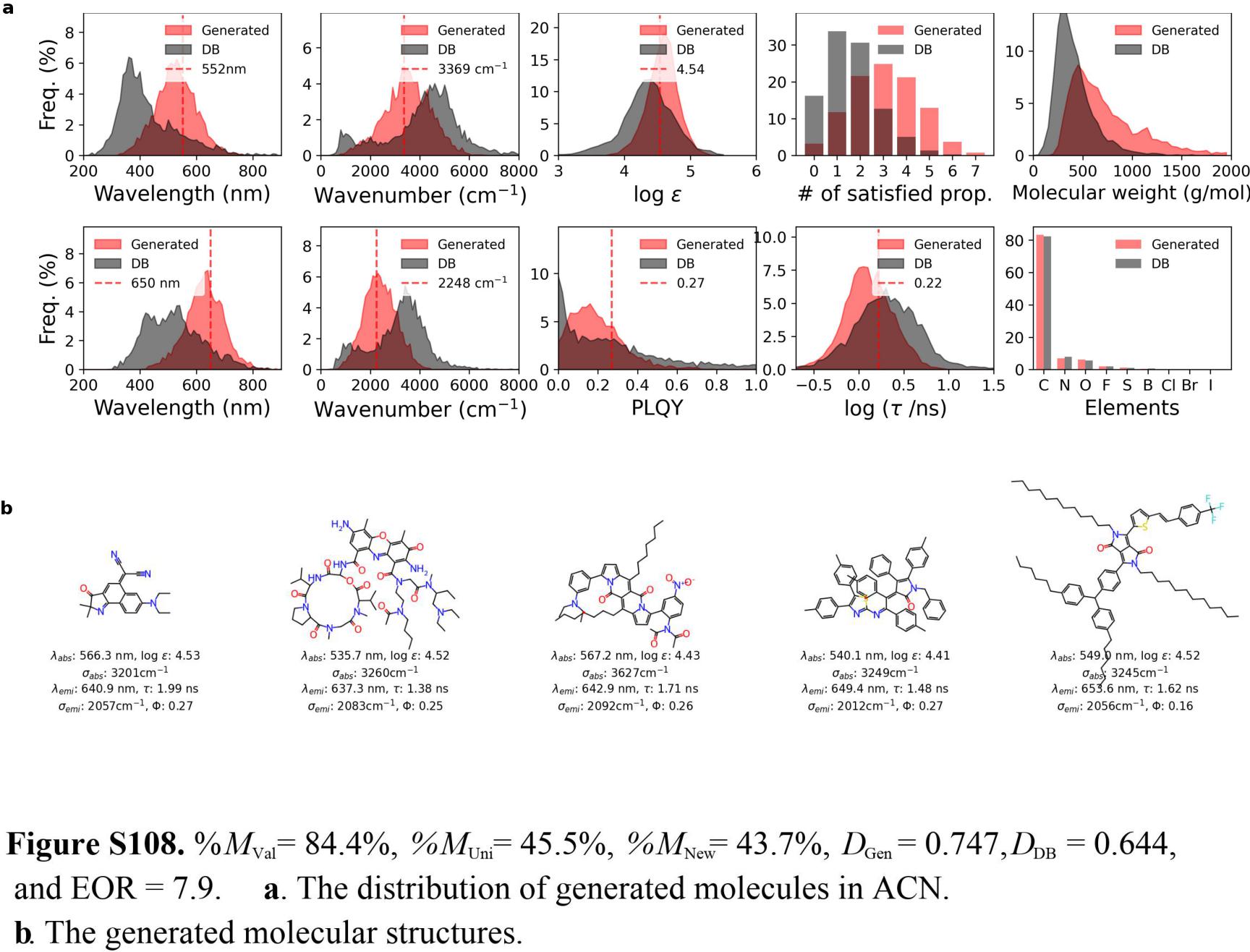

Supplement: Supplementary file 2 — oc4c00656_si_002.zip [file oc4c00656_si_002.zip › FigureS108.jpg]

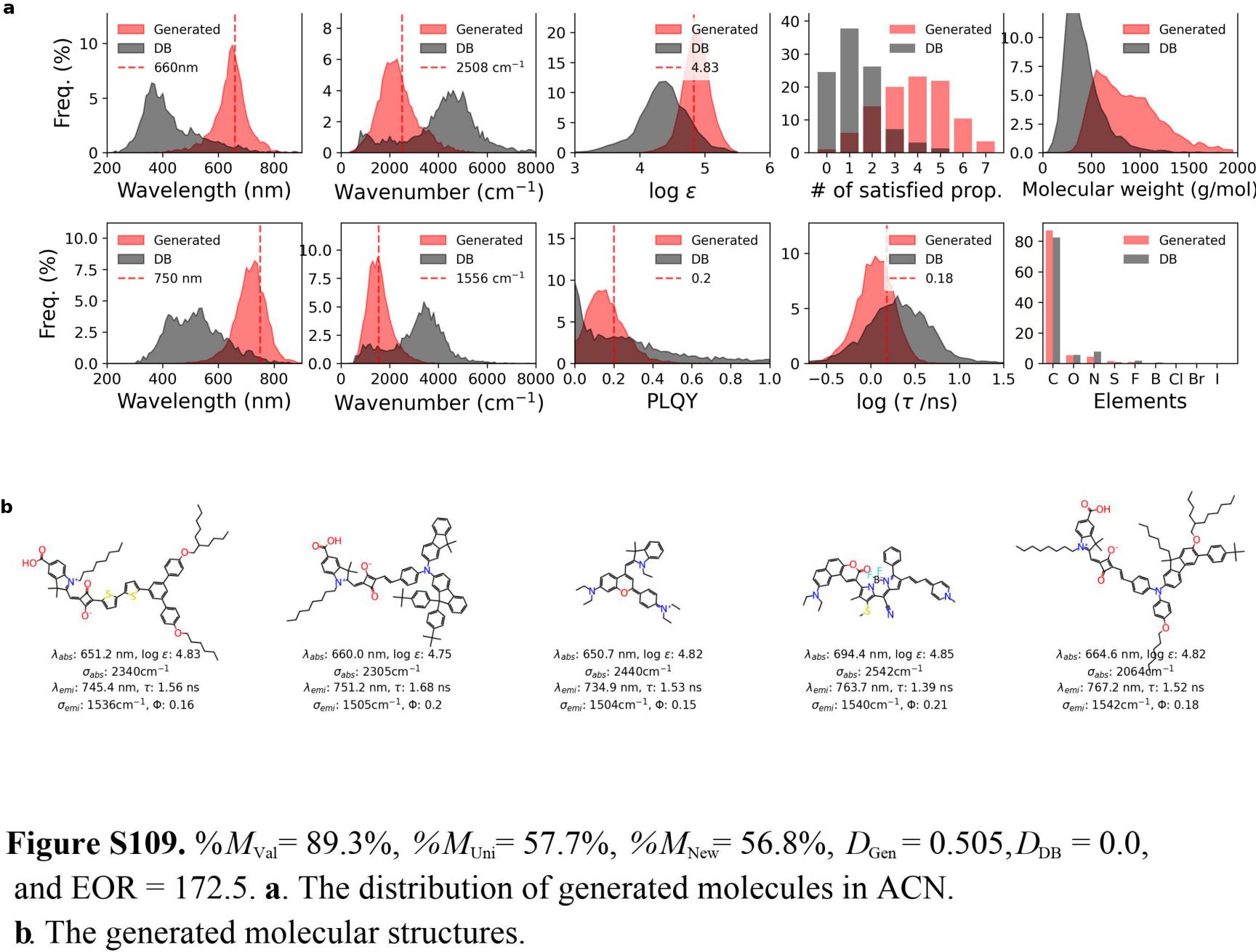

Supplement: Supplementary file 2 — oc4c00656_si_002.zip [file oc4c00656_si_002.zip › FigureS109.jpg]

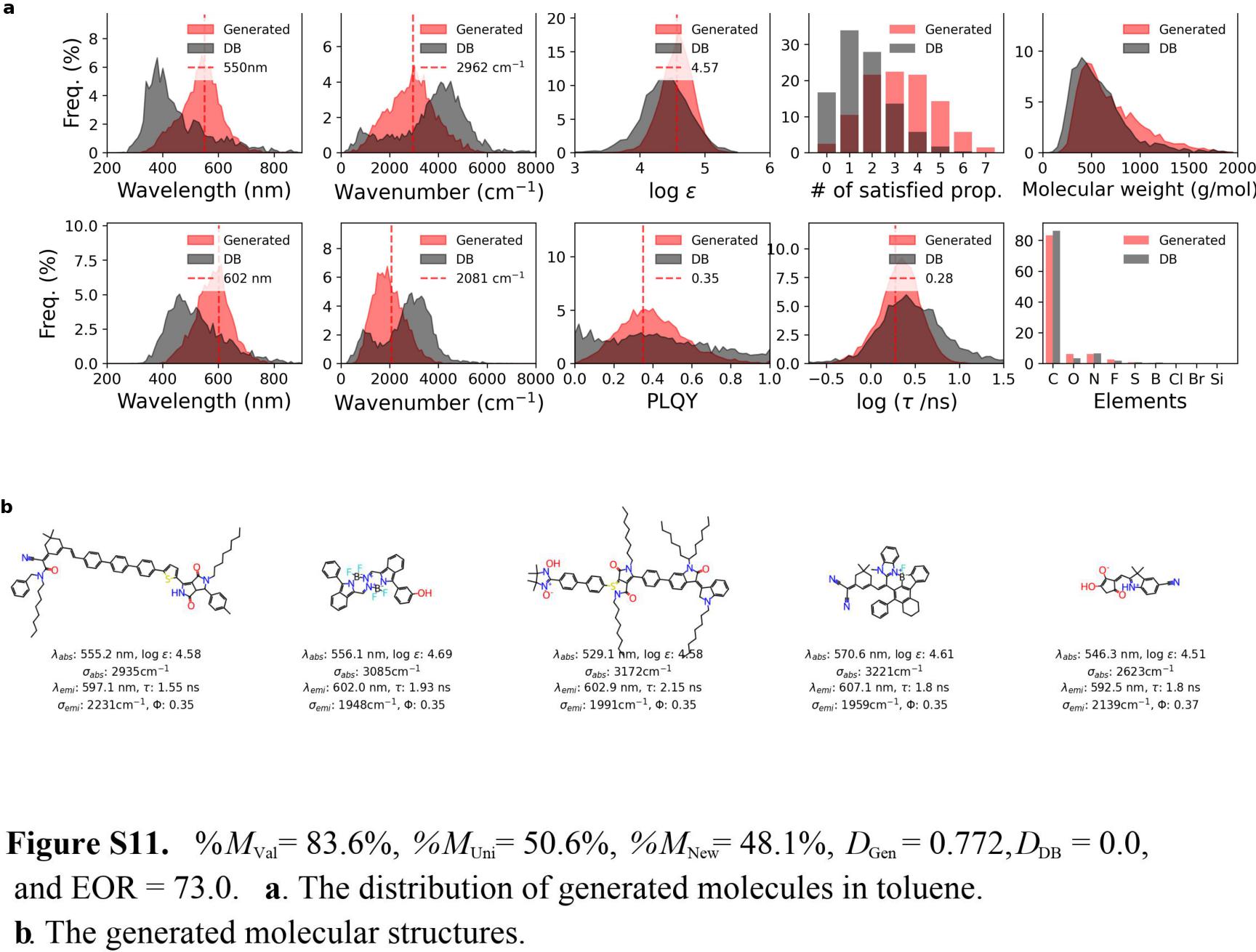

Supplement: Supplementary file 2 — oc4c00656_si_002.zip [file oc4c00656_si_002.zip › FigureS11.jpg]

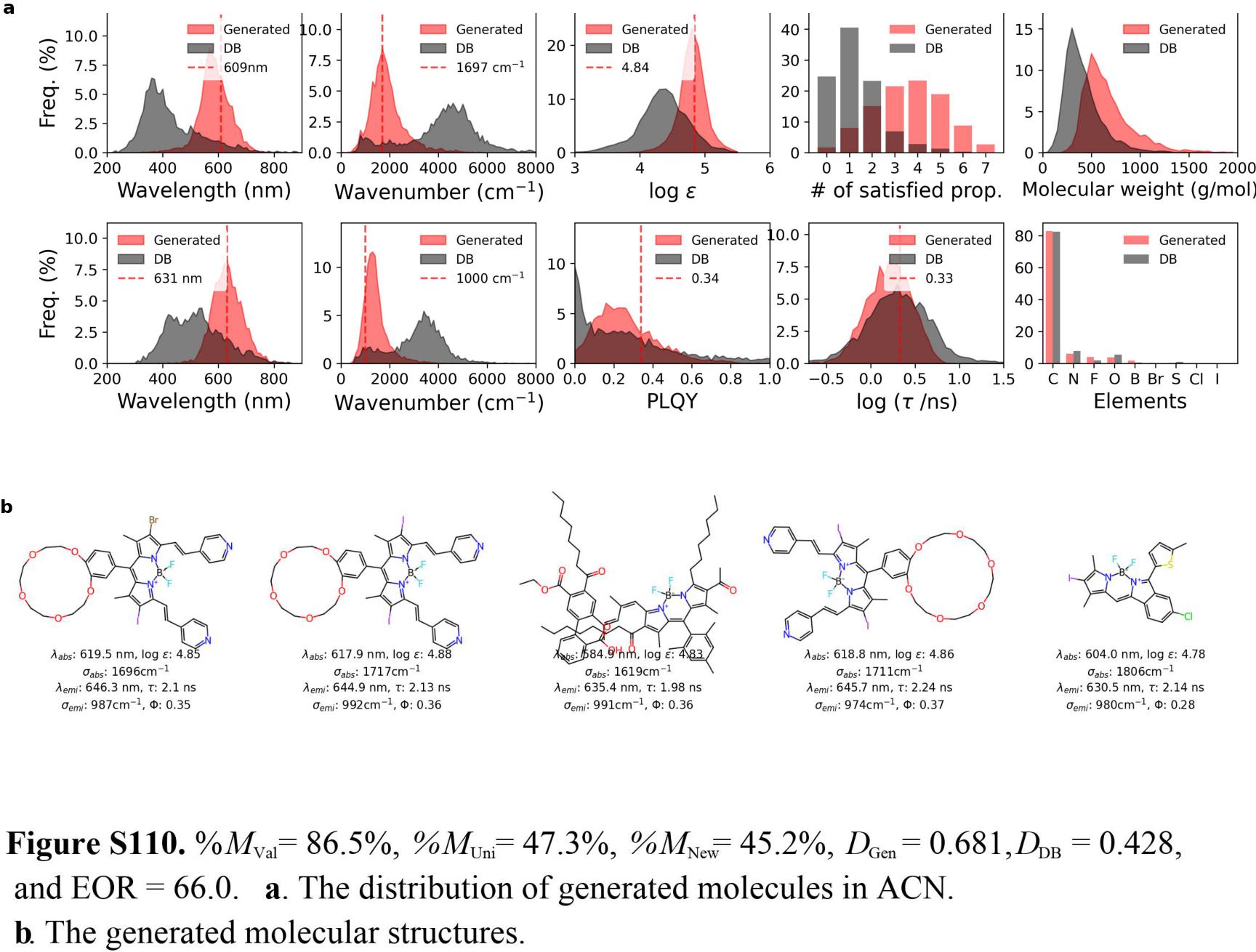

Supplement: Supplementary file 2 — oc4c00656_si_002.zip [file oc4c00656_si_002.zip › FigureS110.jpg]

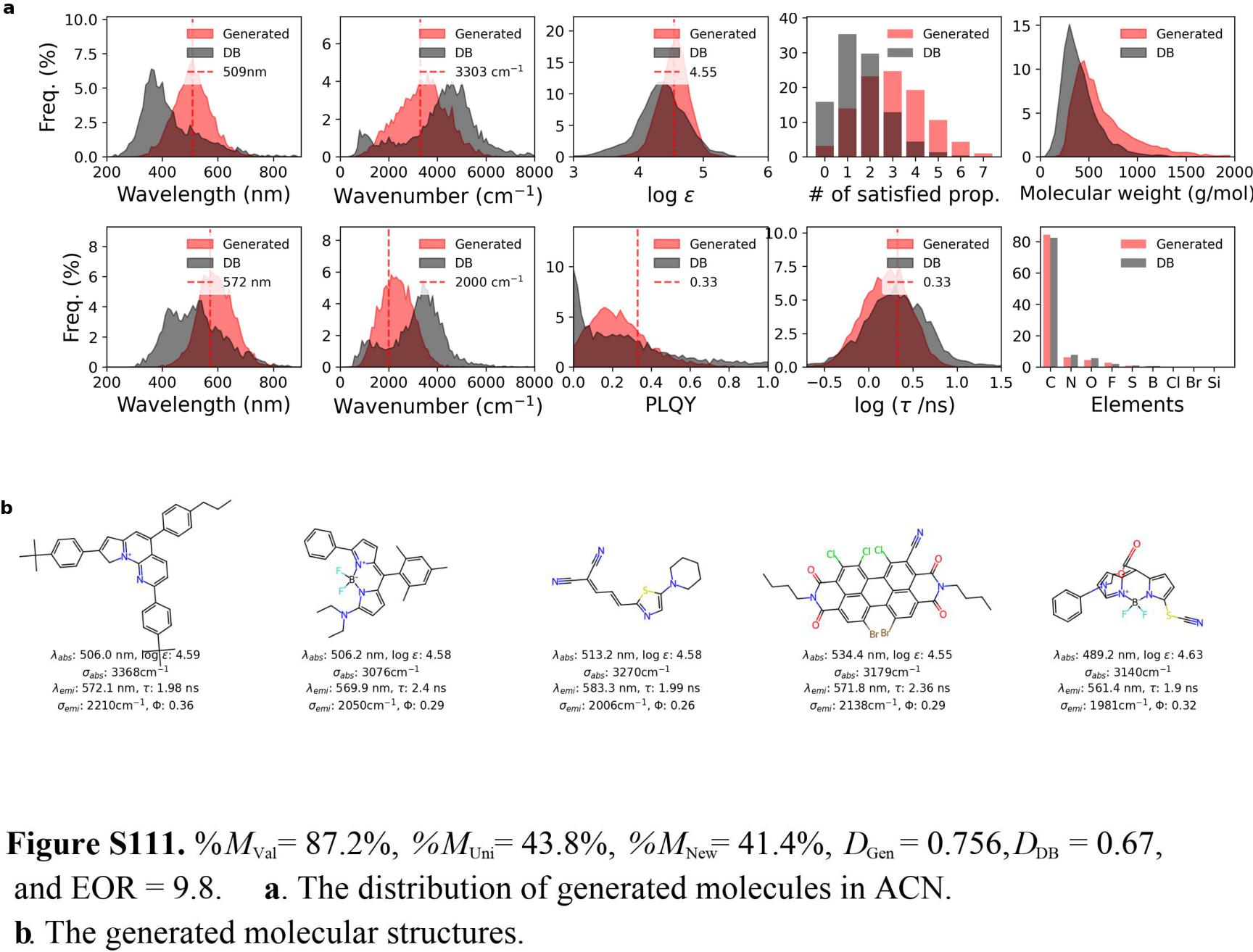

Supplement: Supplementary file 2 — oc4c00656_si_002.zip [file oc4c00656_si_002.zip › FigureS111.jpg]

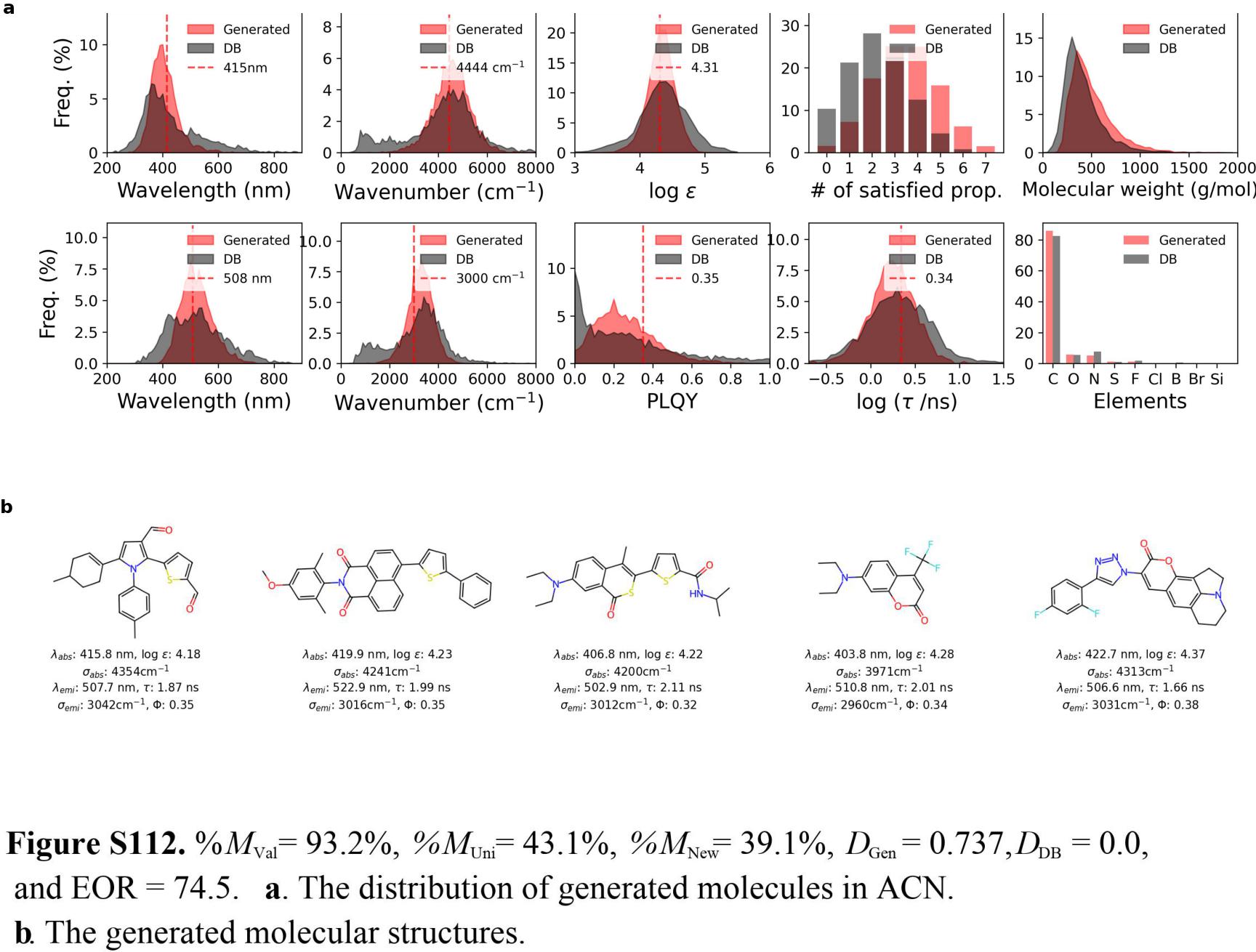

Supplement: Supplementary file 2 — oc4c00656_si_002.zip [file oc4c00656_si_002.zip › FigureS112.jpg]

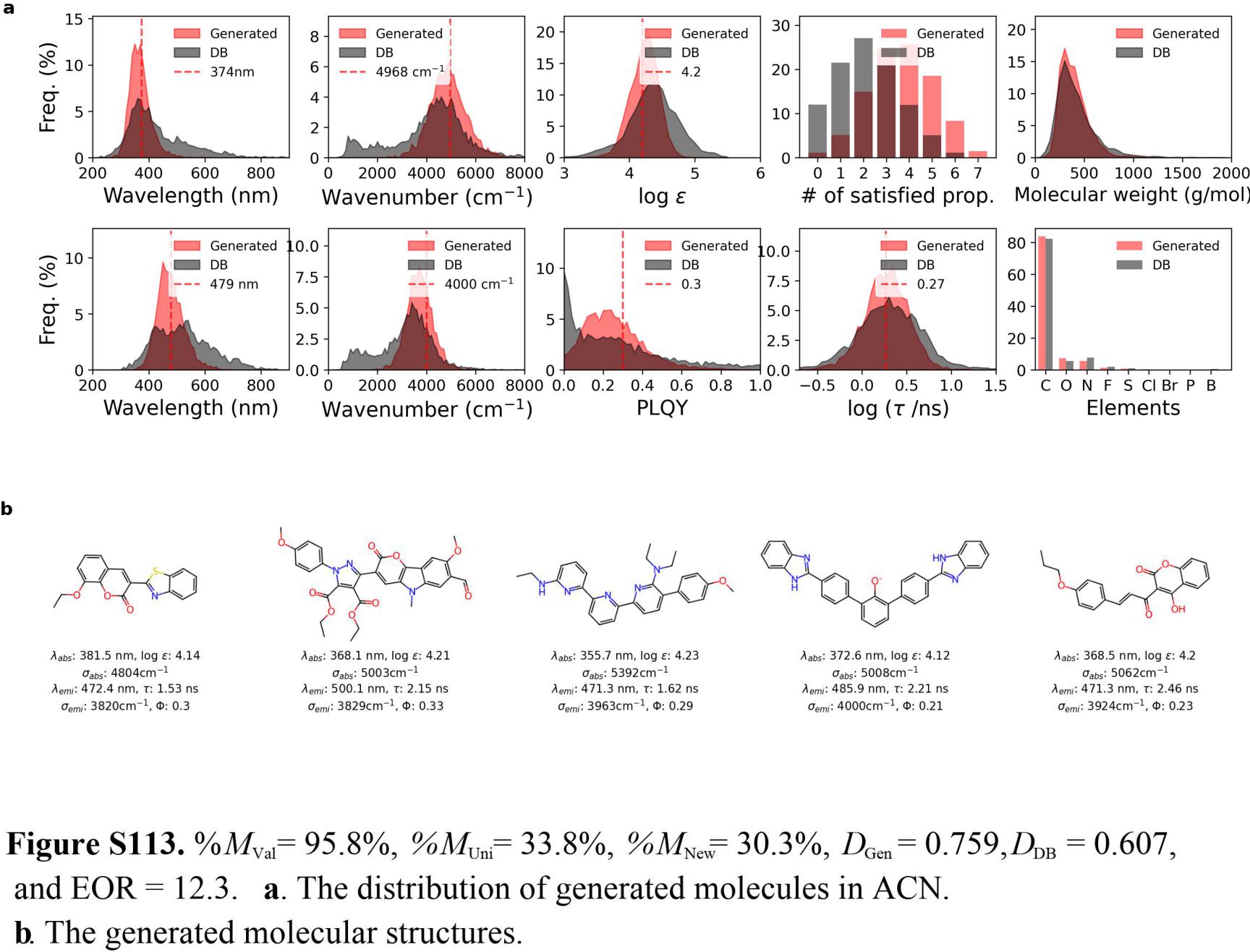

Supplement: Supplementary file 2 — oc4c00656_si_002.zip [file oc4c00656_si_002.zip › FigureS113.jpg]

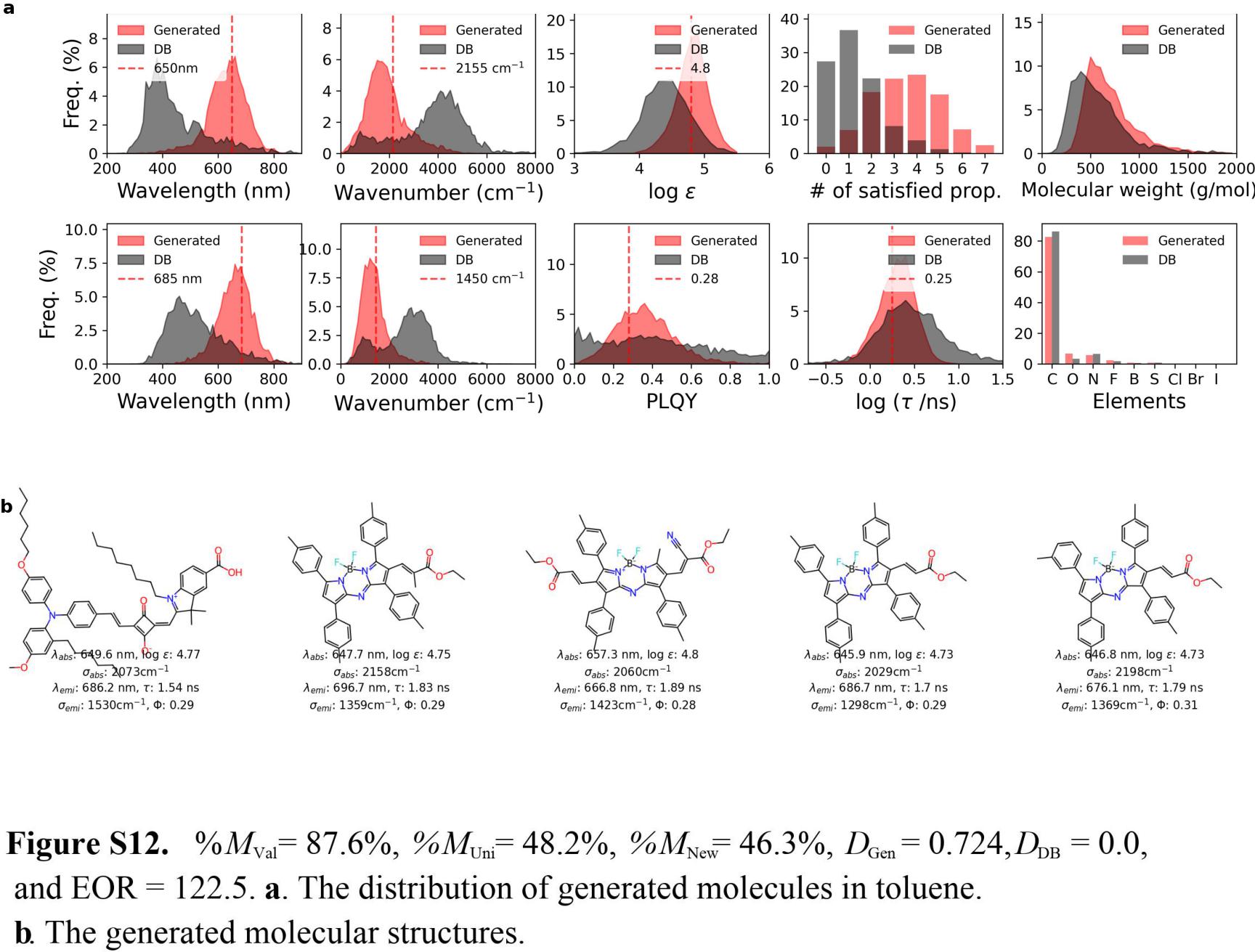

Supplement: Supplementary file 2 — oc4c00656_si_002.zip [file oc4c00656_si_002.zip › FigureS12.jpg]

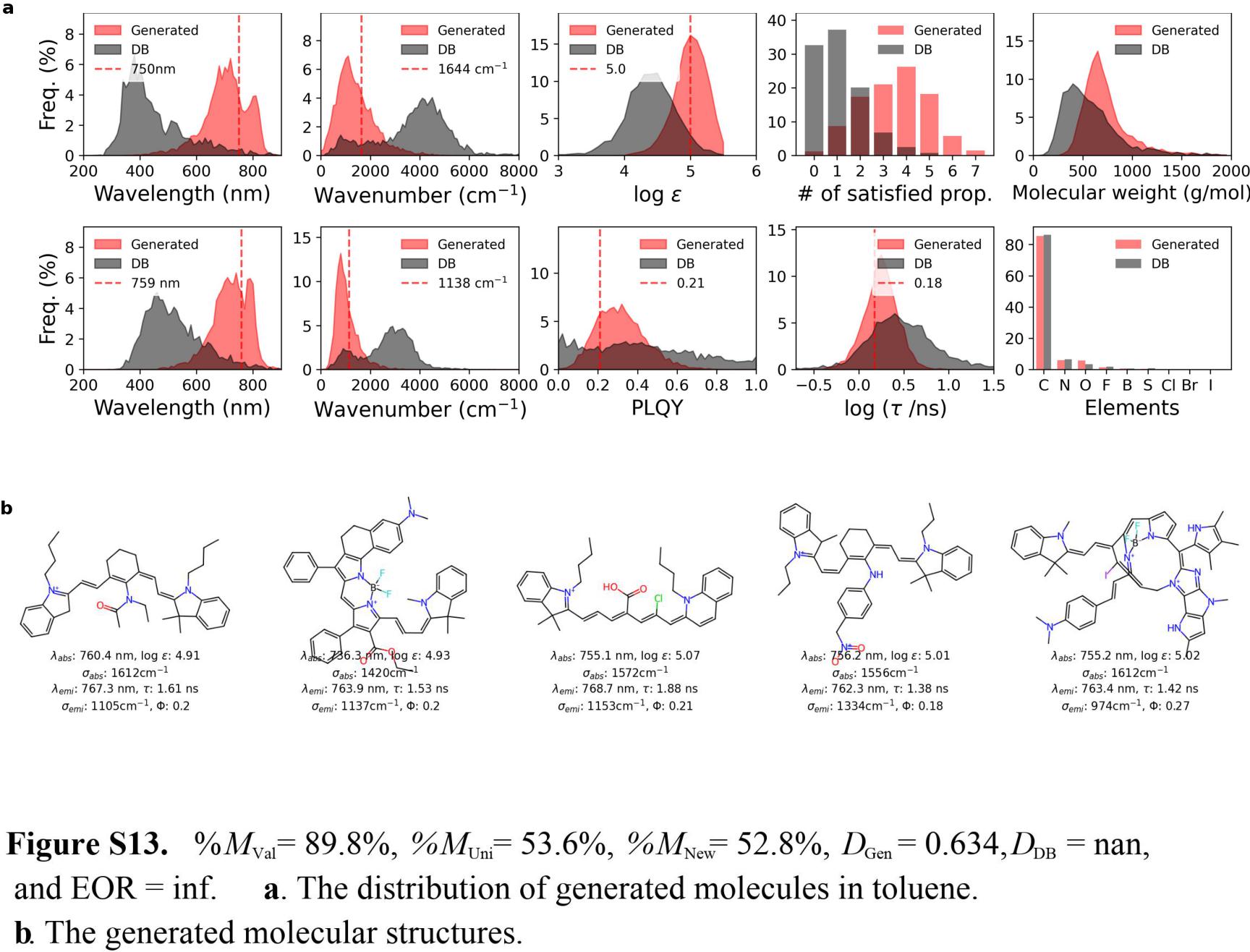

Supplement: Supplementary file 2 — oc4c00656_si_002.zip [file oc4c00656_si_002.zip › FigureS13.jpg]

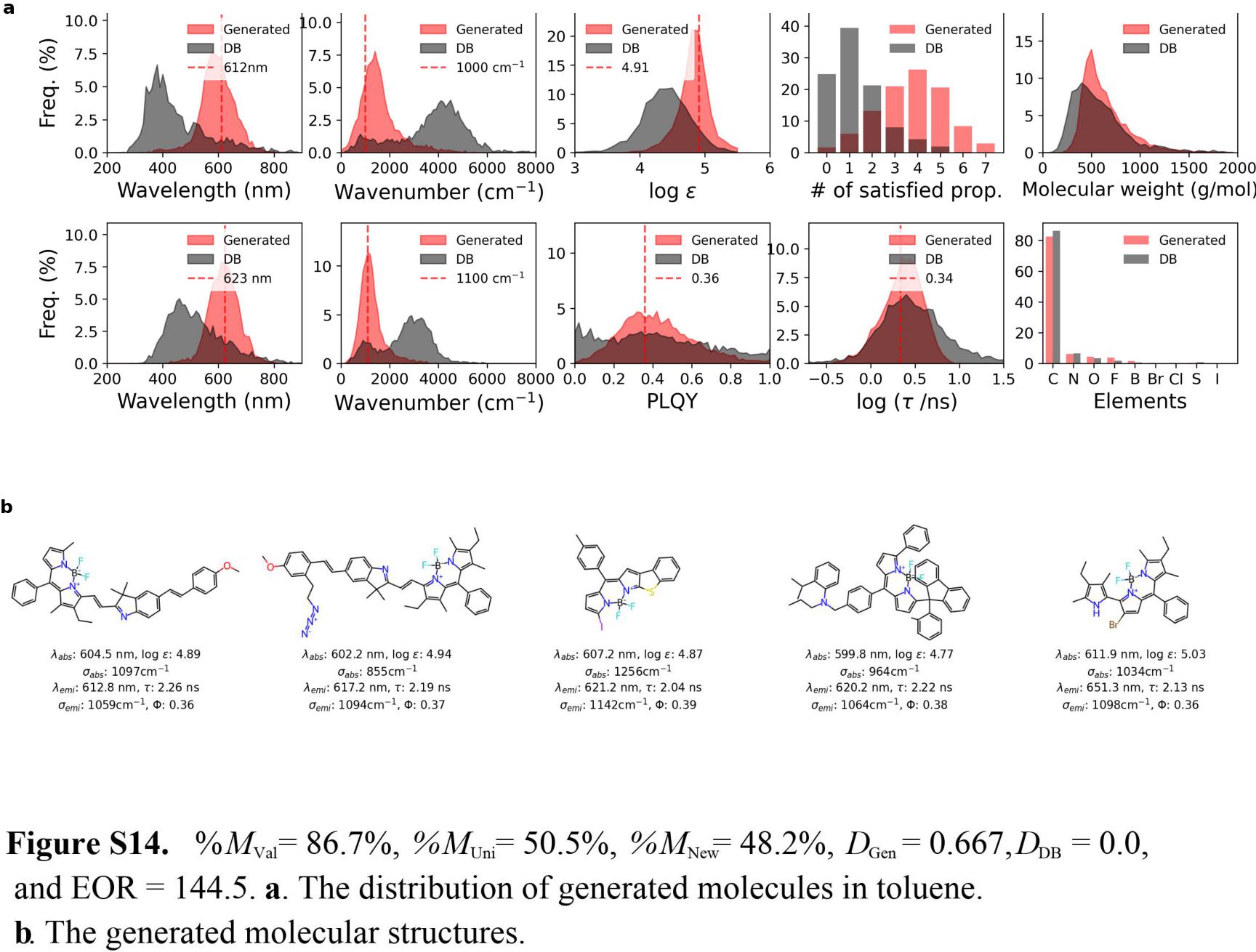

Supplement: Supplementary file 2 — oc4c00656_si_002.zip [file oc4c00656_si_002.zip › FigureS14.jpg]

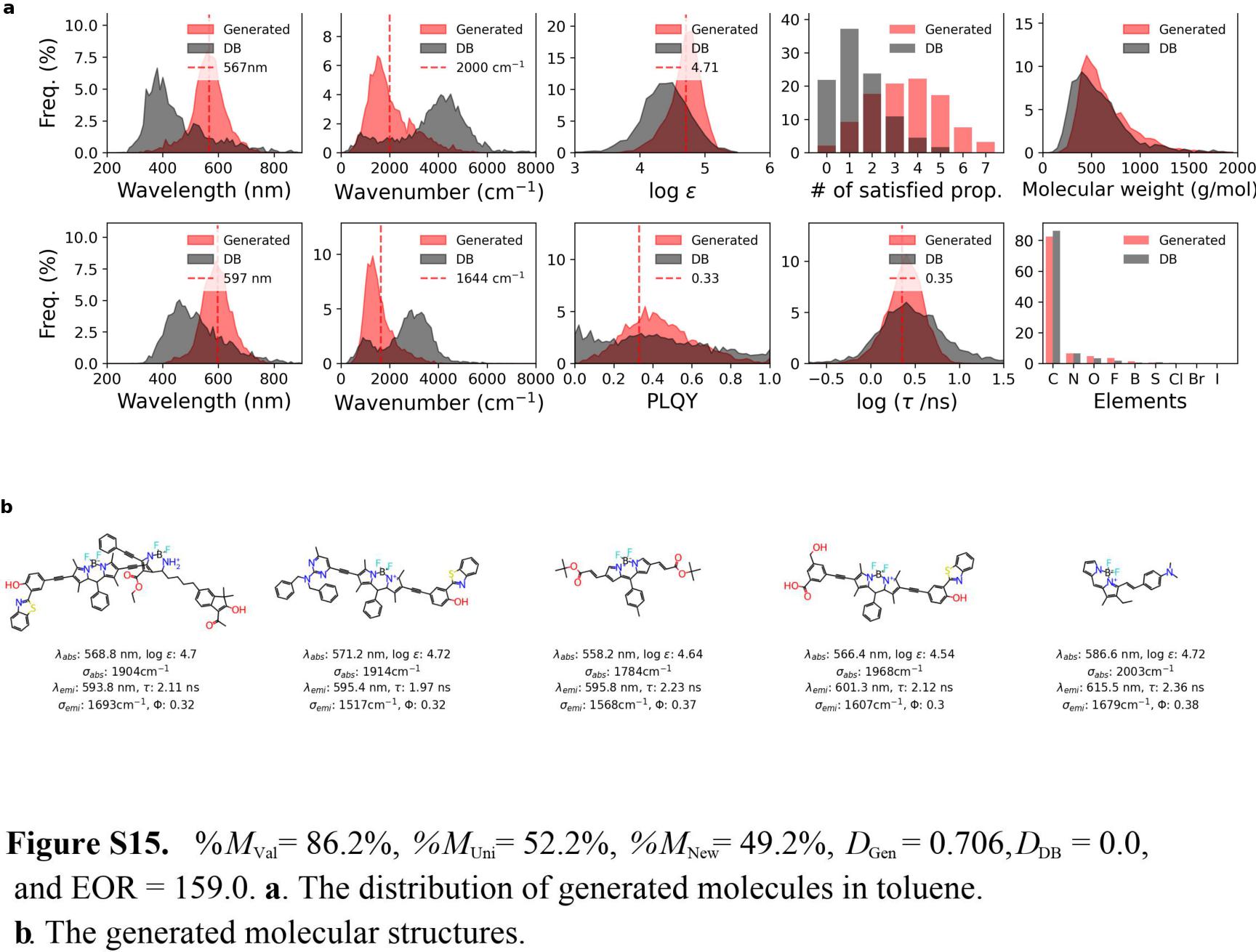

Supplement: Supplementary file 2 — oc4c00656_si_002.zip [file oc4c00656_si_002.zip › FigureS15.jpg]

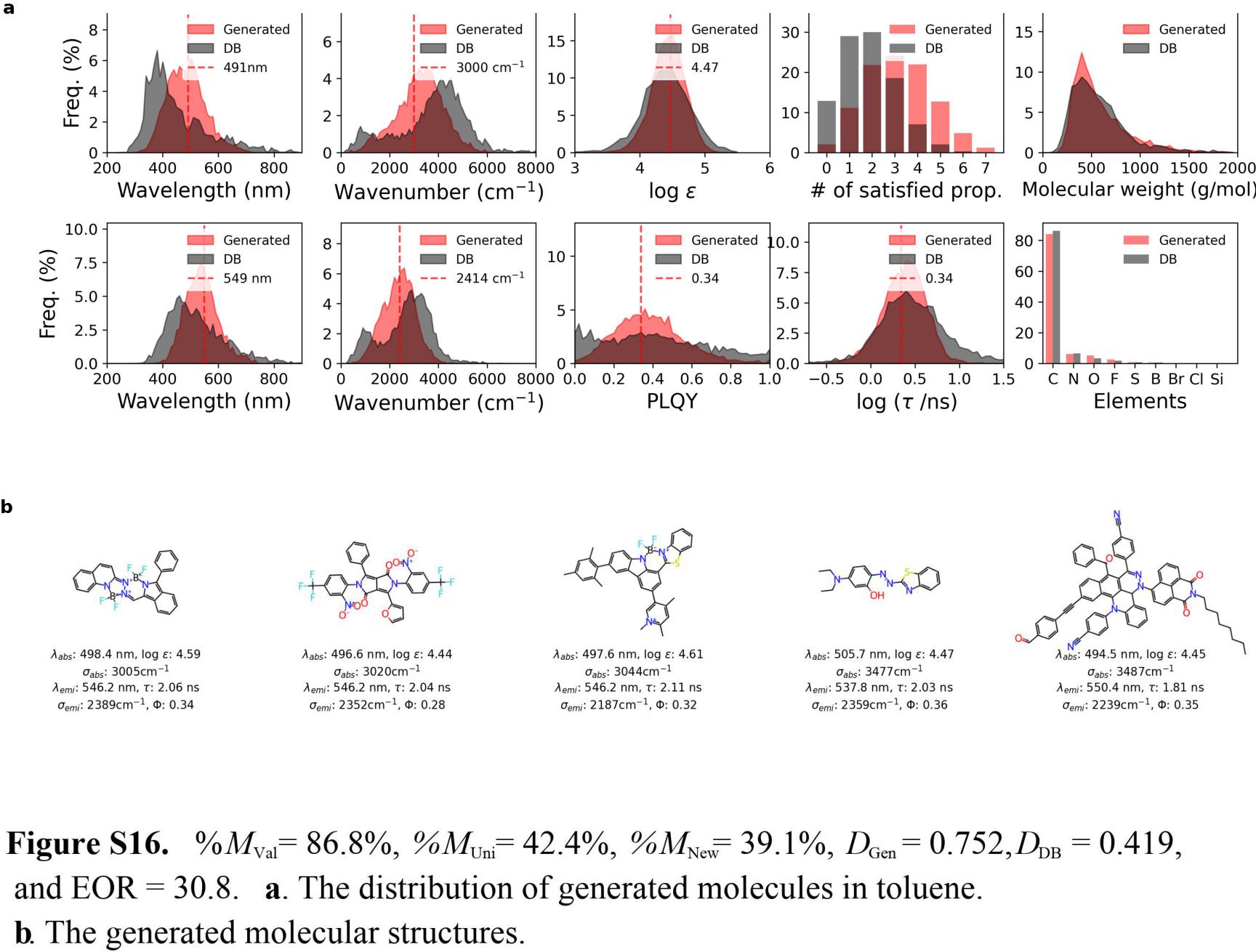

Supplement: Supplementary file 2 — oc4c00656_si_002.zip [file oc4c00656_si_002.zip › FigureS16.jpg]

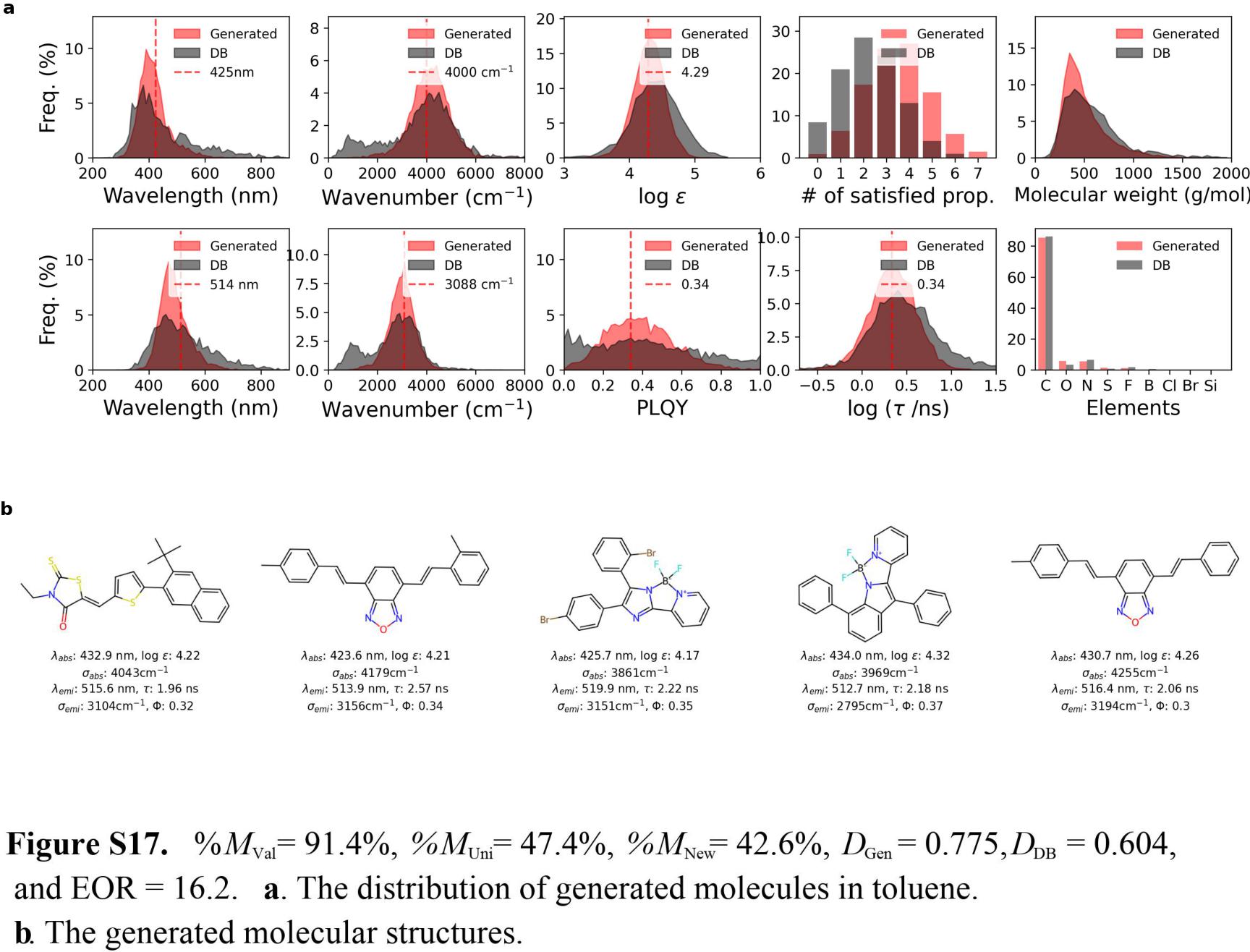

Supplement: Supplementary file 2 — oc4c00656_si_002.zip [file oc4c00656_si_002.zip › FigureS17.jpg]

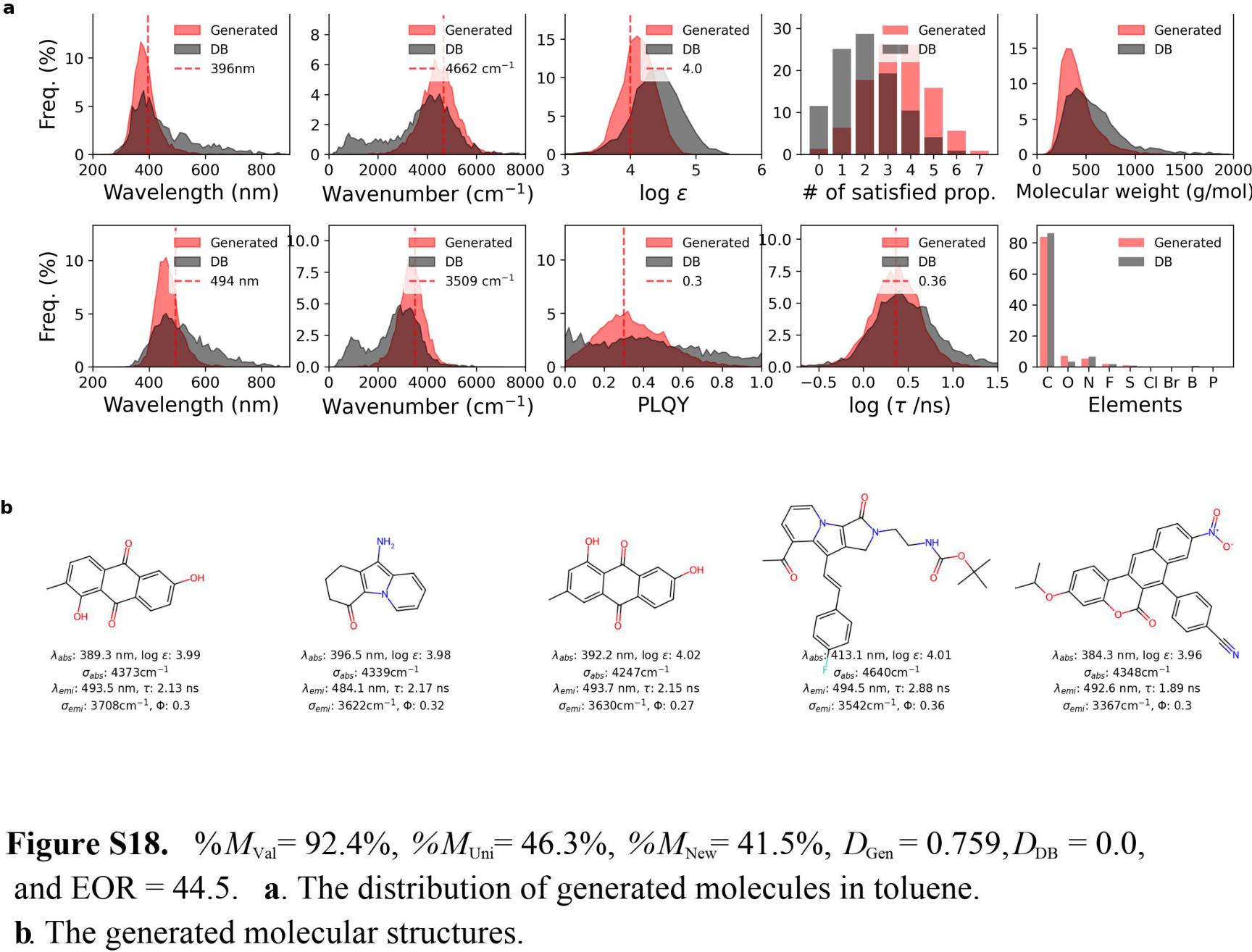

Supplement: Supplementary file 2 — oc4c00656_si_002.zip [file oc4c00656_si_002.zip › FigureS18.jpg]

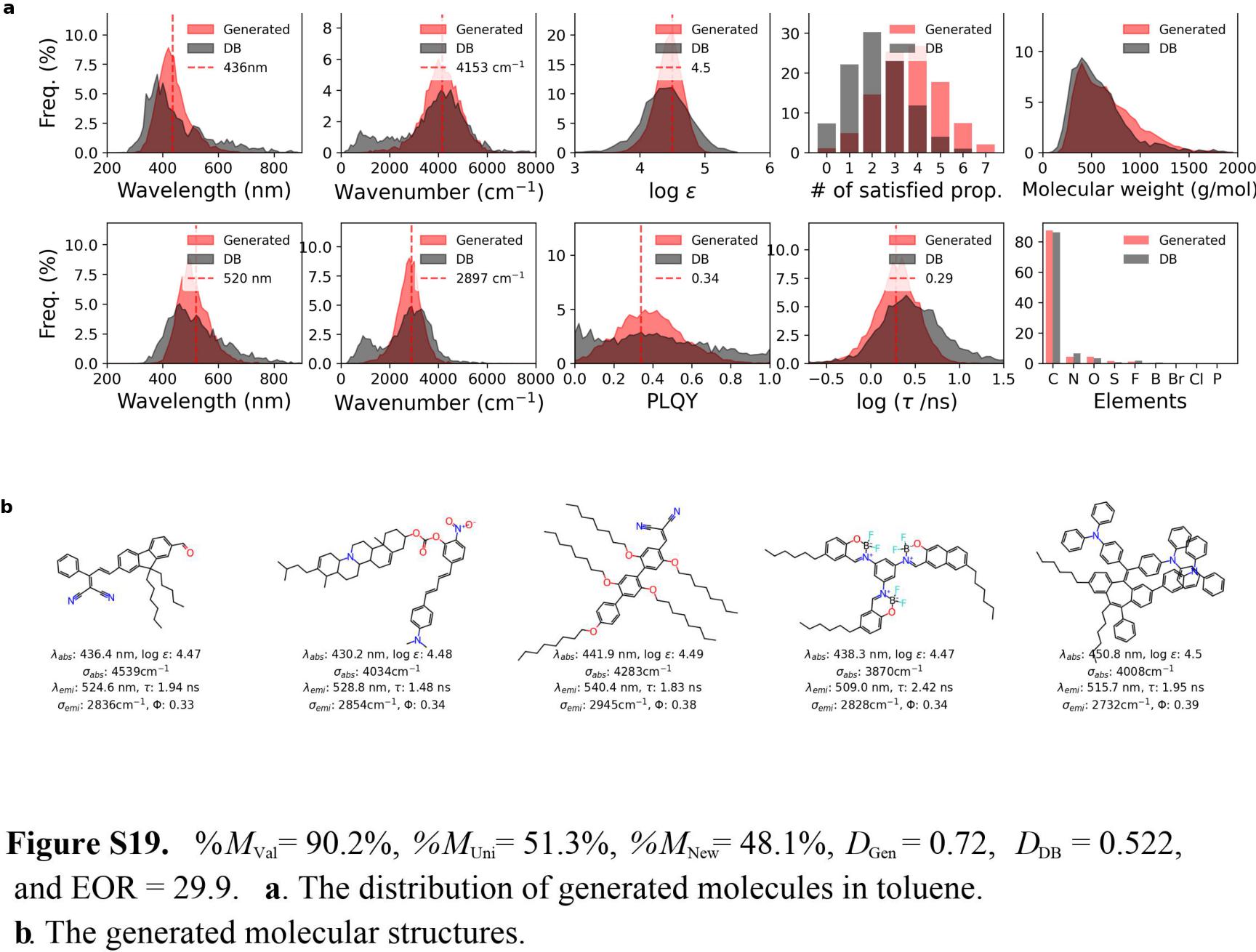

Supplement: Supplementary file 2 — oc4c00656_si_002.zip [file oc4c00656_si_002.zip › FigureS19.jpg]

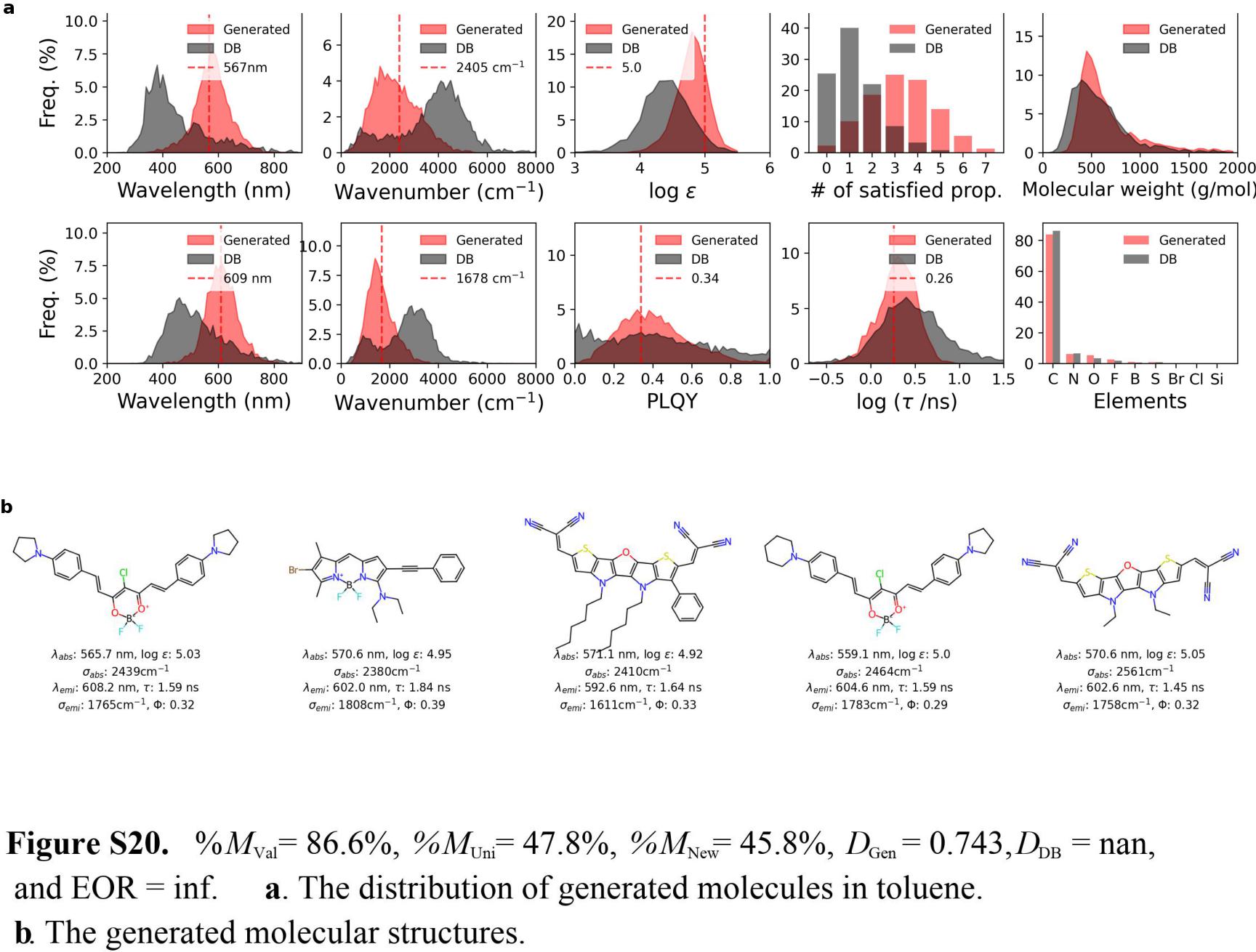

Supplement: Supplementary file 2 — oc4c00656_si_002.zip [file oc4c00656_si_002.zip › FigureS20.jpg]

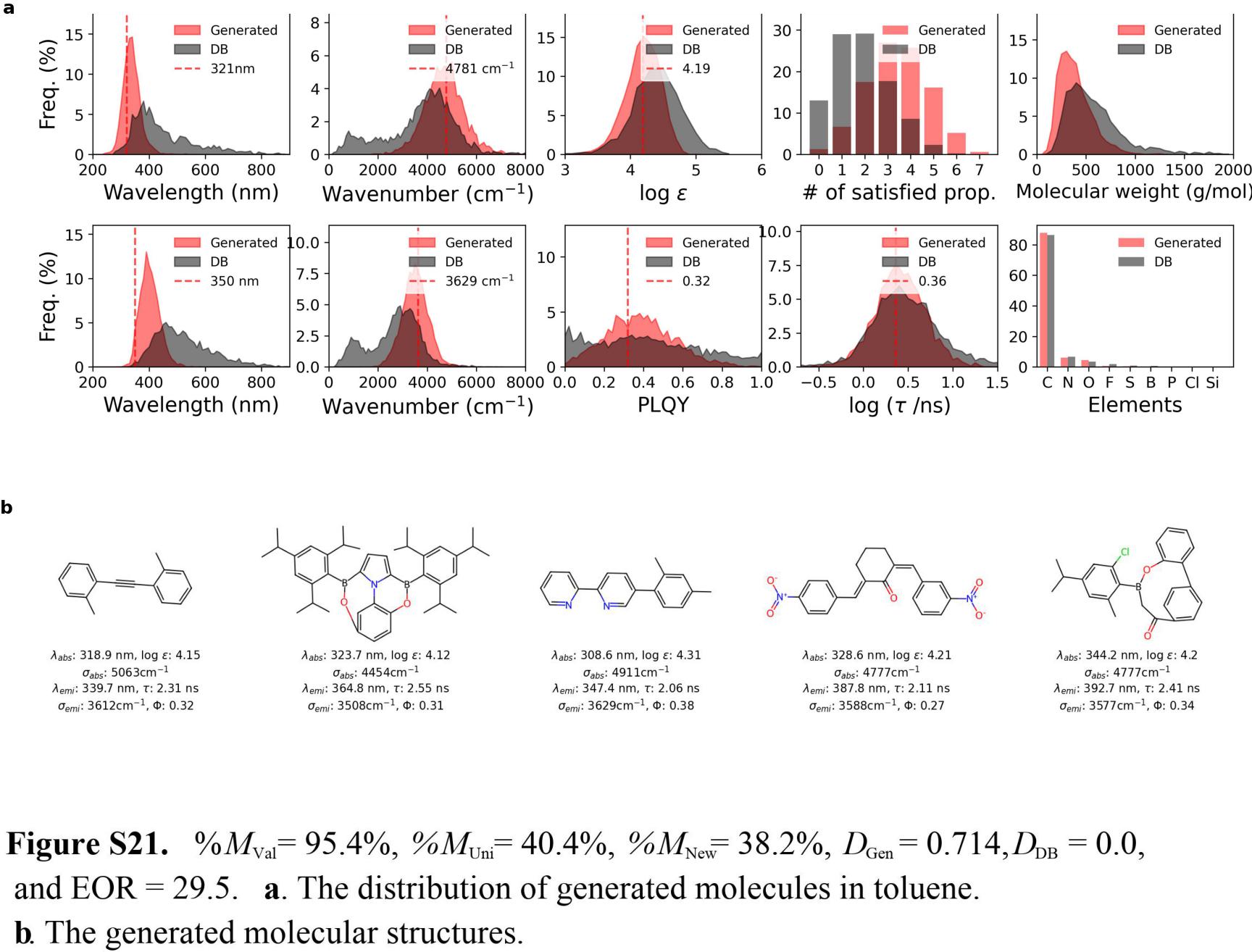

Supplement: Supplementary file 2 — oc4c00656_si_002.zip [file oc4c00656_si_002.zip › FigureS21.jpg]

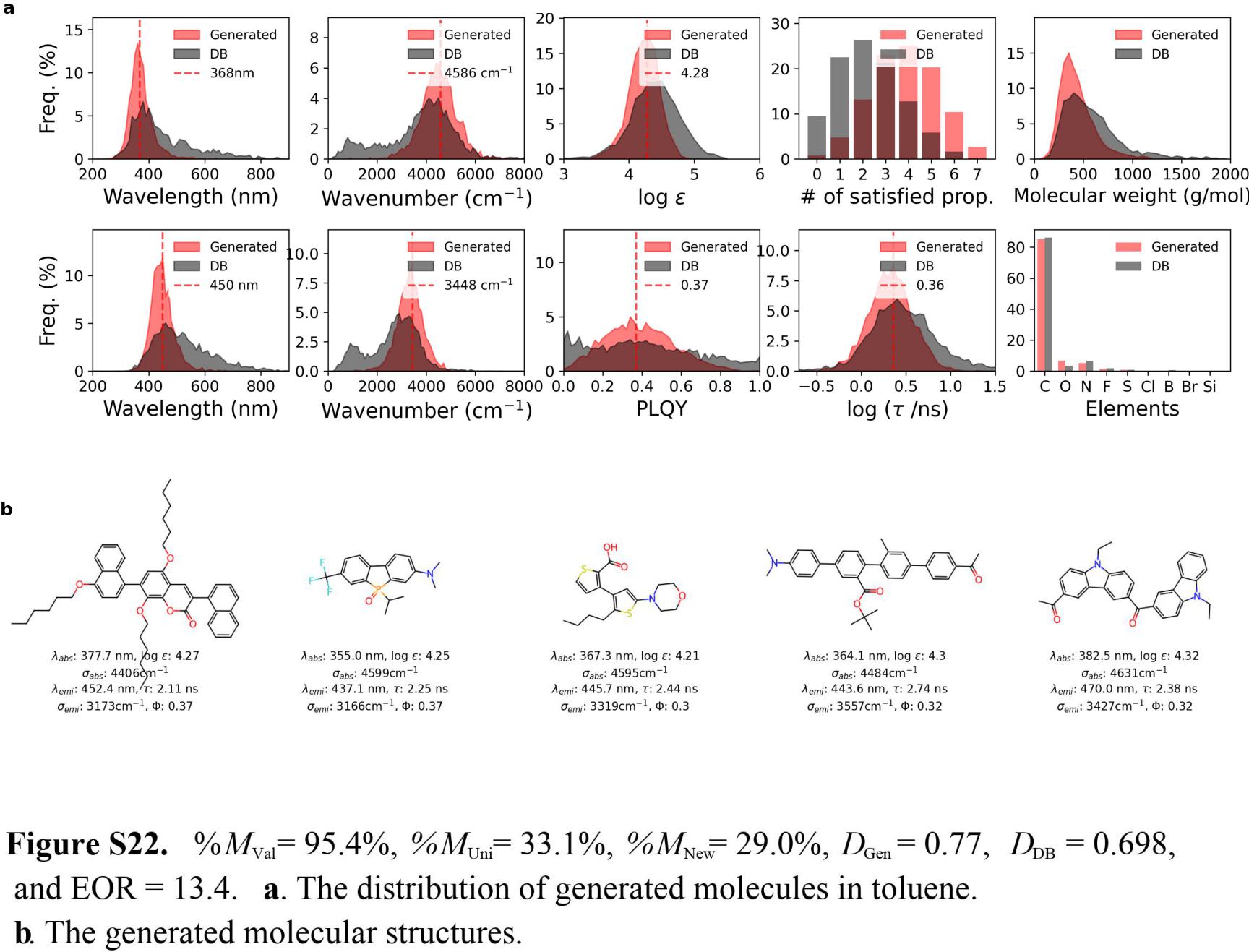

Supplement: Supplementary file 2 — oc4c00656_si_002.zip [file oc4c00656_si_002.zip › FigureS22.jpg]

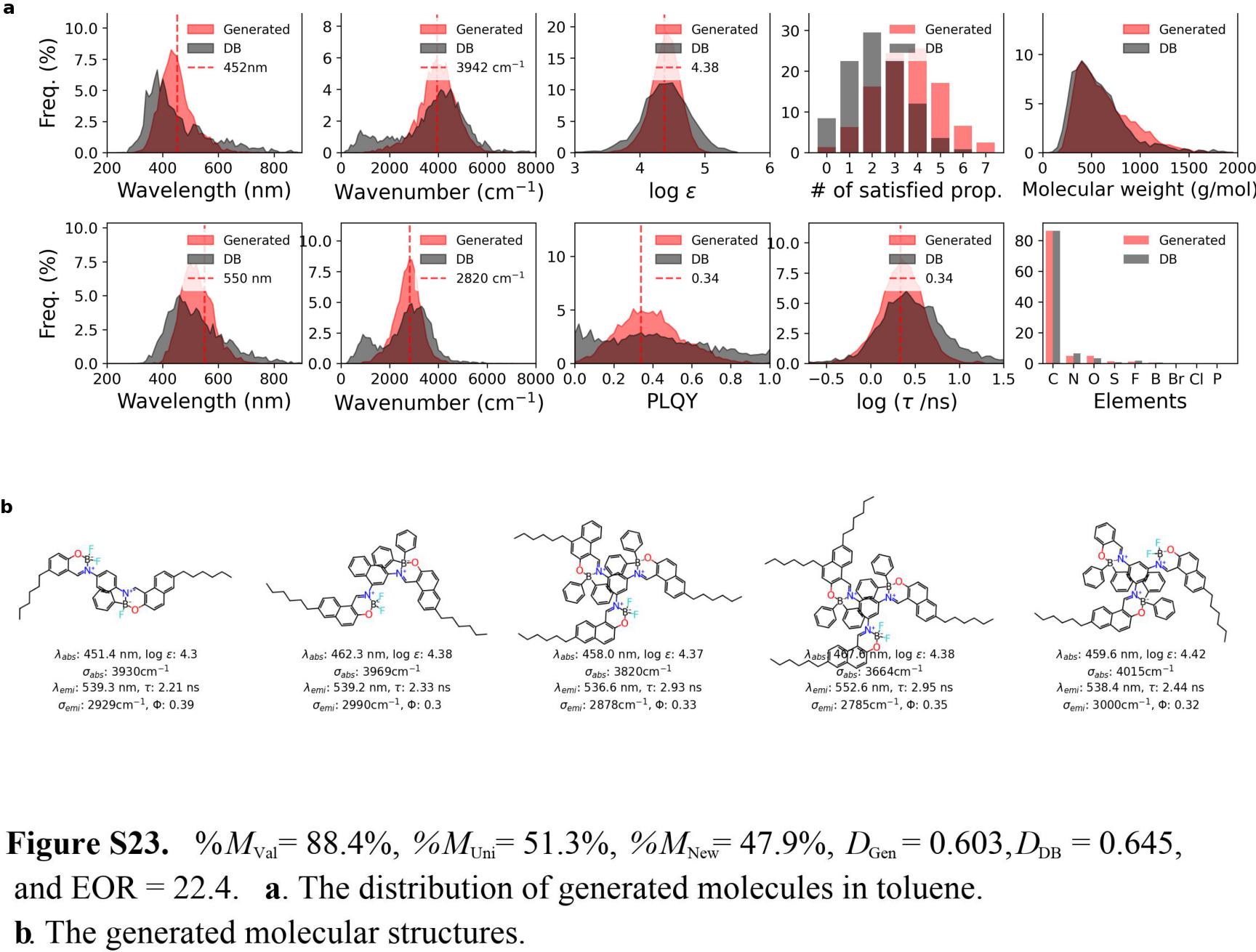

Supplement: Supplementary file 2 — oc4c00656_si_002.zip [file oc4c00656_si_002.zip › FigureS23.jpg]

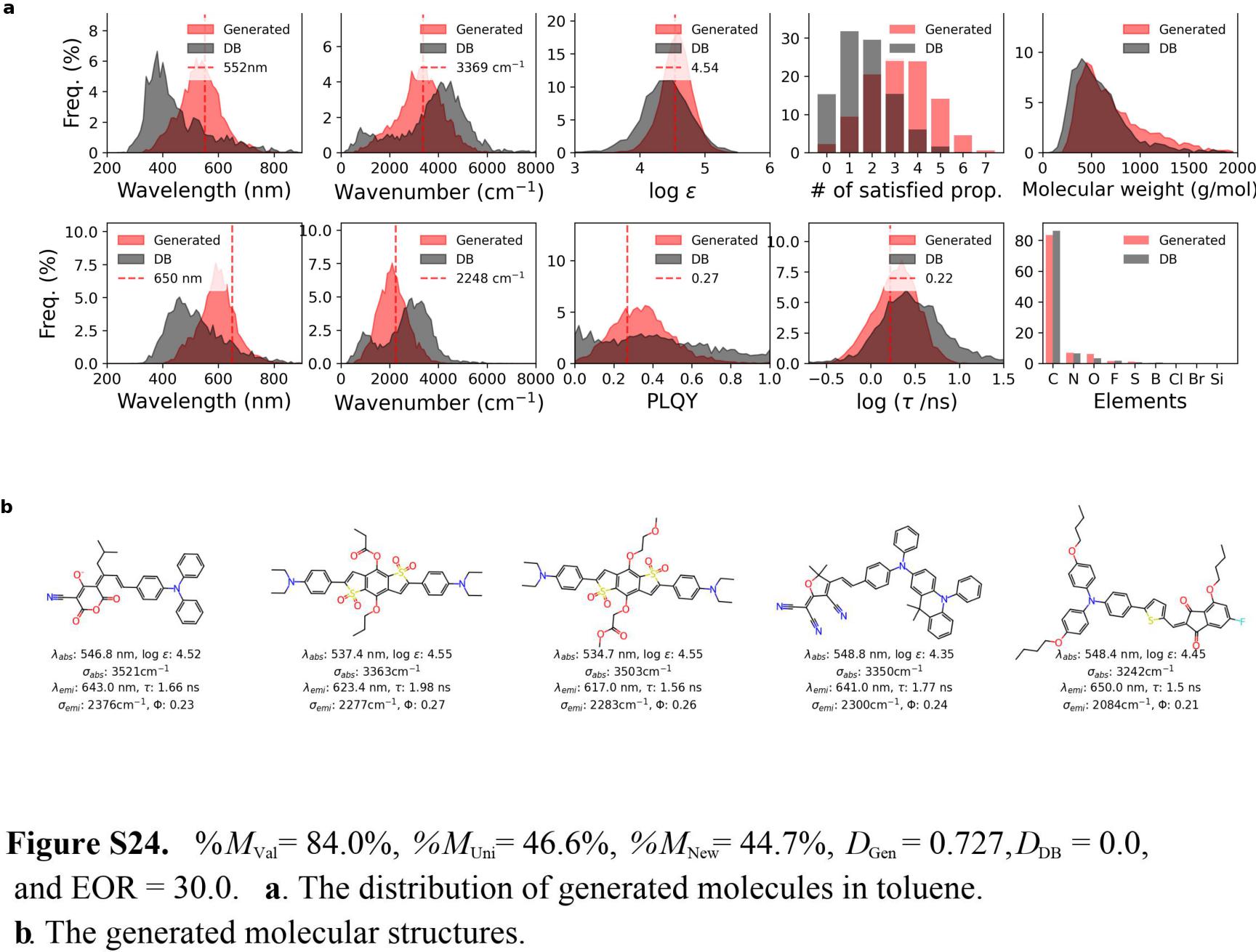

Supplement: Supplementary file 2 — oc4c00656_si_002.zip [file oc4c00656_si_002.zip › FigureS24.jpg]

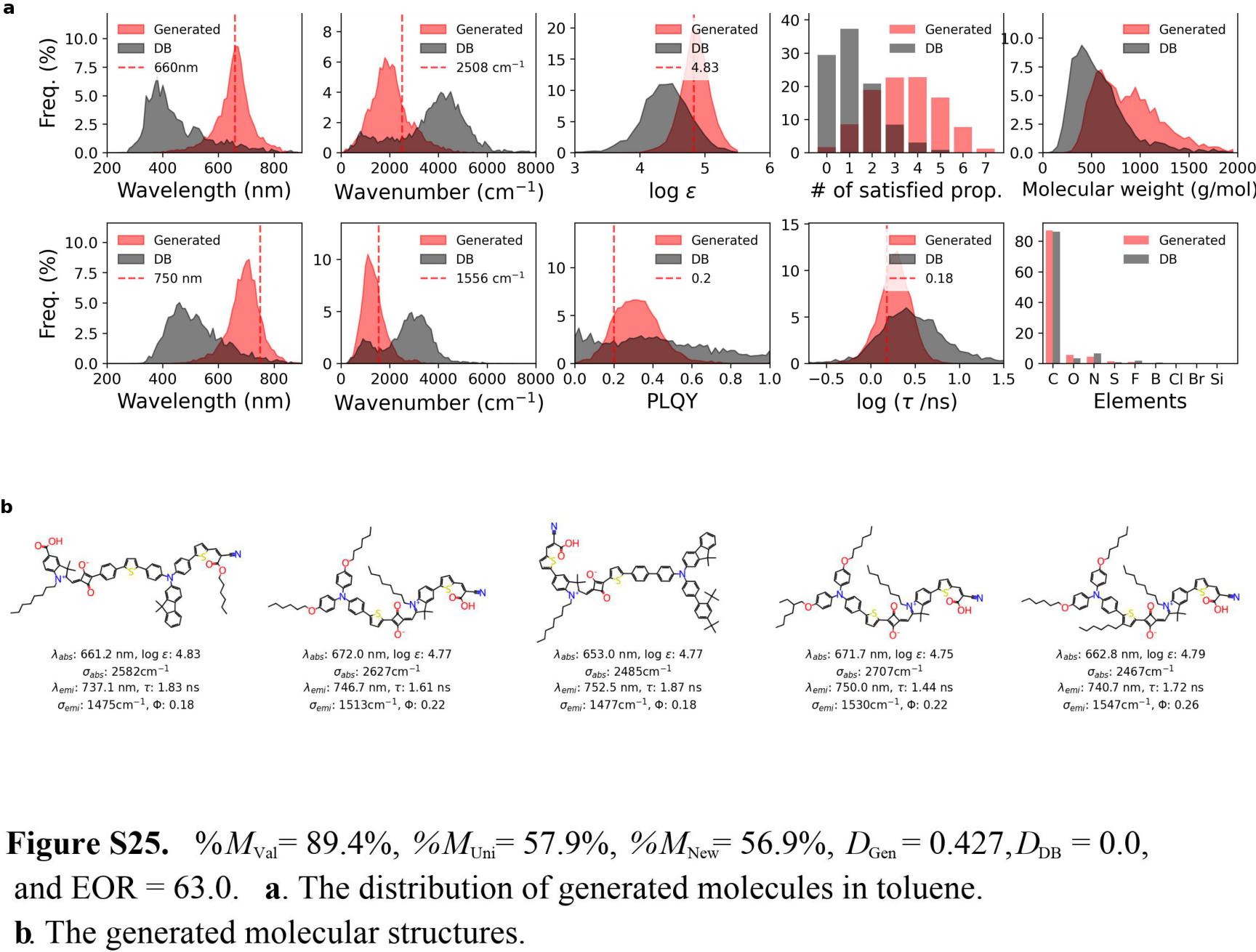

Supplement: Supplementary file 2 — oc4c00656_si_002.zip [file oc4c00656_si_002.zip › FigureS25.jpg]

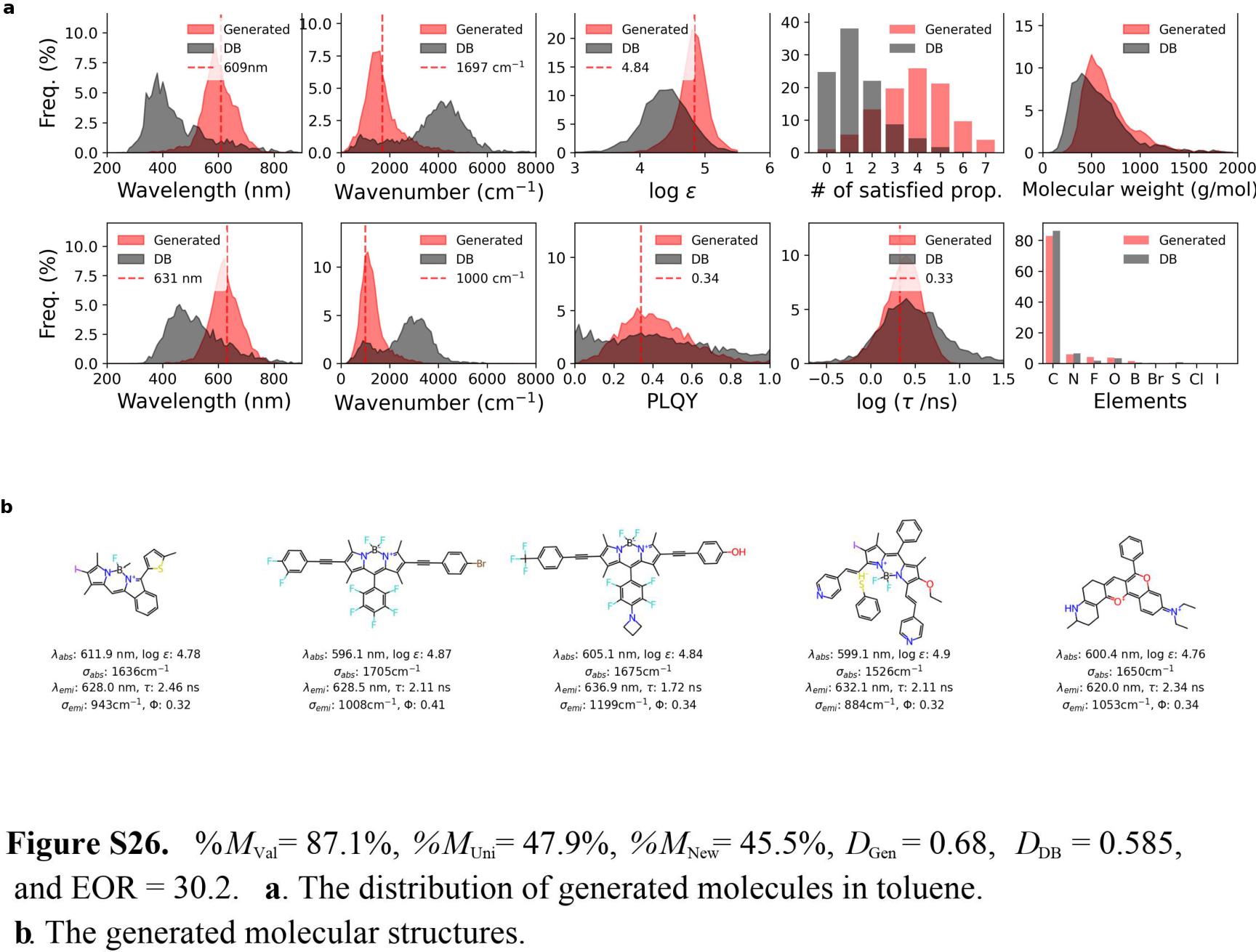

Supplement: Supplementary file 2 — oc4c00656_si_002.zip [file oc4c00656_si_002.zip › FigureS26.jpg]

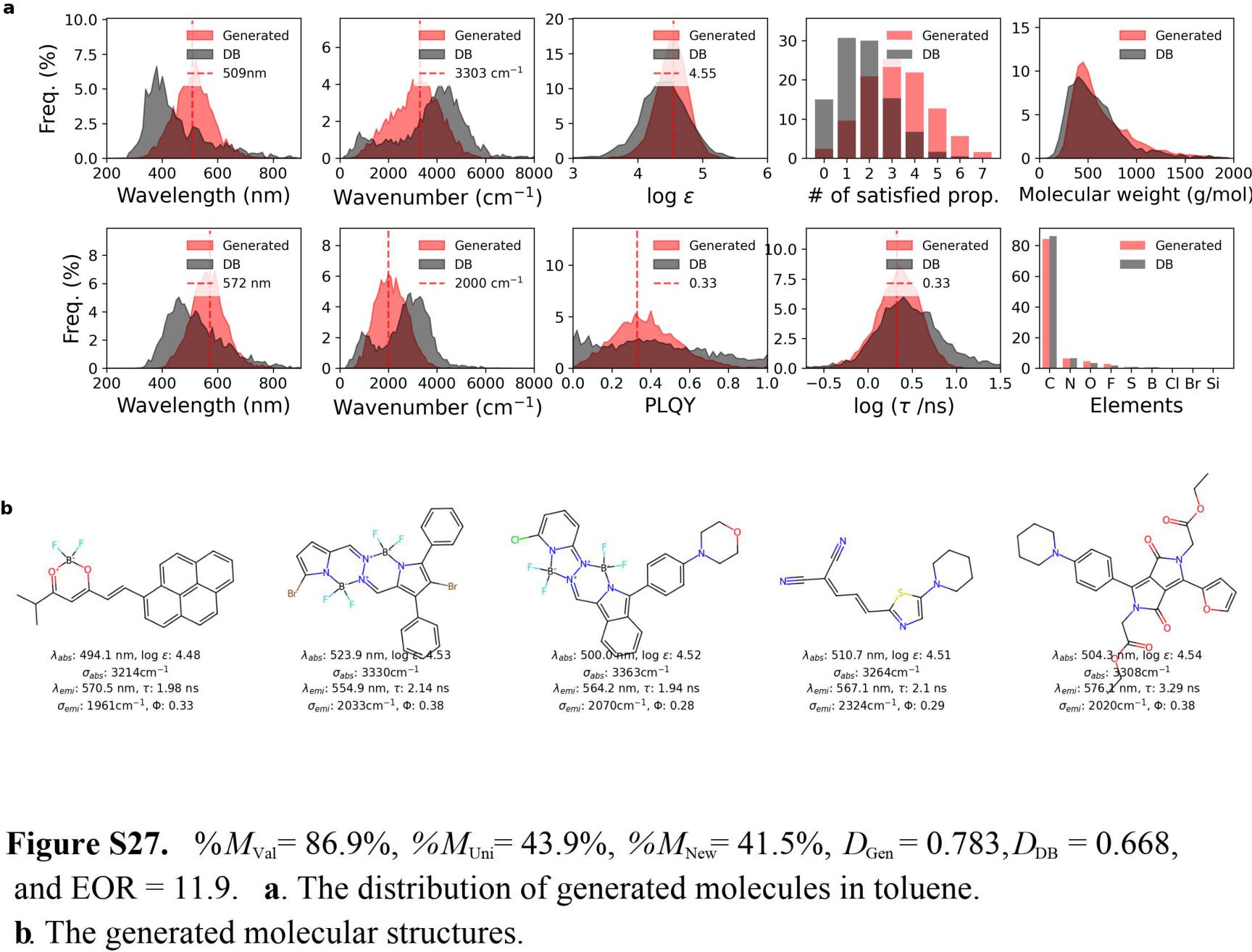

Supplement: Supplementary file 2 — oc4c00656_si_002.zip [file oc4c00656_si_002.zip › FigureS27.jpg]

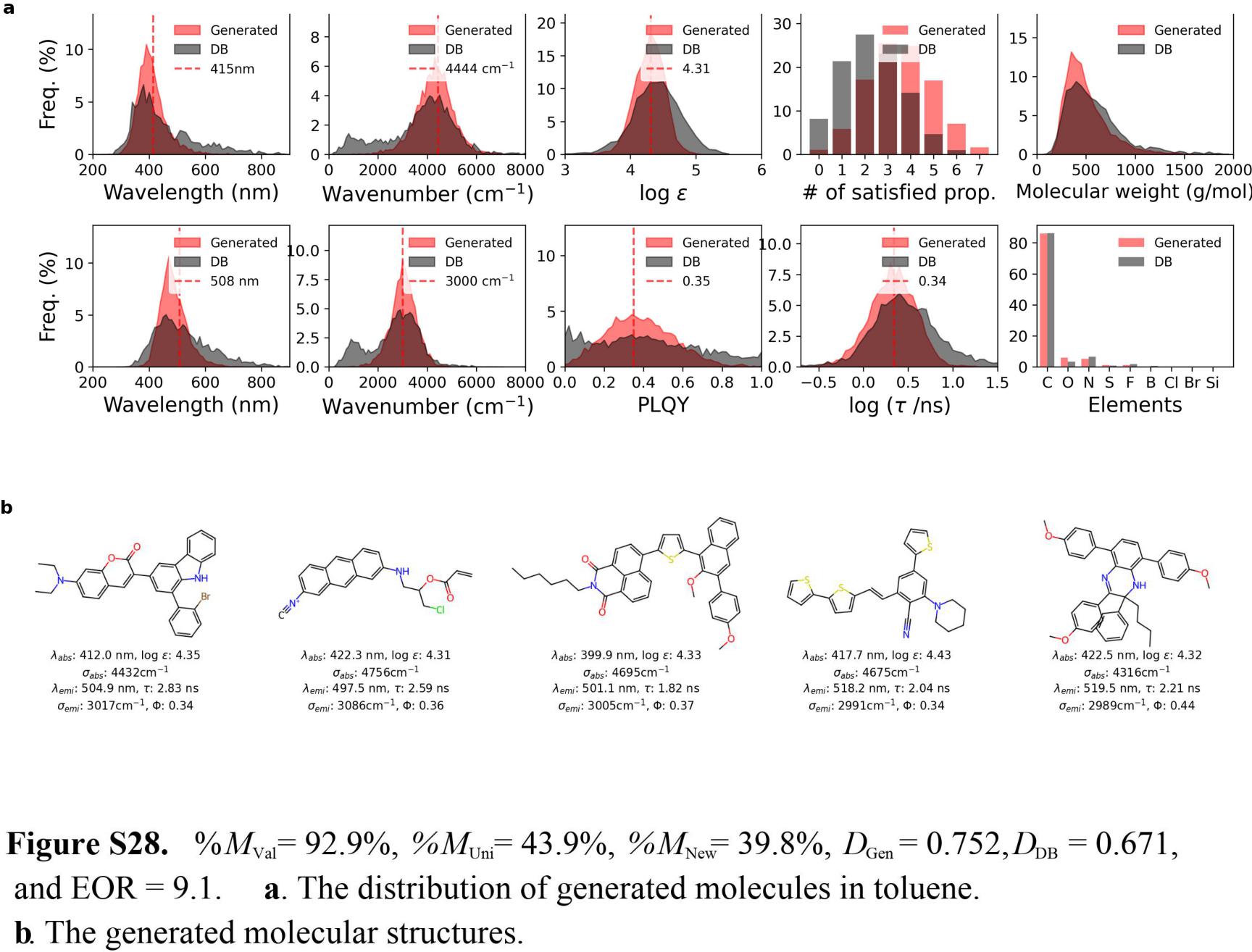

Supplement: Supplementary file 2 — oc4c00656_si_002.zip [file oc4c00656_si_002.zip › FigureS28.jpg]

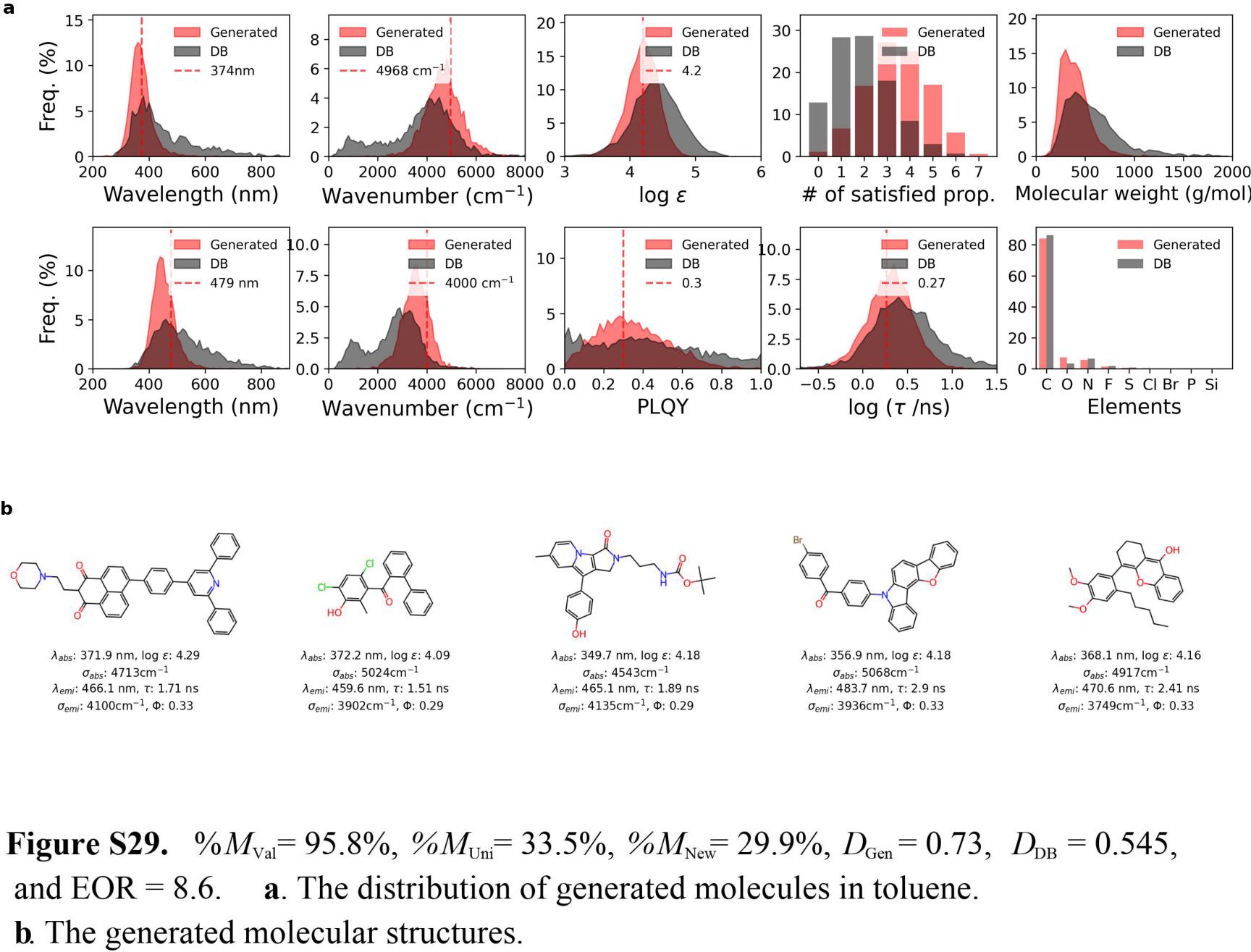

Supplement: Supplementary file 2 — oc4c00656_si_002.zip [file oc4c00656_si_002.zip › FigureS29.jpg]

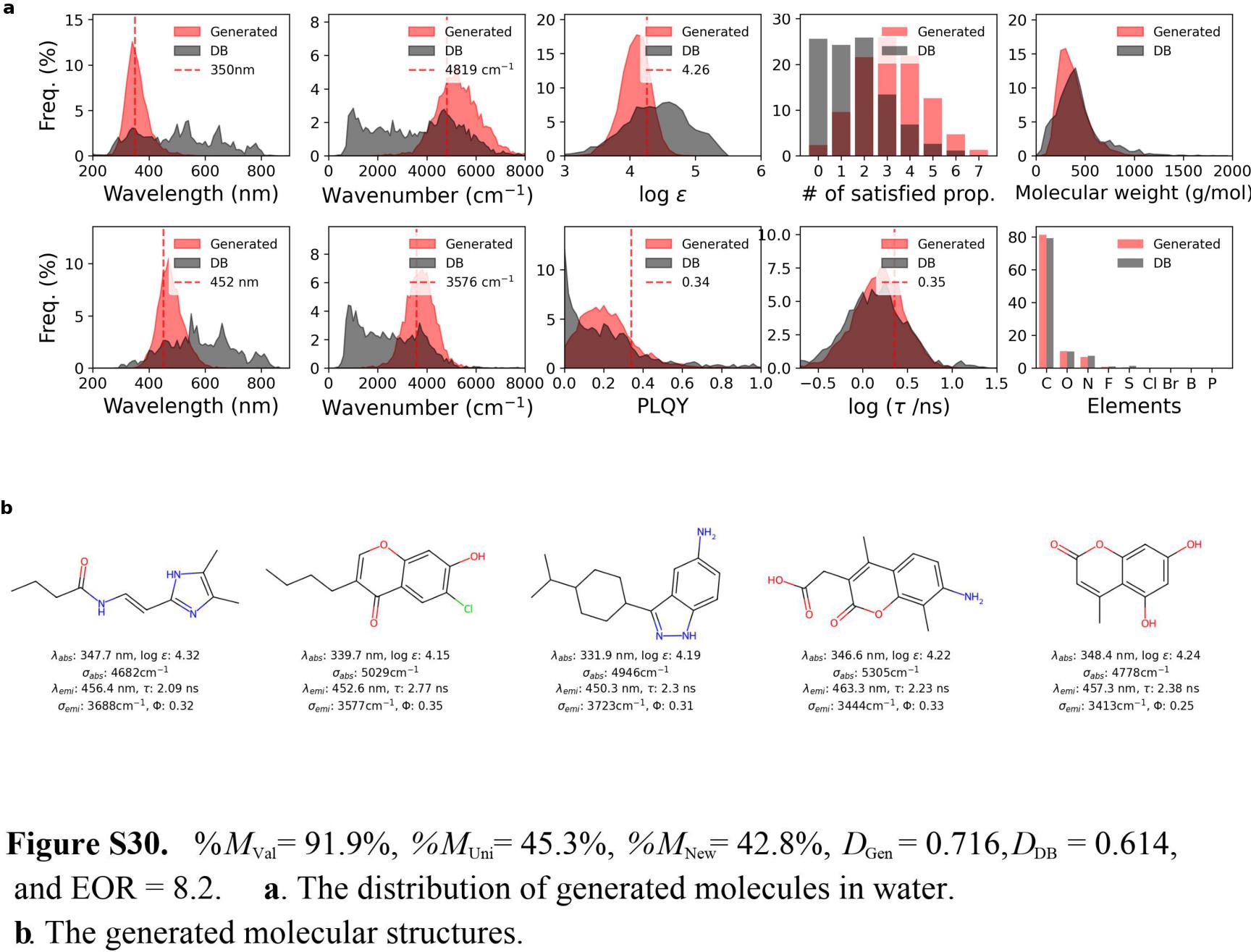

Supplement: Supplementary file 2 — oc4c00656_si_002.zip [file oc4c00656_si_002.zip › FigureS30.jpg]

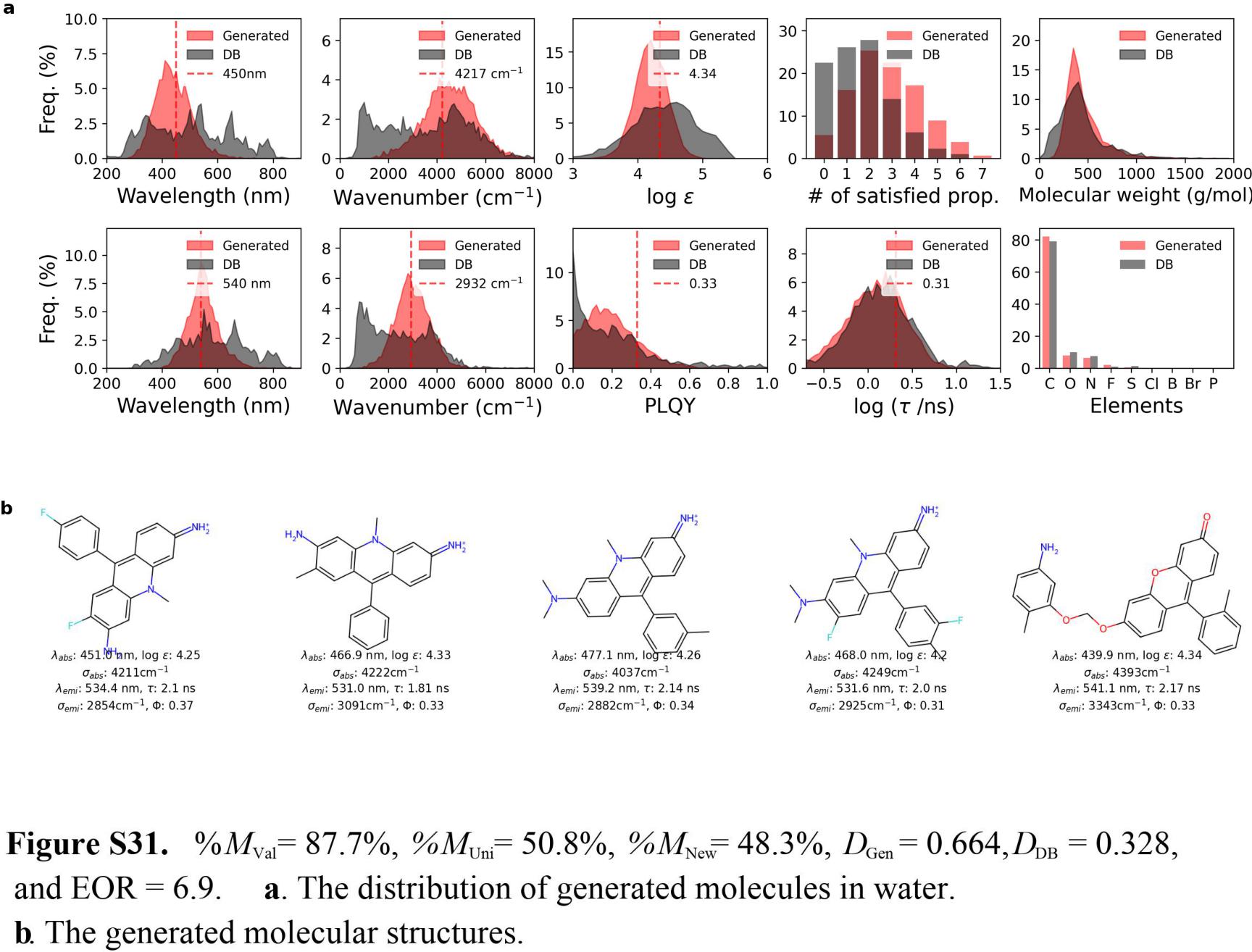

Supplement: Supplementary file 2 — oc4c00656_si_002.zip [file oc4c00656_si_002.zip › FigureS31.jpg]

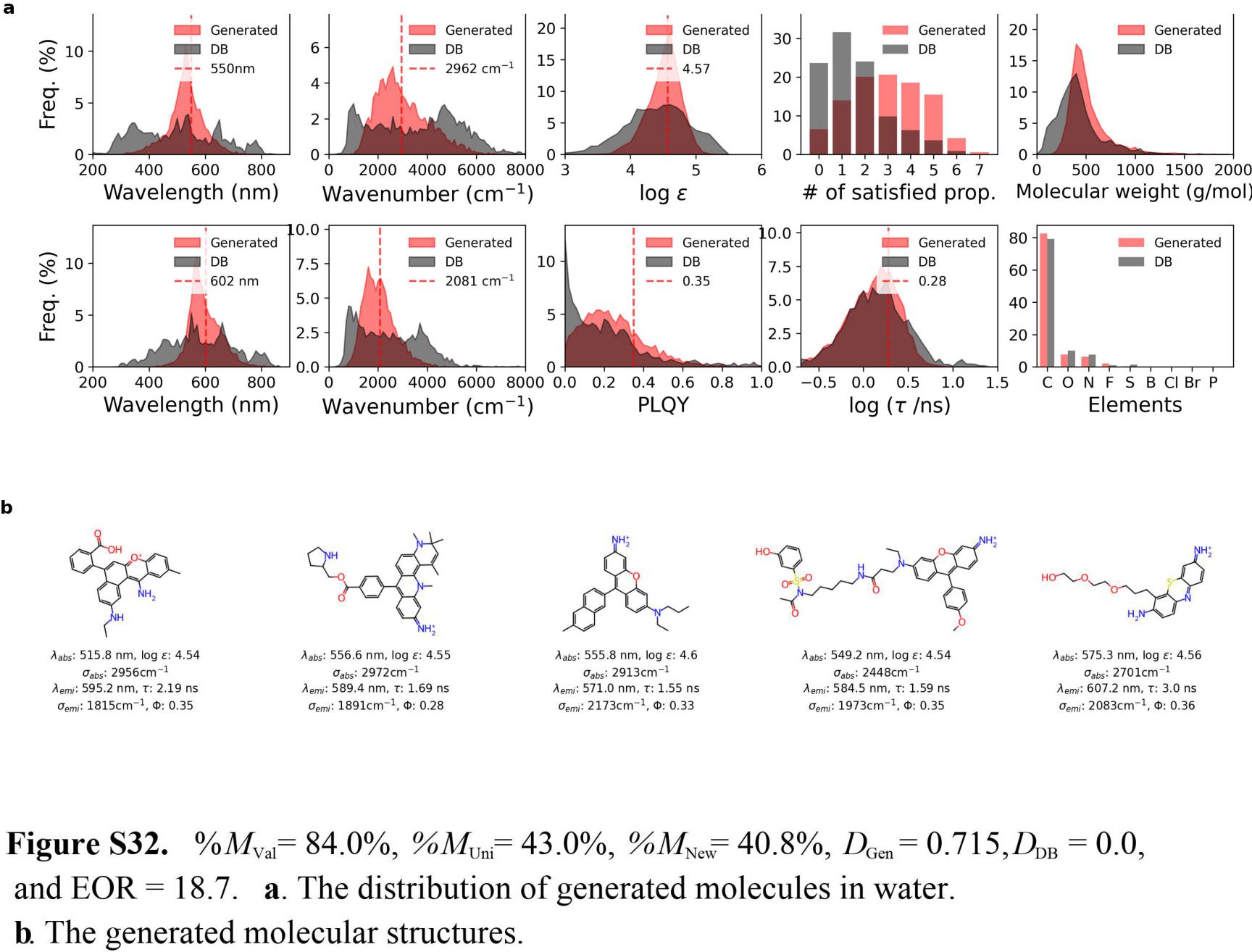

Supplement: Supplementary file 2 — oc4c00656_si_002.zip [file oc4c00656_si_002.zip › FigureS32.jpg]

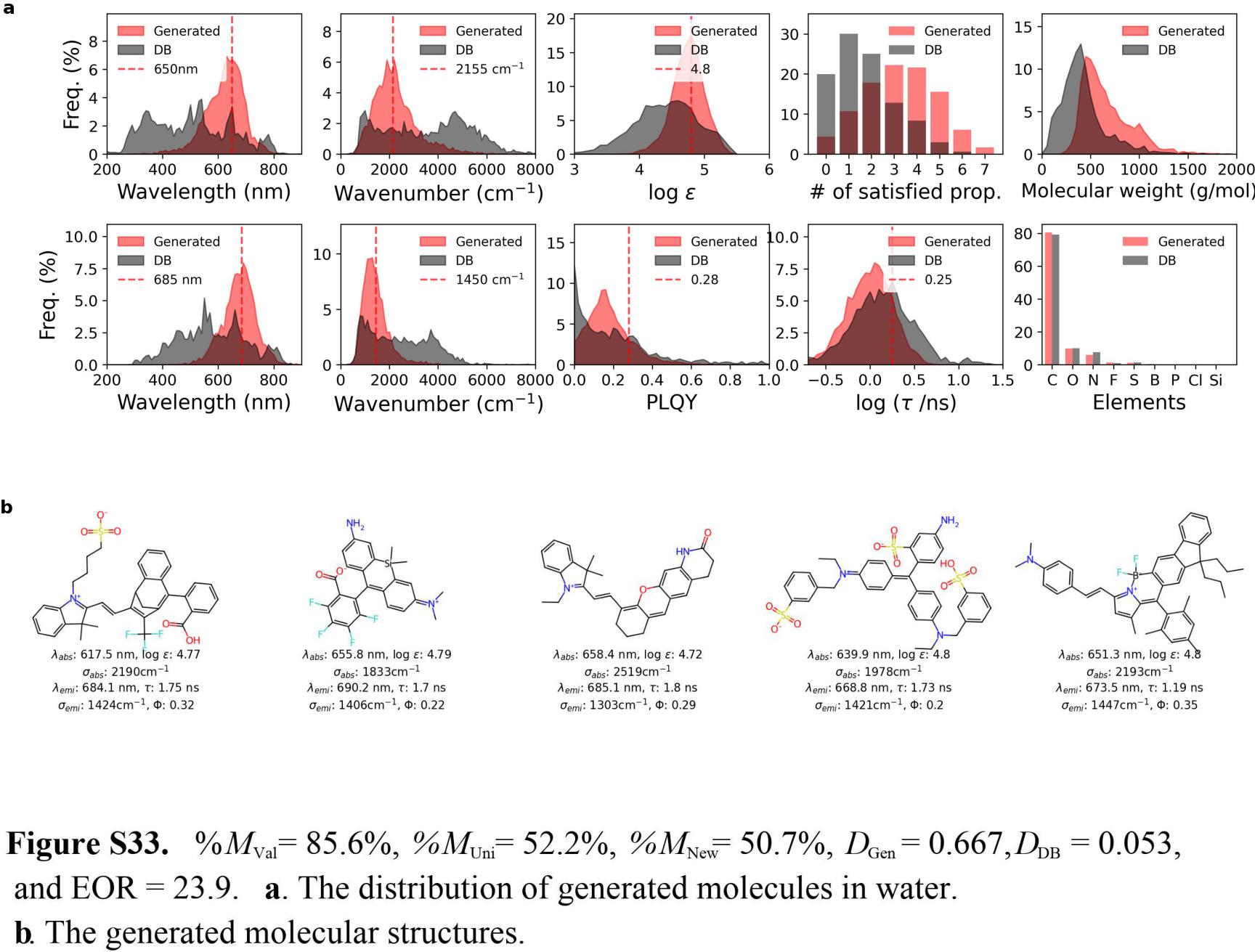

Supplement: Supplementary file 2 — oc4c00656_si_002.zip [file oc4c00656_si_002.zip › FigureS33.jpg]

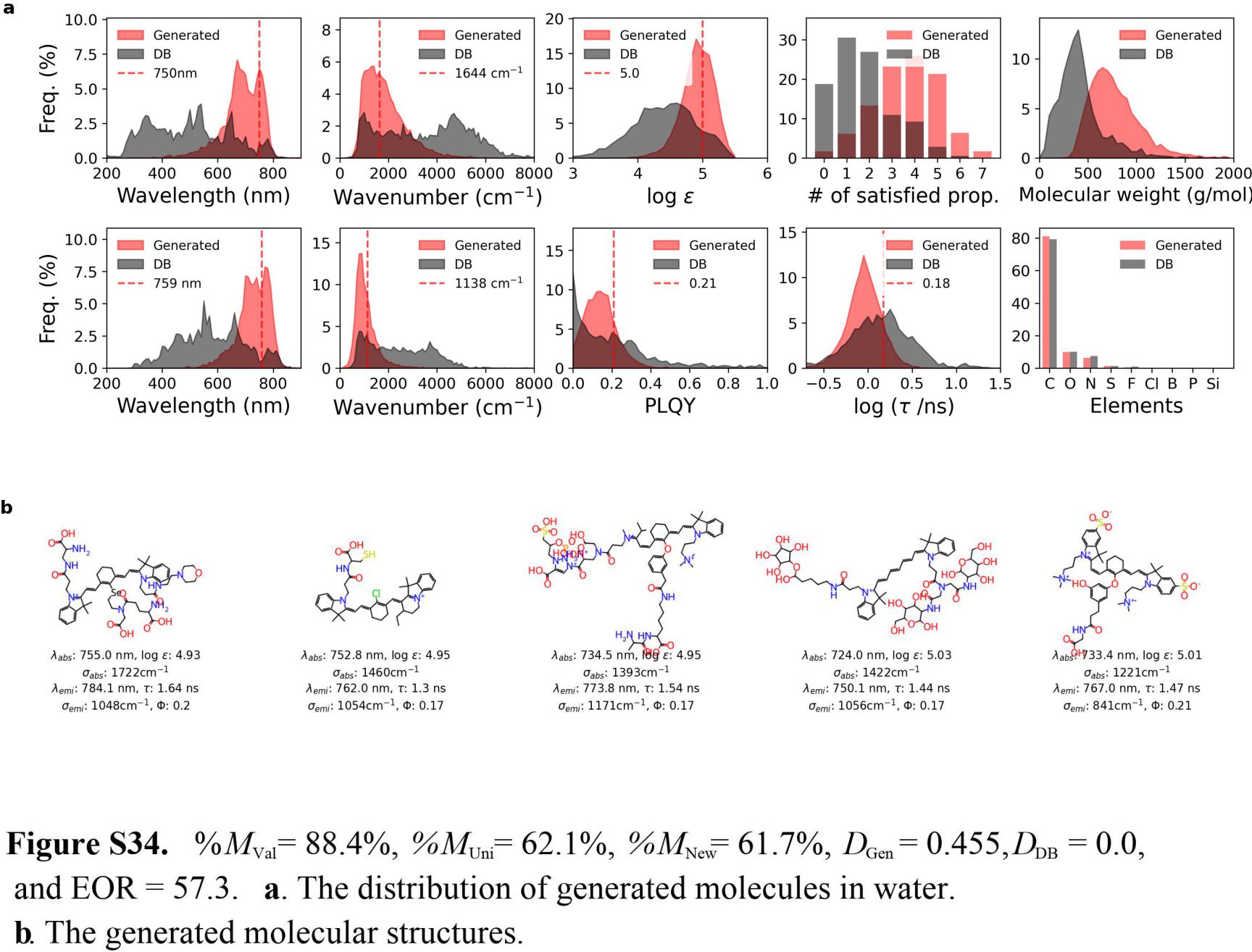

Supplement: Supplementary file 2 — oc4c00656_si_002.zip [file oc4c00656_si_002.zip › FigureS34.jpg]

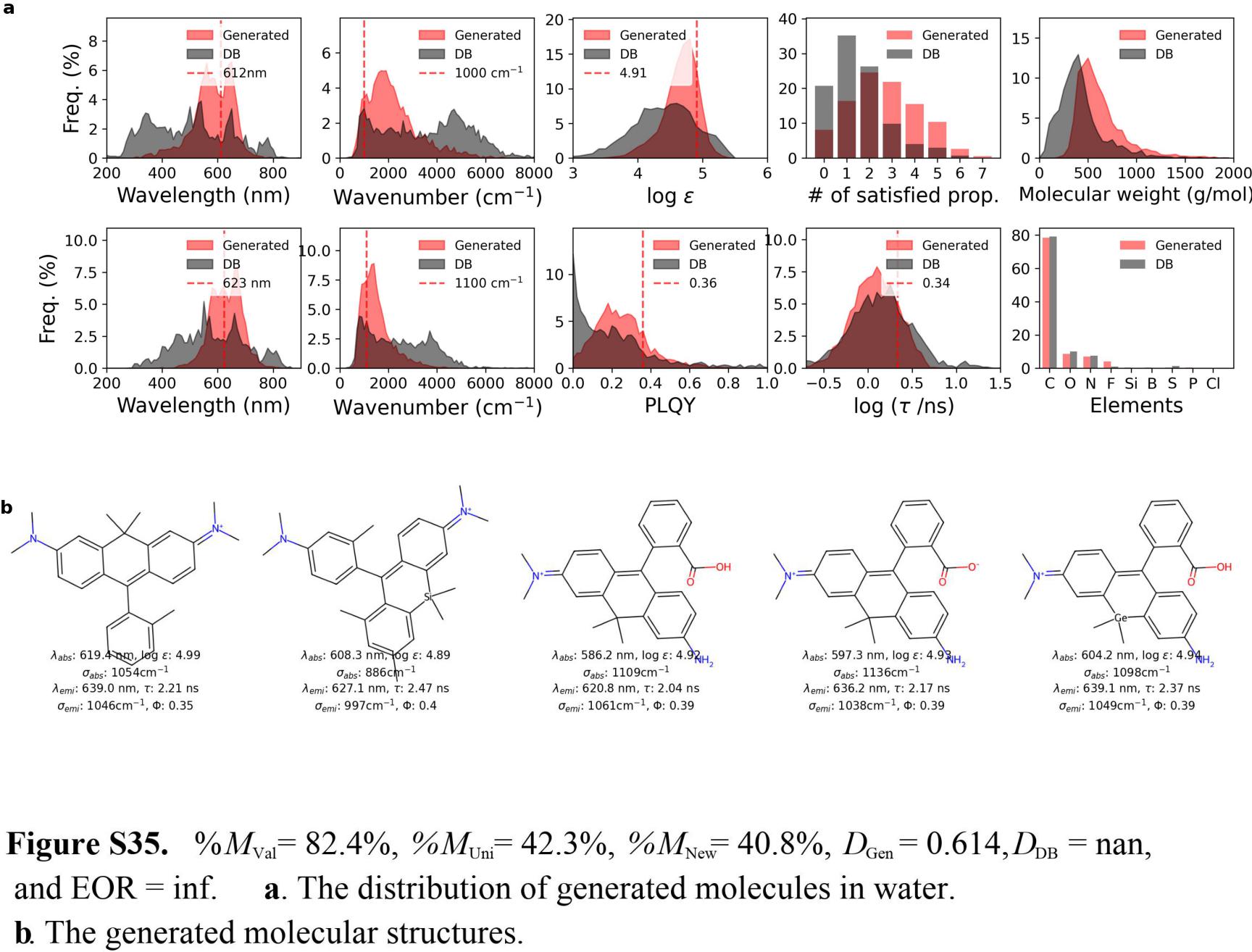

Supplement: Supplementary file 2 — oc4c00656_si_002.zip [file oc4c00656_si_002.zip › FigureS35.jpg]

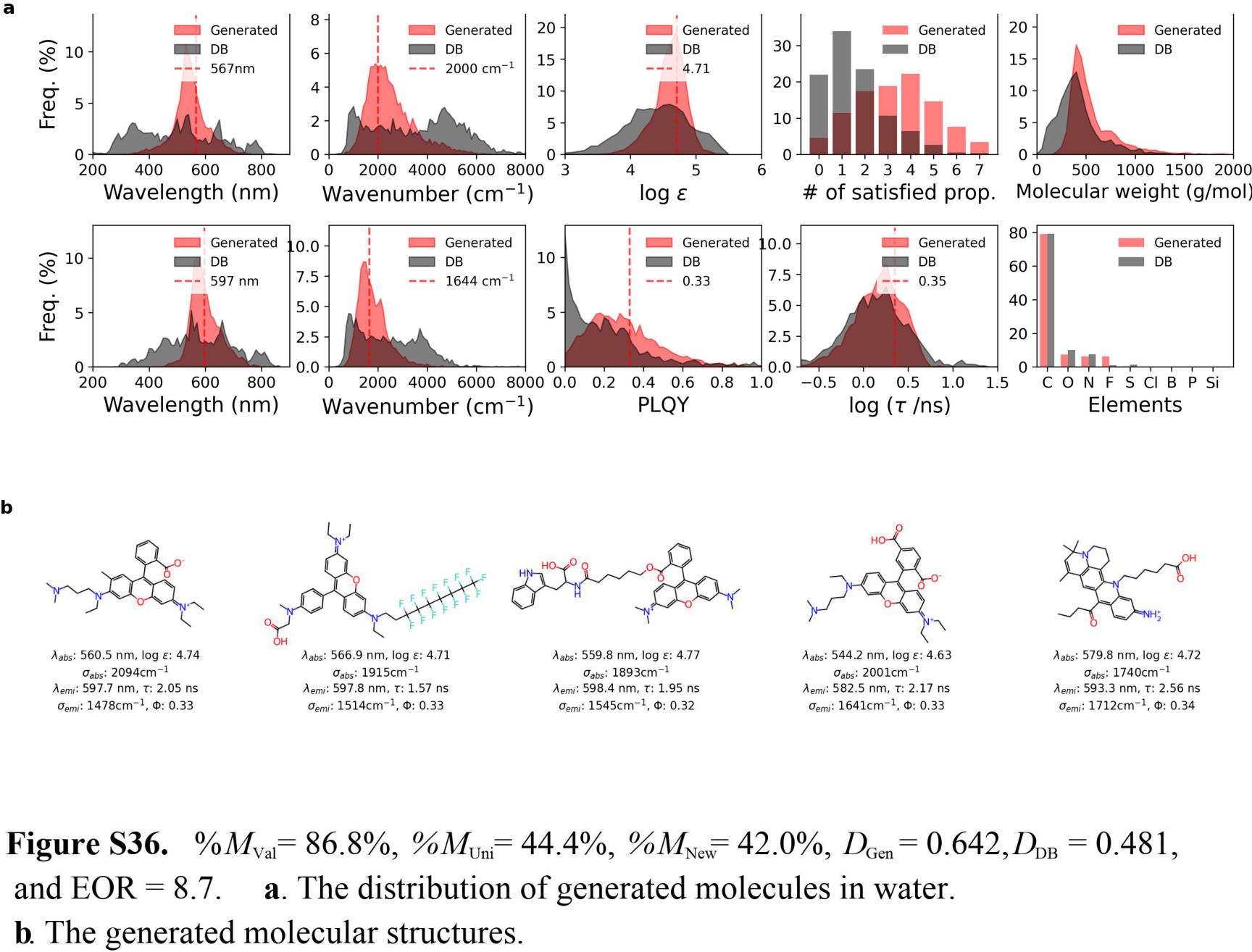

Supplement: Supplementary file 2 — oc4c00656_si_002.zip [file oc4c00656_si_002.zip › FigureS36.jpg]

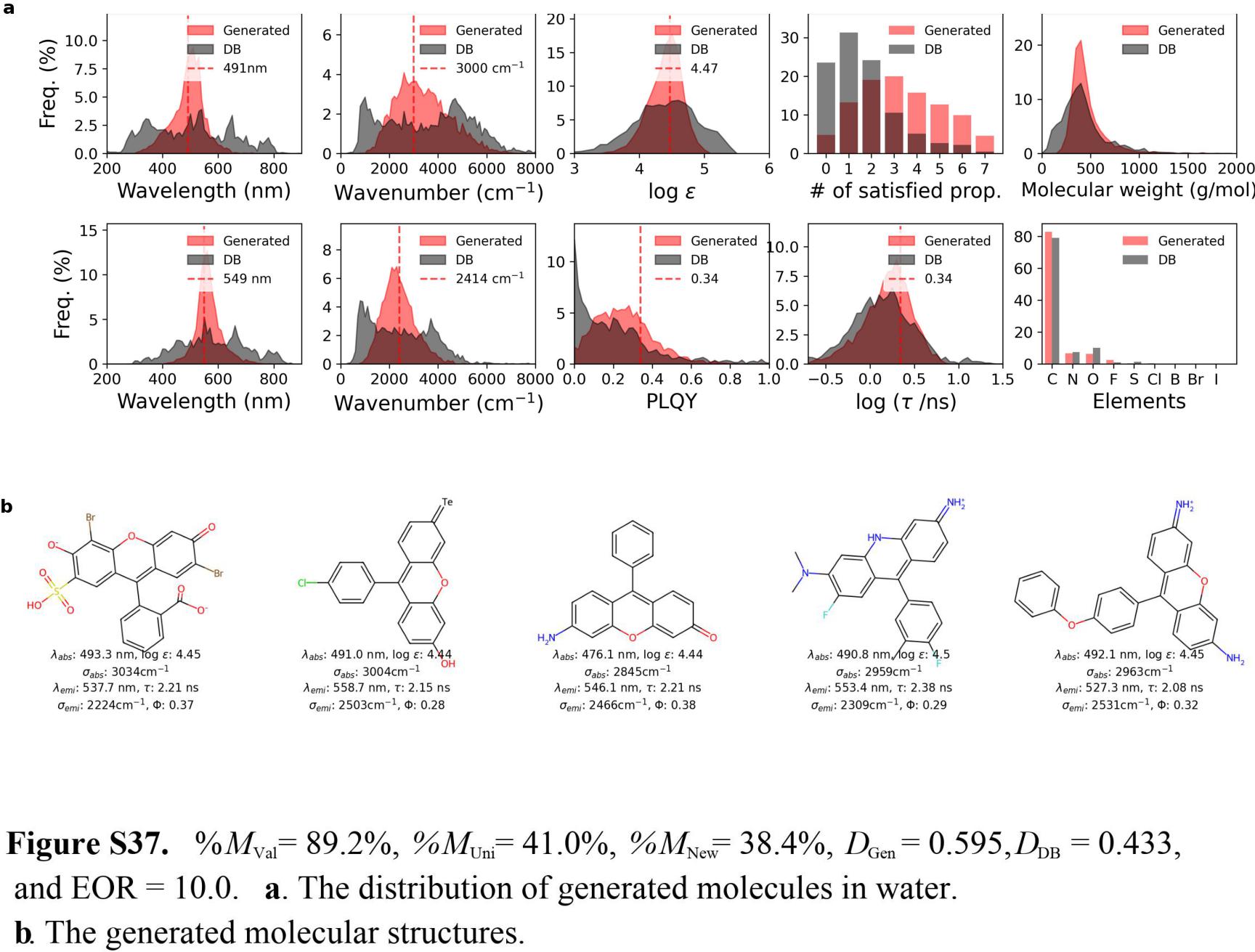

Supplement: Supplementary file 2 — oc4c00656_si_002.zip [file oc4c00656_si_002.zip › FigureS37.jpg]

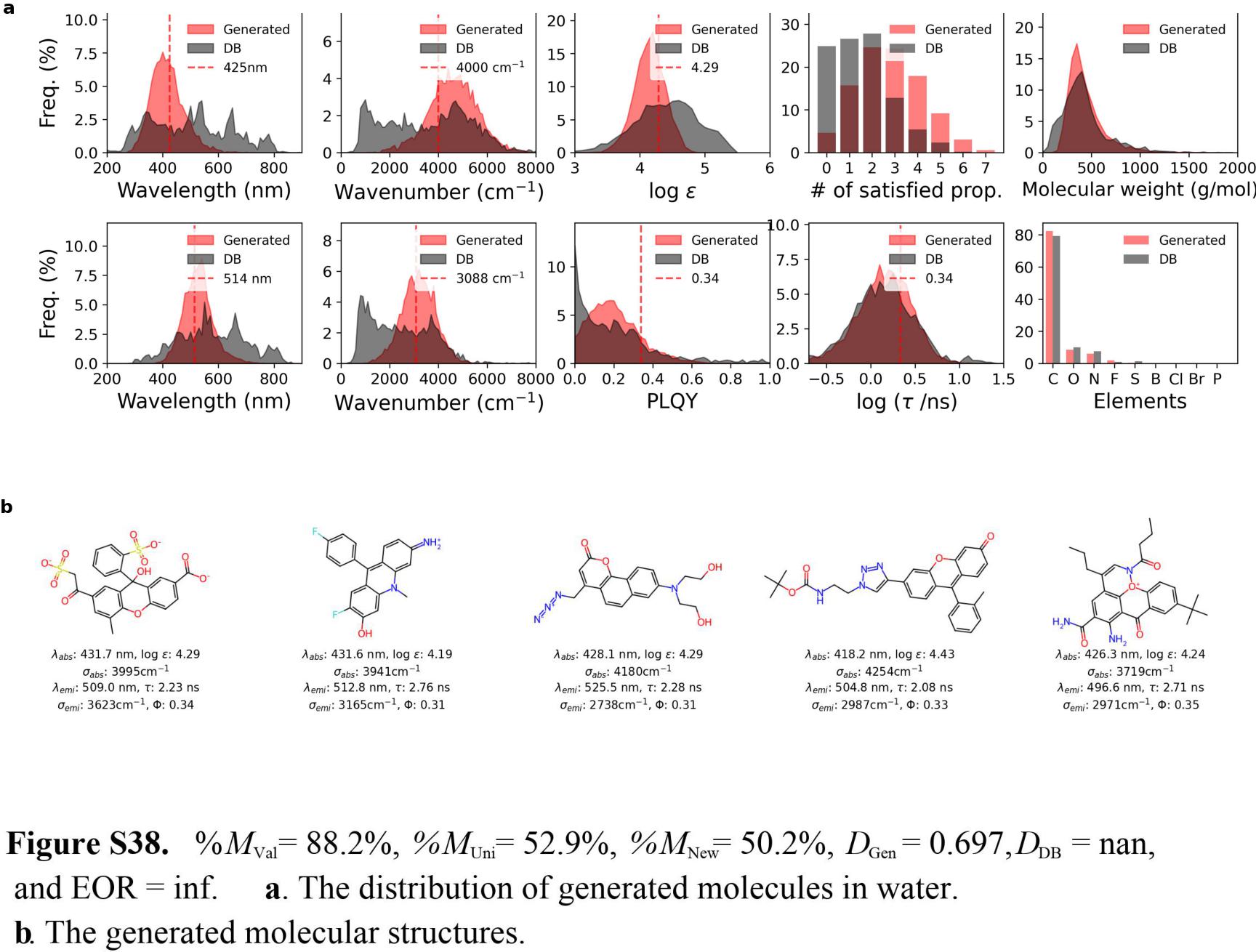

Supplement: Supplementary file 2 — oc4c00656_si_002.zip [file oc4c00656_si_002.zip › FigureS38.jpg]

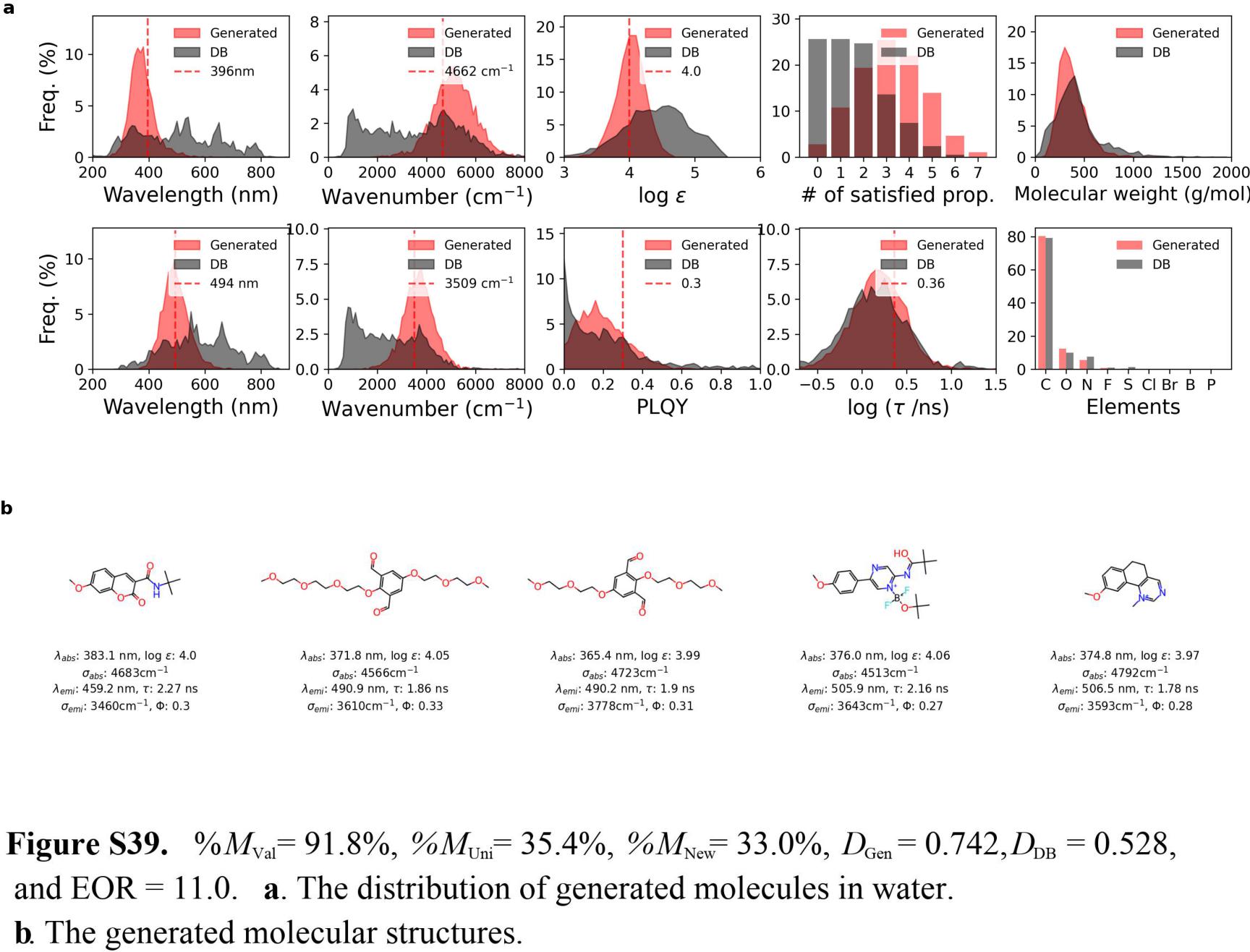

Supplement: Supplementary file 2 — oc4c00656_si_002.zip [file oc4c00656_si_002.zip › FigureS39.jpg]

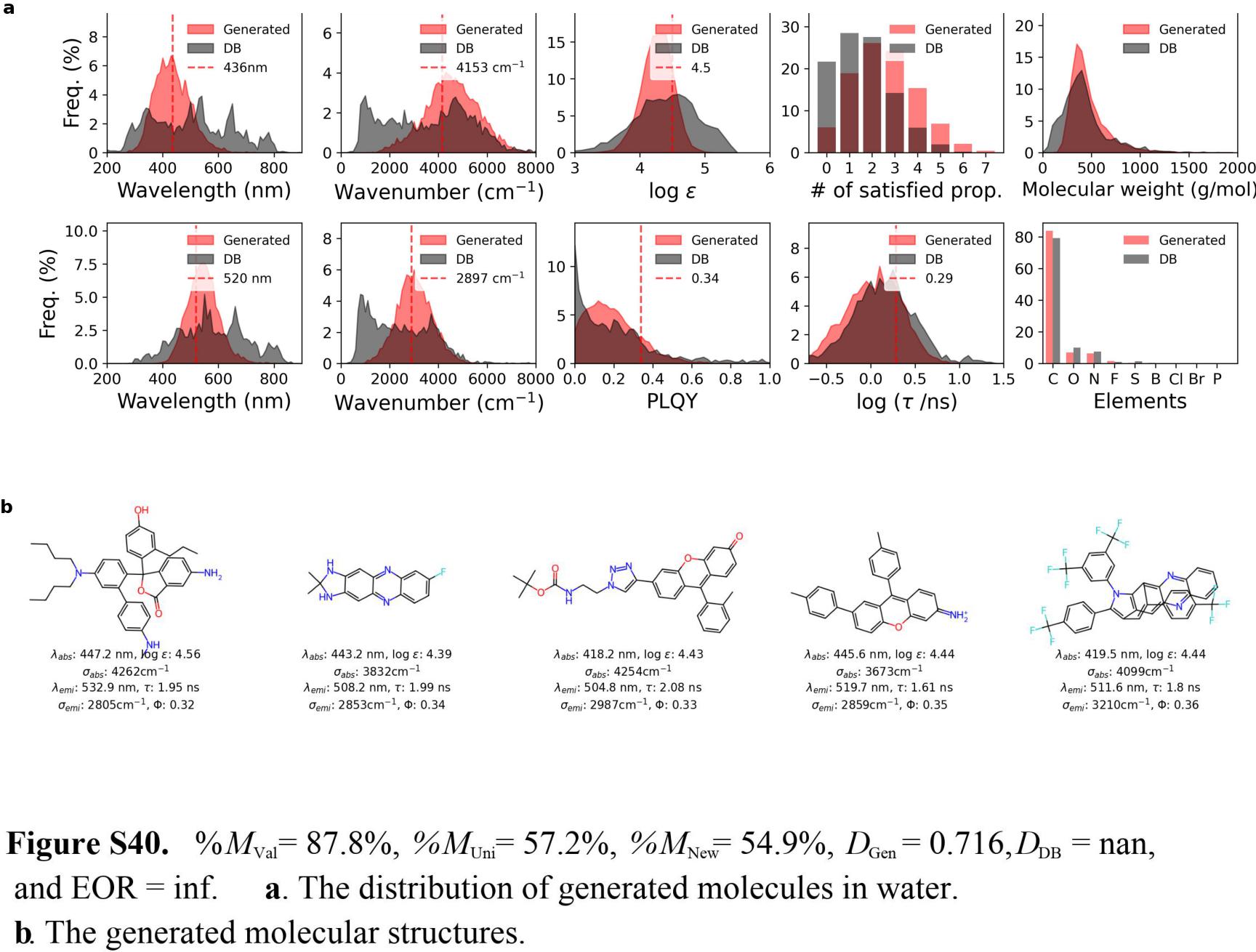

Supplement: Supplementary file 2 — oc4c00656_si_002.zip [file oc4c00656_si_002.zip › FigureS40.jpg]

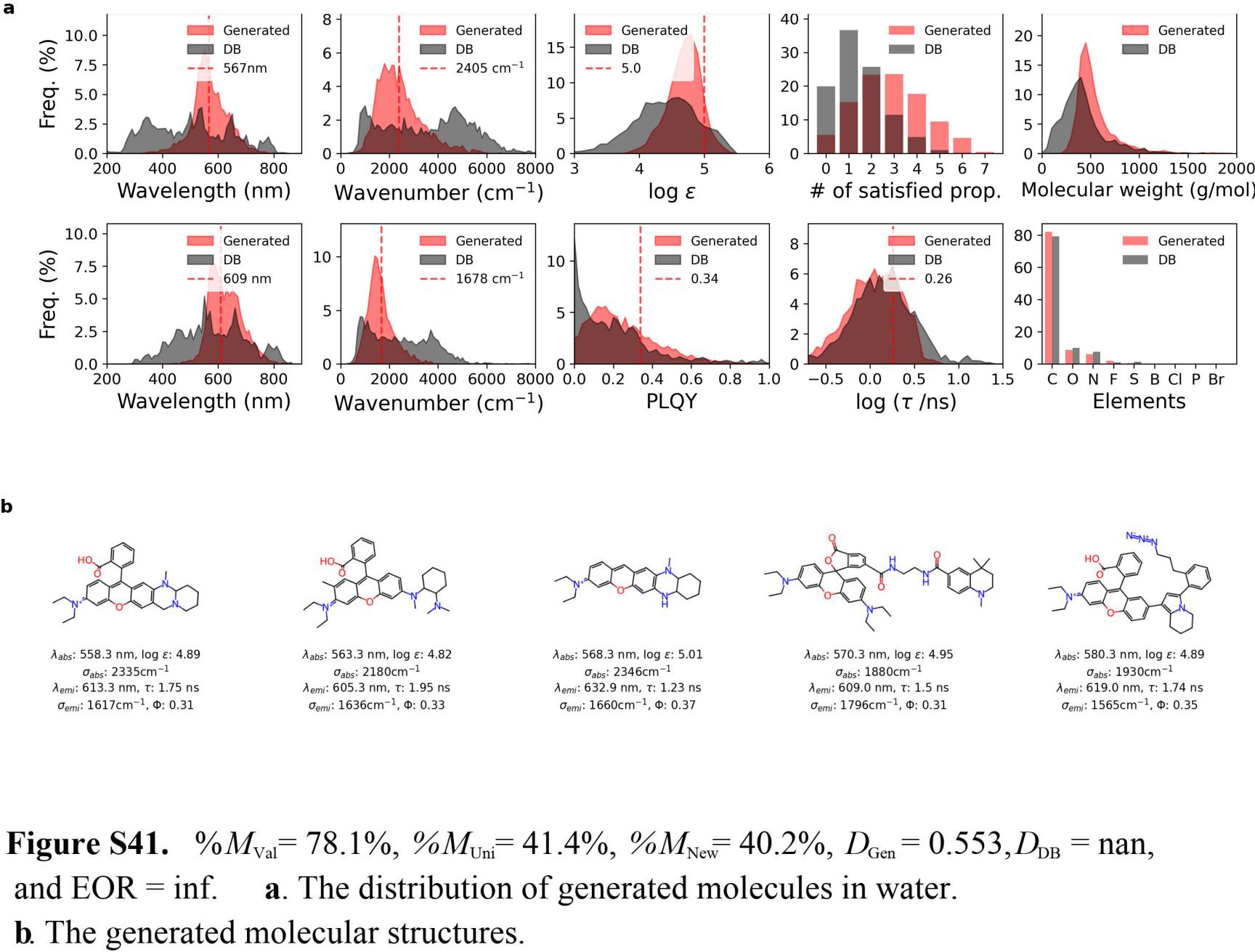

Supplement: Supplementary file 2 — oc4c00656_si_002.zip [file oc4c00656_si_002.zip › FigureS41.jpg]

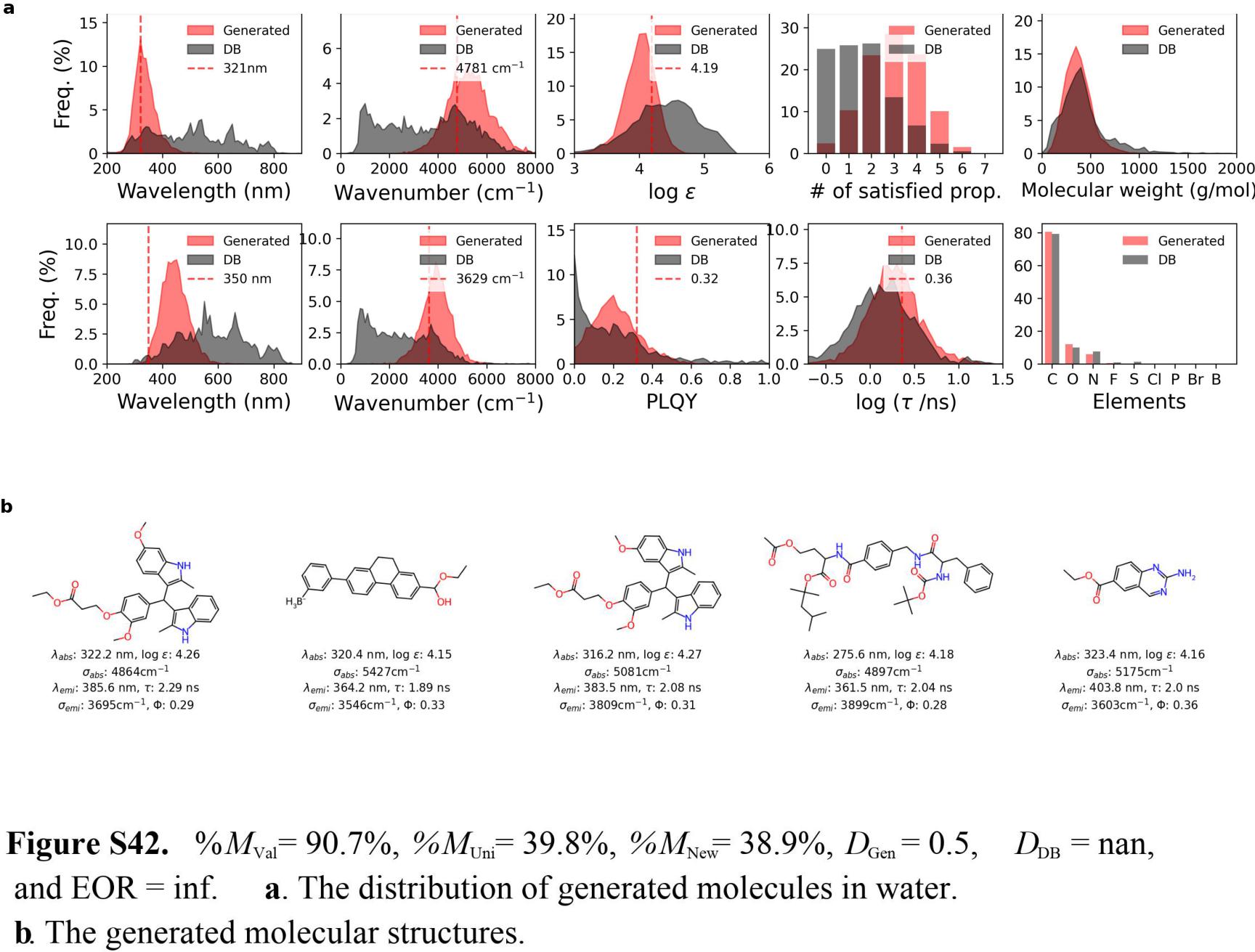

Supplement: Supplementary file 2 — oc4c00656_si_002.zip [file oc4c00656_si_002.zip › FigureS42.jpg]

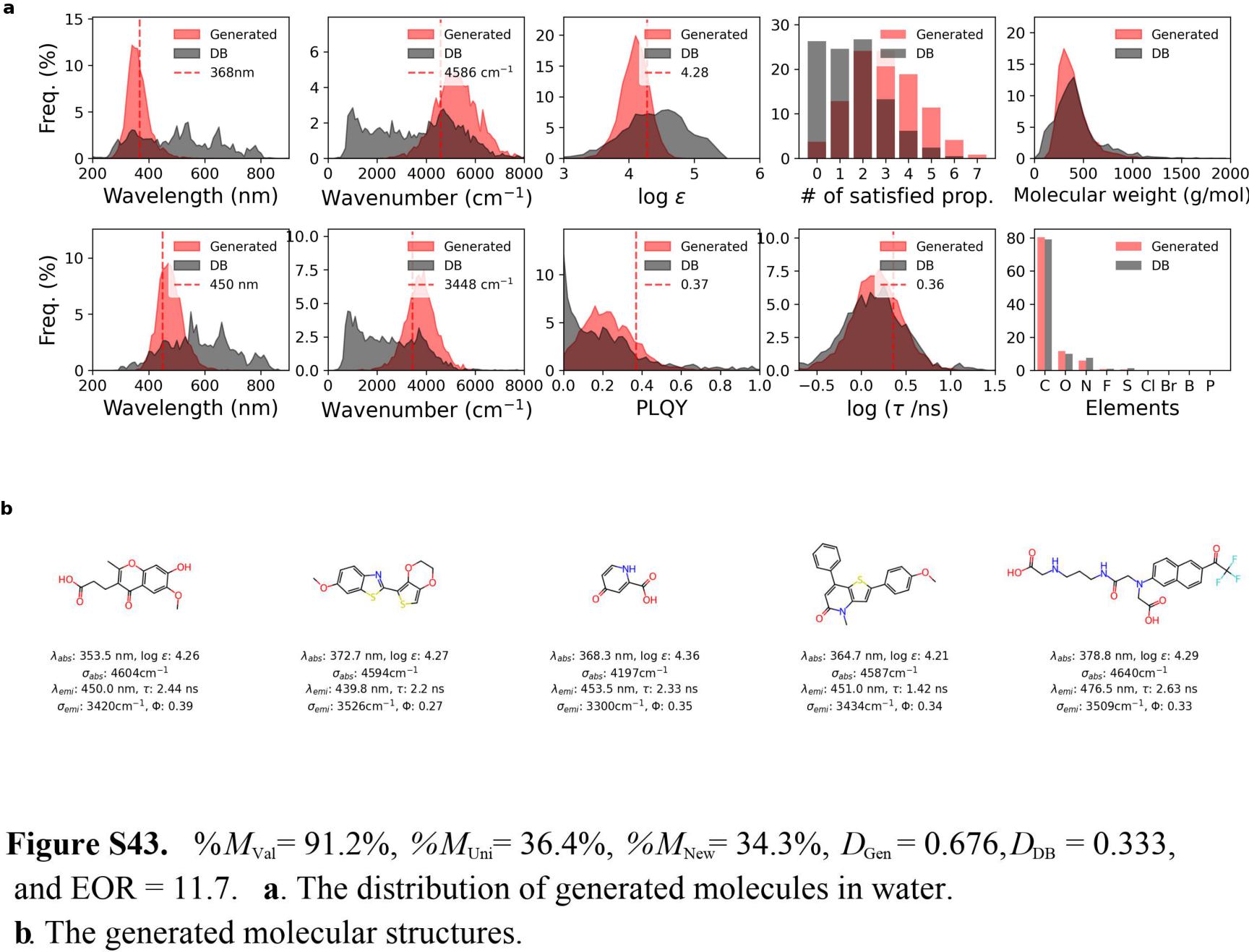

Supplement: Supplementary file 2 — oc4c00656_si_002.zip [file oc4c00656_si_002.zip › FigureS43.jpg]

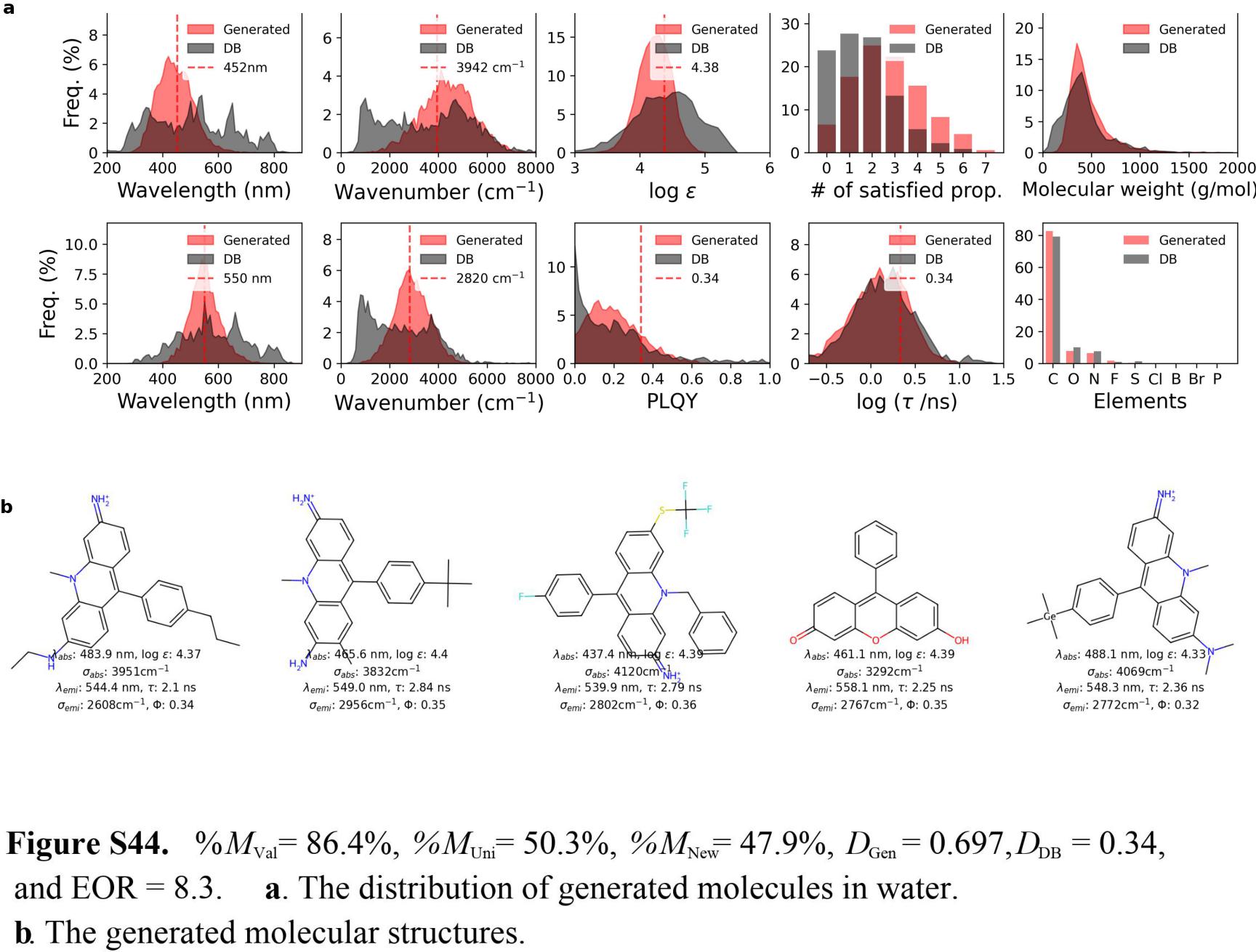

Supplement: Supplementary file 2 — oc4c00656_si_002.zip [file oc4c00656_si_002.zip › FigureS44.jpg]

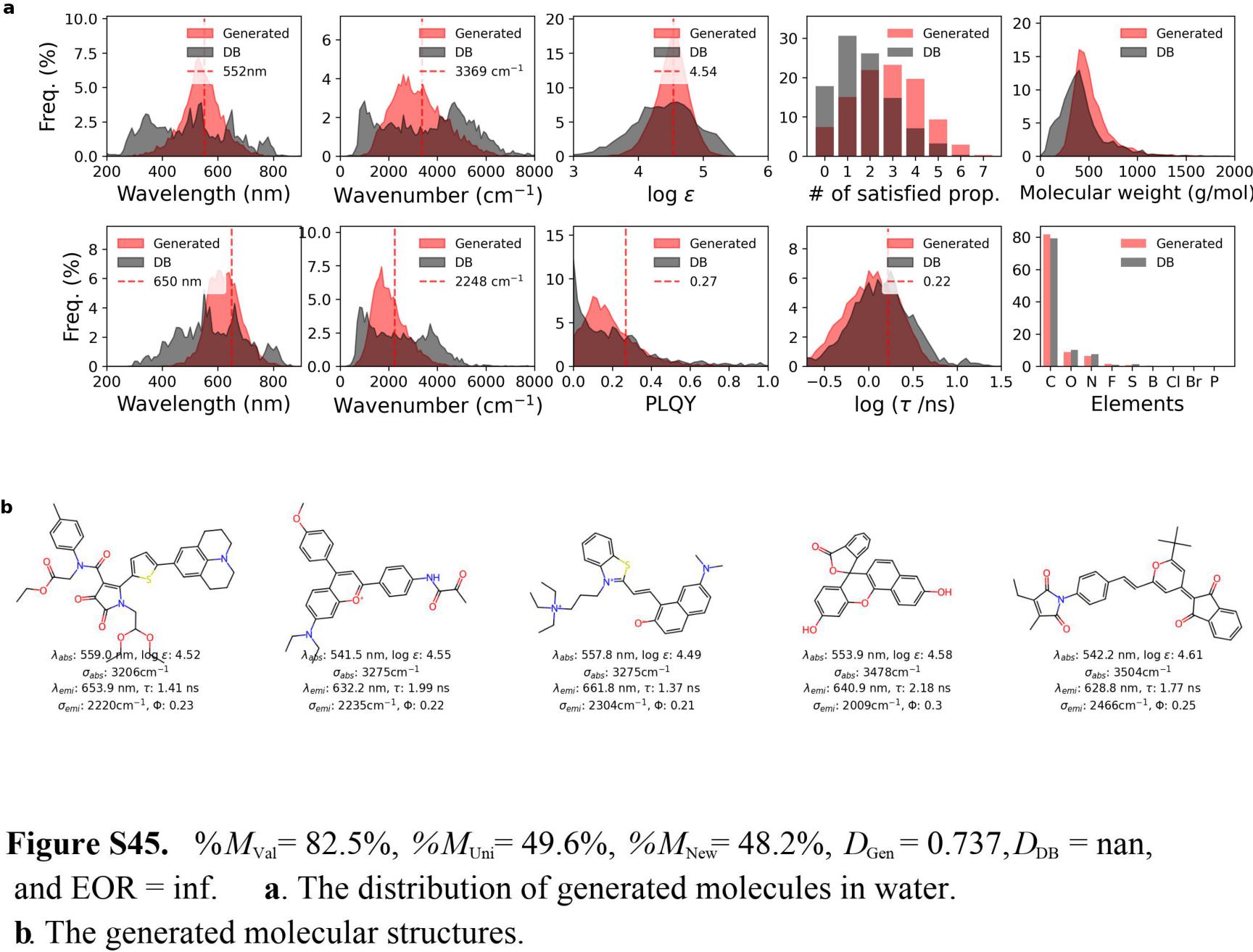

Supplement: Supplementary file 2 — oc4c00656_si_002.zip [file oc4c00656_si_002.zip › FigureS45.jpg]

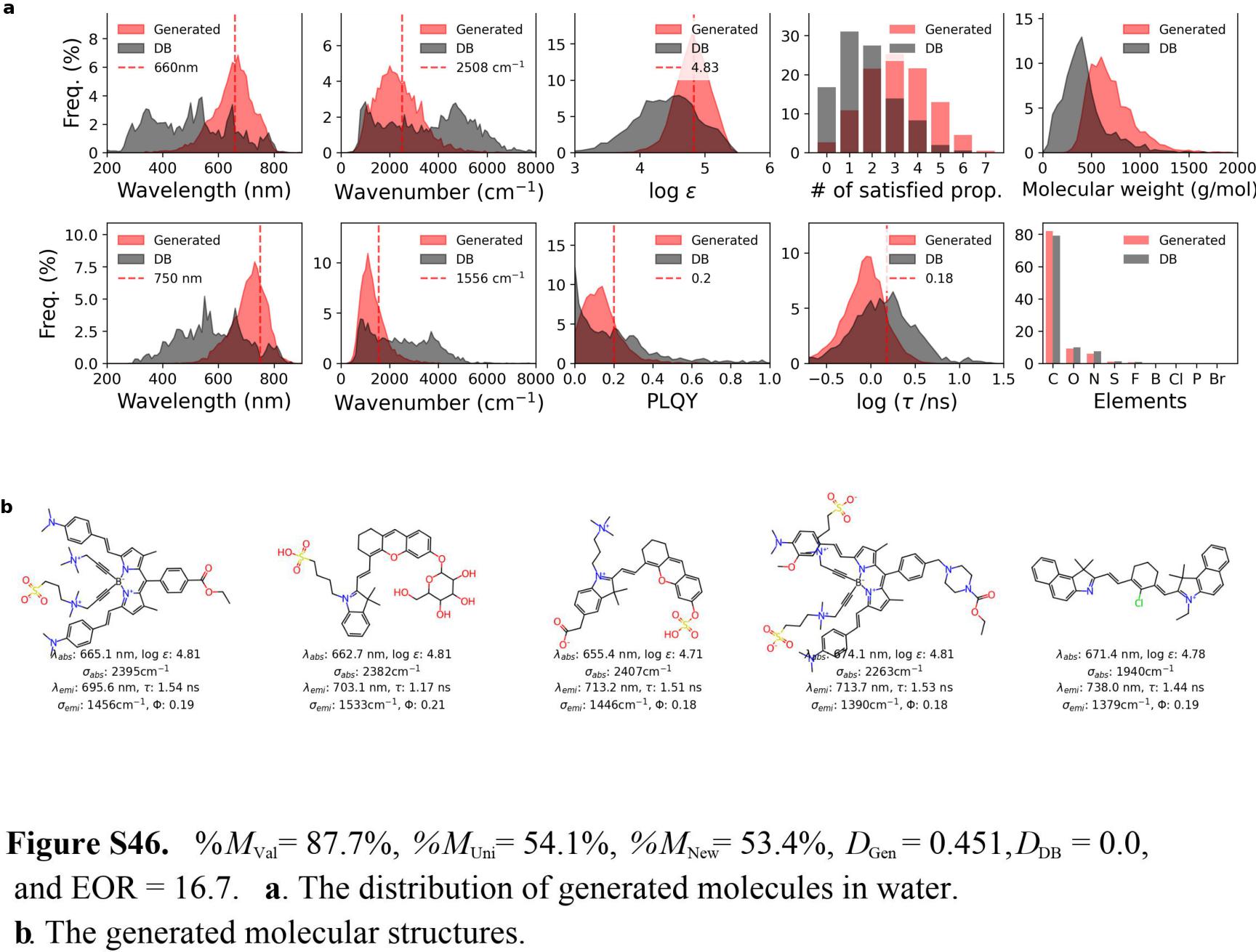

Supplement: Supplementary file 2 — oc4c00656_si_002.zip [file oc4c00656_si_002.zip › FigureS46.jpg]

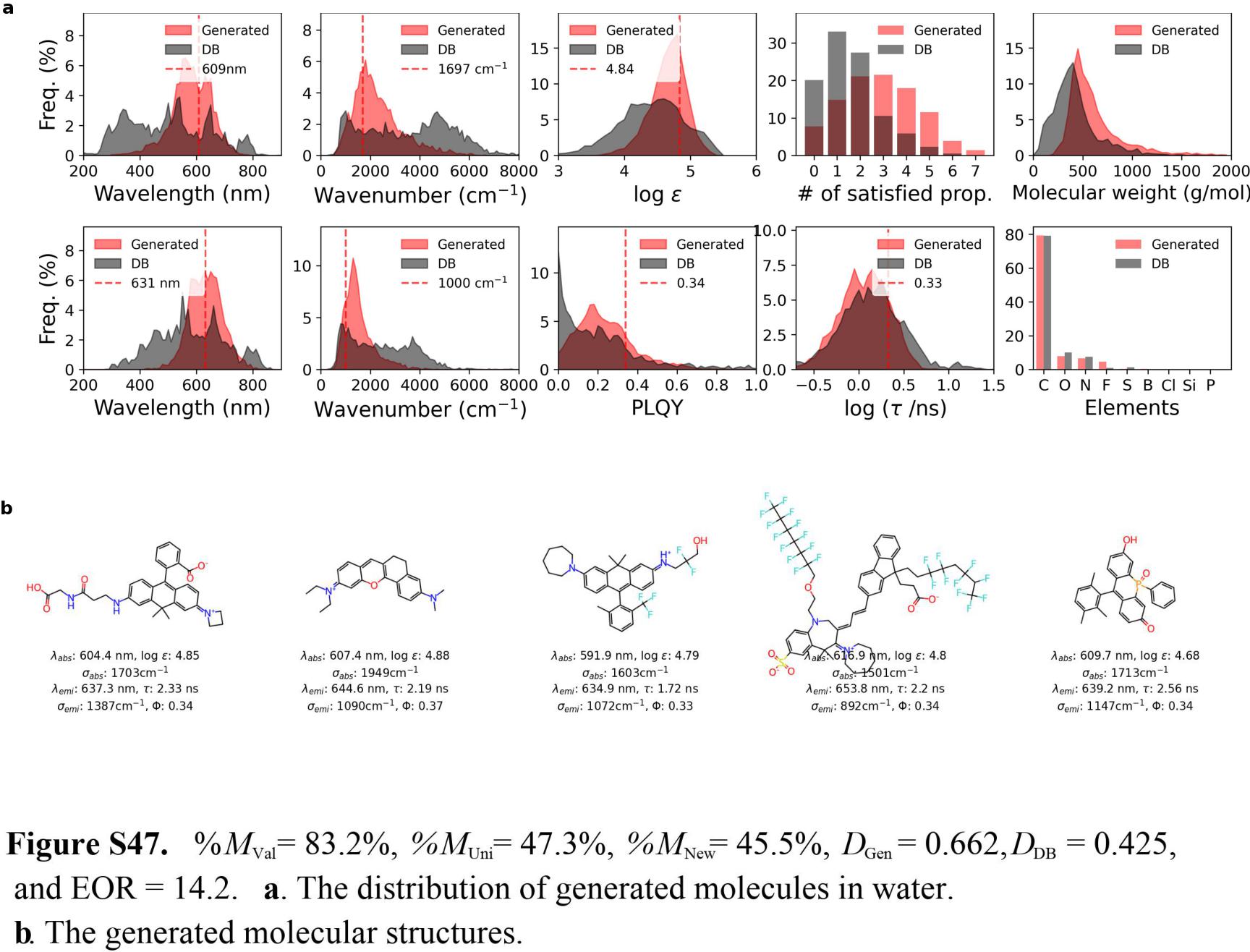

Supplement: Supplementary file 2 — oc4c00656_si_002.zip [file oc4c00656_si_002.zip › FigureS47.jpg]

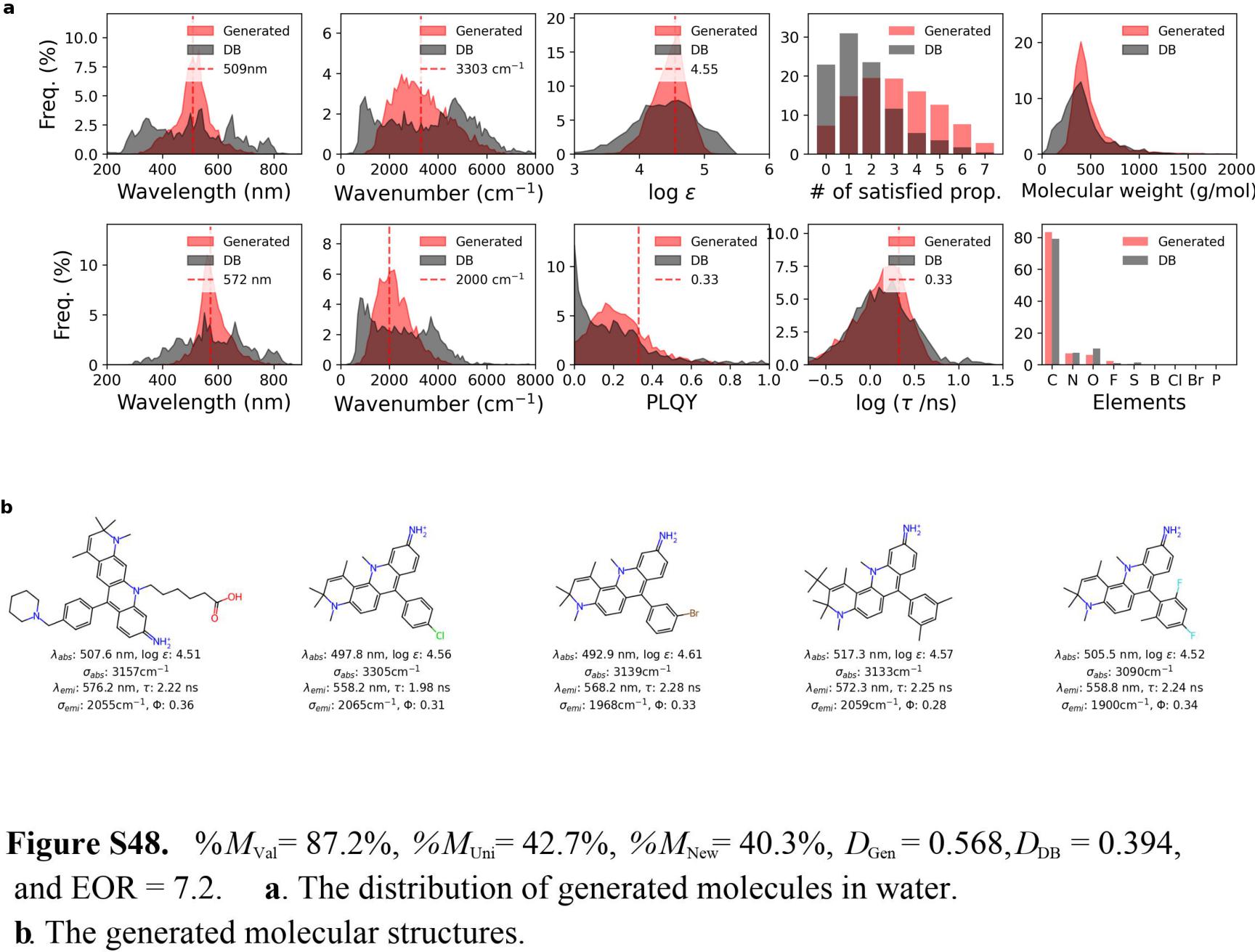

Supplement: Supplementary file 2 — oc4c00656_si_002.zip [file oc4c00656_si_002.zip › FigureS48.jpg]

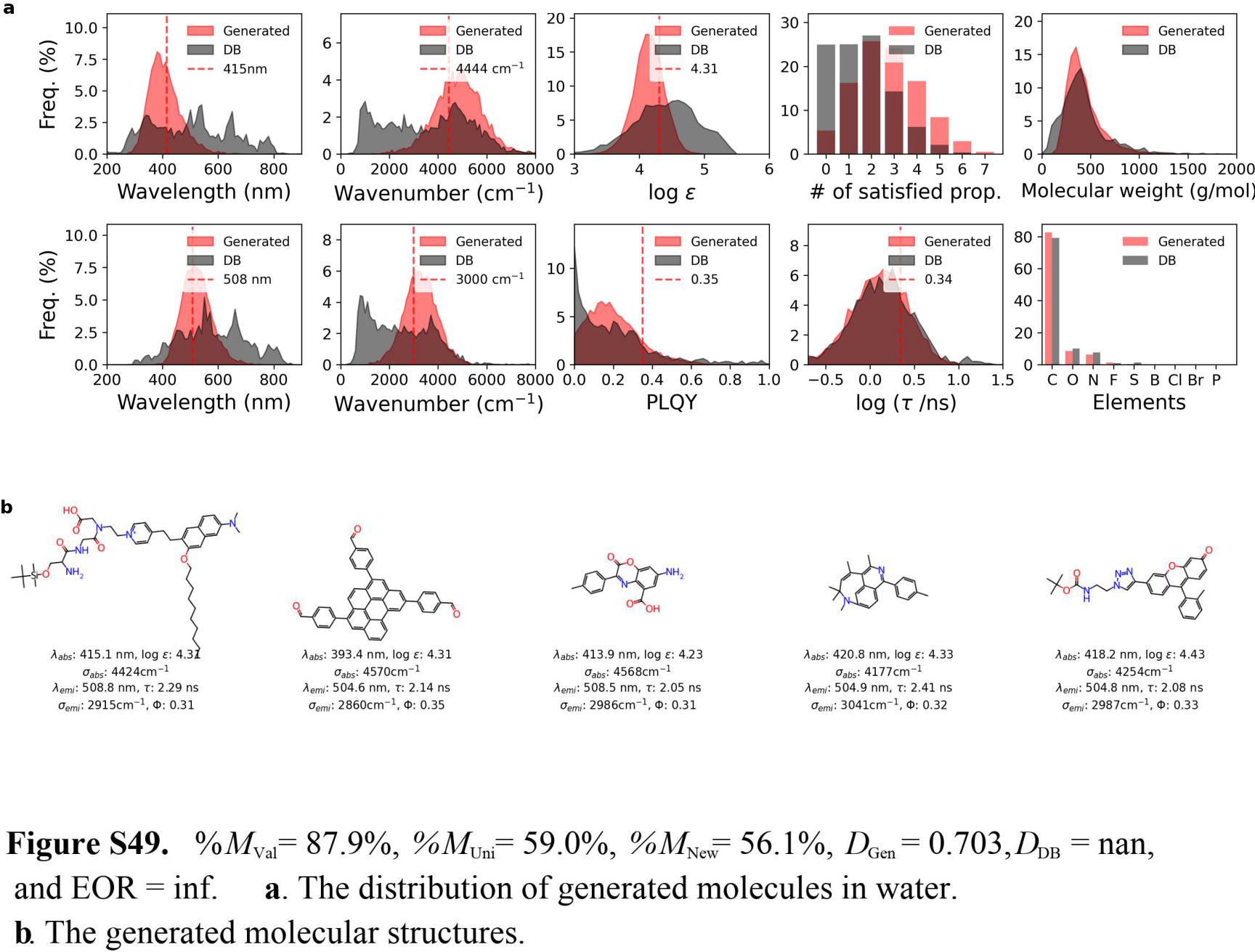

Supplement: Supplementary file 2 — oc4c00656_si_002.zip [file oc4c00656_si_002.zip › FigureS49.jpg]

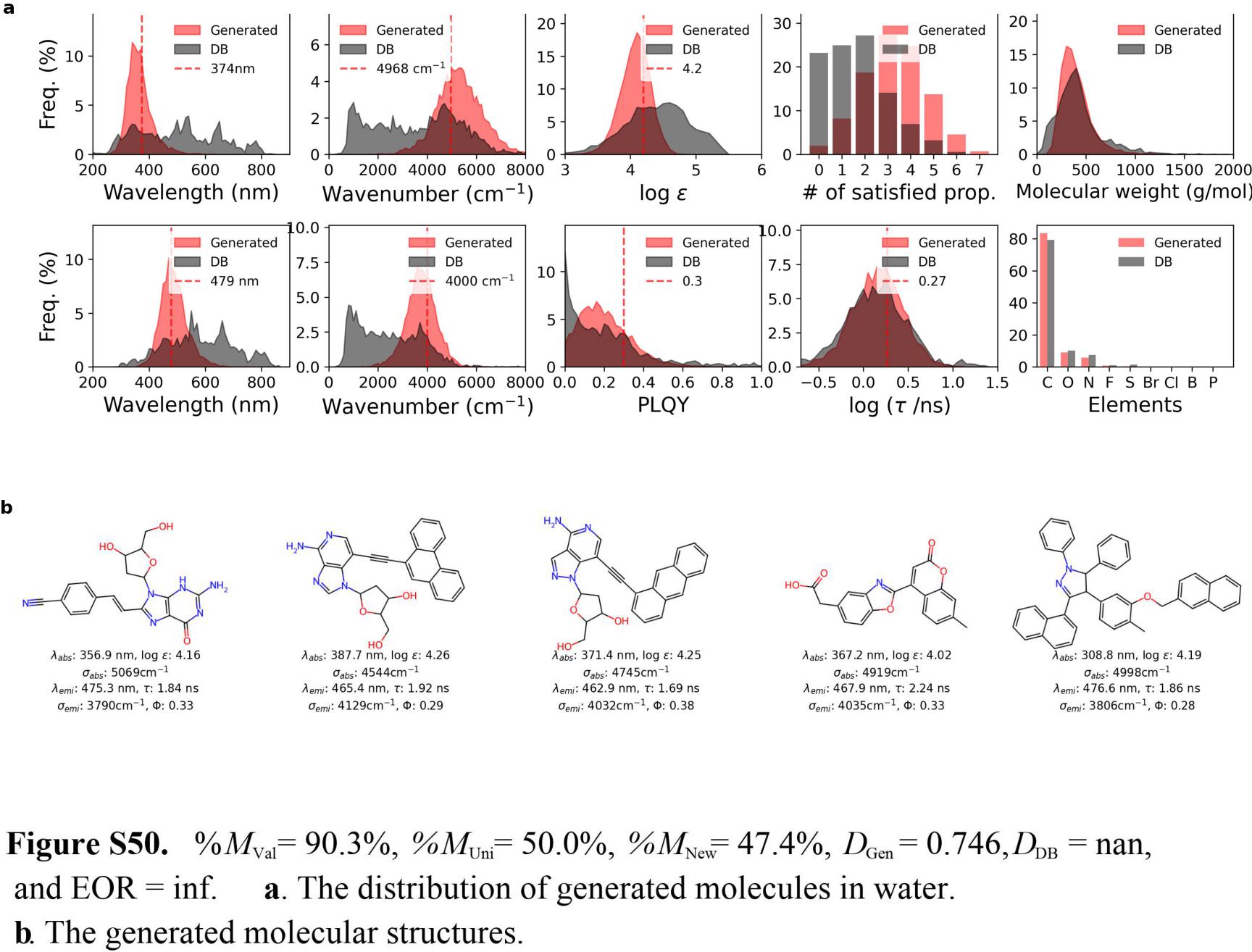

Supplement: Supplementary file 2 — oc4c00656_si_002.zip [file oc4c00656_si_002.zip › FigureS50.jpg]

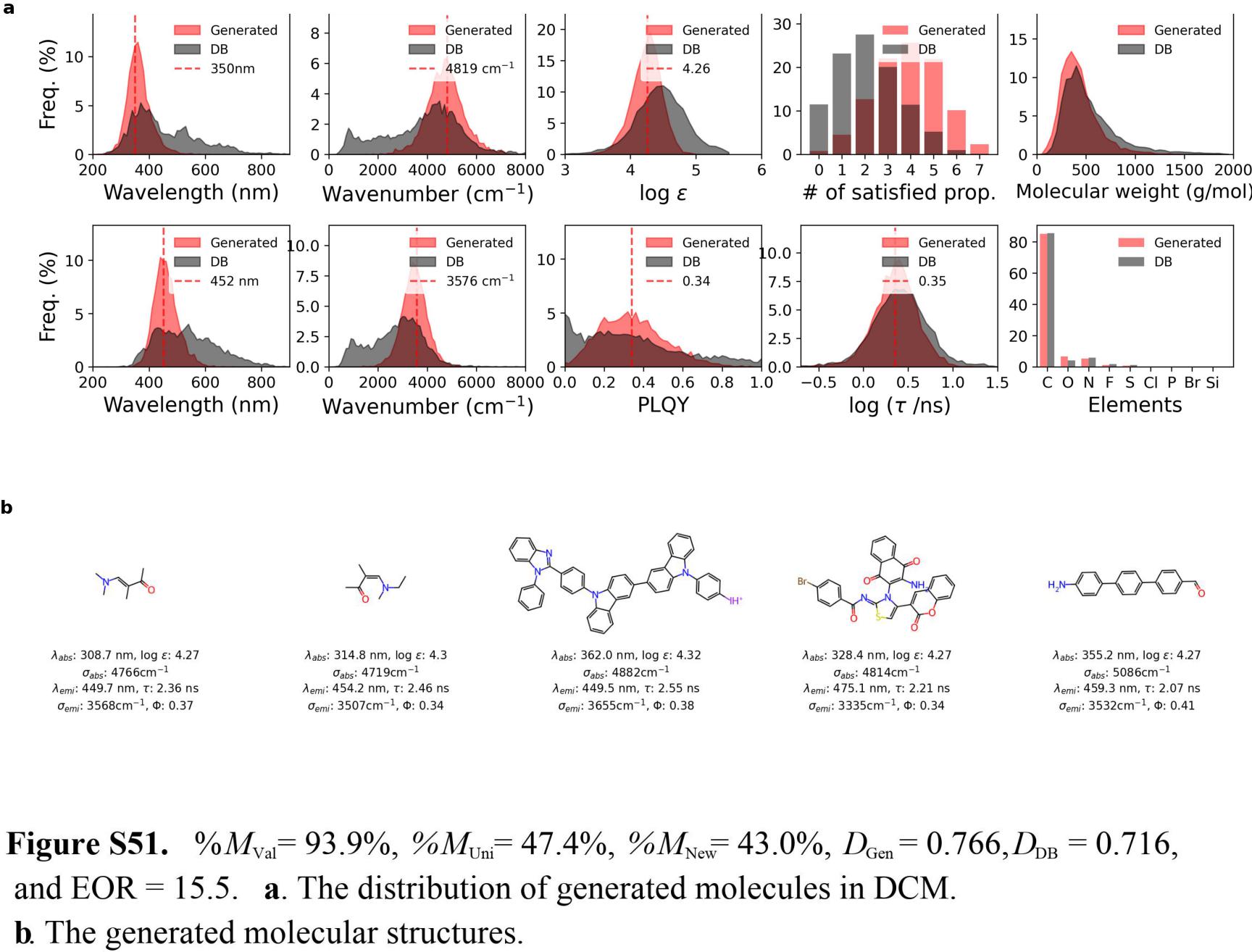

Supplement: Supplementary file 2 — oc4c00656_si_002.zip [file oc4c00656_si_002.zip › FigureS51.jpg]

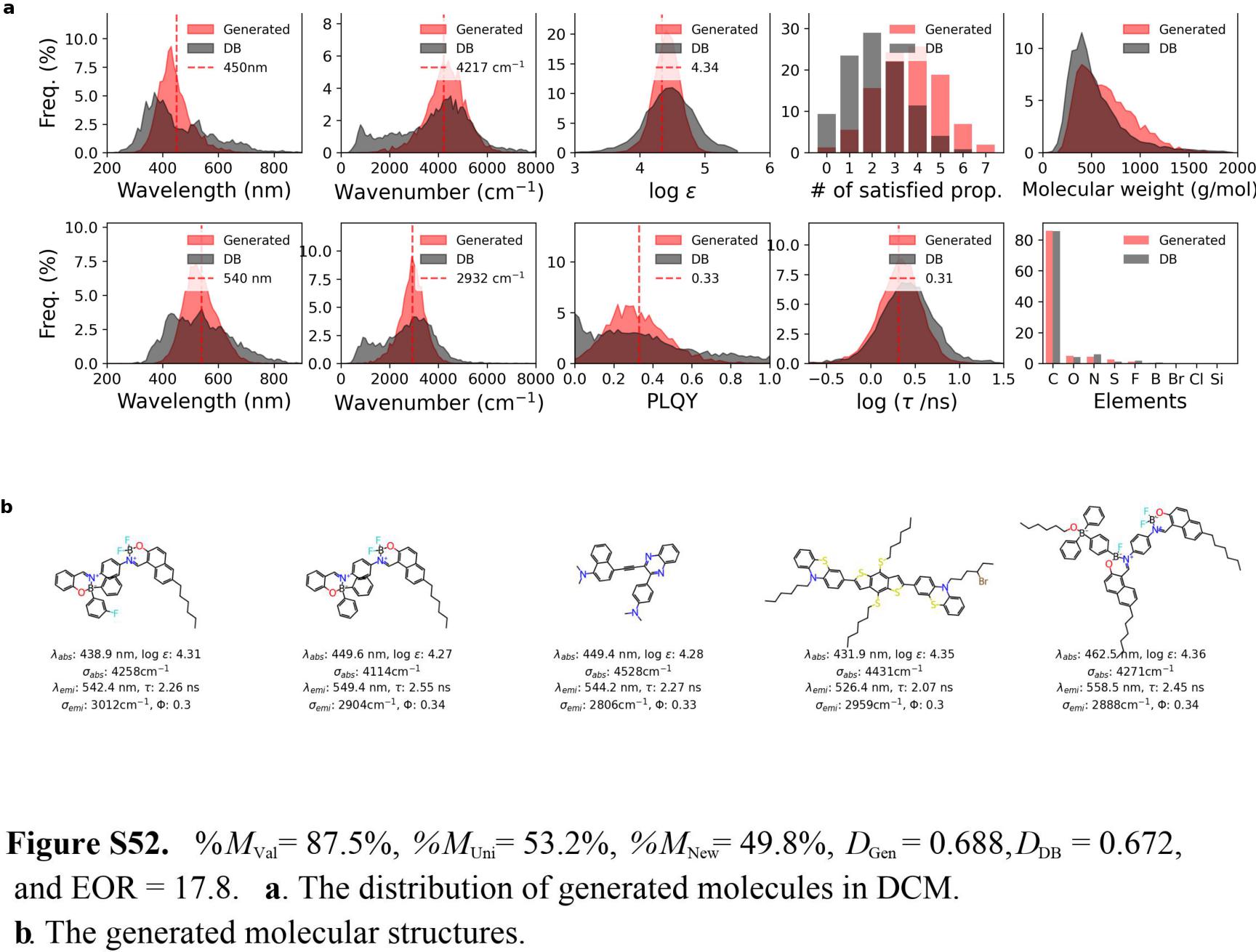

Supplement: Supplementary file 2 — oc4c00656_si_002.zip [file oc4c00656_si_002.zip › FigureS52.jpg]

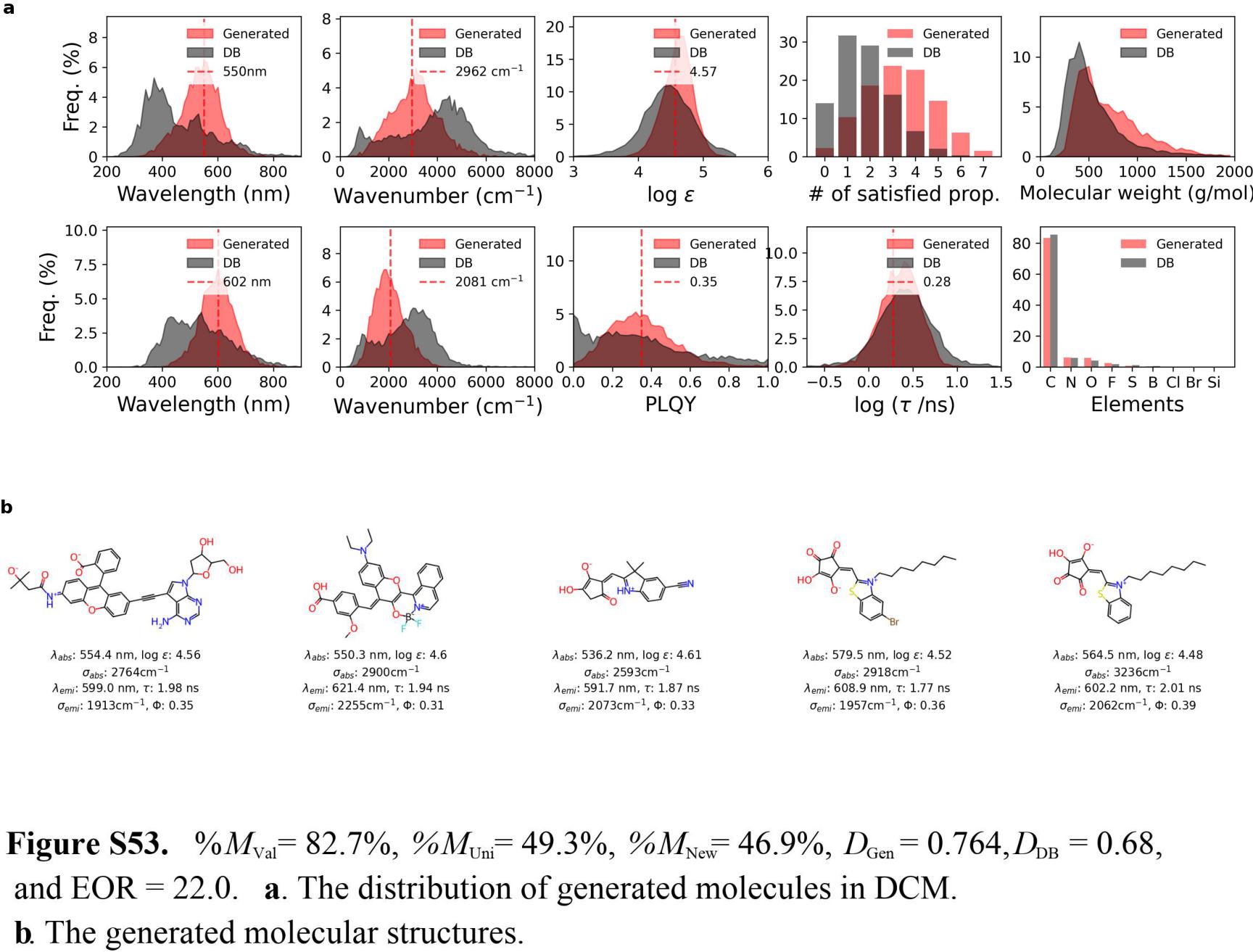

Supplement: Supplementary file 2 — oc4c00656_si_002.zip [file oc4c00656_si_002.zip › FigureS53.jpg]

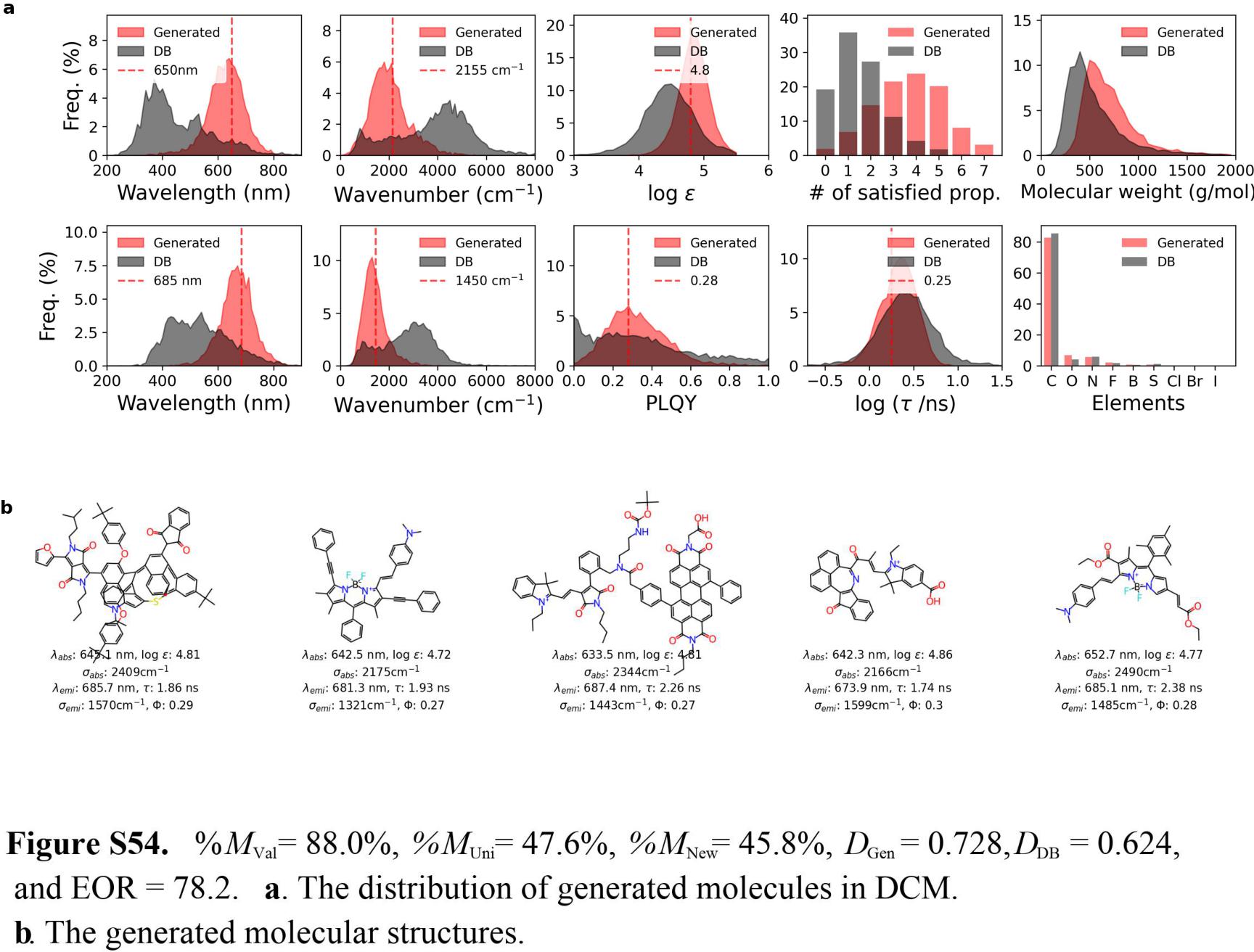

Supplement: Supplementary file 2 — oc4c00656_si_002.zip [file oc4c00656_si_002.zip › FigureS54.jpg]

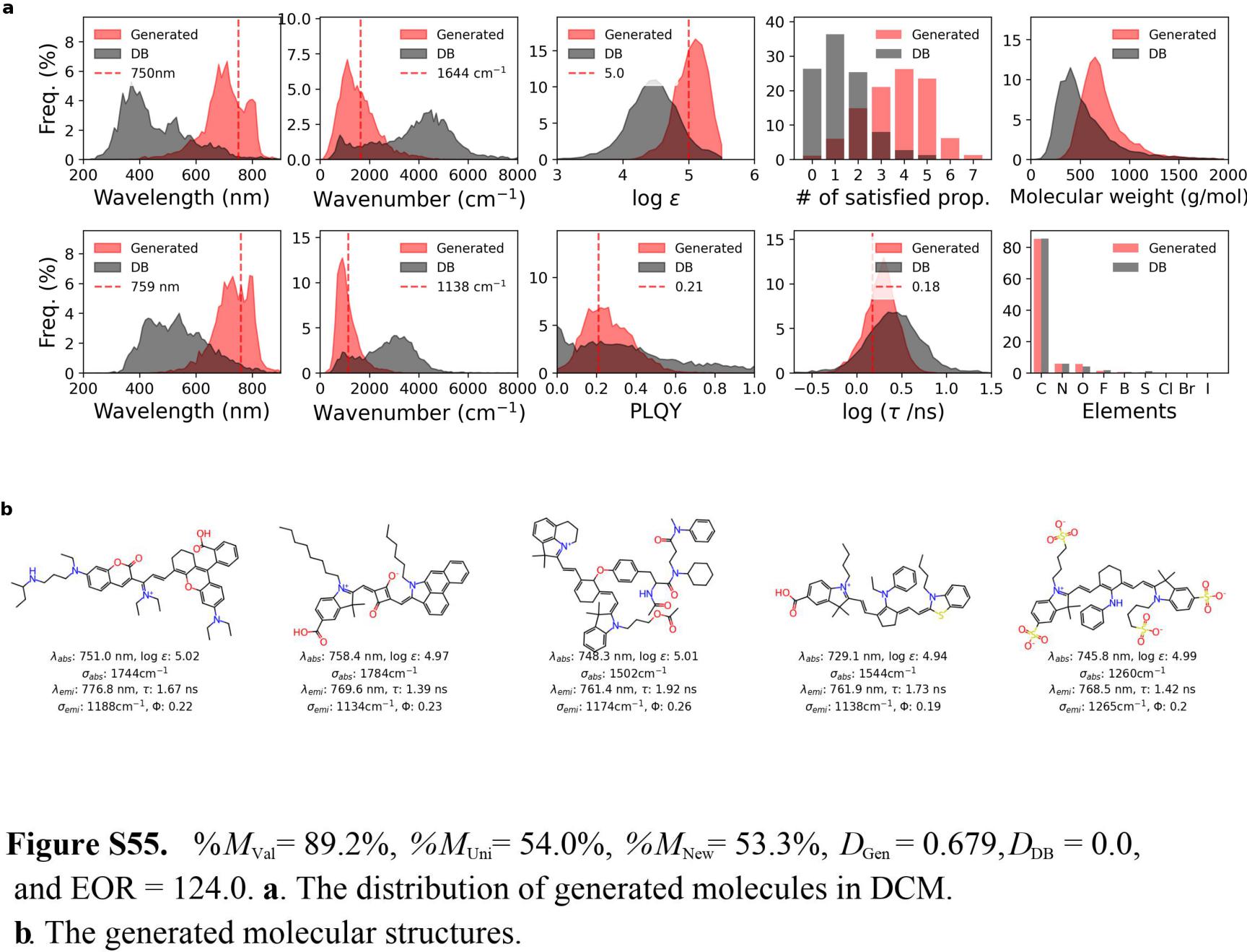

Supplement: Supplementary file 2 — oc4c00656_si_002.zip [file oc4c00656_si_002.zip › FigureS55.jpg]

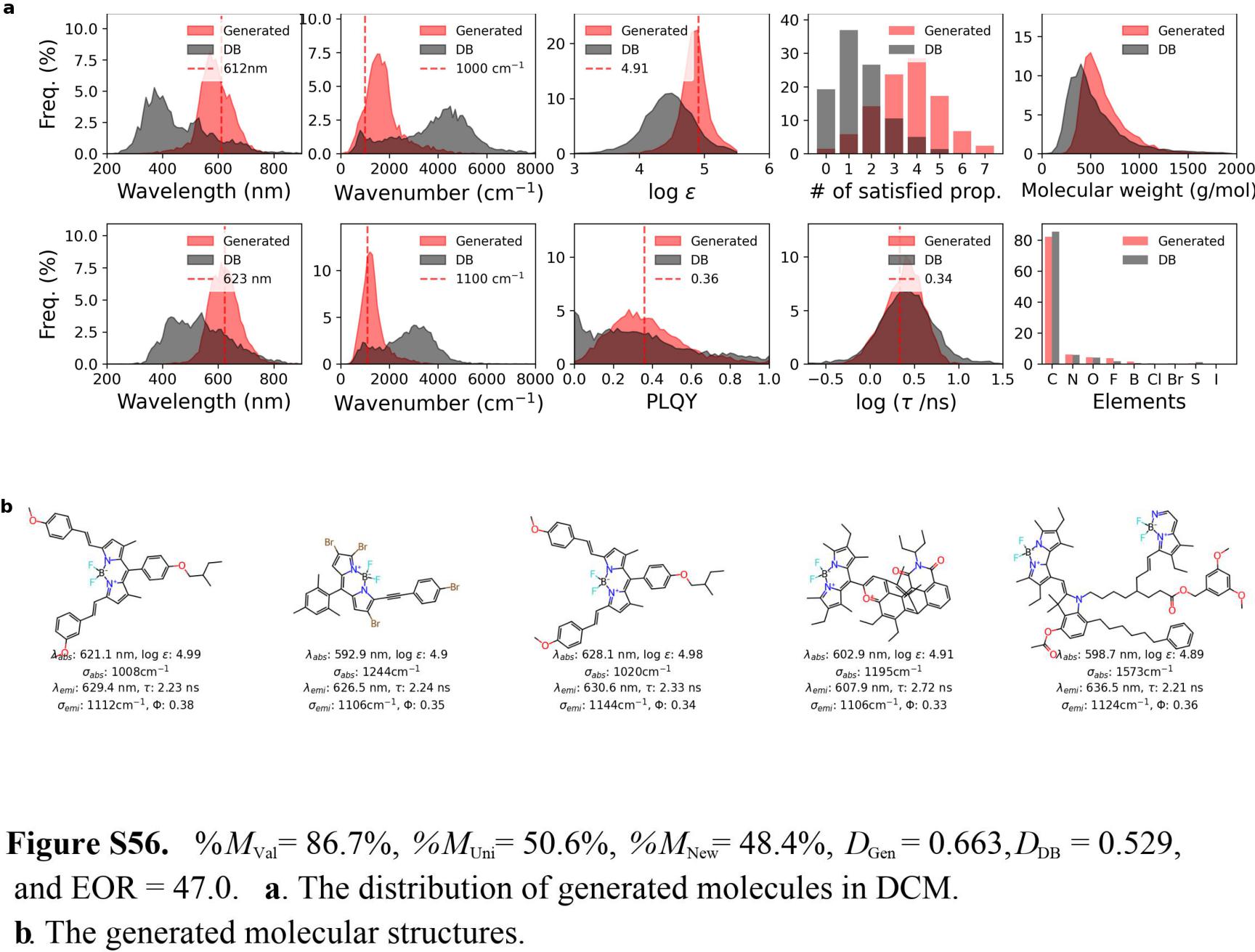

Supplement: Supplementary file 2 — oc4c00656_si_002.zip [file oc4c00656_si_002.zip › FigureS56.jpg]

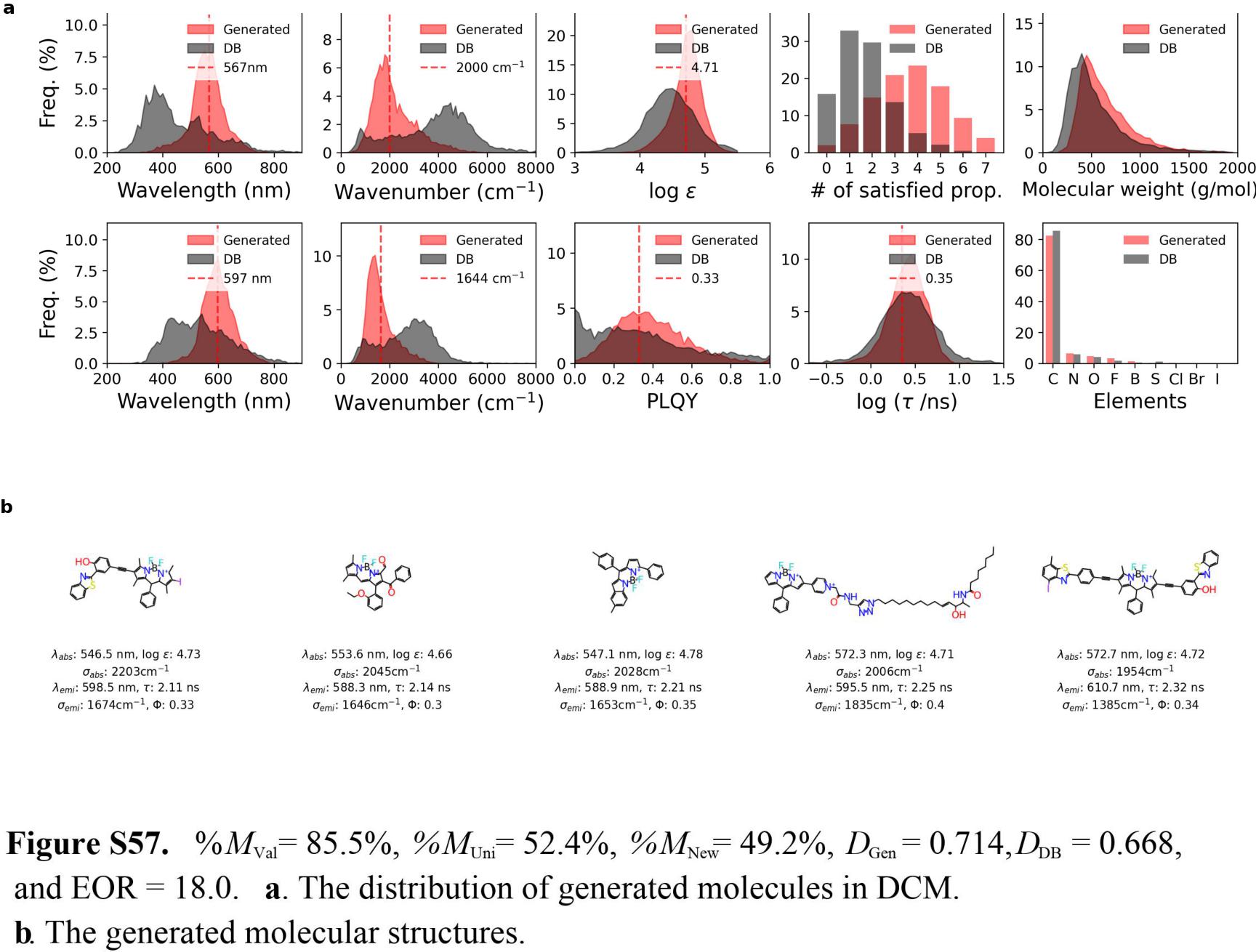

Supplement: Supplementary file 2 — oc4c00656_si_002.zip [file oc4c00656_si_002.zip › FigureS57.jpg]

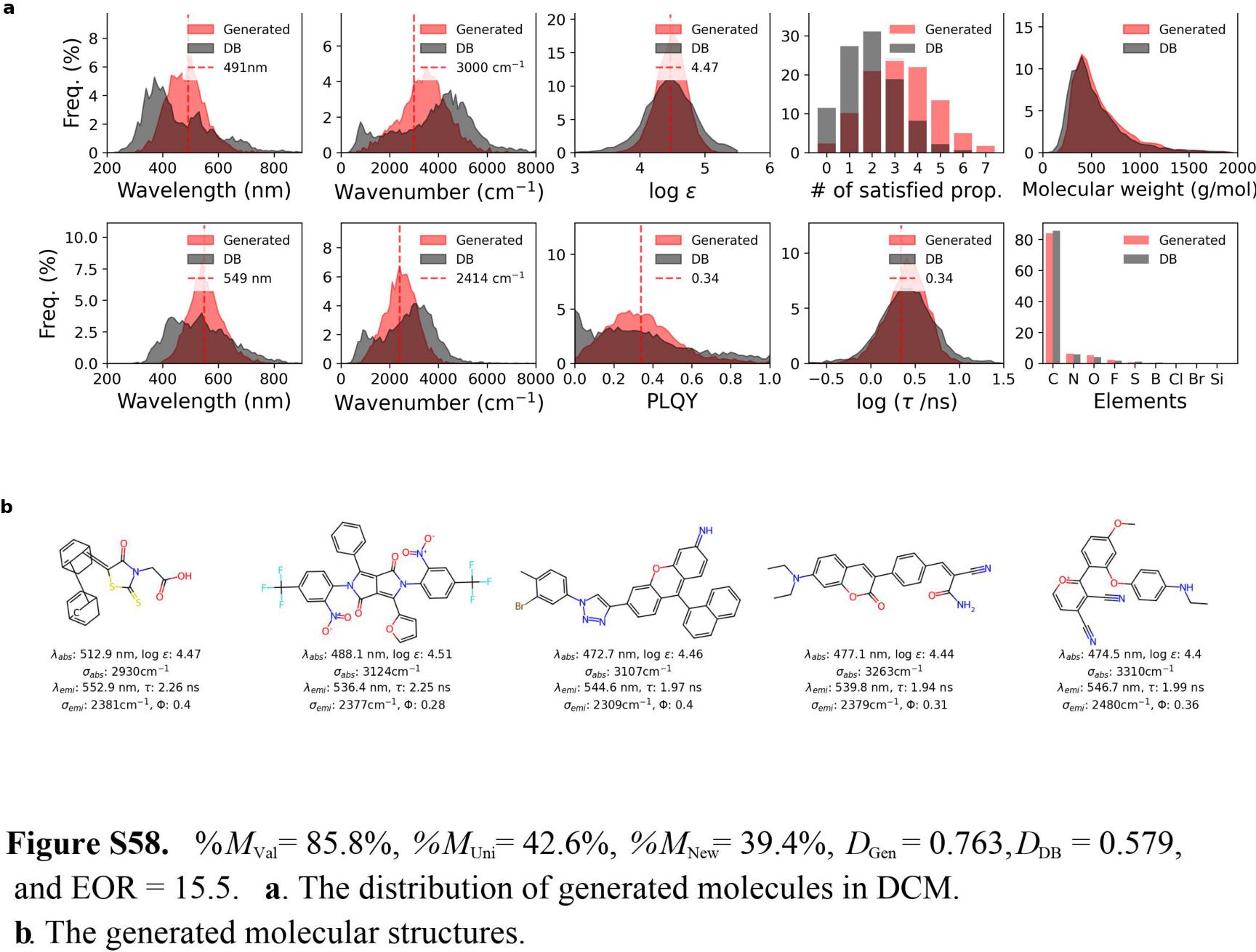

Supplement: Supplementary file 2 — oc4c00656_si_002.zip [file oc4c00656_si_002.zip › FigureS58.jpg]

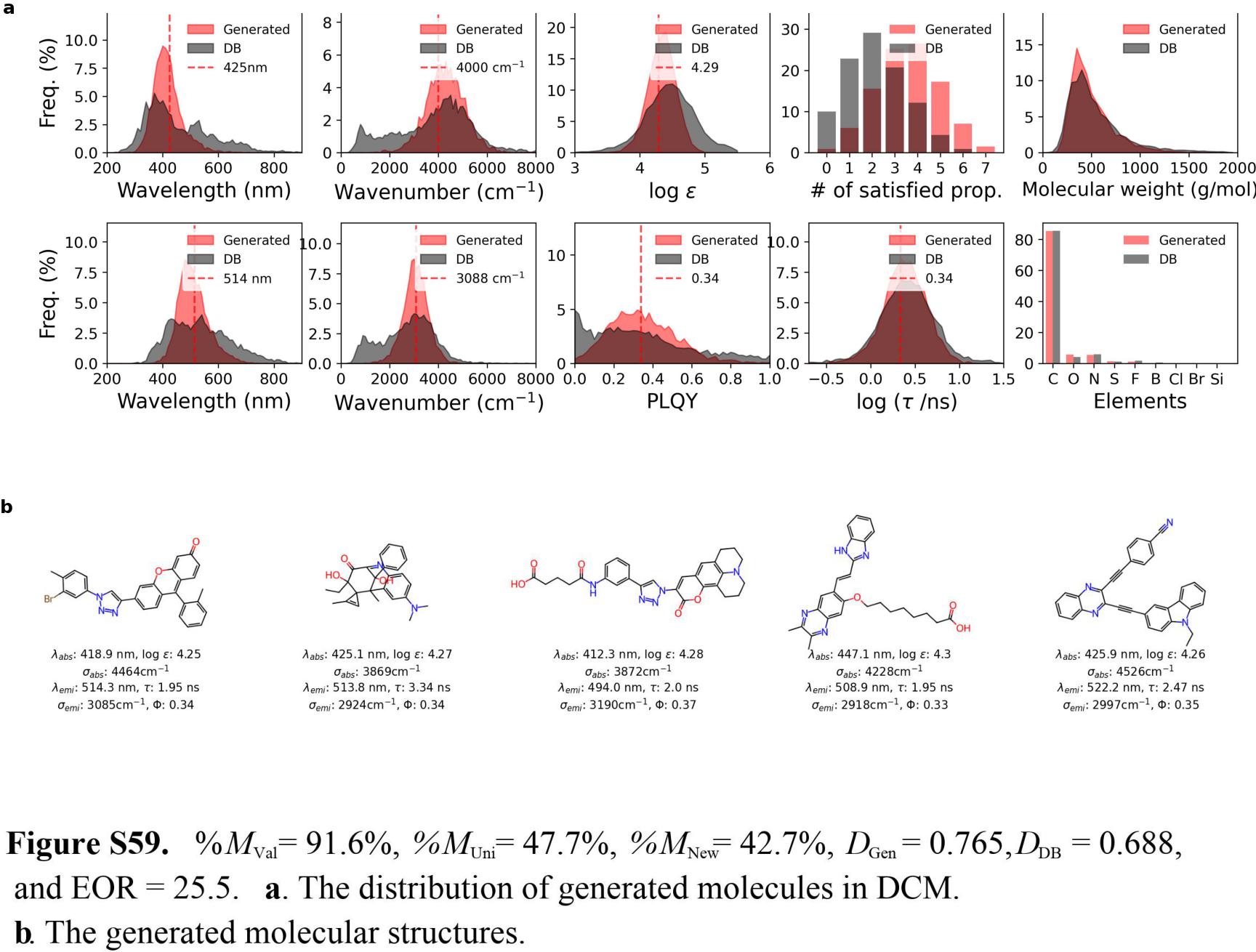

Supplement: Supplementary file 2 — oc4c00656_si_002.zip [file oc4c00656_si_002.zip › FigureS59.jpg]

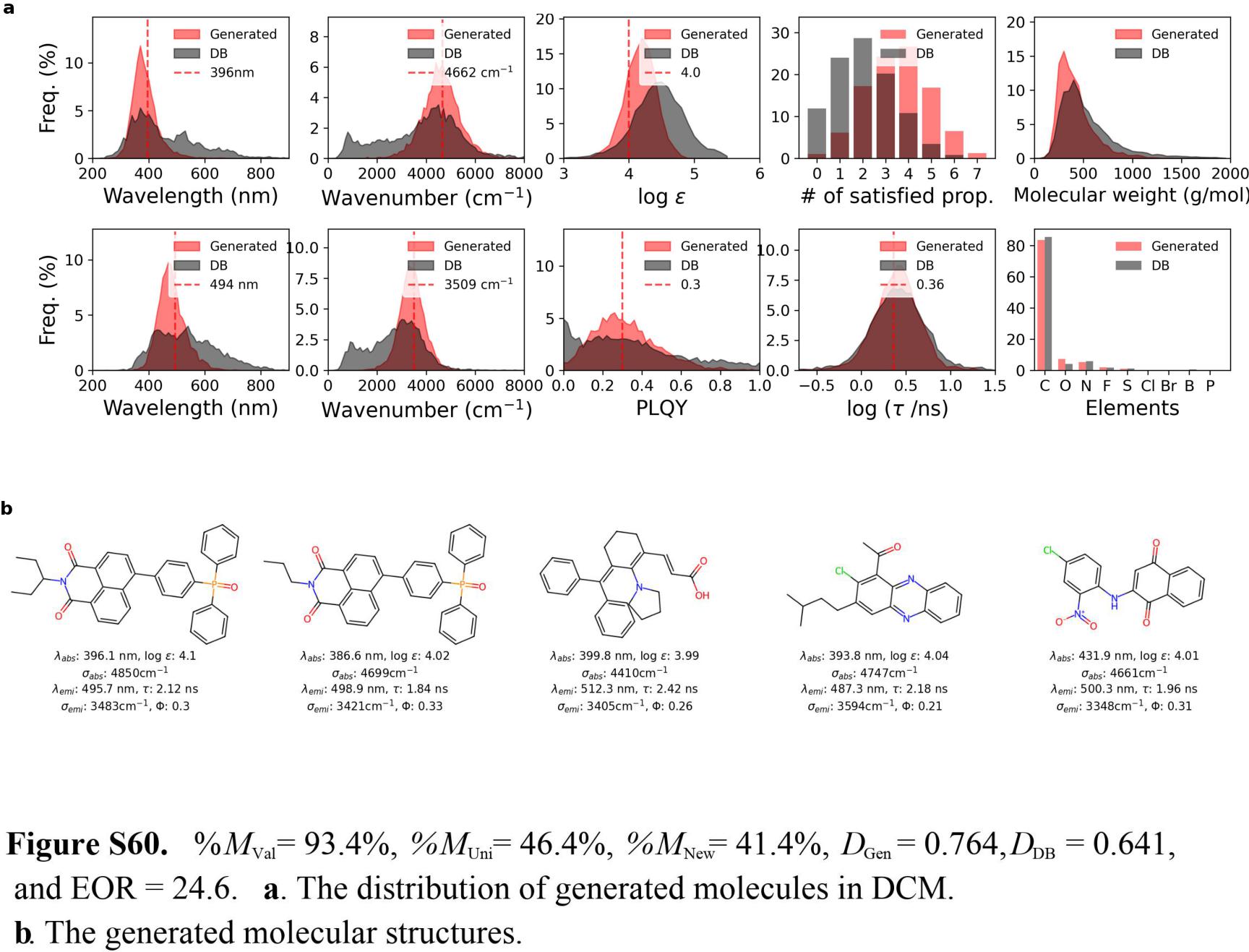

Supplement: Supplementary file 2 — oc4c00656_si_002.zip [file oc4c00656_si_002.zip › FigureS60.jpg]

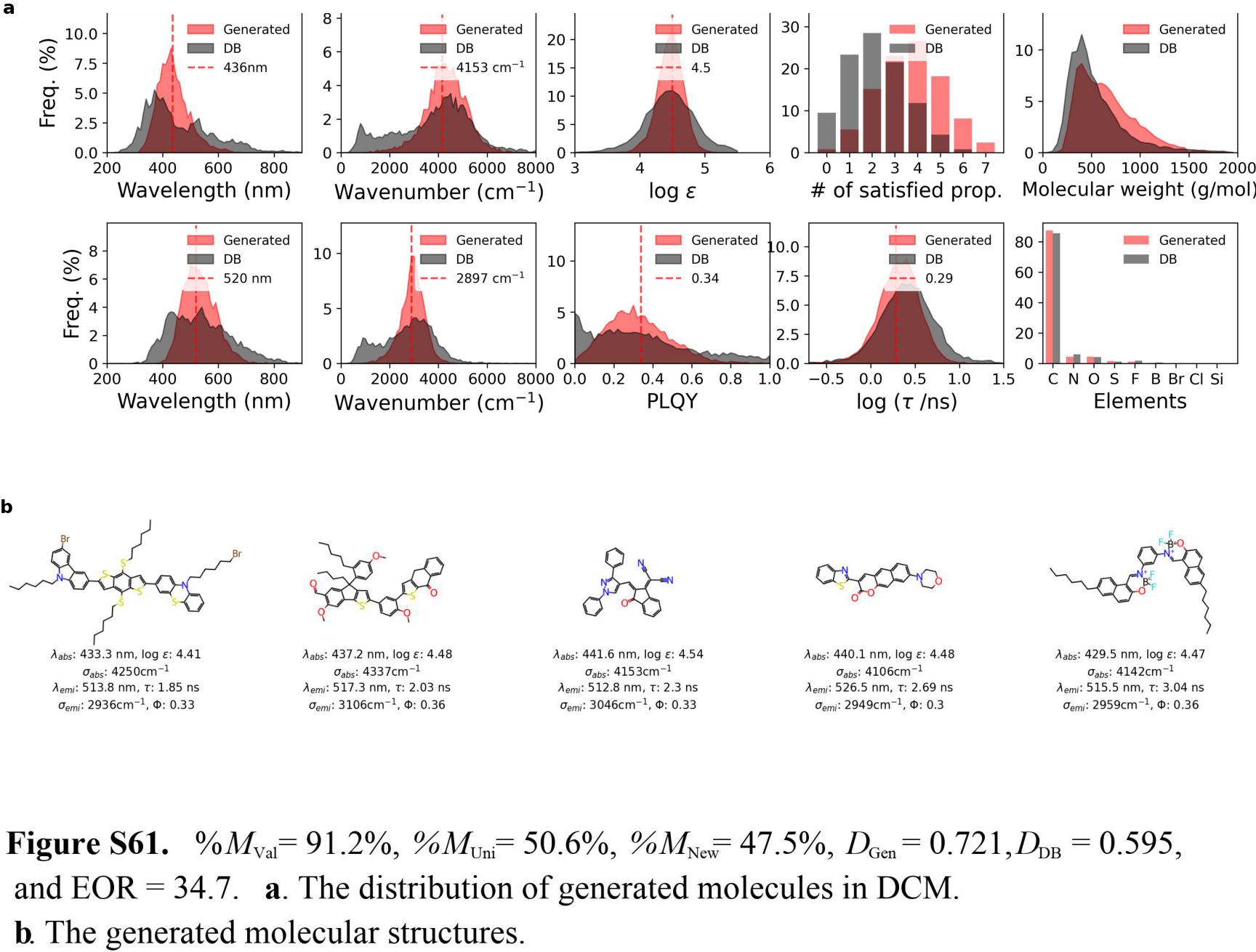

Supplement: Supplementary file 2 — oc4c00656_si_002.zip [file oc4c00656_si_002.zip › FigureS61.jpg]

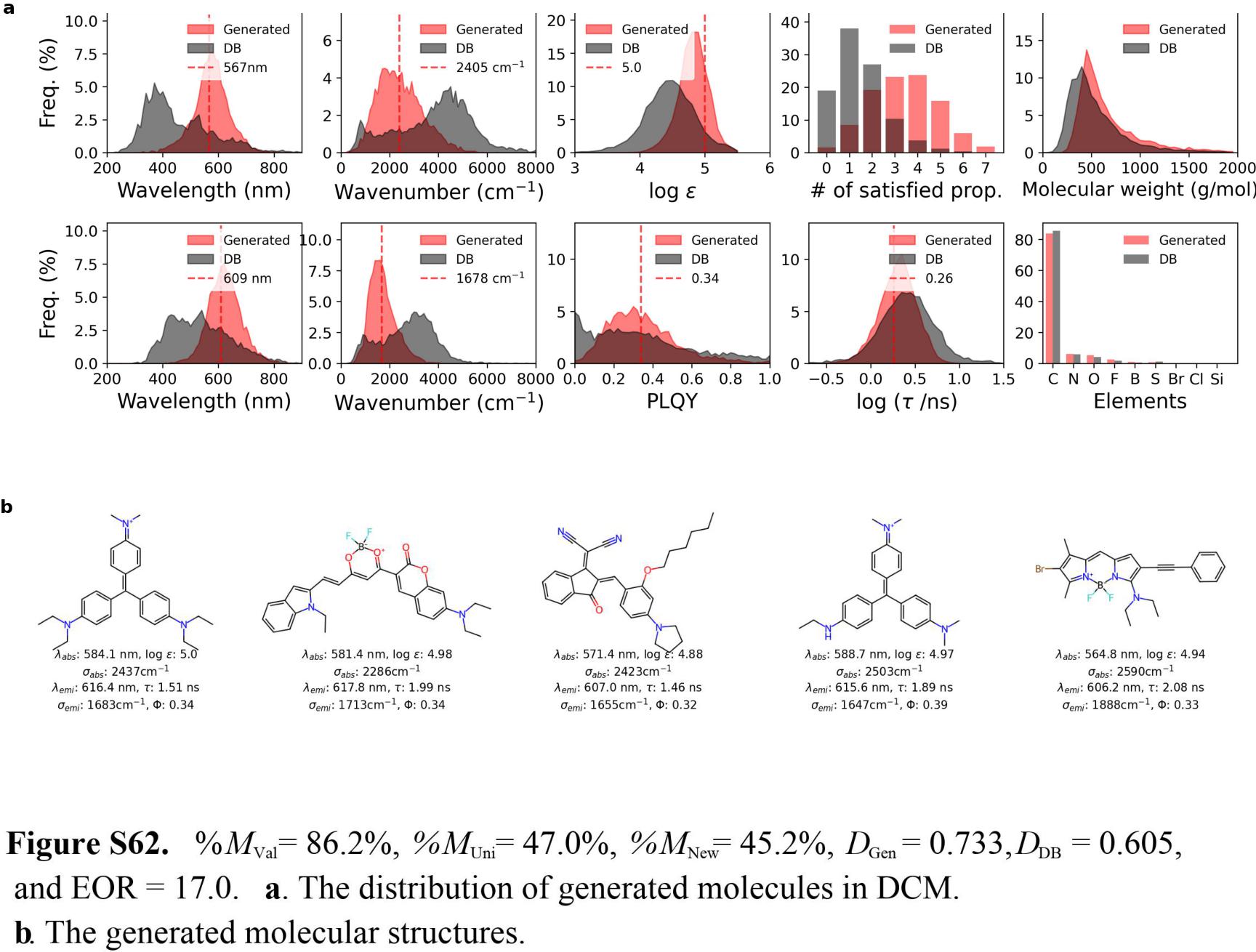

Supplement: Supplementary file 2 — oc4c00656_si_002.zip [file oc4c00656_si_002.zip › FigureS62.jpg]

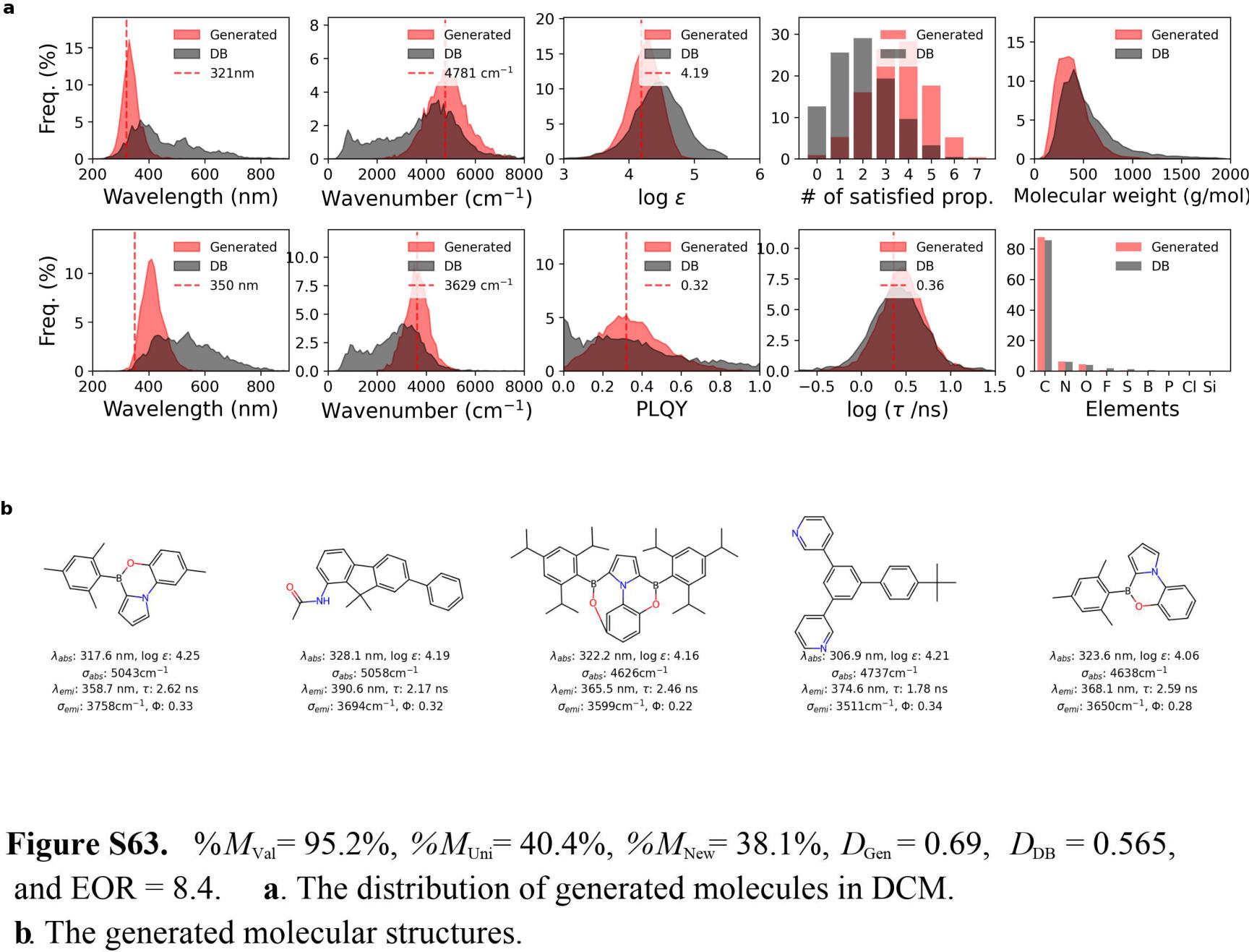

Supplement: Supplementary file 2 — oc4c00656_si_002.zip [file oc4c00656_si_002.zip › FigureS63.jpg]

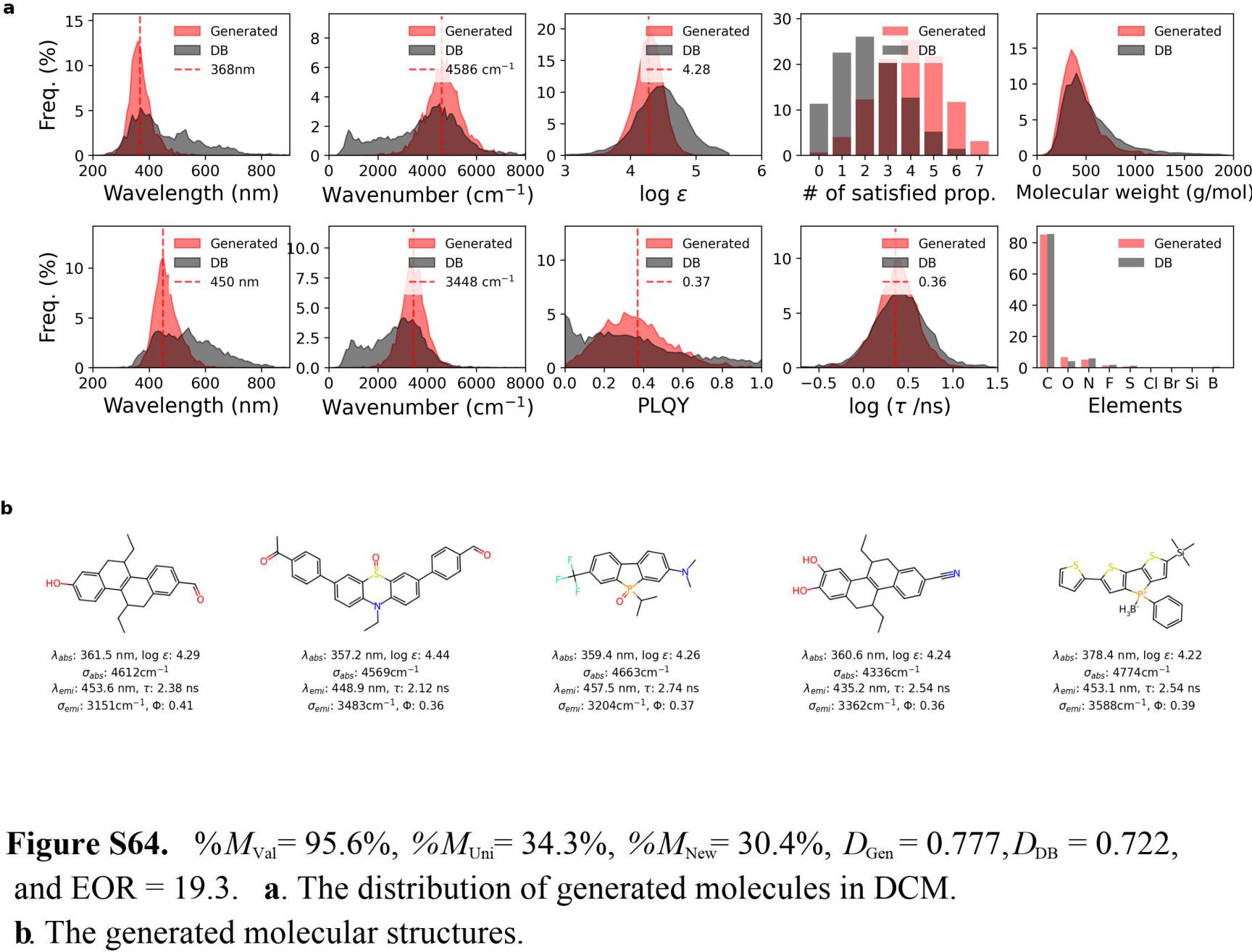

Supplement: Supplementary file 2 — oc4c00656_si_002.zip [file oc4c00656_si_002.zip › FigureS64.jpg]

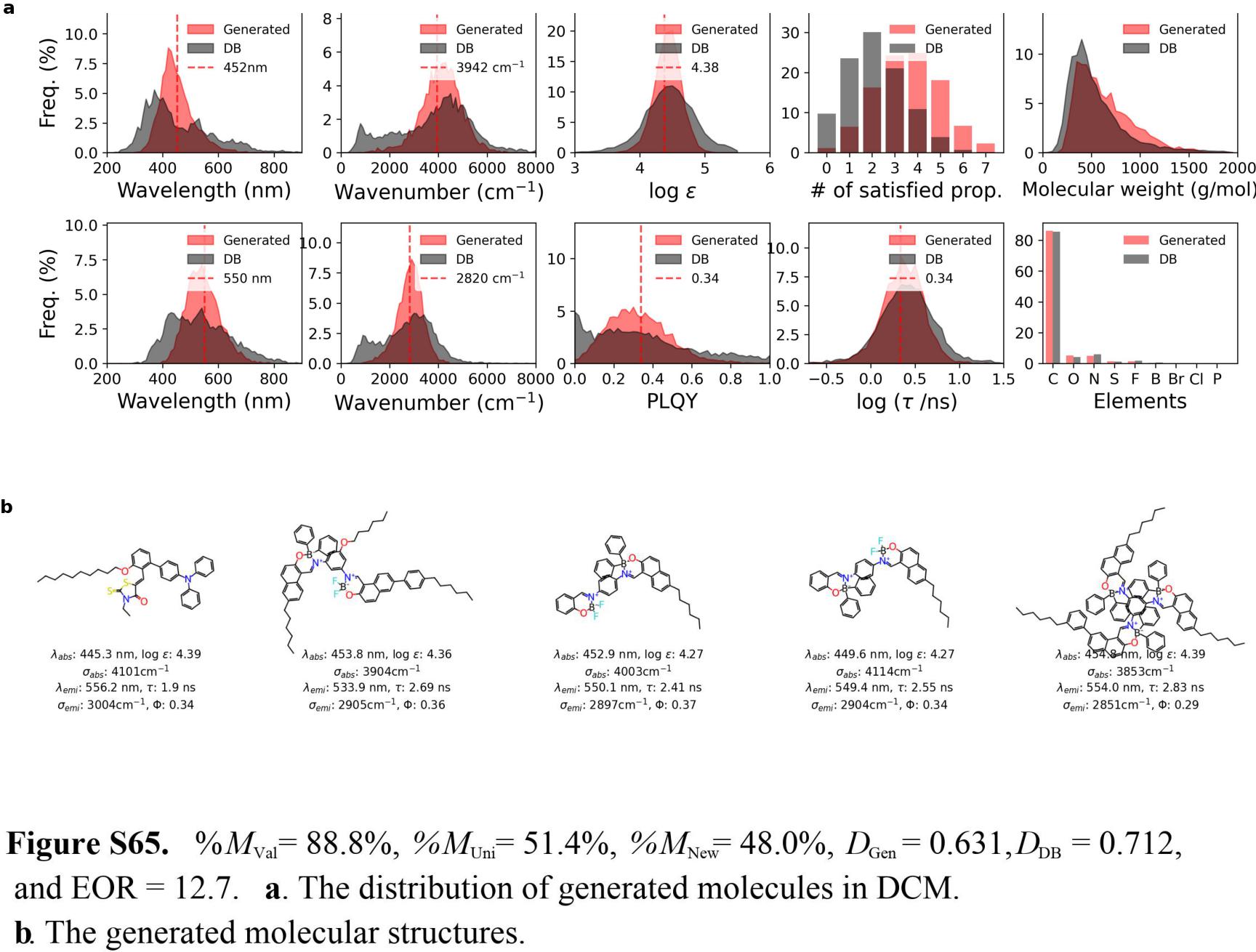

Supplement: Supplementary file 2 — oc4c00656_si_002.zip [file oc4c00656_si_002.zip › FigureS65.jpg]

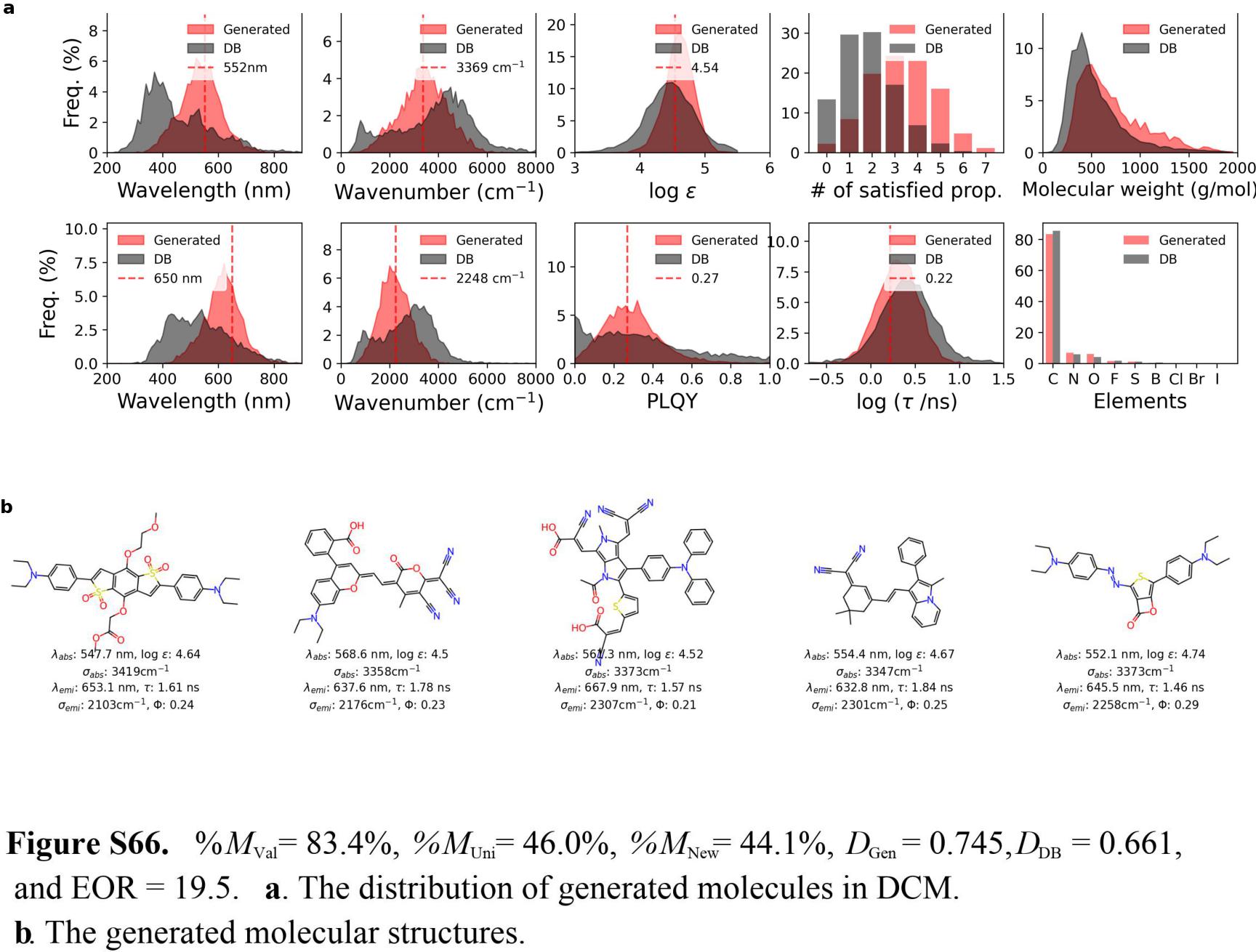

Supplement: Supplementary file 2 — oc4c00656_si_002.zip [file oc4c00656_si_002.zip › FigureS66.jpg]

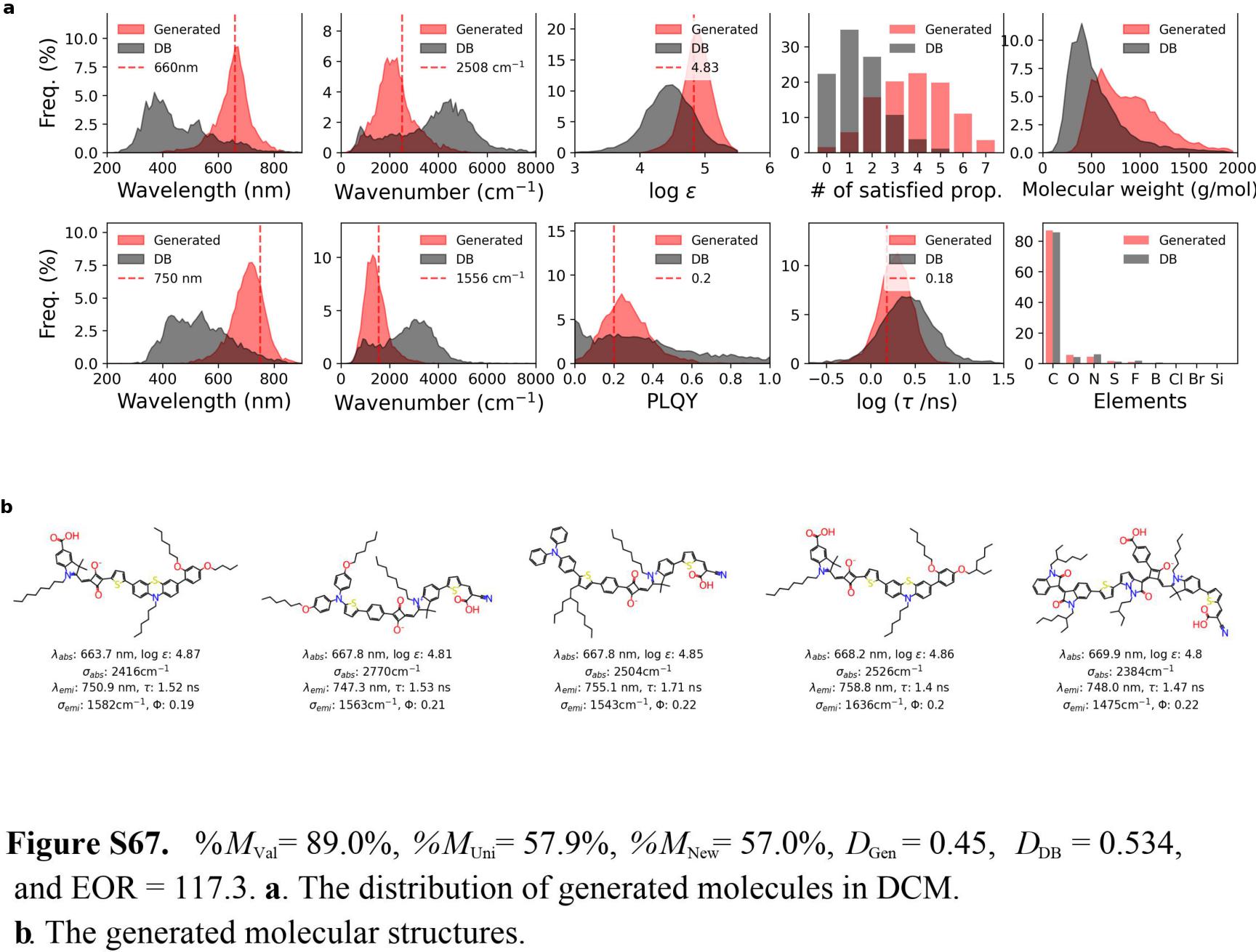

Supplement: Supplementary file 2 — oc4c00656_si_002.zip [file oc4c00656_si_002.zip › FigureS67.jpg]

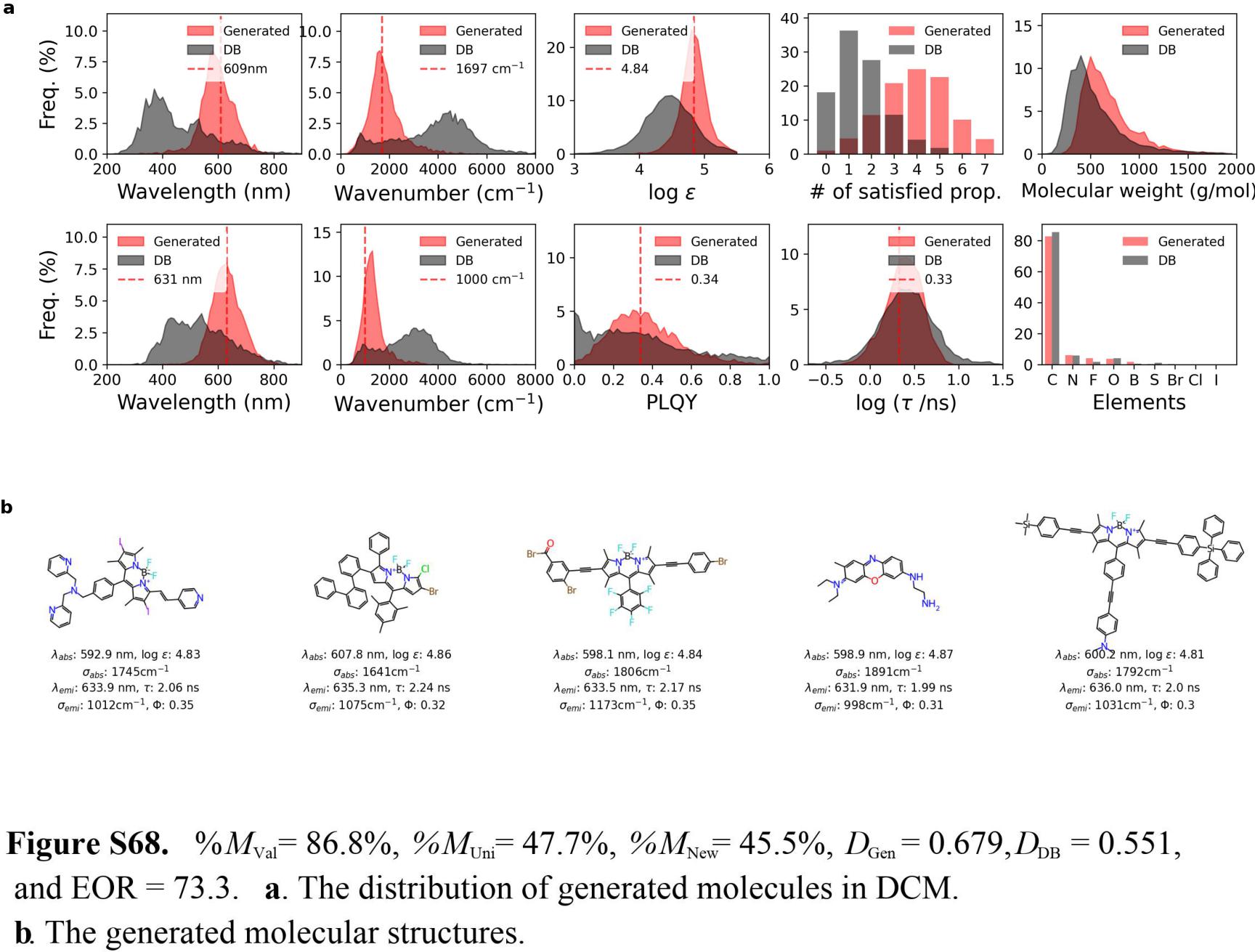

Supplement: Supplementary file 2 — oc4c00656_si_002.zip [file oc4c00656_si_002.zip › FigureS68.jpg]

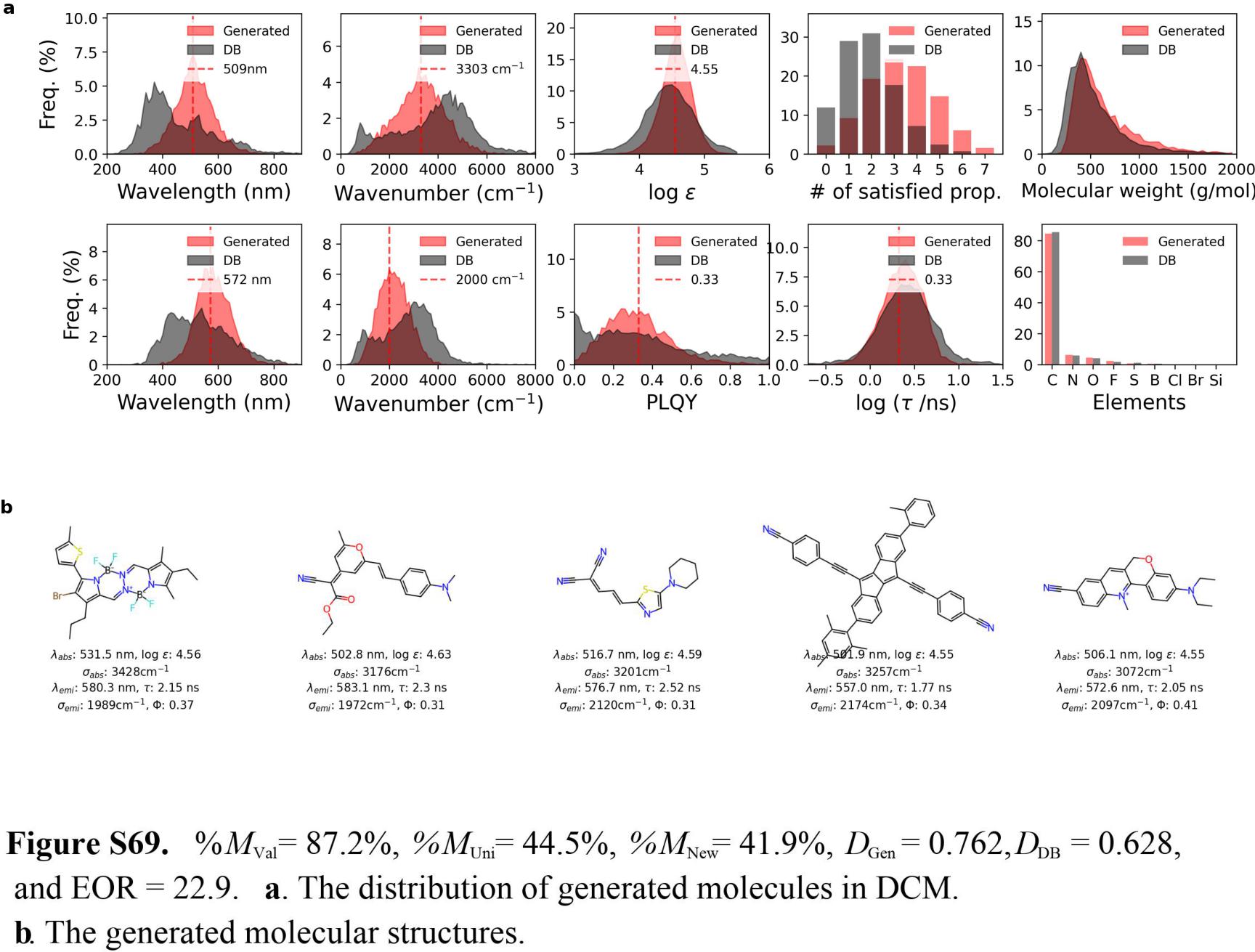

Supplement: Supplementary file 2 — oc4c00656_si_002.zip [file oc4c00656_si_002.zip › FigureS69.jpg]

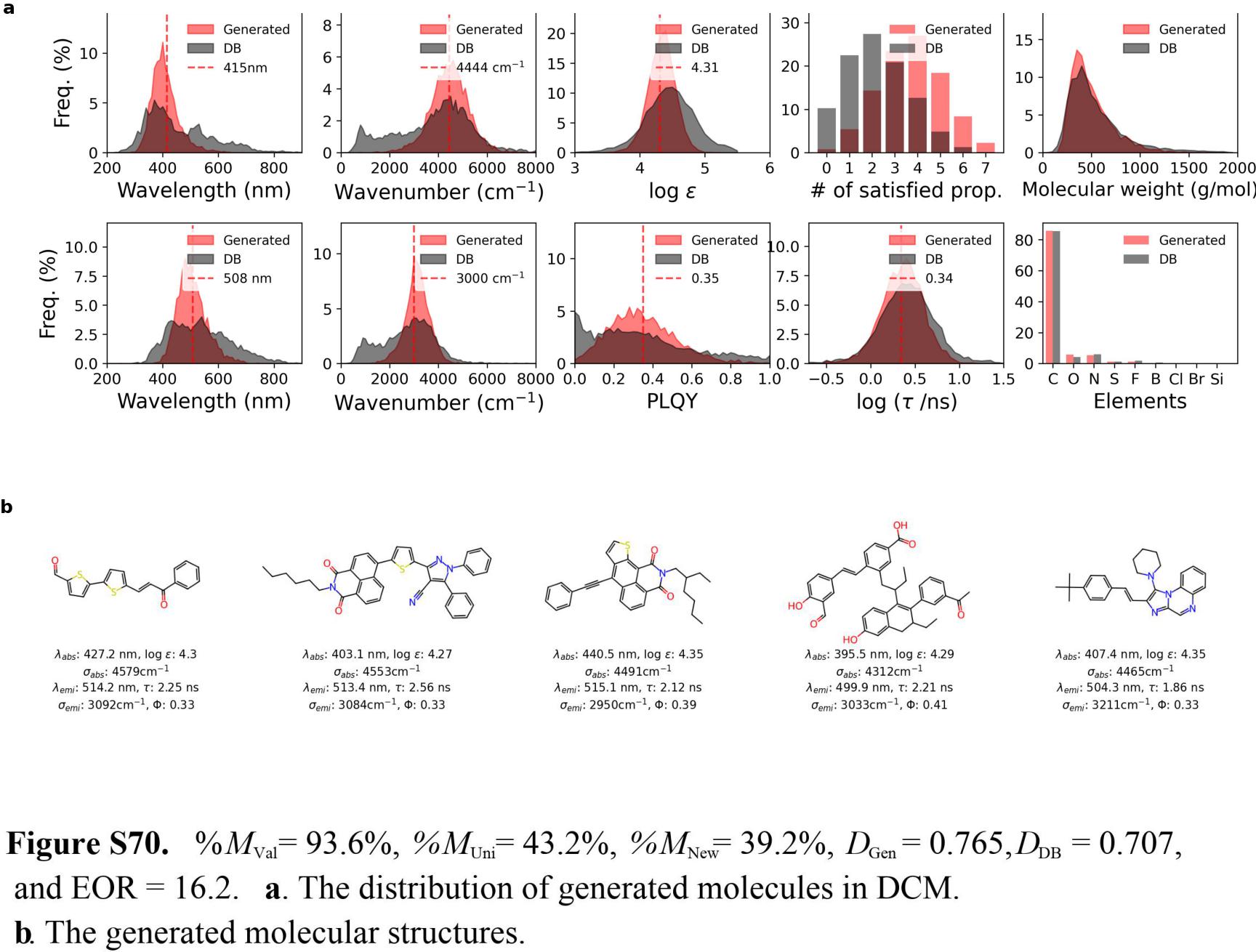

Supplement: Supplementary file 2 — oc4c00656_si_002.zip [file oc4c00656_si_002.zip › FigureS70.jpg]

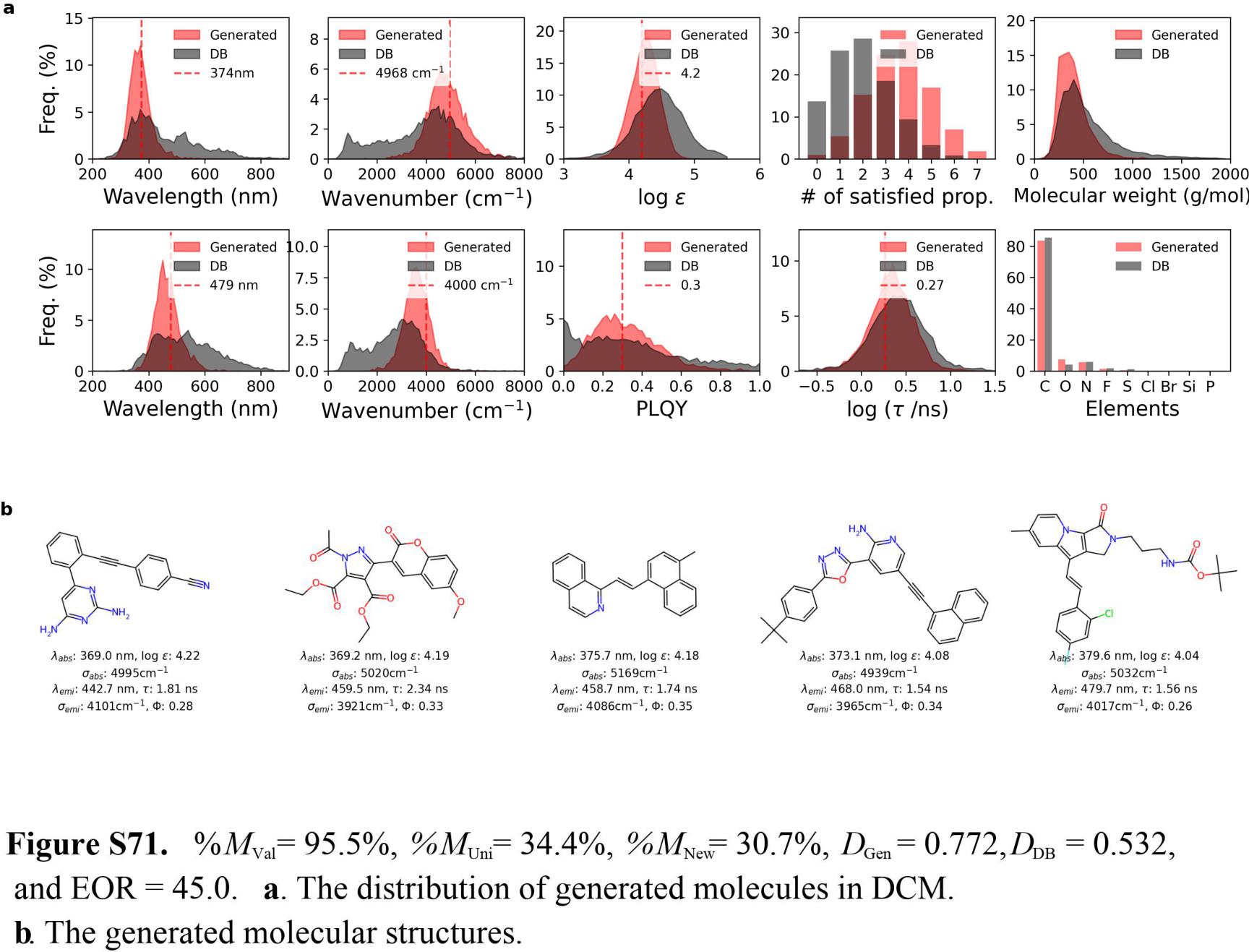

Supplement: Supplementary file 2 — oc4c00656_si_002.zip [file oc4c00656_si_002.zip › FigureS71.jpg]

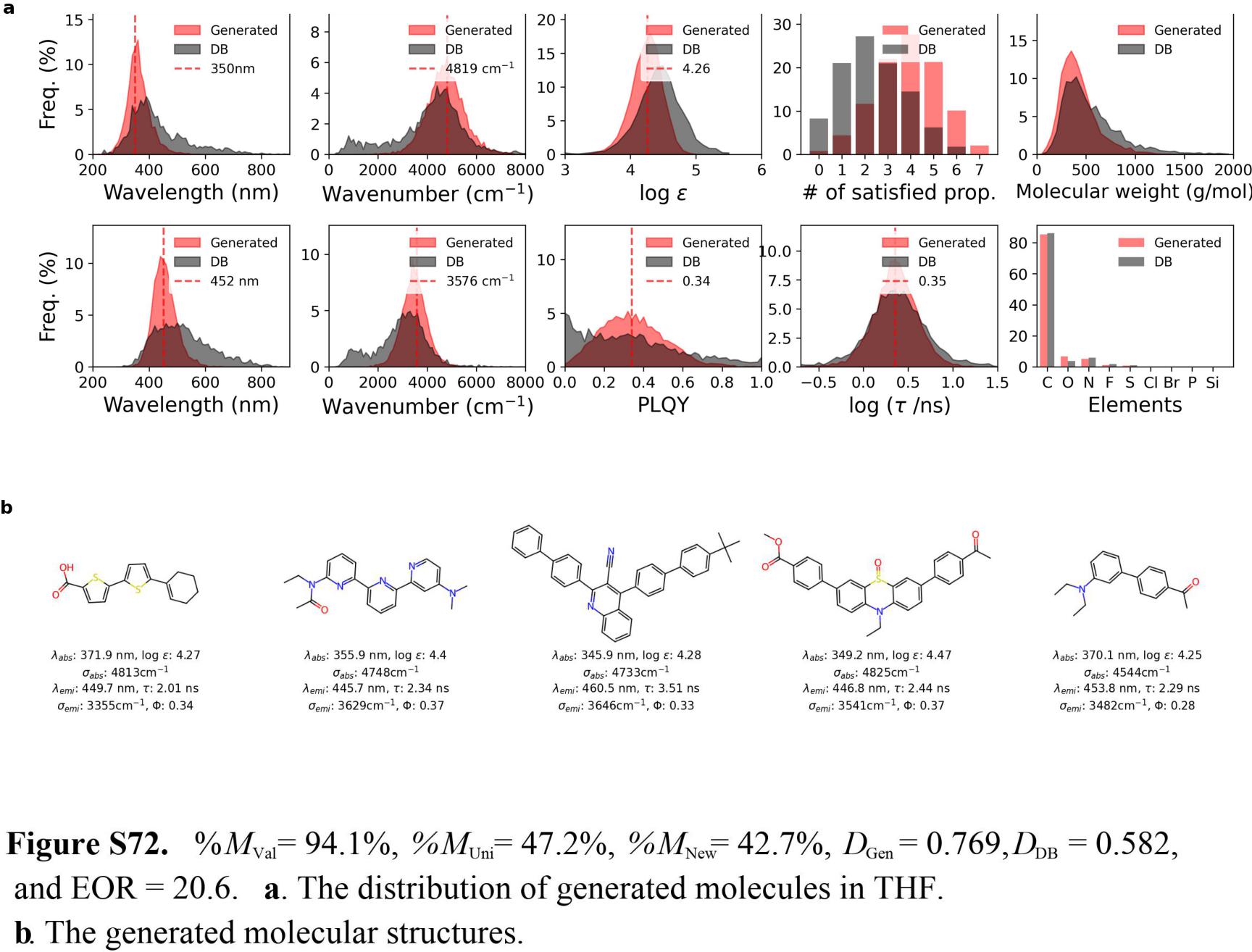

Supplement: Supplementary file 2 — oc4c00656_si_002.zip [file oc4c00656_si_002.zip › FigureS72.jpg]

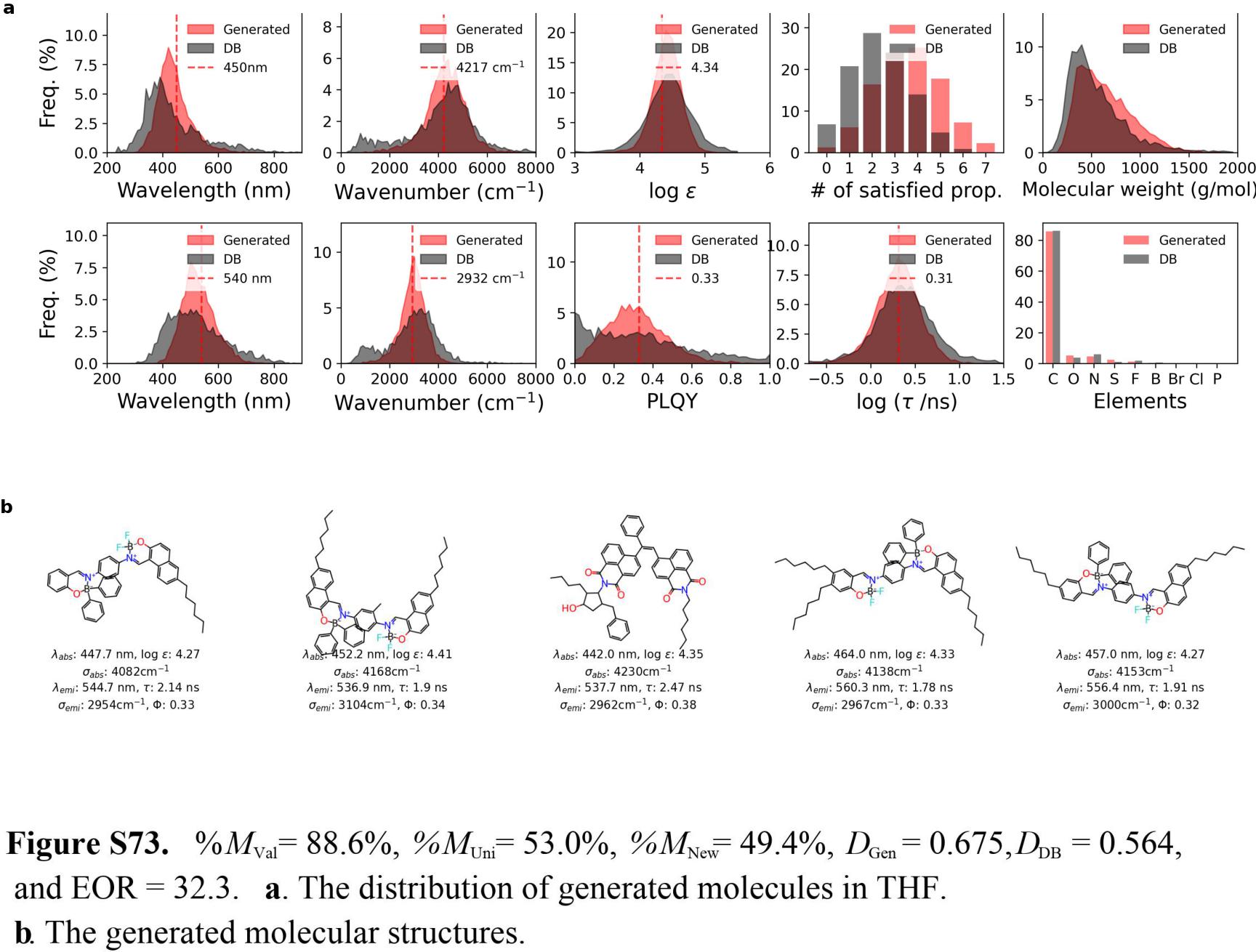

Supplement: Supplementary file 2 — oc4c00656_si_002.zip [file oc4c00656_si_002.zip › FigureS73.jpg]

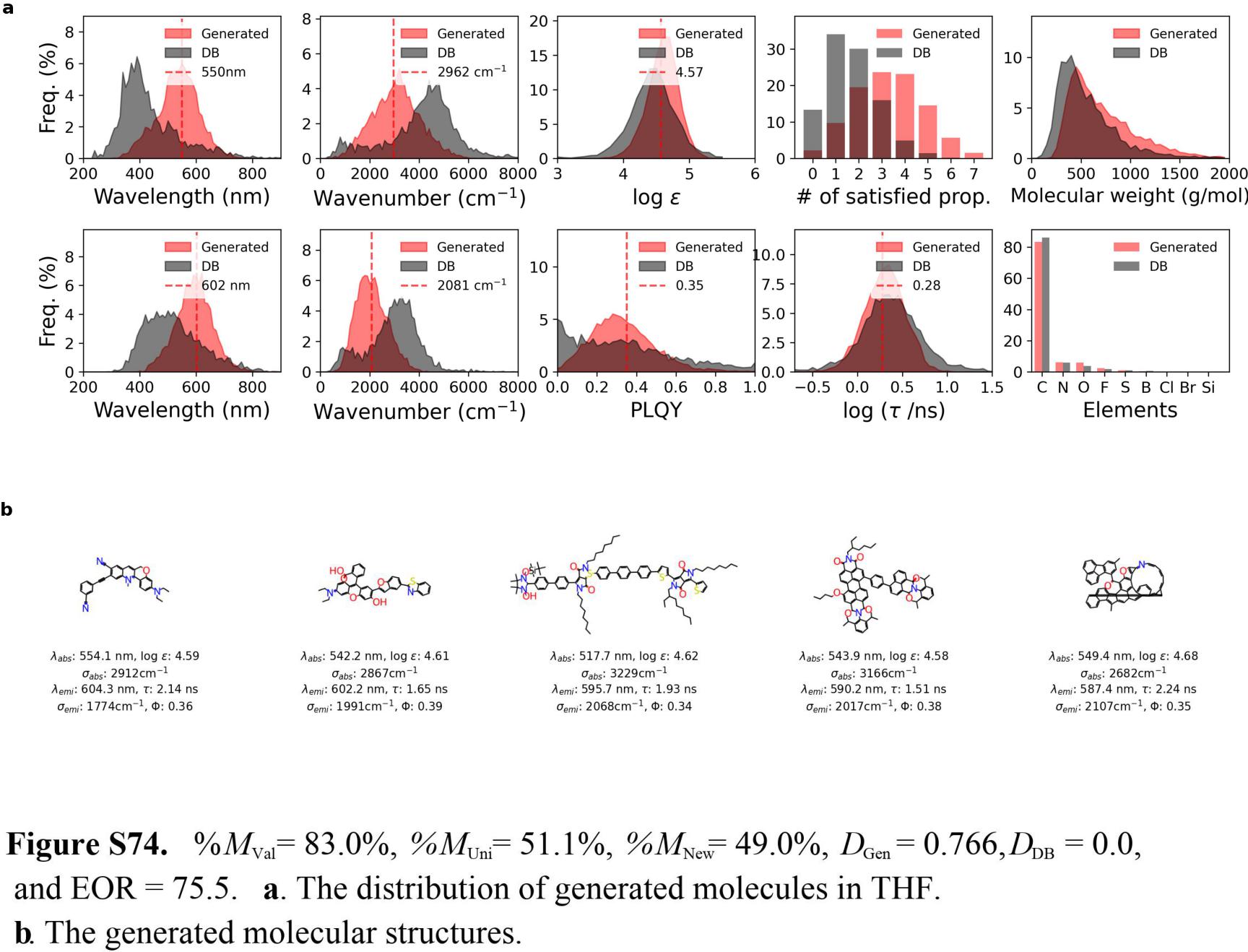

Supplement: Supplementary file 2 — oc4c00656_si_002.zip [file oc4c00656_si_002.zip › FigureS74.jpg]

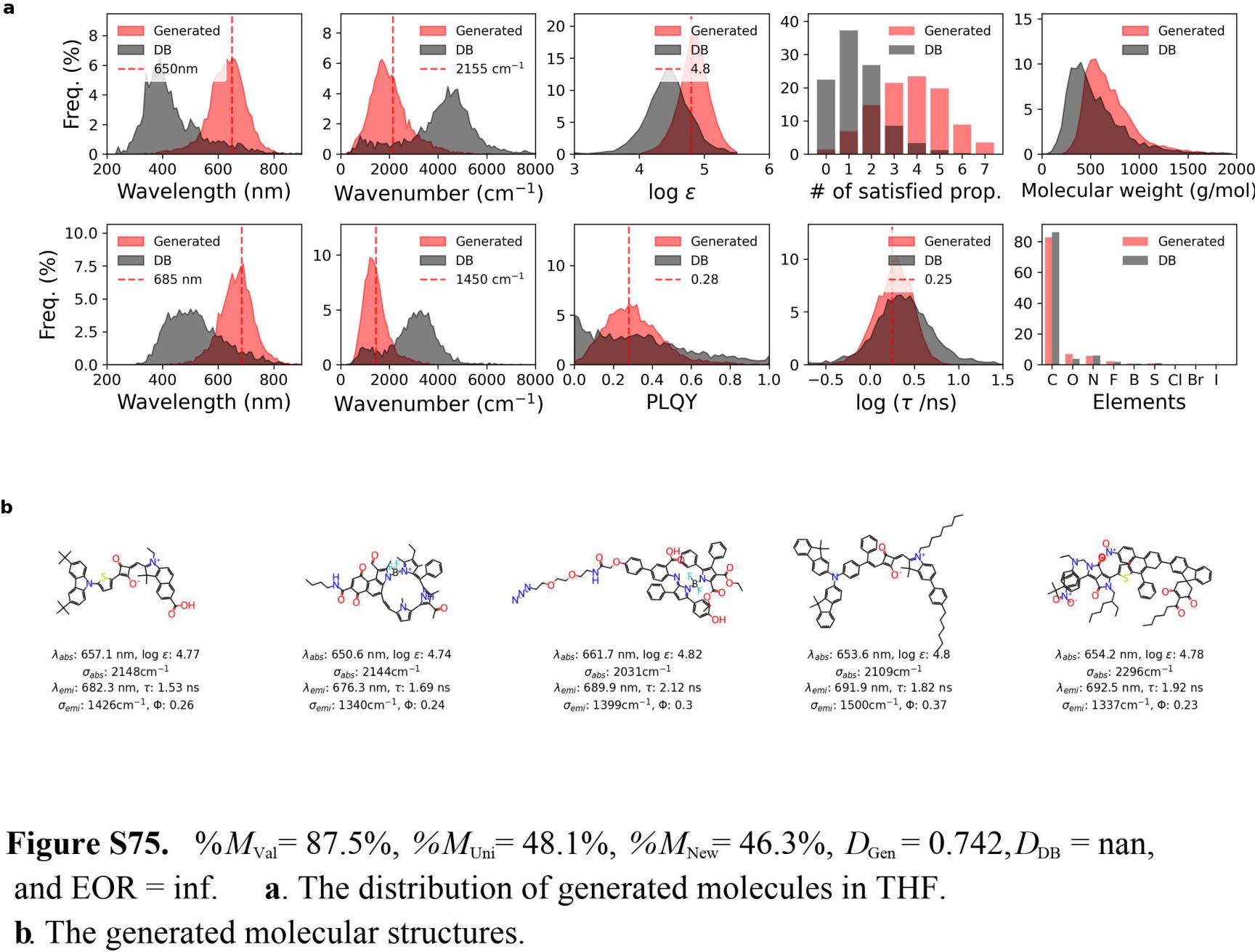

Supplement: Supplementary file 2 — oc4c00656_si_002.zip [file oc4c00656_si_002.zip › FigureS75.jpg]

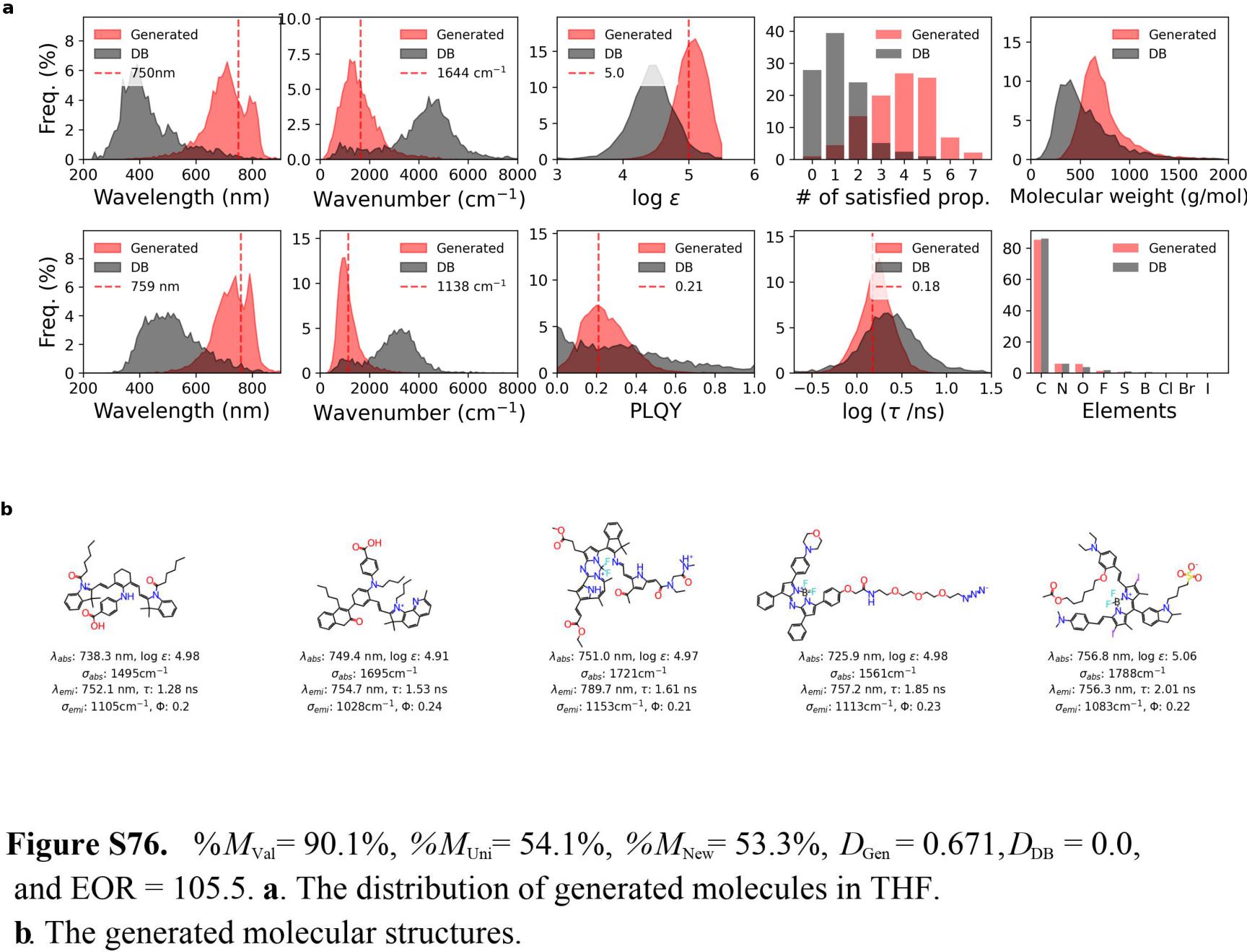

Supplement: Supplementary file 2 — oc4c00656_si_002.zip [file oc4c00656_si_002.zip › FigureS76.jpg]

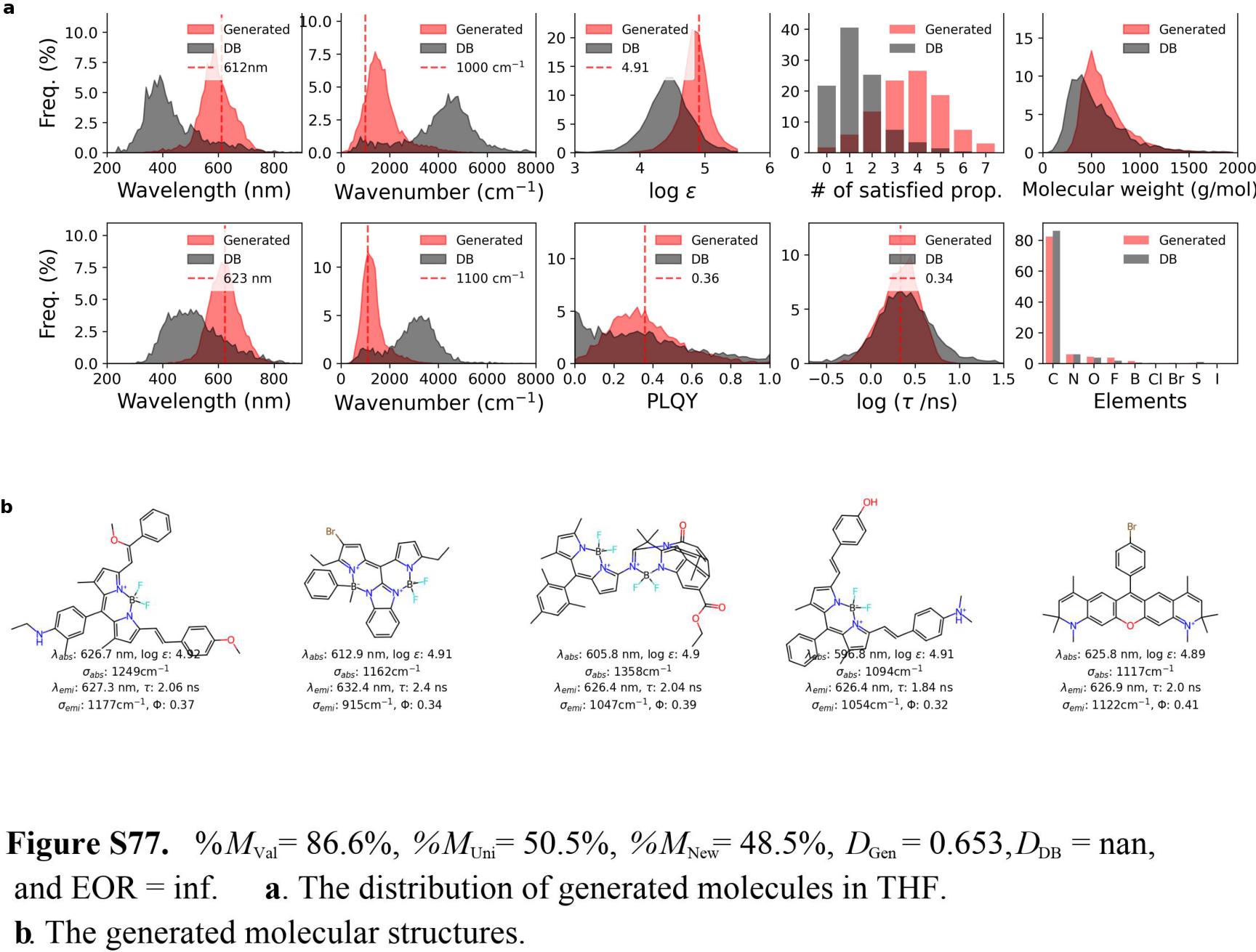

Supplement: Supplementary file 2 — oc4c00656_si_002.zip [file oc4c00656_si_002.zip › FigureS77.jpg]

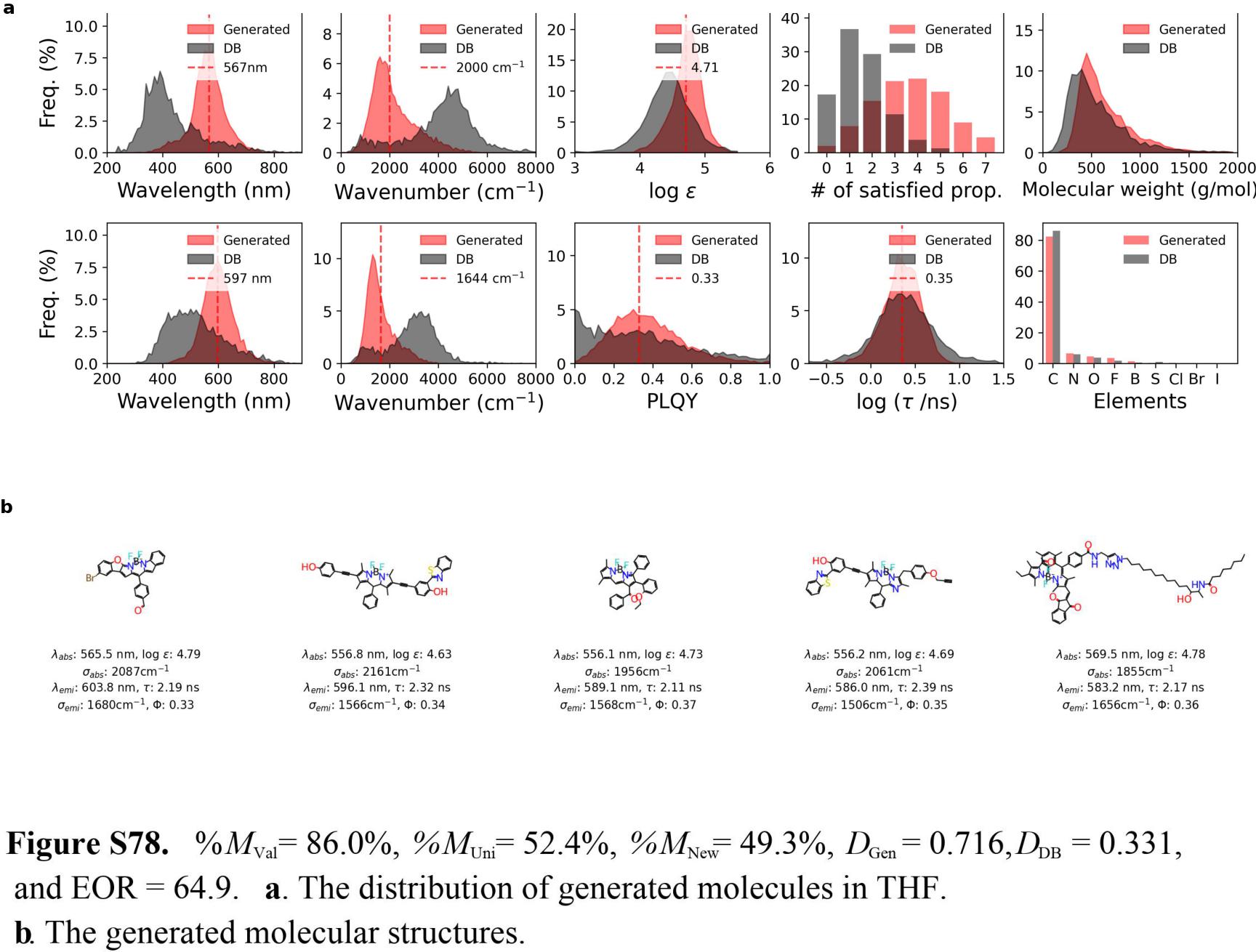

Supplement: Supplementary file 2 — oc4c00656_si_002.zip [file oc4c00656_si_002.zip › FigureS78.jpg]

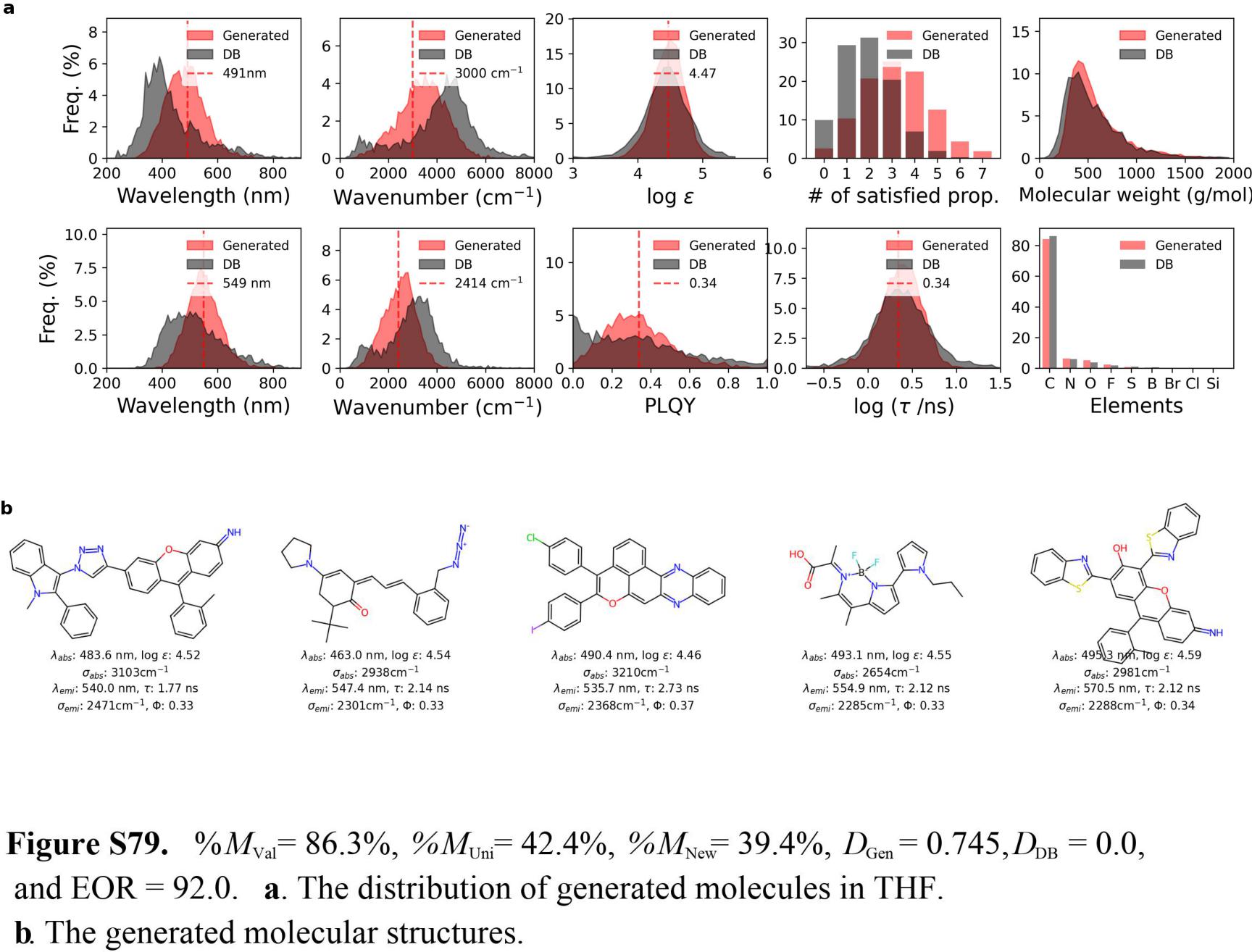

Supplement: Supplementary file 2 — oc4c00656_si_002.zip [file oc4c00656_si_002.zip › FigureS79.jpg]

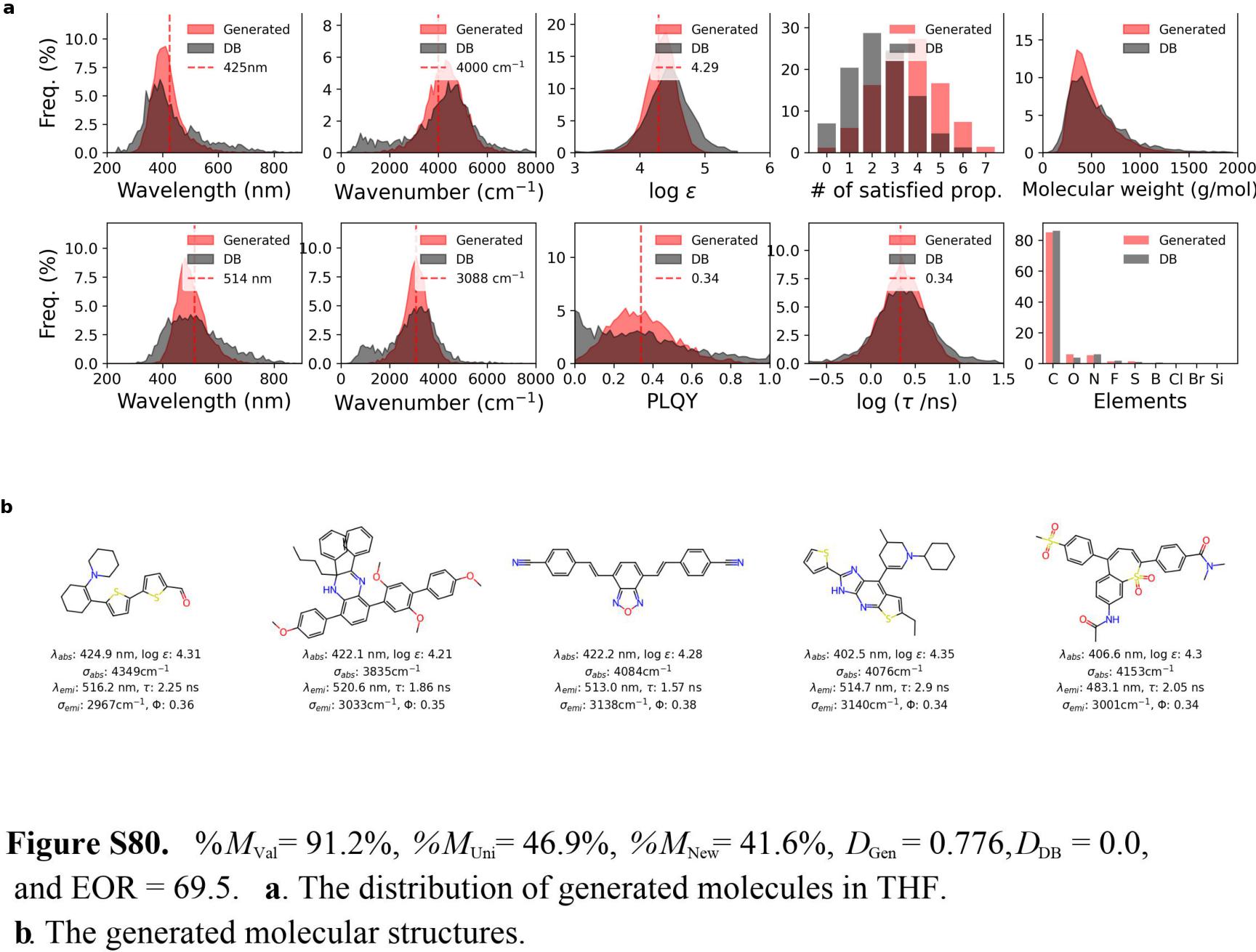

Supplement: Supplementary file 2 — oc4c00656_si_002.zip [file oc4c00656_si_002.zip › FigureS80.jpg]

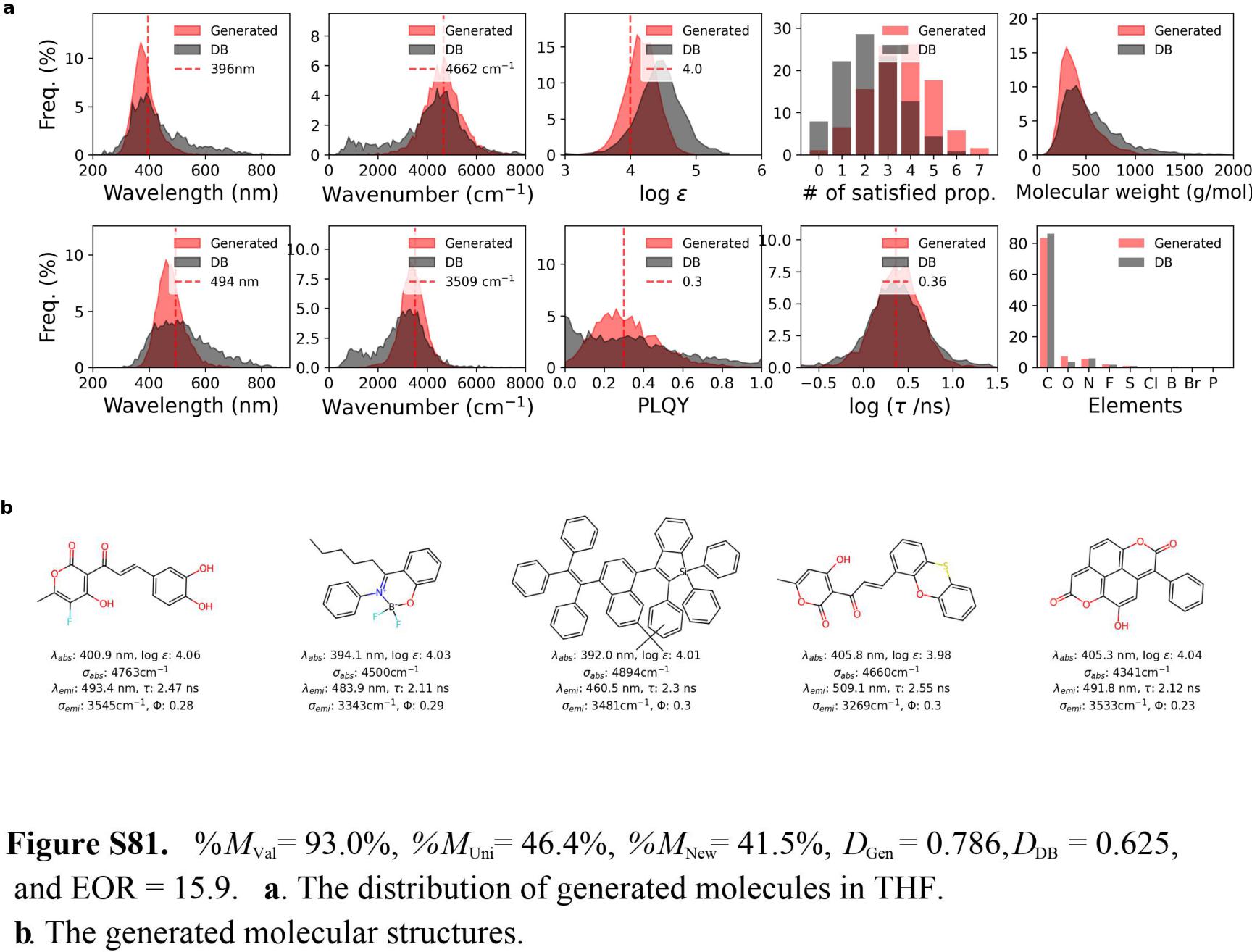

Supplement: Supplementary file 2 — oc4c00656_si_002.zip [file oc4c00656_si_002.zip › FigureS81.jpg]

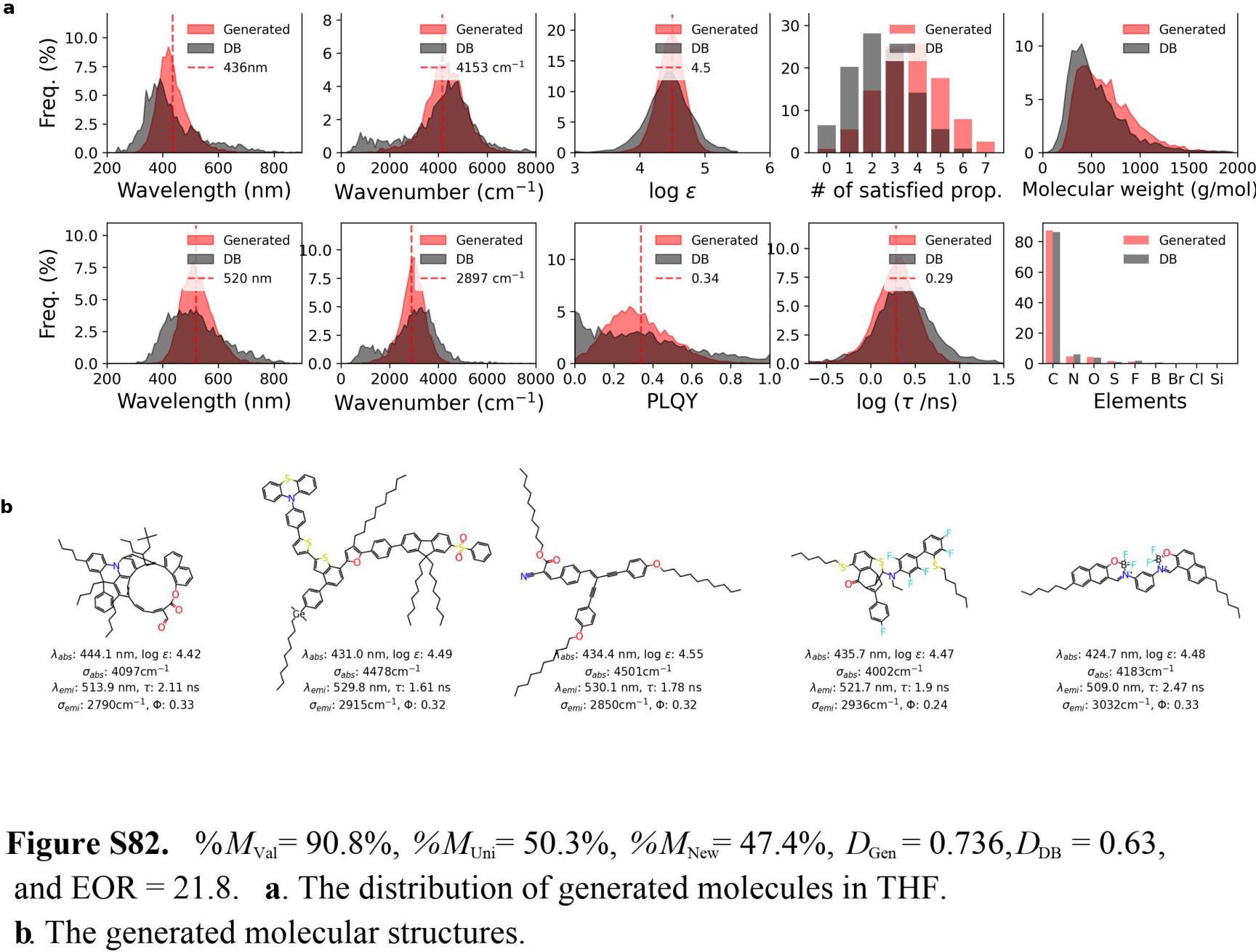

Supplement: Supplementary file 2 — oc4c00656_si_002.zip [file oc4c00656_si_002.zip › FigureS82.jpg]

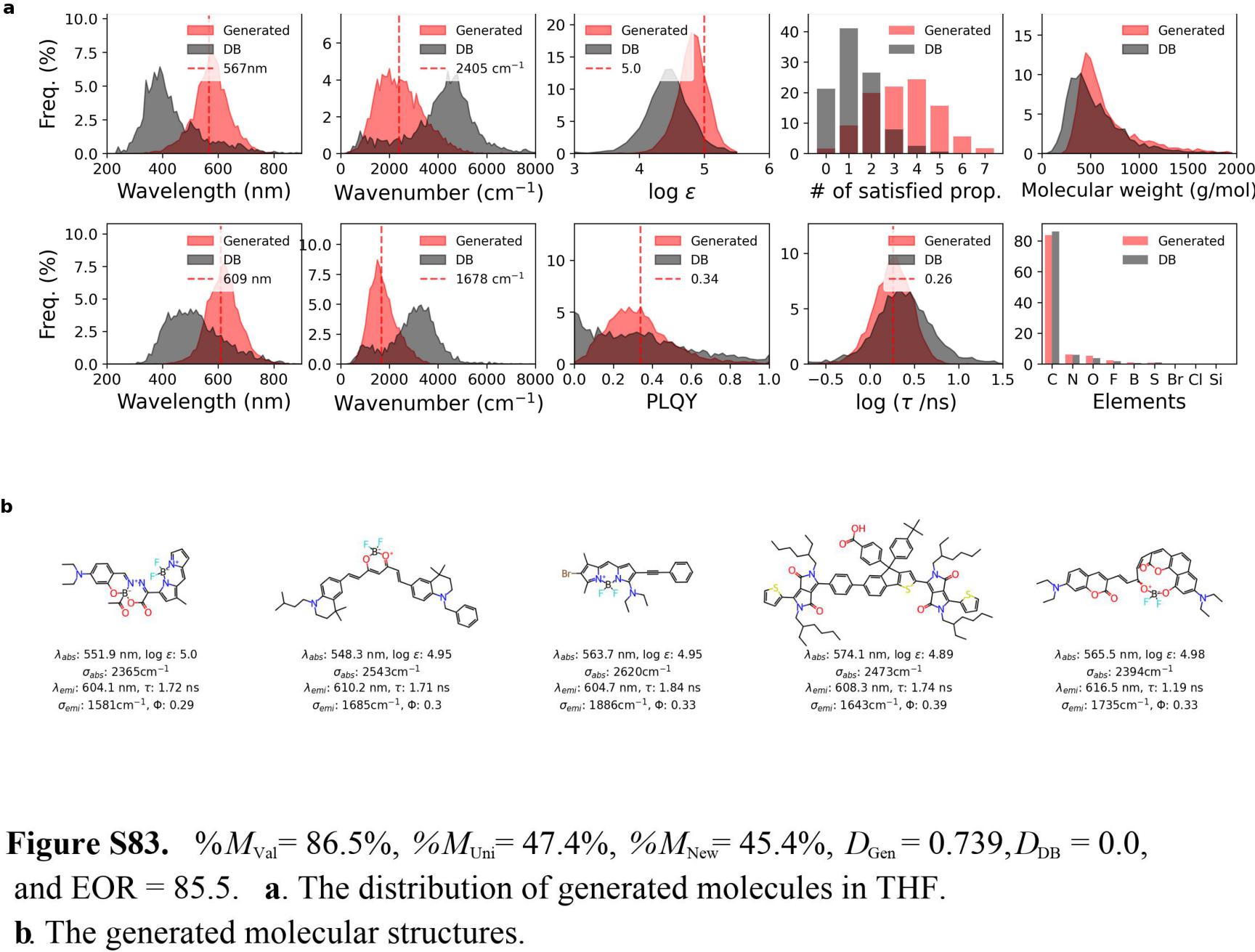

Supplement: Supplementary file 2 — oc4c00656_si_002.zip [file oc4c00656_si_002.zip › FigureS83.jpg]

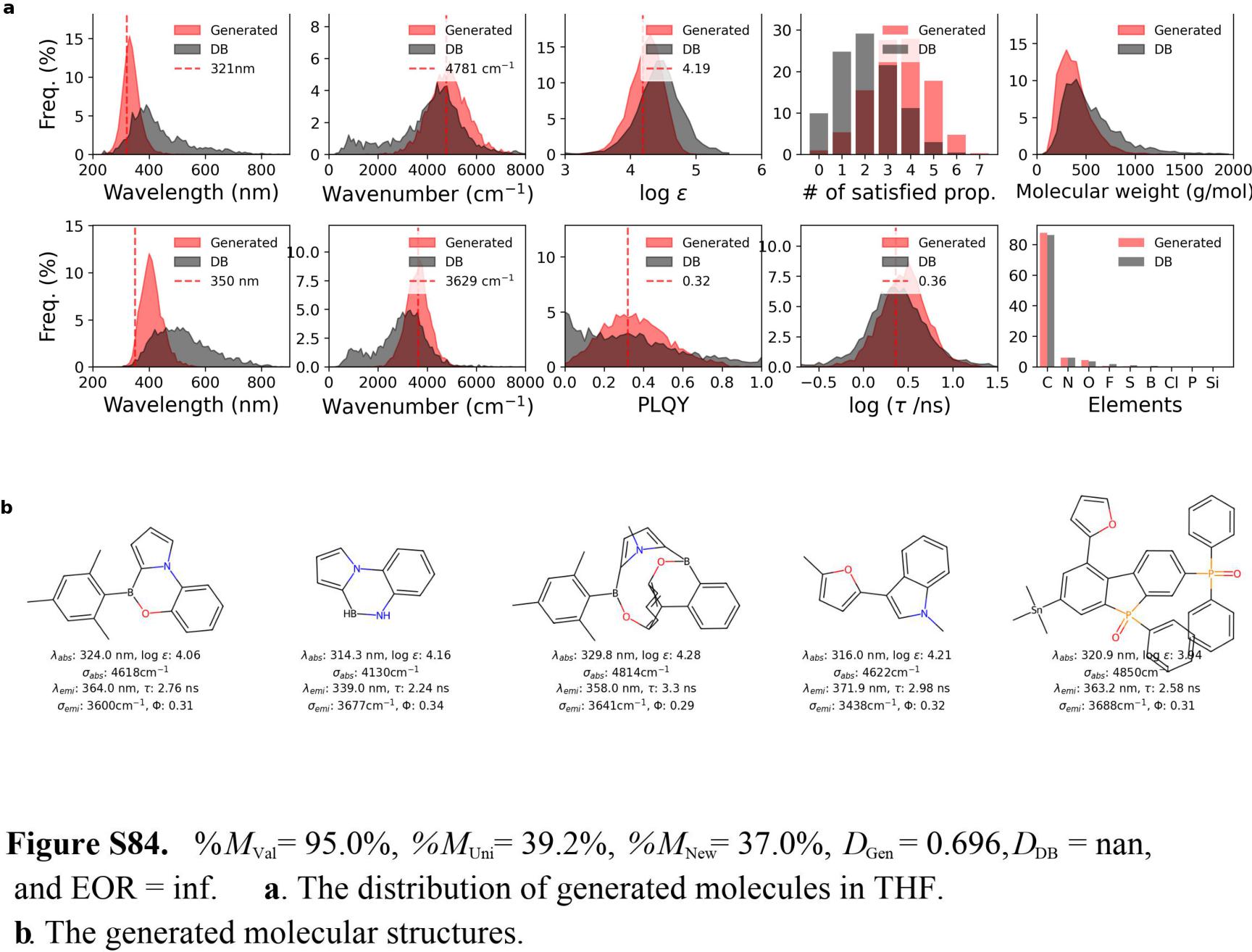

Supplement: Supplementary file 2 — oc4c00656_si_002.zip [file oc4c00656_si_002.zip › FigureS84.jpg]

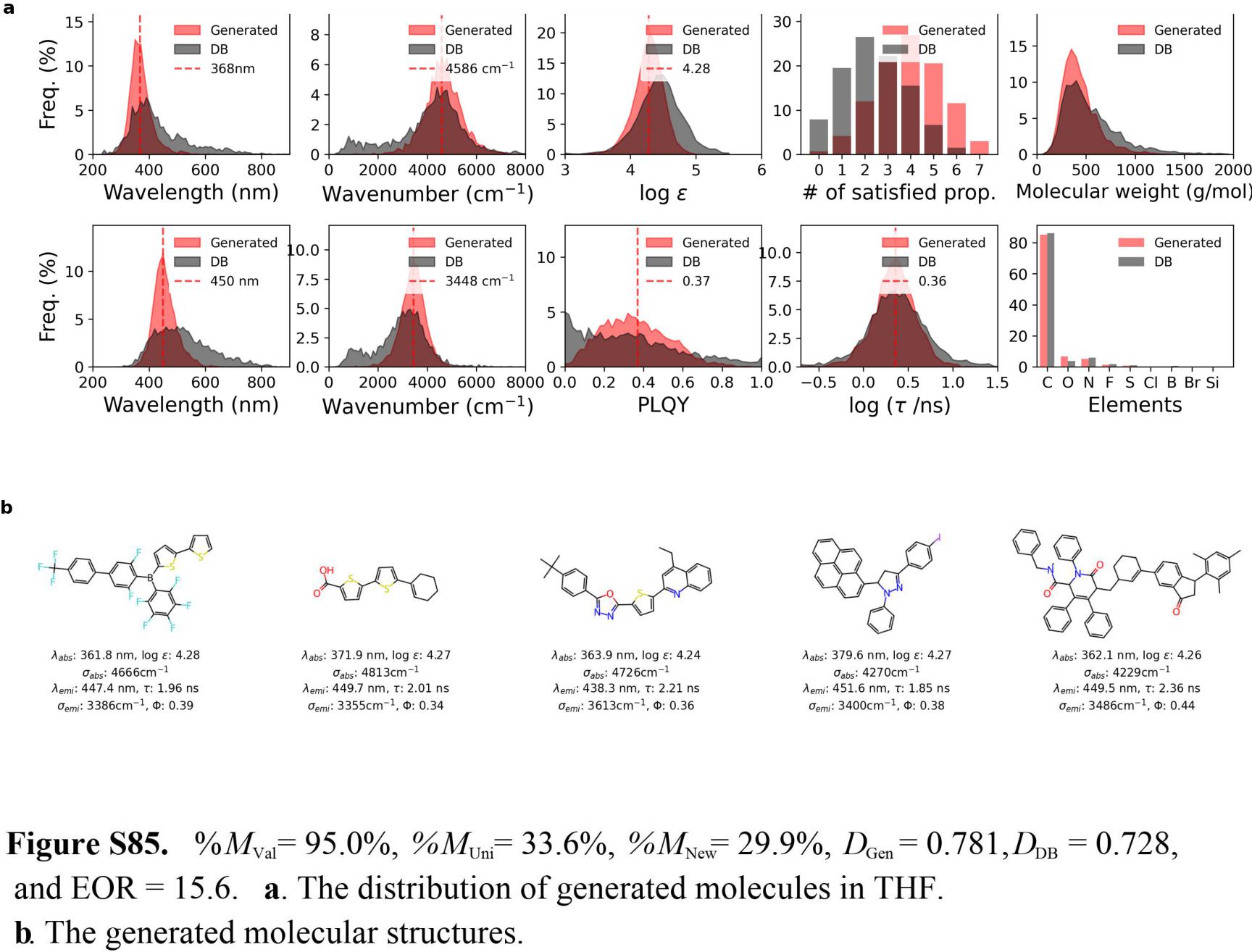

Supplement: Supplementary file 2 — oc4c00656_si_002.zip [file oc4c00656_si_002.zip › FigureS85.jpg]

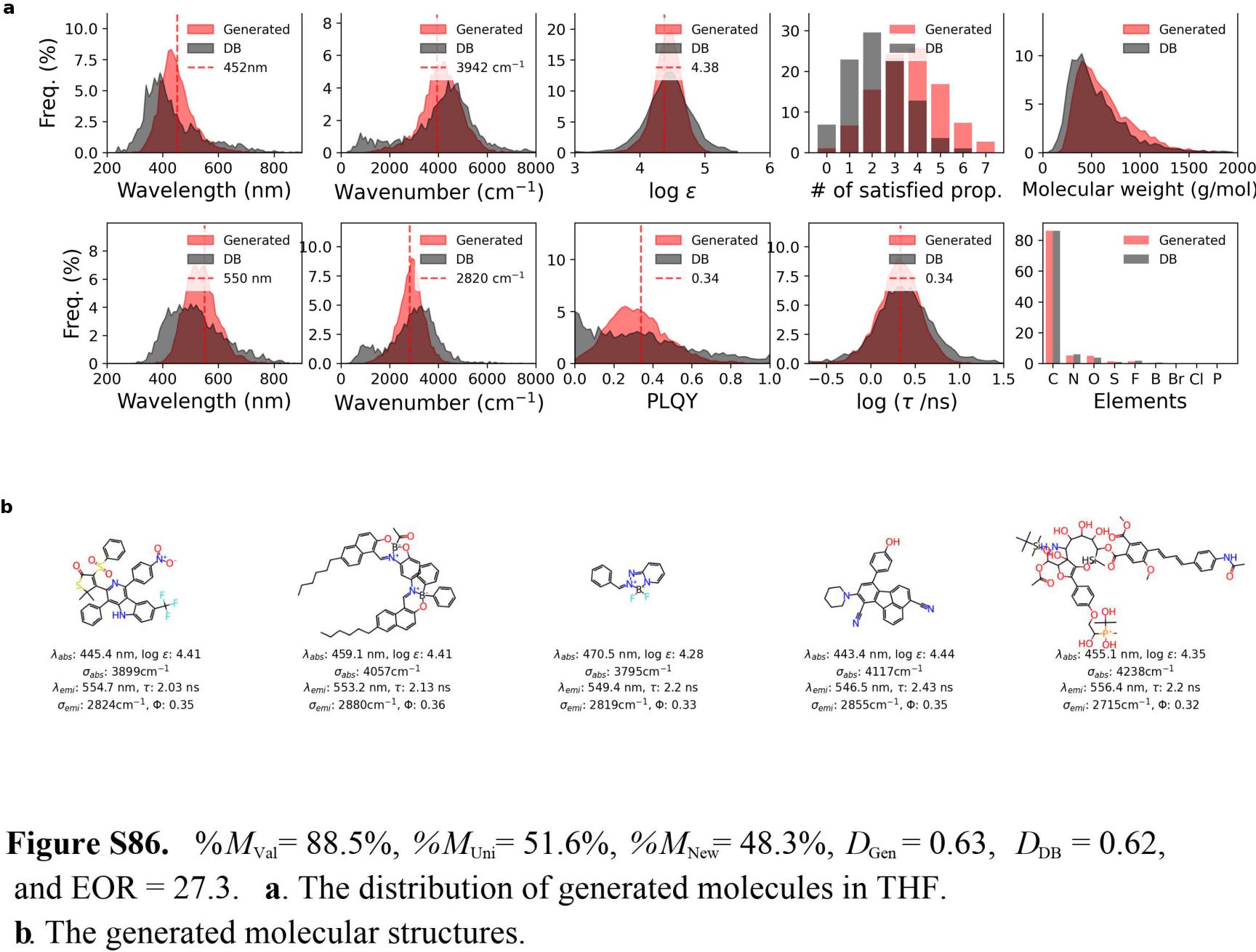

Supplement: Supplementary file 2 — oc4c00656_si_002.zip [file oc4c00656_si_002.zip › FigureS86.jpg]

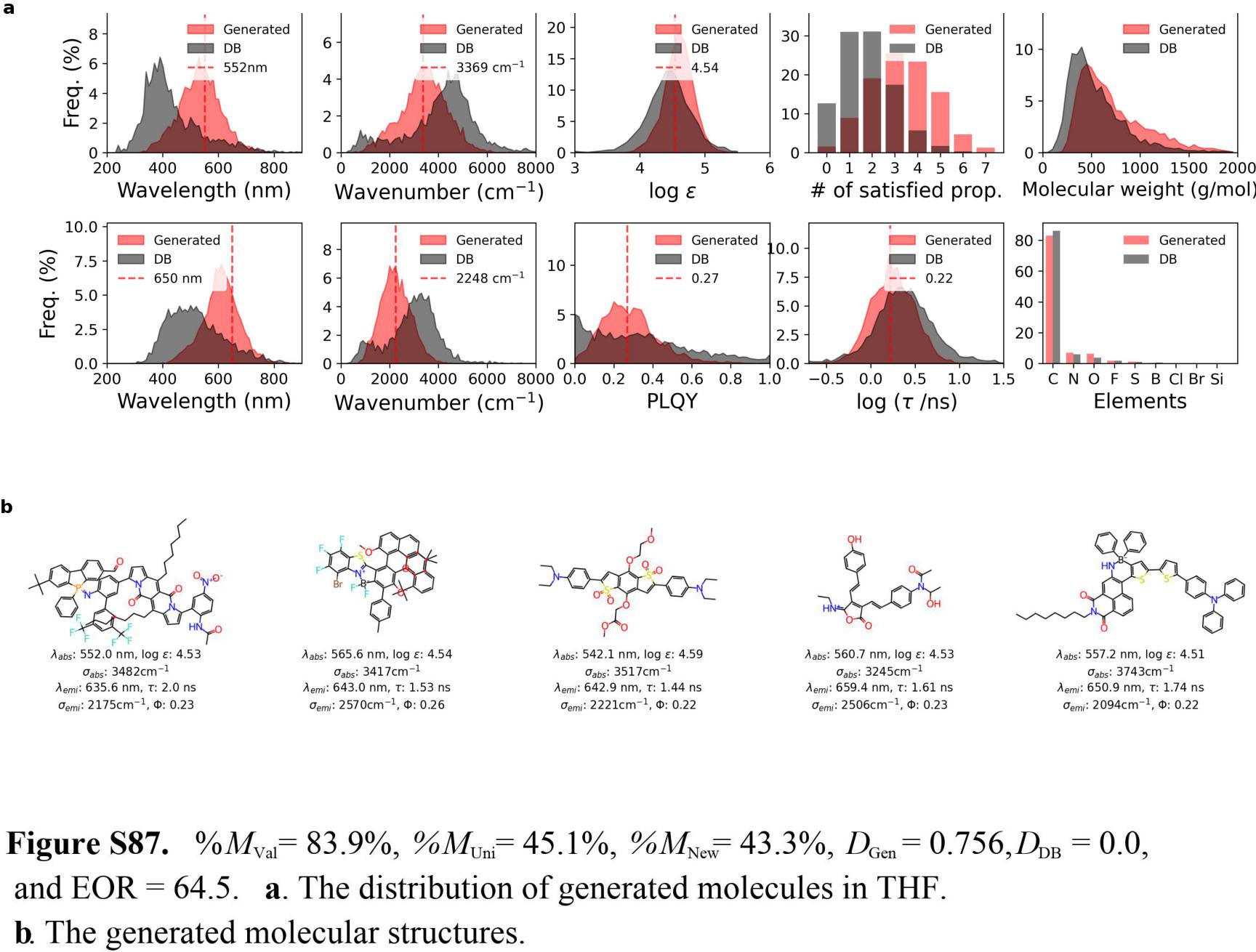

Supplement: Supplementary file 2 — oc4c00656_si_002.zip [file oc4c00656_si_002.zip › FigureS87.jpg]

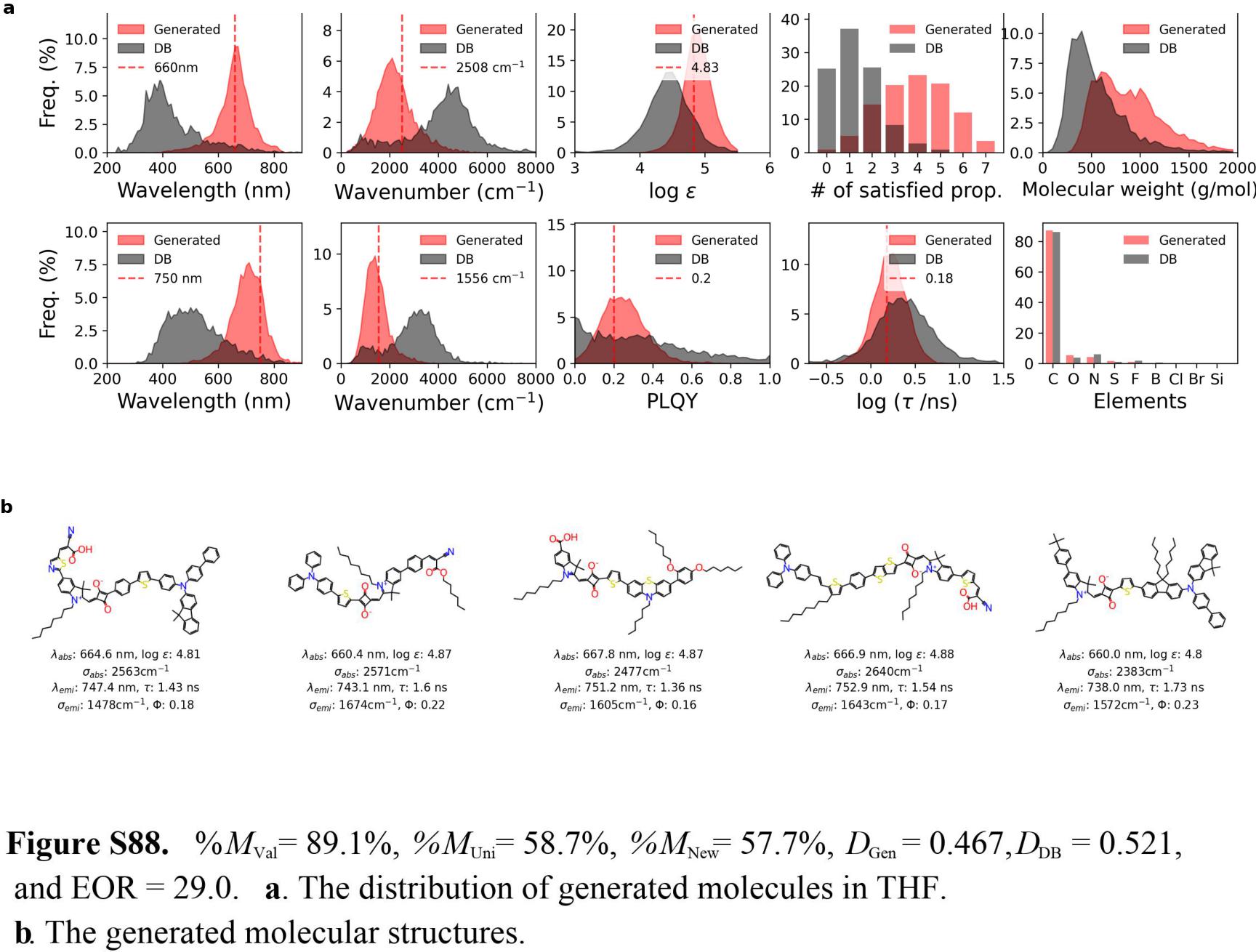

Supplement: Supplementary file 2 — oc4c00656_si_002.zip [file oc4c00656_si_002.zip › FigureS88.jpg]

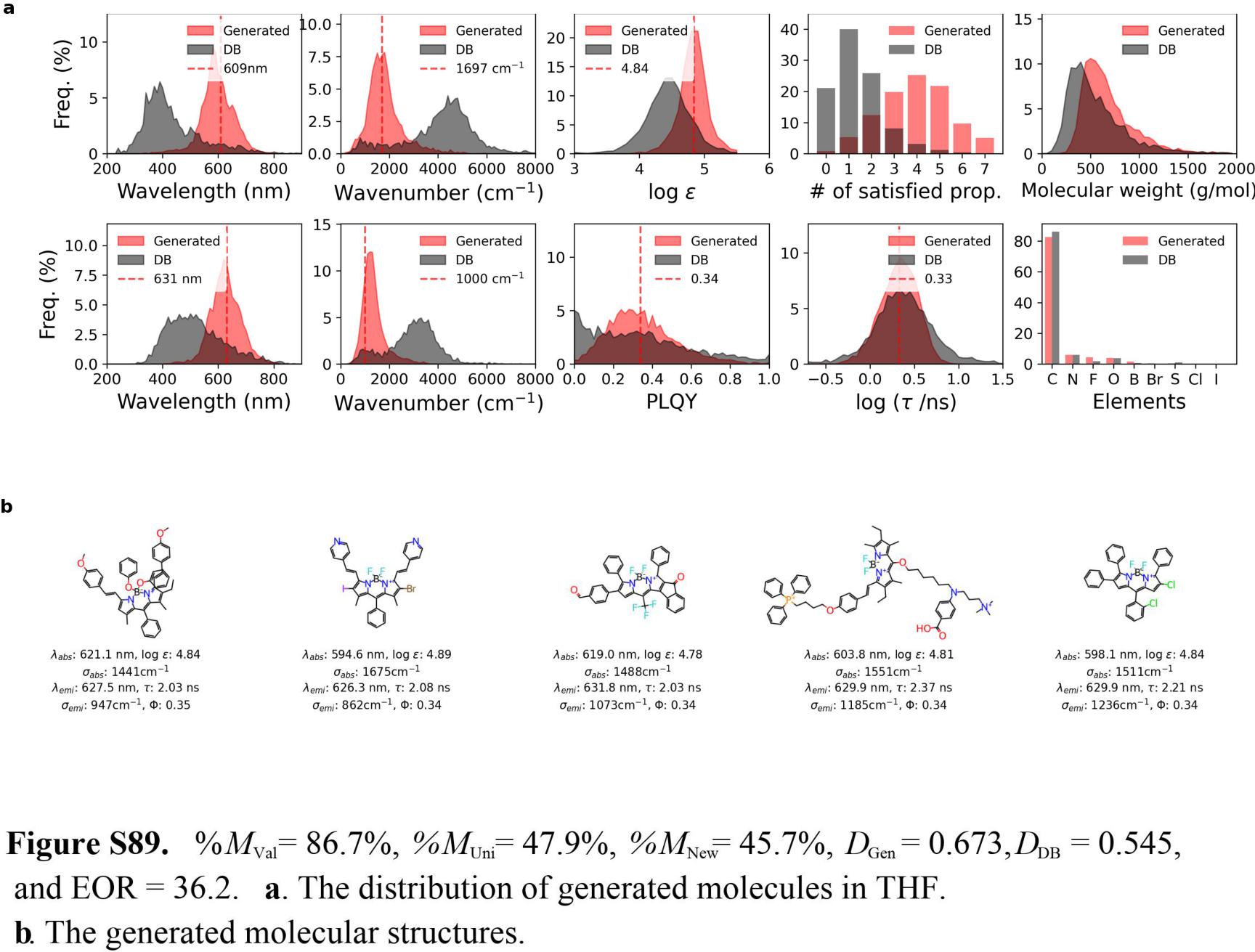

Supplement: Supplementary file 2 — oc4c00656_si_002.zip [file oc4c00656_si_002.zip › FigureS89.jpg]

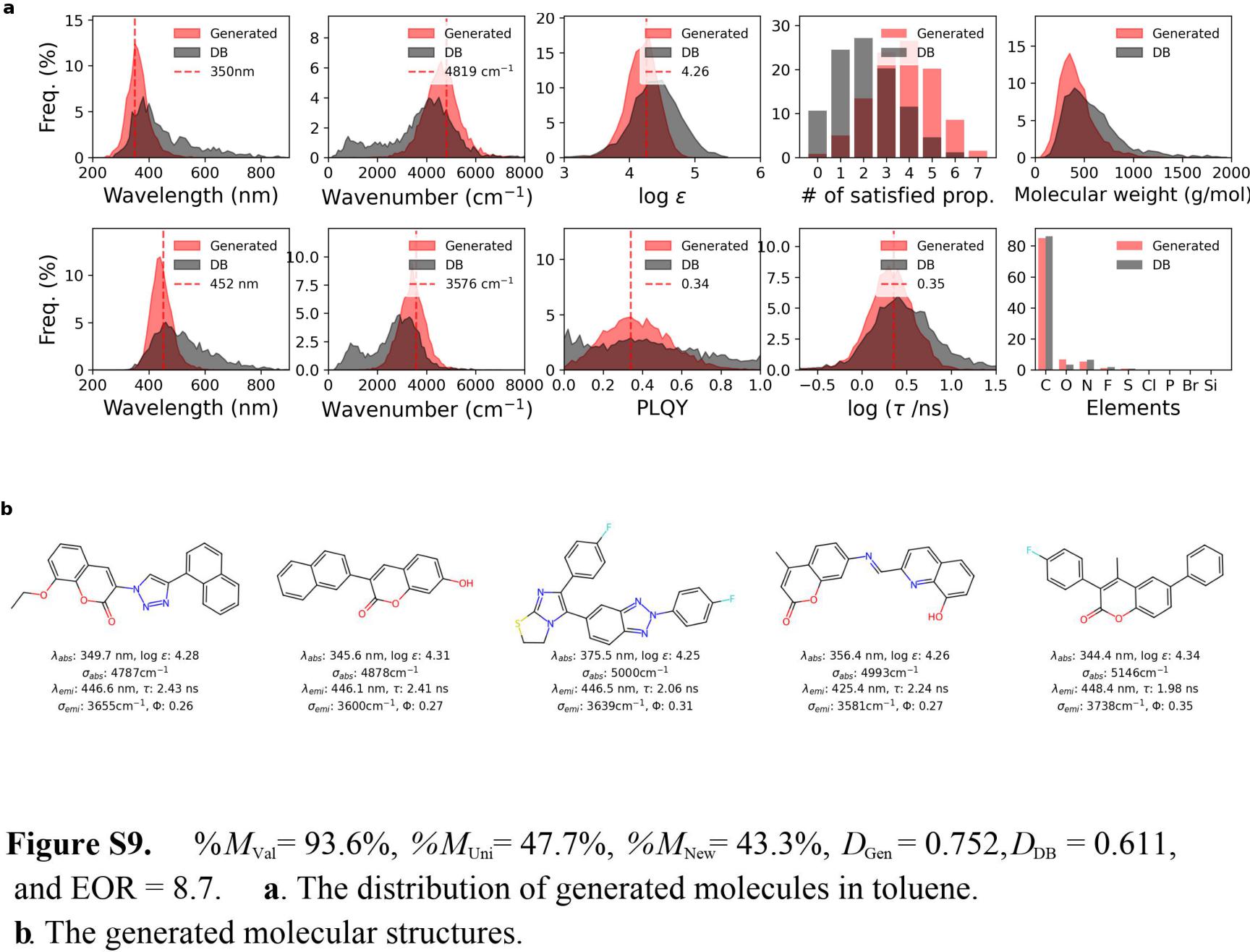

Supplement: Supplementary file 2 — oc4c00656_si_002.zip [file oc4c00656_si_002.zip › FigureS9.jpg]

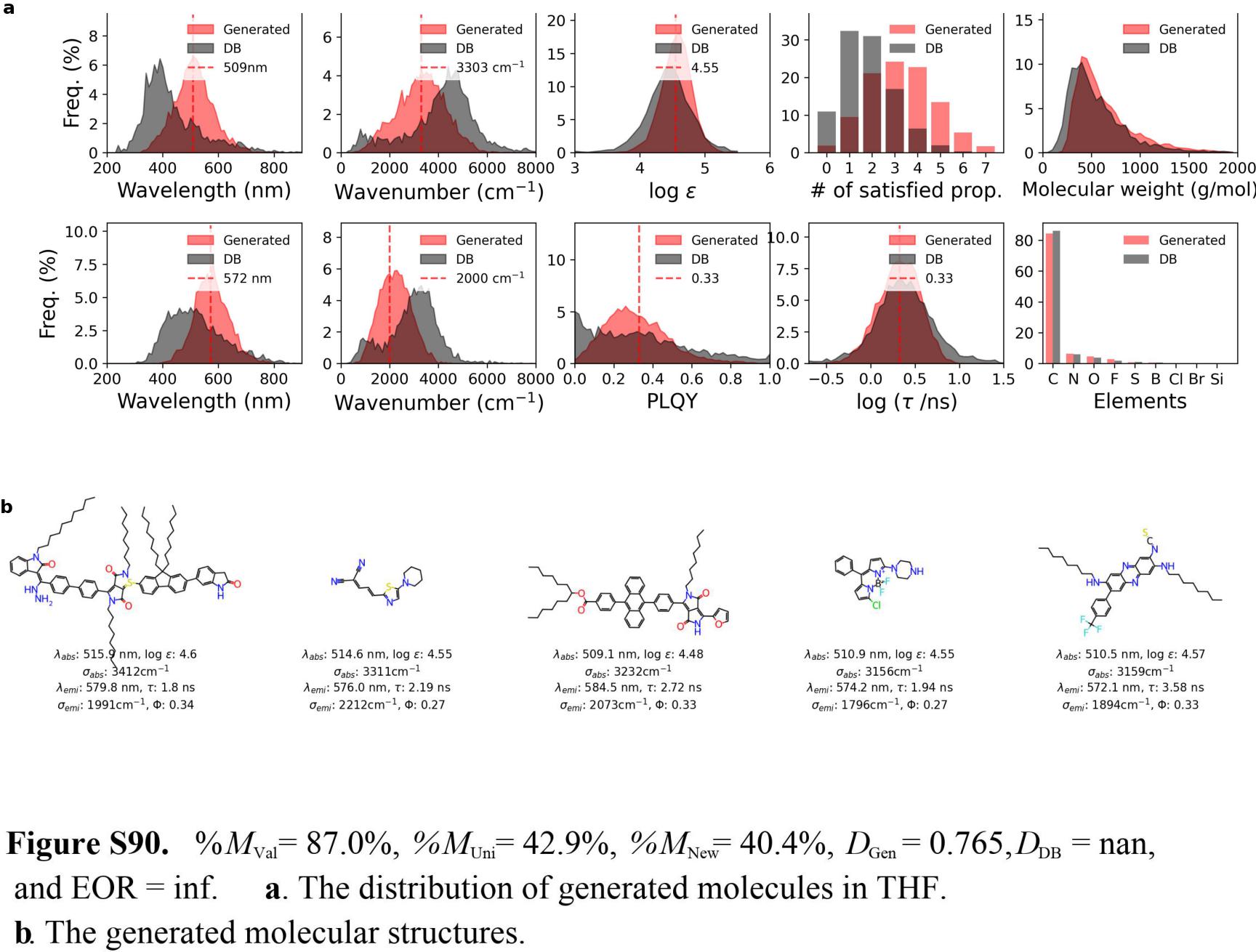

Supplement: Supplementary file 2 — oc4c00656_si_002.zip [file oc4c00656_si_002.zip › FigureS90.jpg]

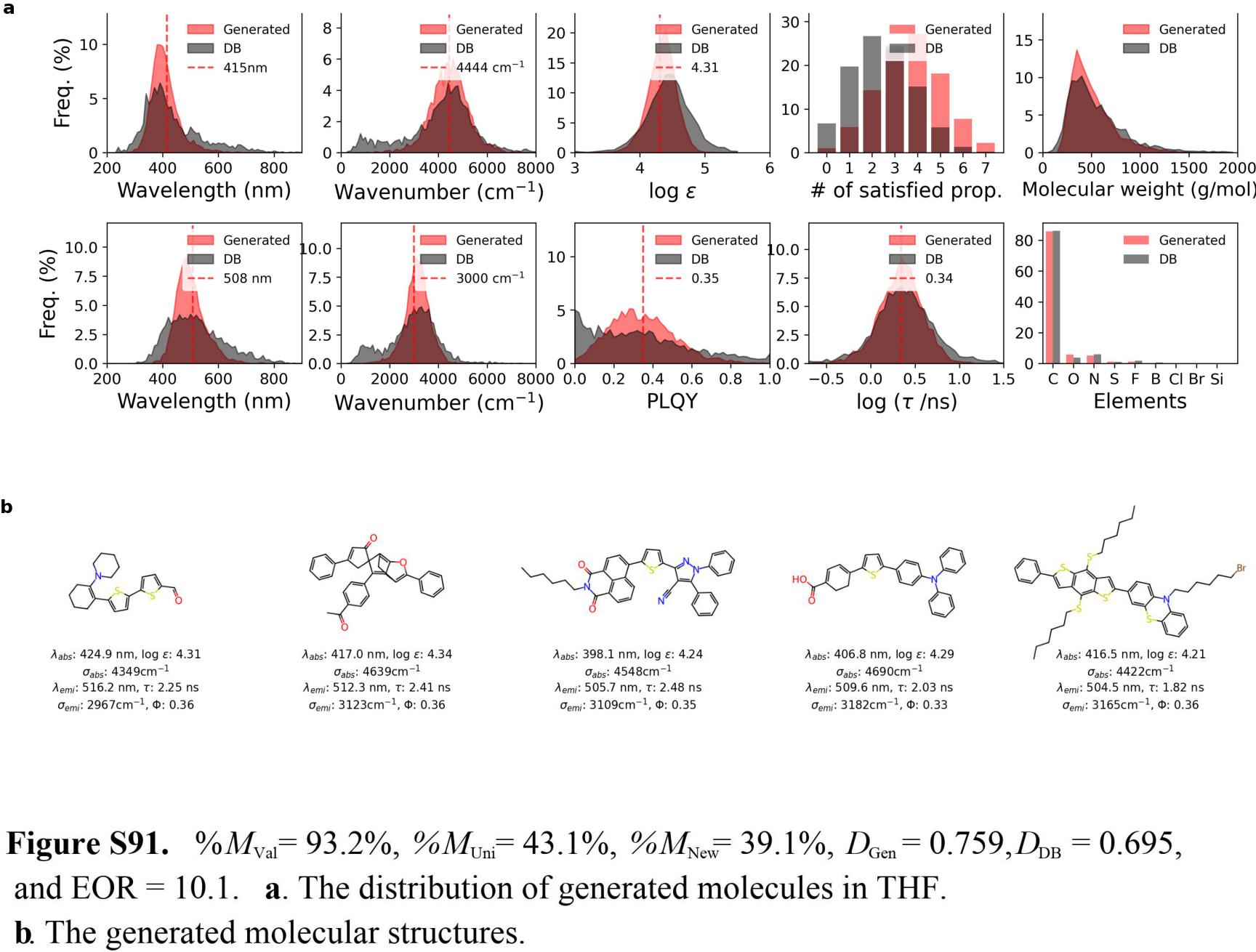

Supplement: Supplementary file 2 — oc4c00656_si_002.zip [file oc4c00656_si_002.zip › FigureS91.jpg]

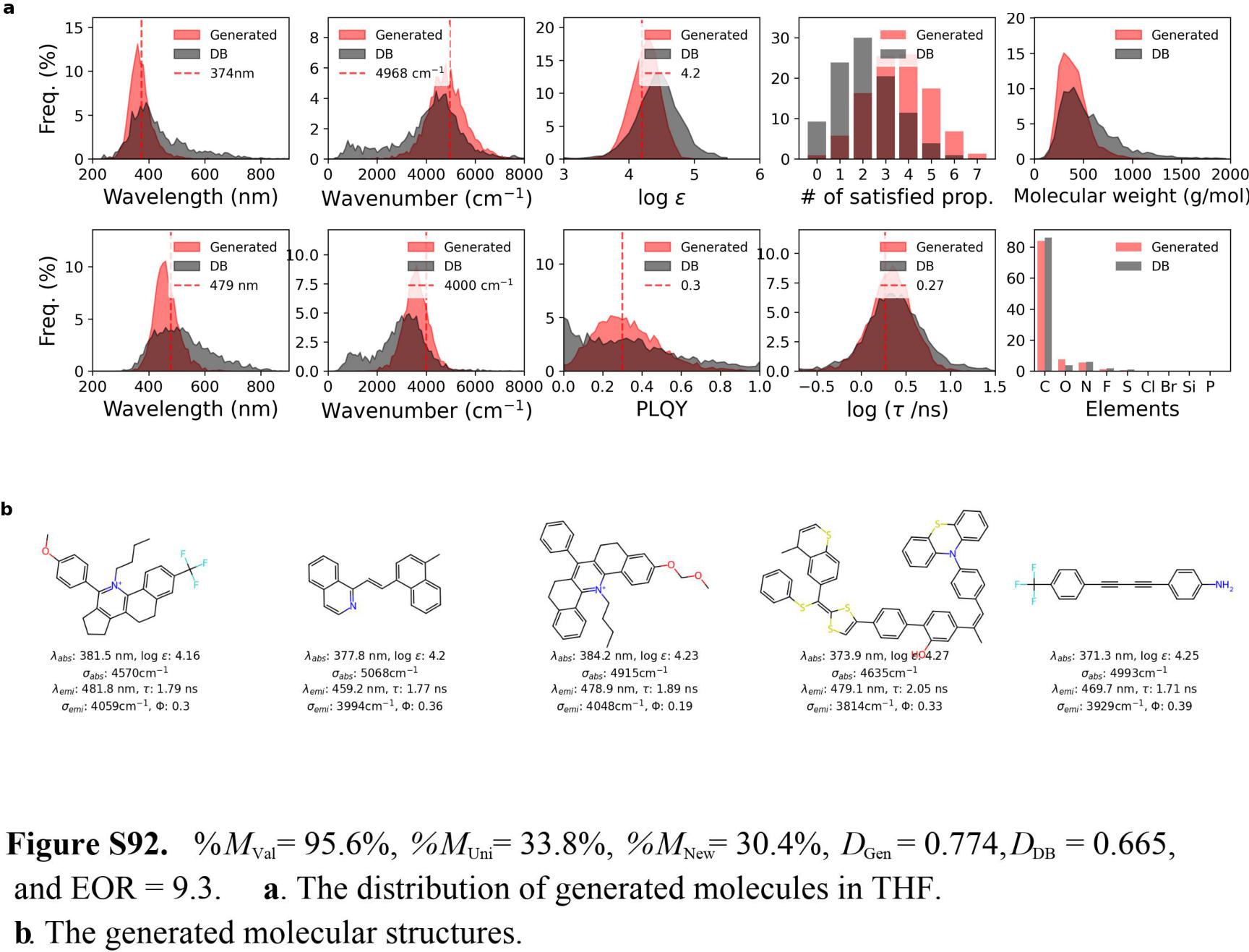

Supplement: Supplementary file 2 — oc4c00656_si_002.zip [file oc4c00656_si_002.zip › FigureS92.jpg]

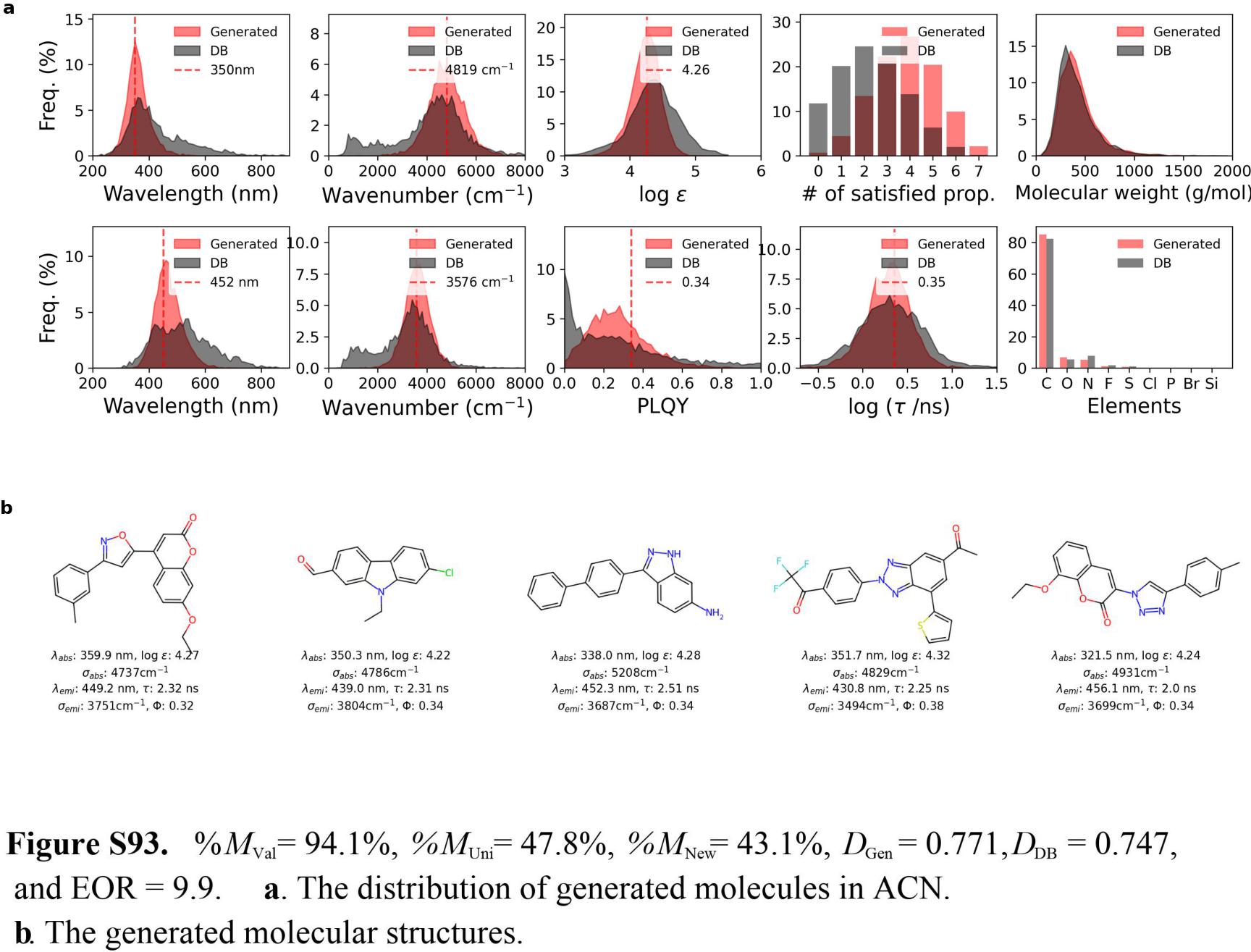

Supplement: Supplementary file 2 — oc4c00656_si_002.zip [file oc4c00656_si_002.zip › FigureS93.jpg]

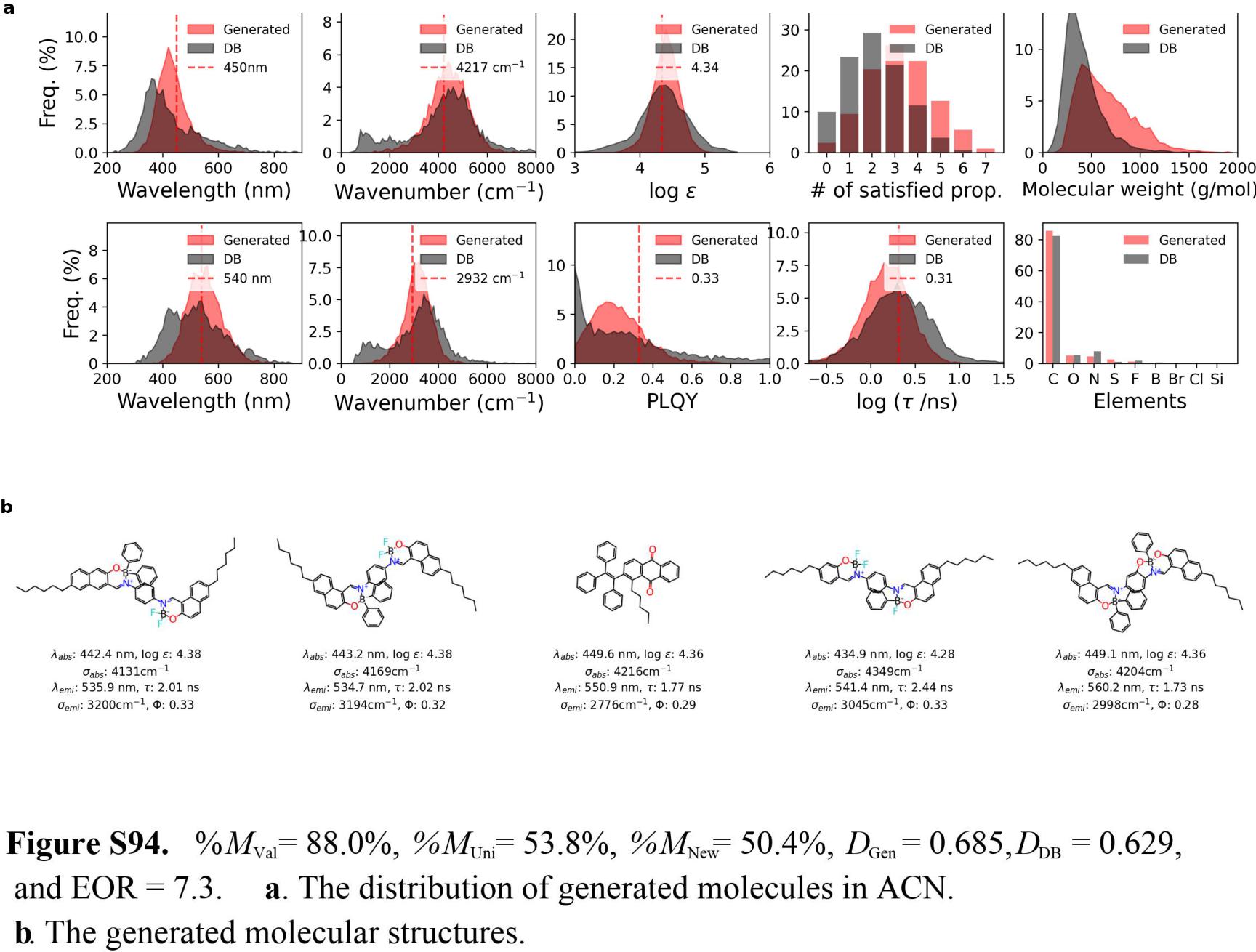

Supplement: Supplementary file 2 — oc4c00656_si_002.zip [file oc4c00656_si_002.zip › FigureS94.jpg]
