# Supplementary material for: Generative Deep Learning-Based Efficient Design of Organic Molecules with Tailored Properties
Source: ACS Cent Sci. 2024 Aug 30;11(2):219–27. doi: 10.1021/acscentsci.4c00656 (PMC11869130; doi:10.1021/acscentsci.4c00656)
Supplement: Supplementary file 3 — oc4c00656_si_003.pdf [file oc4c00656_si_003.pdf]

oc-2024-00656d.R1

Name: Peer Review Information for "Generative deep learning-based efficient design of organic molecules with tailored properties"

First Round of Reviewer Comments

Reviewer: 1

Comments to the Author
This manuscript presents a multi-objective design approach that stands out for its use of experimental data, including solvent information. This is a refreshing departure from the typical reliance on computational datasets and the common omission of solvent details. The paper is well-written and concise, making it a pleasure to read.

However, there are some major questions that need to be addressed. Firstly, is it possible to apply this methodology when access is limited to only a subset of the seven target properties, rather than all of them? This scenario is quite common during the design process. Secondly, could the authors provide a link to the source code and all datasets used? The web interface provided does not allow for the reproduction of the results presented in the paper, and the inference time is too slow for running more large-scale inferences. Lastly, could the authors include metrics on the percentage of valid generated molecules? Some seemingly infeasible structures were observed during tests on the web interface. This is understandable given the authors' use of atom-by-atom generation instead of fragment-based generation, but it would be helpful for readers to understand how this impacts validity.

There are also some minor questions and comments. On page 3, line 10, the authors mention that the field "has relied largely on expert knowledge and trial-and-error methods." Could the authors add citations for methods that rely on expert knowledge or trial-and-error? On page 5, line 35, the authors state that they "included only molecules in solutions and host films by excluding the molecules in solid states and gas phases from DBPred." What is the justification for adding these molecules to DBPred but excluding them from DBGen? Finally, on page 12, line 38, the authors compare their work with UV-visible absorption and emission spectra, and mention that the PLQY database is relatively noisy. Could the authors expand more on the noisiness of the PLQY database and the origins of its inaccuracy? These clarifications would greatly enhance the manuscript.

Reviewer: 2

Comments to the Author
The manuscript reports a supervised generative model that can generate molecules that satisfy multiple optical properties (e.g. absorption peak, bandwidth, extinction coefficient). While the distribution of final properties that are predicted is fairly broad, the model seems to be able to tune properties within the domain that is represented by the training dataset. Both generative models and optical property prediction models already exist and have previously been reported, but the proposed approach enables the simultaneous optimisation of multiple properties, which is an advance. The approach may be highly useful in identifying candidate molecules for specific optical/electronic properties. The manuscript leaves many technical questions open, which I raise below. These need to be addressed before the manuscript can be considered for publication.

- Access to the model is provided via a webpage, however, access to the trained model weights and biases is not provided. Are the datasets available? Is the model publicly available?

- The model is only trained for 200 epochs as it otherwise produces too few new molecules and mostly reproduces the molecules in the training dataset. The plots in Fig. S8 only show training up to 500 epochs, which are also relatively few. What happens if the model is trained substantially longer? At what percentage of M_new does it saturate? If that percentage is too low, then maybe changing the model architecture would be a better strategy than truncating the training early.

- The generative model was only trained on molecules in solutions and host films and molecules in solid state and gasphase were excluded. It should be explained why that was done. Does that mean that the remit of the model is restricted to molecules in solution? This should be explicitly stated

- Both predictive model and generative model have been tested on the same dataset. The generative model has been trained on labels partially generated with the property prediction model. The predictive model shows significantly worse prediction for the test set (see Fig. S2). What is the performance of Pred-DL for the generated molecules? Is it clear that the pred model still works for the generated molecules? Has this been validated? As it is trained on experimental data, how would one validate the prediction? The model might deteriorate and therefore guide the molecule generation into the wrong direction. I am not sure how the whole workflow can be validated. The manuscript doesn't show convincing evidence that the generated molecules actually have the properties that the predictive model assigns them.

- The step-by-step model building creates molecules stochastically to optimize the target labels. This includes the termination of the generation process. How will the model truncate the molecule? Will it truncate for certain when the property is within a certain threshold or according to a probability distribution conditioned by that property? Does that mean that the model is biased towards smaller molecules that satisfy the properties compared to larger ones? Consider the following scenario: If the construction starts from large scaffolds and there is also a probabilistic step of removing atoms, will the model create a similar distribution of molecules? If that is not the case, the current choice of molecule building steps creates an uncontrolled bias.

- What is the average size of generated molecules compared to the training database? What is the average elemental composition compared to the training database? The current analysis of structural similarity between generated molecules and training database seems insufficient to judge on the viability and robustness of the algorithm. The t-SNE analysis based on Morgan fingerprints does not present clear clustering and t-SNE can be misleading in the way how it is used here. In addition, Morgan fingerprints do not sufficiently discriminate molecules, which is evident from the fact that the t-SNE doesn't more clearly separate classes of molecules.

- The generative model is a supervised model and all generated molecules show properties in the same space as the original database. Is it possible to predict molecules with this approach with properties outside of the domain covered by the labels? Can it be assessed if both models are predicting within or outside of the training distribution? How would the user know when predictions cannot be trusted?

- It is stated that the “Gen-DL” model generates more hydrophilic molecules in water than in toluene. Why does it do that? This needs to be understood.

- Page 14, It is stated that the solvent effects are appropriately reflected by the Gen-DL model based on the Stokes shift trends. The arguments seem very handwavy.

Author's Response to Peer Review Comments:

2-August-2024

Journal: ACS Central Science
Manuscript ID: oc-2024-00656d
Title: "Generative deep learning-based efficient design of organic molecules with tailored properties"

Dear Editor,

Thank you for sending the reviewers' comments on our manuscript. The reviewers' comments are helpful to improve our manuscript. We have carefully reviewed all comments and suggestions. Below find a point-by-point response (in blue) to the comments (italic). The revised sections are indicated in blue in the manuscript.

Additionally, we read the original manuscript and SI and corrected typos.

With all these changes, we hope that the revised manuscript will be suitable for publication in *ACS Central Science*.

Sungnam Park, Ph.D.

Professor
Department of Chemistry, Korea University
Seoul, 02841, Korea

Reviewer(s)' Comments to Author:

Reviewer: 1

Recommendation: Publish in ACS Central Science after minor revisions noted.

Comments:
This manuscript presents a multi-objective design approach that stands out for its use of experimental data, including solvent information. This is a refreshing departure from the typical reliance on computational datasets and the common omission of solvent details. The paper is well-written and concise, making it a pleasure to read.

Author reply: We would like to thank the reviewer for pointing out the key findings of our manuscript and providing positive comments.

However, there are some major questions that need to be addressed. Firstly, is it possible to apply this methodology when access is limited to only a subset of the seven target properties, rather than all of them? This scenario is quite common during the design process.

Author reply: We would like to thank the reviewers for this comment. The current version of the Gen-DL model requires seven target optical properties as input parameters. As briefly mentioned in the manuscript, we found that some optical properties are correlated, but some are not (Figure S3). If researchers only want to use a few target optical properties (less than 7), the remaining optical properties can be easily selected from the correlation maps (Figure S3). In this way, researchers can use our Gen-DL model to solve a technical issue using target optical properties less than 7.

Our web application (http://deep4chem.korea.ac.kr/DeepMoleculeGen) has been modified to allow a few target optical properties (less than 7) to be used as input parameters and the remaining optical properties are selected from correlation maps.

Secondly, could the authors provide a link to the source code and all datasets used? The web interface provided does not allow for the reproduction of the results presented in the paper, and the inference time is too slow for running more large-scale inferences.

Author reply: We uploaded the source code and optimized parameters of the Gen-DL model to GitHub (https://github.com/spark8ku/DeepMoleculeGen) and made them available to the public. We are currently preparing a manuscript to submit our experimental database to *Scientific Data* for publication. For the time being, we will make our experimental database available for academic purposes upon request. In fact, our current Gen-DL model can be trained using the experimental database published in *Sci. Data,* **7**, 295 (2020) (https://www.nature.com/articles/s41597-020-00634-8). Since our experimental database (~20,000 datapoints) was published *Sci. Data,* **7**, 295 (2020), it has been significantly

expanded to ~ 80,000 datapoints.

We added the following section (Data Availability Statement) in the revised manuscript:

Data Availability Statement

Our Gen-DL model is publicly available as a web-based application (http://deep4chem.korea.ac.kr/DeepMoleculeGen). The codes and data for implementing our Gen-DL model are available at https://github.com/spark8ku/DeepMoleculeGen. The experimental database can be available from https://www.nature.com/articles/s41597-020-00634-8 or the corresponding author upon request for academic purposes only.

Lastly, could the authors include metrics on the percentage of valid generated molecules? Some seemingly infeasible structures were observed during tests on the web interface. This is understandable given the authors' use of atom-by-atom generation instead of fragment-based generation, but it would be helpful for readers to understand how this impacts validity.

Author reply: We would like to thank the reviewers for this comment. In fact, in Figure S9-113 in the original version, we have shown some metrics related to the molecules generated by the Gen-DL model. However, we found that we did not mention the metrics in the manuscript or Supporting Information. In the revised version, we included metrics such as the average validity, uniqueness, novelty, and success rate of all generated molecules as defined in the Supporting Information.

We added the following sentences on page 9 in the revised manuscript:

"The Gen-DL model was found to exhibit excellent performance in generating M_{TOP} in a given solvent (%M_{TOP} = ~4.4 %), as shown in Figure S9~S113. In addition, our Gen-DL model was found to generate molecules with 88.9 % validity, 47.1 % uniqueness, and 44.4 % novelty (see the SI for details)."

There are also some minor questions and comments.

On page 3, line 10, the authors mention that the field "has relied largely on expert knowledge and trial-and-error methods." Could the authors add citations for methods that rely on expert knowledge or trial-and-error?

Author reply: This statement is made to refer to the common practice of developing new molecules in chemistry and materials science. For example, in many research areas such as OLEDs, OPVs, PDTs and so on, new molecules with target properties are designed by experts

(researchers) based on their knowledge and experience, designed molecular structures are synthesized, and their properties are characterized. Such a development process (design, synthesis, and characterization) is repeated until the target properties of the final molecule are satisfied as shown in Figure below,

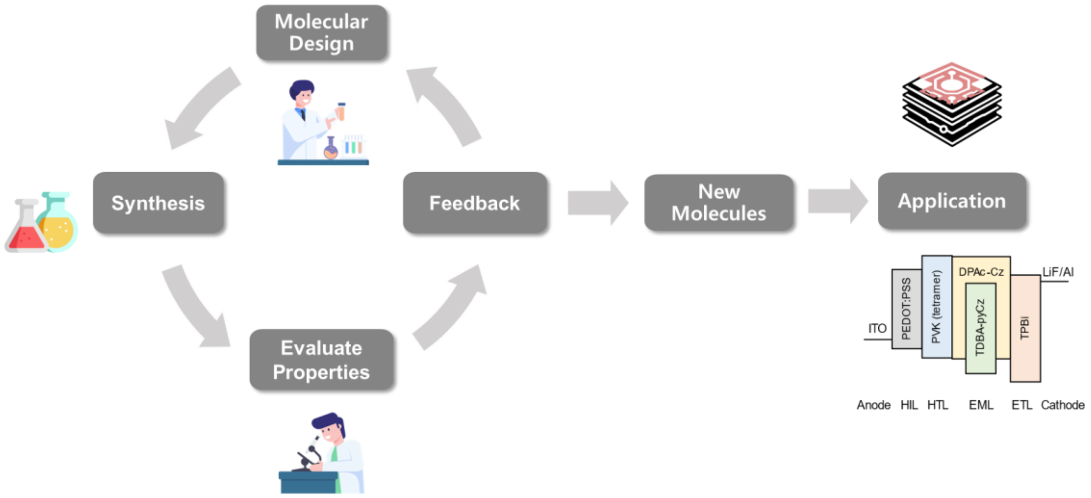

Reliably and quickly predicting molecular properties through deep learning models can accelerate the development process.

We hope that our intention in using this statement is not misleading.

We cited the following papers on page 3 in the revised manuscript:

"……… expert knowledge and trial-and-error methods.[1, 2] "

- Zachary, B. H. *et al.* Design strategies for organic semiconductors beyond the molecular formula, *Nat. Chem.* **4**, 699-704 (2012)

- Turab, L. *et al.* Active learning in materials science with emphasis on adaptive sampling using uncertainties for targeted design, *npj Comput. Mater.* **5**, 21 (2019)

On page 5, line 35, the authors state that they "included only molecules in solutions and host films by excluding the molecules in solid states and gas phases from DB_{Pred}." What is the justification for adding these molecules to DB_{Pred} but excluding them from DB_{Gen}?

Author reply: Our initial Pred-DL model (*JACS Au,* **1**, 427-438 (2021)) was designed to be trained using DB_{Pred} including molecules in solutions and host films, gas phases (molecules without solvents), and solid states (molecules in solvents with the same molecules). However, our Gen-DL model in the current work was trained using the molecules in solutions and host films.

In our Gen-DL model, the initial backbone (or any atom), solvent, and target optical properties

are used as initial inputs. The molecule-solvent interaction is taken into account in our Gen-DL model to generate molecules with target optical properties. For gas phases and solid states, the solvent is not well defined in our Gen-DL model. In principle, the zero matrix can be used as an input for the solvent for the gas phase. However, for the solid state, the solvent is treated identically to the molecule in our Gen-DL model, and thus it is hard to pre-define the solvent before the molecule is finally generated in our Gen-DL model.

In short, our Gen-DL model can be trained with organic molecules in solutions, host films, and gas phases but not with solid states.

When we developed the Gen-DL model, we were rather more interested in generating molecules in solutions and host films considering the molecule-solvent interactions, which seems to be more practically useful in the development process of new molecules.

Finally, on page 12, line 38, the authors compare their work with UV-visible absorption and emission spectra, and mention that the PLQY database is relatively noisy. Could the authors expand more on the noisiness of the PLQY database and the origins of its inaccuracy? These clarifications would greatly enhance the manuscript.

Author reply: In general, the PLQY (photoluminescence quantum yield, 0.0 ~ 1.0) is experimentally measured using either reference molecules (relative PLQY) or integrating spheres (absolute PLQY). Believe or not, both relative and absolute PLQYs are differently measured due to technical inconsistency or skills of researchers, and thus the experimental errors of PLQYs are found to be a lot larger compared to UV-visible absorption and photoluminescence spectra. Such experimental errors of PLQYs are well manifest in the experimental database. The same molecules have different PLQY values reported from different research papers.

To demonstrate this, we searched our experimental database for identical molecule/solvent pairs with different PLQYs and found 273 molecule/solvent pairs. The difference in the PLQY values for the same molecule/solvent pairs was calculated as shown in Figure below,

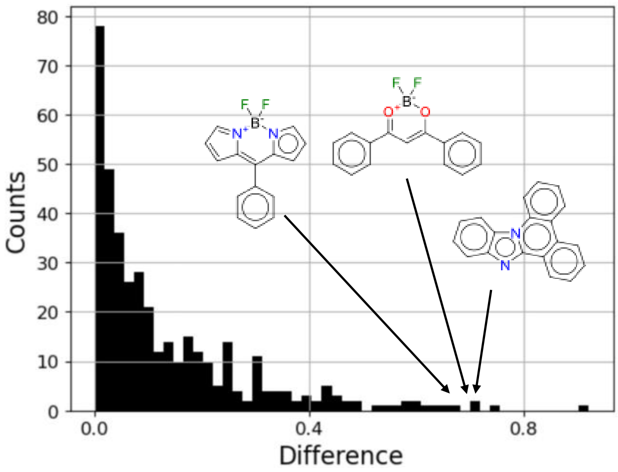

Three examples:

1. Benzimidazole 9a in DCM, PLQY = 0.14, *J. Org. Chem.* **80**, 11, 5753–5763
   3aa in DCM, PLQY = 0.85, *Org. Lett.* **16**, 7, 1872–1875

2. 1,3-diketoboronates 12 in ACN, PLQY = 0.1, *Journal of Photochemistry*, **32**, 2, 177-189
   BF2-1 in ACN, PLQY = 0.8, *Dyes and Pigments*, **175**, 108176

3. 1c in DCM, PLQY = 0.05, *Org. Lett.* **22**, 19, 7694–7698
   BODIPY 1d in DCM, PLQY = 0.74, *J. Org. Chem.* **84**, 9, 5078–5090

As shown in the above examples, the PLQY values of the same molecule/solvent pairs in different research papers have been reported quite differently.

To train DL models in this work, we used the average PLQY value when multiple PLQY values were reported for the same molecule/solvent pair.

We cited the following papers on page 12 in the revised manuscript.

"……. the PLQY database is relatively noisy.[42, 43] "

- Felix, F and Sebastian, R, Statistical treatment of Photoluminescence Quantum Yield Measurements, *Sci. Rep.* **9**, 15638 (2019)

- Albert, M. B, Standards for photoluminescence quantum yield measurements in solution (IUPAC Technical Report), *Pure Appl. Chem.* **83**, 2213 (2011)

Additional Questions:

Quality of experimental data, technical rigor: High

Significance to chemistry researchers in this and related fields: High

Broad interest to other researchers: Top 5%

Novelty: High

Is this research study suitable for media coverage or a First Reactions (a News & Views piece in the journal)?: No

Reviewer: 2

Recommendation: Reconsider after major revisions noted.

Comments:
The manuscript reports a supervised generative model that can generate molecules that satisfy multiple optical properties (e.g. absorption peak, bandwidth, extinction coefficient). While the distribution of final properties that are predicted is fairly broad, the model seems to be able to tune properties within the domain that is represented by the training dataset. Both generative models and optical property prediction models already exist and have previously been reported, but the proposed approach enables the simultaneous optimisation of multiple properties, which is an advance. The approach may be highly useful in identifying candidate molecules for specific optical/electronic properties. The manuscript leaves many technical questions open, which I raise below. These need to be addressed before the manuscript can be considered for publication.

Author reply: We appreciate your recognition of the advancement of our approach to generate molecules with simultaneous optimization of multiple optical properties. We have responded to each comment below to address the issues raised by the reviewer.

- Access to the model is provided via a webpage, however, access to the trained model weights and biases is not provided. Are the datasets available? Is the model publicly available?

Author reply: We uploaded the source code and optimized parameters of the Gen-DL model to GitHub (https://github.com/spark8ku/DeepMoleculeGen) and made them available to the public. We are currently preparing a manuscript to submit our experimental database to *Scientific Data* for publication. For the time being, we will make our experimental database available for academic purposes upon request. In fact, our current Gen-DL model can be trained using the experimental database published in *Sci. Data*, 7, 295 (2020) (https://www.nature.com/articles/s41597-020-00634-8). Since our experimental database (~20,000 datapoints) was published *Sci. Data*, 7, 295 (2020), it has been significantly expanded to ~ 80,000 datapoints.

We added the following section (Data Availability Statement) in the revised manuscript:

Data Availability Statement

Our Gen-DL model is publicly available as a web-based application (http://deep4chem.korea.ac.kr/DeepMoleculeGen). The codes and data for implementing our Gen-DL model are available at https://github.com/spark8ku/DeepMoleculeGen. The

experimental database can be available from https://www.nature.com/articles/s41597-020-00634-8 or the corresponding author upon request for academic purposes only.

- The model is only trained for 200 epochs as it otherwise produces too few new molecules and mostly reproduces the molecules in the training dataset. The plots in Fig. S8 only show training up to 500 epochs, which are also relatively few. What happens if the model is trained substantially longer? At what percentage of M_new does it saturate? If that percentage is too low, then maybe changing the model architecture would be a better strategy than truncating the training early.

Author reply: In Figure S8 of the original version, the y-scale is shown from ~ 40 % to 100 %. In this plot, the performance of our Gen-DL model seems a bit misleading. If Figure S8 is plotted on the full scale of the y-axis from 0% to 100% without the counts of M_{TOP}, it looks like this:

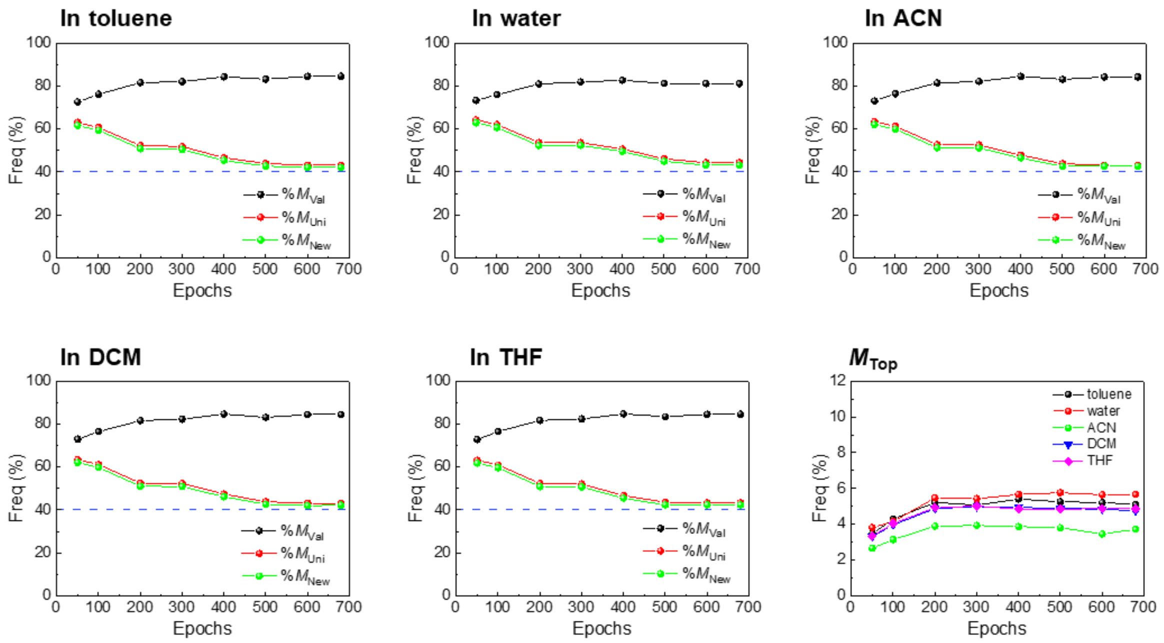

Figure S8. Performance metrics of the Gen-DL model depending on the epoch. 40% is indicated by blue dashed lines.

After training for 200 epochs, the Gen-DL model was found to generate molecules with $\%M_{\mathrm{Val}}$ = ~88.9 %, $\%M_{\mathrm{Uni}}$ = ~47.1 %, $\%M_{\mathrm{New}}$ = ~44.4 %, and $\%M_{\mathrm{TOP}}$ = ~4.4 % (see the SI for details).

As requested by the reviewer, we trained the Gen-DL model more, but it took much longer than expected. During the revision, we were able to train the Gen-DL model up to 680 epochs. After training for 680 epochs, $\%M_{\mathrm{New}}$ is still over 40 % and appears to be saturated.

Accordingly, we replaced Figure S8 with the above figure in the SI.

- The generative model was only trained on molecules in solutions and host films and molecules in solid state and gas phase were excluded. It should be explained why that was done. Does that mean that the remit of the model is restricted to molecules in solution? This should be explicitly stated.

Author reply: Our initial Pred-DL model (*JACS Au,* **1**, 427-438 (2021)) was designed to be trained using DB_{Pred} including molecules in solutions and host films, gas phases (molecules without solvents), and solid states (molecules in solvents with the same molecules). However, our Gen-DL model in the current work was trained using the molecules in solutions and host films.

In our Gen-DL model, the initial backbone (or any atom), solvent, and target optical properties are used as initial inputs. The molecule-solvent interaction is taken into account in our Gen-DL model to generate molecules with target optical properties. For gas phases and solid states, the solvent is not well defined in our Gen-DL model. In principle, the zero matrix can be used as an input for the solvent for the gas phase. However, for the solid state, the solvent is treated identically to the molecule in our Gen-DL model, and thus it is hard to pre-define the solvent before the molecule is finally generated in our Gen-DL model.

In short, our Gen-DL model can be trained with organic molecules in solutions, host films, and gas phases but not with solid states.

When we developed the Gen-DL model, we were rather more interested in generating molecules in solutions and host films considering the molecule-solvent interactions, which seems to be more practically useful in the development process of new molecules.

- Both predictive model and generative model have been tested on the same dataset. The generative model has been trained on labels partially generated with the property prediction model. The predictive model shows significantly worse prediction for the test set (see Fig. S2). What is the performance of Pred-DL for the generated molecules? Is it clear that the pred model still works for the generated molecules? Has this been validated? As it is trained on experimental data, how would one validate the prediction? The model might deteriorate and therefore guide the molecule generation into the wrong direction. I am not sure how the whole workflow can be validated. The manuscript doesn't show convincing evidence that the generated molecules actually have the properties that the predictive model assigns them.

Author reply: The RMSE (root mean squared error) of our Pred-DL model was presented in in Table S1.

Table S1. Performance of the Pred-DL model trained with the current experimental DB

	Training dataset		Test dataset	
Property	R^2	RMSE	R^2	RMSE
λ_{abs}	0.990	11.9 nm	0.922	26.6 nm
σ_{abs}	0.968	287.4 cm^{-1}	0.879	630 cm^{-1}
log ε	0.975	0.080	0.872	0.164
λ_{emi}	0.986	11.8 nm	0.946	18.3 nm
σ_{emi}	0.958	220.5 cm^{-1}	0.856	495 cm^{-1}
Φ	0.960	0.052	0.846	0.127
log τ	0.963	0.099	0.876	0.246

In Table S1, the RMSE (root mean squared error) of our Pred-DL model on the test dataset is shown to be within the acceptable range, indicating that the Pred-DL model can make reasonable predictions. When our Pred-DL model was reported (*JACS Au*, 1, 427-438 (2021)), it was shown to reliably predict optical properties of organic molecules with various backbone structures covering a wide range of wavelengths from UV to near IR. The performance of our Pred-DL model was compared with that of DFT calculations (B3LYP/6-31G(d)). 131 molecules were randomly selected from our experimental DB and their absorption and emission wavelengths were predicted by the Pred-DL model and DFT and TD-DFT calculations (B3LYP/6-31G(d)). The performance of our Pred-DL model was found to be better as shown in Figure below (Figure S5 in *JACS Au*, 1, 427-438 (2021)),

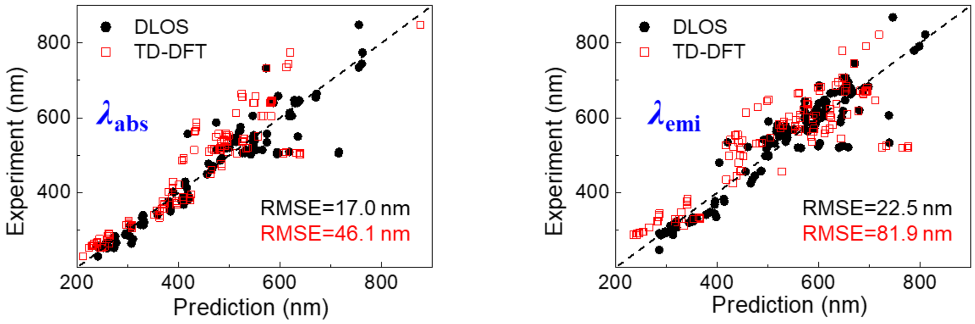

Therefore, our Pred-DL model was used to predict the optical properties of the molecules generated by the Gen-DL model and the prediction accuracy was assumed to be within the prediction errors in Table S1.

Furthermore, we used a test dataset (DB_{Test}), which is mutually exclusive with the training dataset ($DB_{Gen\text{-}DL}$), to determine whether the molecules generated by Gen-DL model exhibit the target optical properties. The molecules generated by Gen-DL model were searched in the DB_{Test} and the optical properties of the molecules found in the DB_{Test} were compared with the target optical properties. In Figure 5, the optical properties of generated molecules predicted by the Pred-DL model, target optical properties, and experimental values were compared. The predicted values are close to experimental values within the prediction errors.

We have revised Figure 5 to additionally include predicted values for comparison.

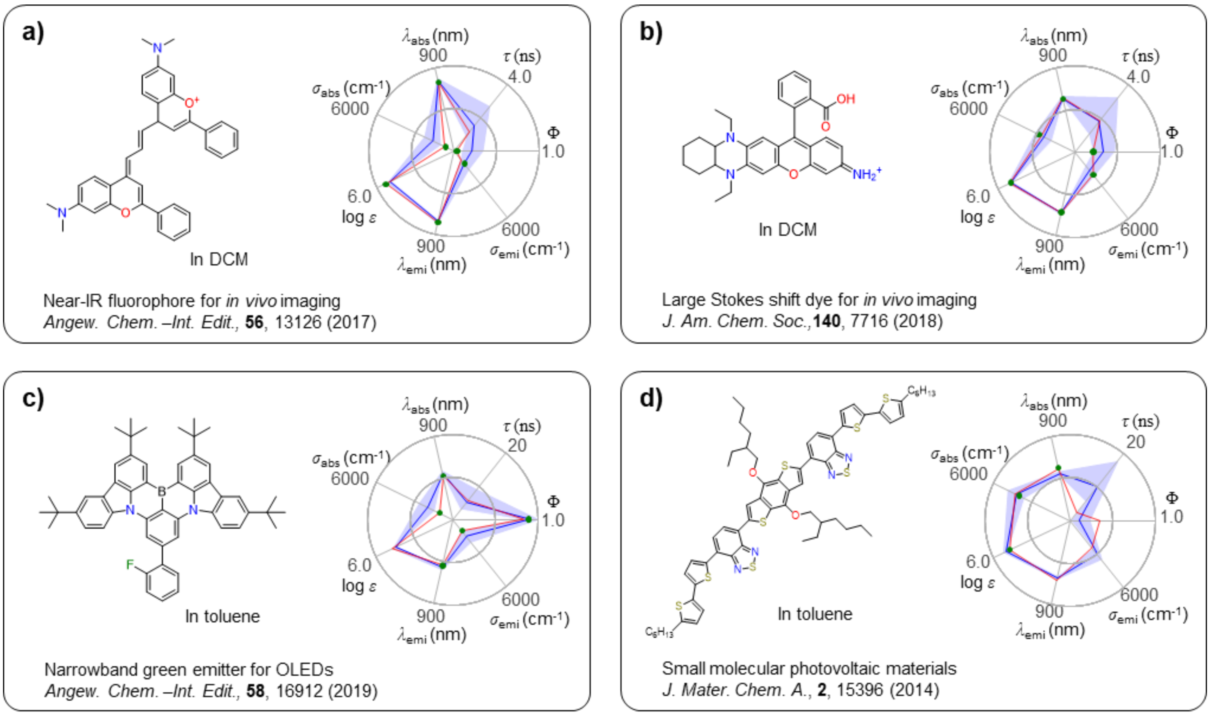

Figure 5. Examples of molecules generated by the Gen-DL model and their practical use. Experimental values (green dots), predicted values (red line), and target values (blue line) are compared in the radar plot. The range of optical properties considered to meet the target optical properties is indicated in shaded blue based on the RMSE of the Pred-DL model.

- The step-by-step model building creates molecules stochastically to optimize the target labels. This includes the termination of the generation process. How will the model truncate the molecule? Will it truncate for certain when the property is within a certain threshold or according to a probability distribution conditioned by that property? Does that mean that the model is biased towards smaller molecules that satisfy the properties compared to larger ones? Consider the following scenario: If the construction starts from large scaffolds and there is also a probabilistic step of removing atoms, will the model create a similar distribution of molecules? If that is not the case, the current choice of molecule building steps creates an uncontrolled bias.

Author reply: Our Gen-DL model generates molecular structures step by step by calculating the probabilities of the next possible actions (addition, connection, and termination), finally producing the molecular structure with the target optical properties (Figure 1). Note that our Gen-DL model does not check the optical properties of intermediate molecular structures. The termination step finalizes the molecular generation process and the molecular weight keeps increasing until the termination is stochastically selected. This guarantees the structural

diversity and a wide range of molecular weight.

When the molecular weight distribution of generated molecules is plotted in Figure below (revised Figure 2a), it is a little bit narrower than that of the training dataset but is sufficiently wide, indicating that the Gen-DL model can generate molecules with a wide range of molecular weights under the given conditions (see Figure below). Accordingly, our Gen-DL model is expected to generate diverse molecular structures including various backbones in the training dataset. However, the stochastic nature of molecular generation process can produce molecules with a new backbone structure.

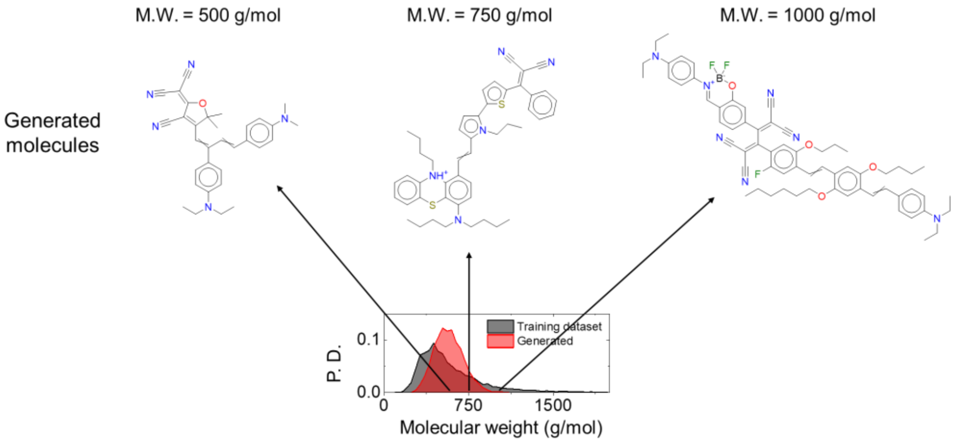

As assumed by the reviewer, in the case where the initial scaffold is very large such that the molecules with target optical properties cannot be generated starting from the large scaffold, the Gen-DL model will generate molecules starting from the scaffold based on three actions (addition, connection, and termination) and the process like removing atoms will not be involved. Accordingly, the generated molecules based on the large scaffold will not meet the target optical properties. In our Gen-DL model, the initial scaffold must be properly given to generate molecules with the target properties.

We have revised Figure 2a to include the molecular weight distribution of generated molecules and molecules in the training dataset.

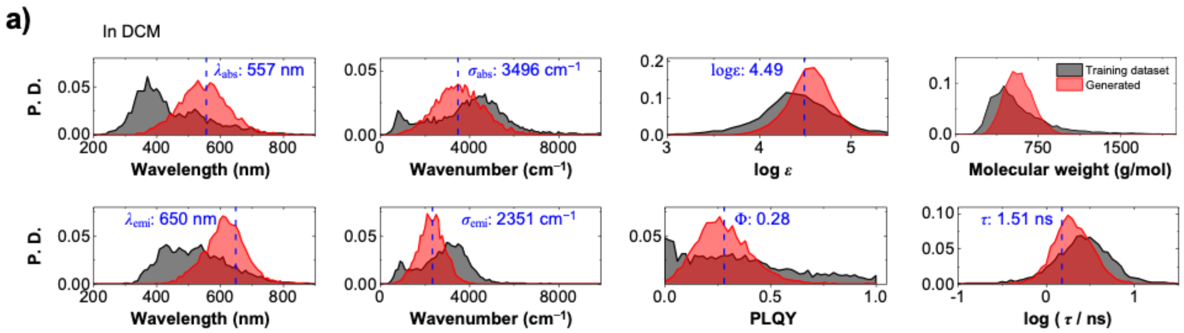

For clarity, we revised the following section on page 7,

"During the molecular generation process, the Gen-DL model generates molecular structures step by step by calculating the probabilities of the next possible actions (addition, connection, and termination), finally producing the molecular structure with the target optical properties (Figure 1)."

- What is the average size of generated molecules compared to the training database? What is the average elemental composition compared to the training database? The current analysis of structural similarity between generated molecules and training database seems insufficient to judge on the viability and robustness of the algorithm. The t-SNE analysis based on Morgan fingerprints does not present clear clustering and t-SNE can be misleading in the way how it is used here. In addition, Morgan fingerprints do not sufficiently discriminate molecules, which is evident from the fact that the t-SNE doesn't more clearly separate classes of molecules.

Author reply: The molecular weight distribution of 10,000 molecules generated under given conditions (solvent = toluene, target properties = [λ_{abs} = 350 nm, σ_{abs} = 4819 cm^{-1}, logε = 4.26, λ_{emi} = 452 nm, σ_{abs} = 3576 cm^{-1}, PLQY= 0.34, τ = 2.2 ns]) is compared with that of the training dataset in Figure below (yellow background). The molecular weight distribution of the generated molecules is found to be slightly narrower than that of the training dataset, and the average molecular weight is also smaller than that of the training dataset. However, the molecular weight distribution of the generated molecules is sufficiently wide, indicating that the Gen-DL model can generate molecules with a wide range of molecular weights under the given conditions. This also indicates that generated molecules may contain a variety of molecular backbones, ranging from small to large molecular weights (up to ~1584 g/mol in Figure below). The molecular weight distribution of molecules is added to the revised Supporting Figures (Figure S9-S113).

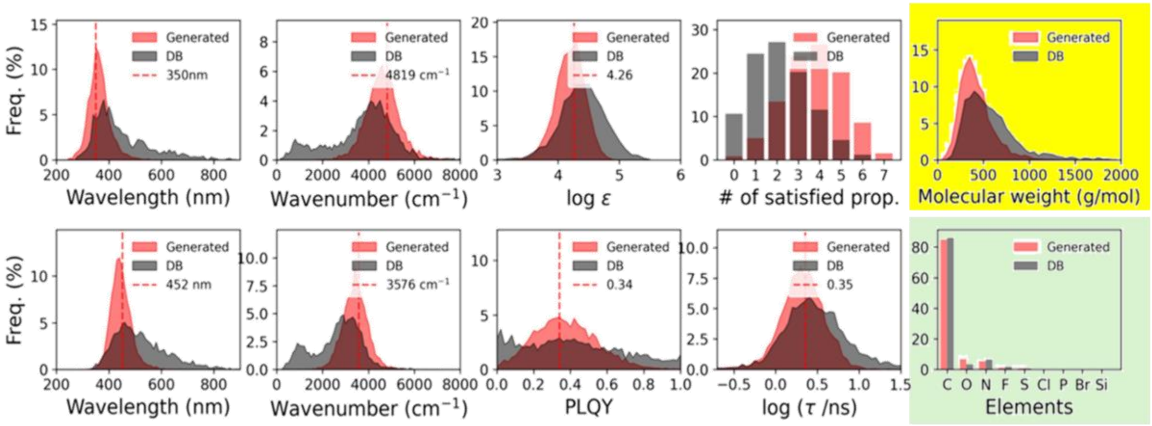

Revised Figure S9(a)

In addition, the elemental composition of generated molecules is compared with that of the training dataset in Figure above (green background). The elemental composition of generated

molecules and the training dataset are found to be very similar. The elemental composition of molecules is added to the revised Supporting Figures (Figure S9-S113)

We used the *t*-SNE analysis based on Morgan fingerprints to examine the *structural diversity* of generated molecules compared to the training dataset. Given the initial conditions, the Gen-DL model can generate molecules with diverse backbone structures, so the generated molecules are expected to be widely distributed in the *t*-SNE plot without local clustering. As shown in Figure 4d, the lack of clear local clustering in the *t*-SNE plot indicates that our Gen-DL model can generate diverse molecular structures that exhibit the same target properties. This structural diversity with the same target properties is a key strength of our Gen-DL model.

- The generative model is a supervised model and all generated molecules show properties in the same space as the original database. Is it possible to predict molecules with this approach with properties outside of the domain covered by the labels? Can it be assessed if both models are predicting within or outside of the training distribution? How would the user know when predictions cannot be trusted?

Author reply: As the reviewer is well aware, the performance of DL models depends on the amount and accuracy of the database. Our experimental database (71,424 molecule/solvent pairs) includes various molecules in terms of diverse molecular backbones, a wide range of molecular weights (over ~2000 g/mol), and a wide range of absorption and emission wavelengths covering from UV to NIR.

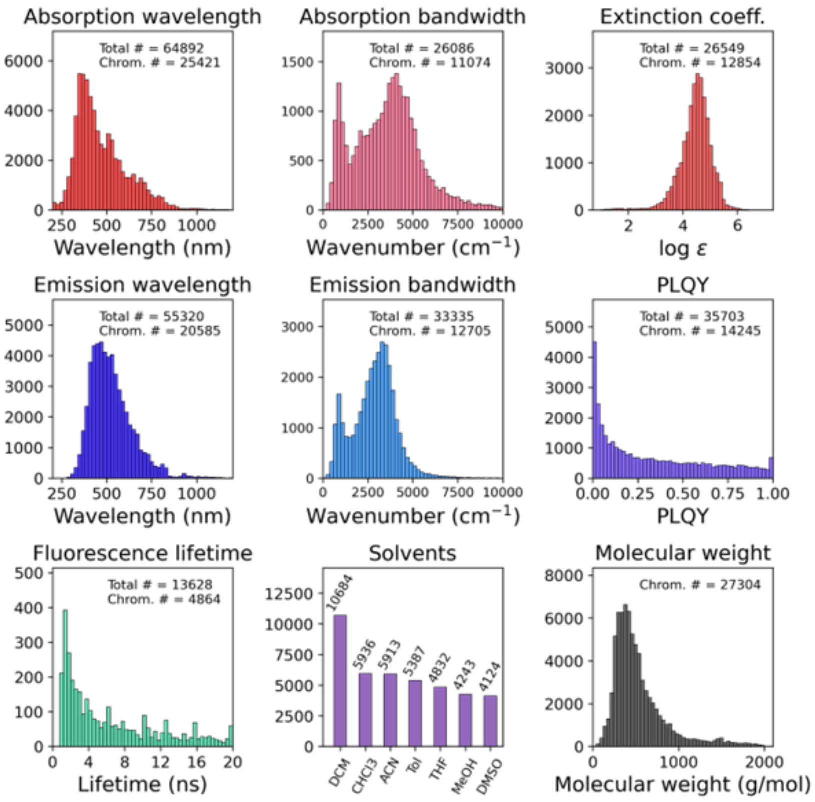

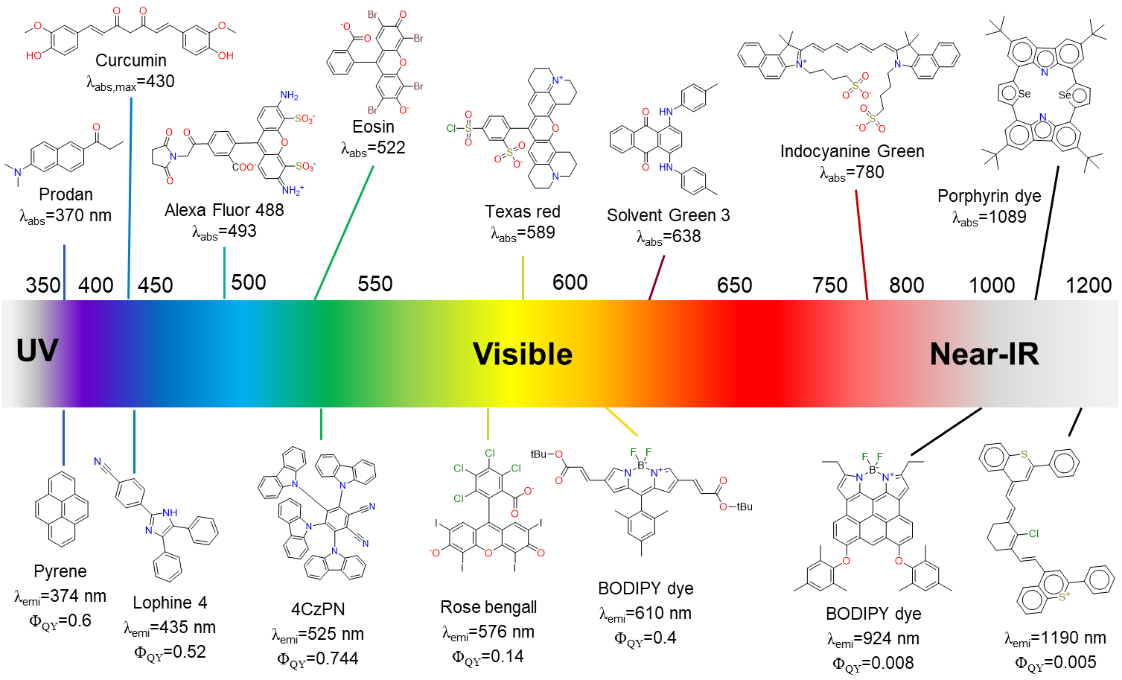

Both Pred-DL and Gen-DL models were trained using this experimental database. The Pred-DL model was shown to predict the optical properties of molecules in the domain of the training dataset within the prediction error (Test dataset) in Table S1. However, for molecules with completely new backbones (outside the domain of the training dataset), the prediction accuracy of our Pred-DL model can still be reliable within the prediction error unless the new molecules are based on completely new backbones. This is an intrinsic issue of all DL models.

Likewise, our Gen-DL model primarily generates molecules in the domain of the training dataset and it can also generate molecules outside the domain of the training dataset due to the stochastic nature of the molecular generation process. When new molecules outside the training dataset are stochastically generated by the Gen-DL model, the predicted values for the optical properties of the new molecules could still be reliable within the prediction error unless the new molecules are based on completely new backbones.

It is very challenging to assess whether both models are predicting within or outside the domain of the training dataset. In principle, it could be possible if the uncertainty of DL models was estimated. If the uncertainty is relatively high, prediction can be considered outside the domain and vice versa. The Bayesian dropout could be incorporated into our Pred-DL model to estimate the uncertainty of predicted values. This is beyond the scope of this work. Thank the reviewer for this valuable comment.

- It is stated that the "Gen-DL" model generates more hydrophilic molecules in water than in toluene. Why does it do that? This needs to be understood.

Author reply: The optical properties of molecules depend on their local environments. In other words, the molecules exhibit different absorption and emission spectra in different solvents. In this work, we designed the Gen-DL model to generate molecules considering molecule-solvent interactions (Figure S5 and S6). To verify this, we used the Gen-DL model to generate molecules in water and toluene and compared log *P* values of molecules generated in water and toluene in Figure 4a. A larger log *P* value indicates that the molecule is more hydrophobic. Our Gen-DL model is expected to generate hydrophilic molecules more in water than in organic solvents. As we expected, hydrophilic molecules are more generated in water than in toluene (Figure 4a). Two examples of generated molecules are shown in Figure 4a. The molecule on the left is hydrophilic but the molecule on the right is hydrophobic.

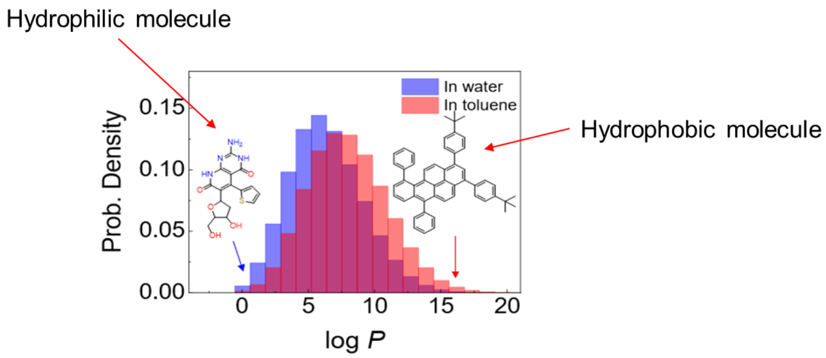

In fact, when the log *P* values of molecules in water and toluene in the training dataset are plotted in Figure below. the molecules in water have smaller log *P* values than those in toluene.

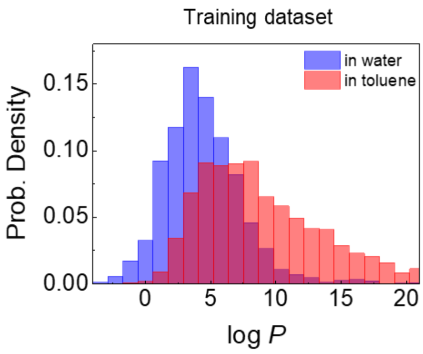

Our Gen-DL model seems to learn this molecular feature implicitly from the training dataset.

We revised the following section on page 13 in the revised manuscript:

"By properly reflecting solvent effects, our Gen-DL model is expected to generate more hydrophilic molecules in water than in organic solvents. In Figure 4a, the distribution of the log *P* values of the molecules generated in water shows a tendency toward smaller log *P* values compared with the molecules generated in toluene. This indicates that the Gen-DL model reflects solvent effects when generating molecules in different solvents."

- Page 14, It is stated that the solvent effects are appropriately reflected by the Gen-DL model based on the Stokes shift trends. The arguments seem very handwavy.

Author reply: The Stokes shift is another feature to examine the solvent effects on the optical properties of molecules. For the same molecule, the Stokes shift is larger in highly polar solvents than in weakly polar solvents. In Figure 4b, we compared the Stokes shift of molecules generated in acetonitrile (ACN, $\varepsilon = 37.5$, highly polar) and toluene ($\varepsilon = 2.38$, weakly polar). The molecules generated in ACN are expected to exhibit larger Stokes shifts than those in toluene. The molecule in the inset shows the Stokes shift of 80 nm in toluene and 187 nm in ACN. Accordingly, the solvent effects are appropriately reflected by the Gen-DL model.

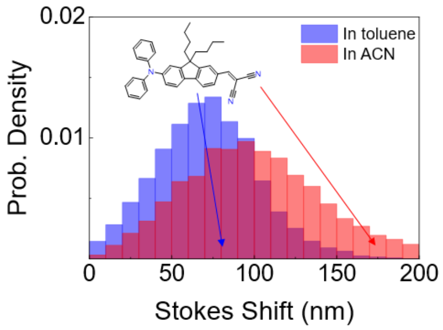

We revised the following section on page 15 in the revised manuscript:

"Figure 4b shows the distributions of the Stokes shifts (λ_{emi} minus λ_{abs}) for the same molecules in toluene ($\varepsilon = 2.38$, weakly polar) and acetonitrile (ACN, $\varepsilon = 37.5$, highly polar)."

Additional Questions:

Quality of experimental data, technical rigor: High

Significance to chemistry researchers in this and related fields: Top 5%

Broad interest to other researchers: High

Novelty: High

Is this research study suitable for media coverage or a First Reactions (a News & Views piece in the journal)?: No
